# Characterization of a Molecular Clone of Deformed Wing Virus B

**DOI:** 10.3390/v16060980

**Published:** 2024-06-18

**Authors:** Sandra Barth, Sebastian Affeldt, Claudia Blaurock, Irmin Lobedank, Anette Netsch, Kerstin Seitz, Till Rümenapf, Benjamin Lamp

**Affiliations:** 1 Institute of Virology, Faculty of Veterinary Medicine, Justus-Liebig-University Giessen, Schubertstrasse 81, 35392 Giessen, Germany; sandra.barth-2@vetmed.uni-giessen.de (S.B.); sebastian.affeldt@vetmed.uni-giessen.de (S.A.); claudia.blaurock@vetmed.uni-giessen.de (C.B.); irmin.lobedank@vetmed.uni-giessen.de (I.L.); anette.netsch@vetmed.uni-giessen.de (A.N.); 2 Institute of Virology, Department for Pathobiology, University of Veterinary Medicine, Veterinaerplatz 1, 1210 Vienna, Austria; k.seitz@lk-oe.at (K.S.); till.ruemenapf@vetmeduni.ac.at (T.R.)

**Keywords:** deformed wing virus, molecular virus clone, Iflavirus, reverse genetics, DWV-B, 5′-UTR, virulence, master variant B

## Abstract

Honey bees (*Apis mellifera*) play a crucial role in agriculture through their pollination activities. However, they have faced significant health challenges over the past decades that can limit colony performance and even lead to collapse. A primary culprit is the parasitic mite *Varroa destructor*, known for transmitting harmful bee viruses. Among these viruses is deformed wing virus (DWV), which impacts bee pupae during their development, resulting in either pupal demise or in the emergence of crippled adult bees. In this study, we focused on DWV master variant B. DWV-B prevalence has risen sharply in recent decades and appears to be outcompeting variant A of DWV. We generated a molecular clone of a typical DWV-B strain to compare it with our established DWV-A clone, examining RNA replication, protein expression, and virulence. Initially, we analyzed the genome using RACE-PCR and RT-PCR techniques. Subsequently, we conducted full-genome RT-PCR and inserted the complete viral cDNA into a bacterial plasmid backbone. Phylogenetic comparisons with available full-length sequences were performed, followed by functional analyses using a live bee pupae model. Upon the transfection of in vitro-transcribed RNA, bee pupae exhibited symptoms of DWV infection, with detectable viral protein expression and stable RNA replication observed in subsequent virus passages. The DWV-B clone displayed a lower virulence compared to the DWV-A clone after the transfection of synthetic RNA, as evidenced by a reduced pupal mortality rate of only 20% compared to 80% in the case of DWV-A and a lack of malformations in 50% of the emerging bees. Comparable results were observed in experiments with low infection doses of the passaged virus clones. In these tests, 90% of bees infected with DWV-B showed no clinical symptoms, while 100% of pupae infected with DWV-A died. However, at high infection doses, both DWV-A and DWV-B caused mortality rates exceeding 90%. Taken together, we have generated an authentic virus clone of DWV-B and characterized it in animal experiments.

## 1. Introduction

The European honey bee (*Apis mellifera* Linnaeus, 1758) is an essential pollinator for numerous agricultural crops, ranking among the most crucial domesticated animals globally [1]. Direct bee products such as honey and wax are prized but pale in significance compared to the indispensable role honey bees play in agricultural pollination.

The global bee population struggles to match the increasing need for pollination services due to various threats to their health [2,3]. Numerous factors affect the health of bee colonies, including poor nutrition because of industrialized agricultural practices, with large-scale monocultures requiring the use of herbicides and pesticides. However, the ectoparasitic mite Varroa destructor (Anderson and Trueman, 2000) and the RNA viruses it spreads are the most critical factors to colony health and regularly lead to colony collapses [3,4].

Originally, the mite infested colonies of the Eastern honey bee (*A. cerana* Fabricius, 1793). In the 19th century, it shifted to the European honey bee, adapting to this novel host [5]. While *A. cerana* had developed effective defense mechanisms through host–parasite co-evolution, the European honey bee was caught off guard, leading to an unprecedented collapse of feral honey bee populations in the northern hemisphere and substantial losses in commercial beekeeping [6,7]. Bees exhibit several behavioral traits (such as grooming, cleaning, re-capping, and removal of infested brood) as well as genetic factors (such as timing of emergence and infertility of mites) that confer resistance to mites, with *A. cerana* exhibiting a much more successful phenotypic expression, as reviewed in [8]. The mite directly harms its host by feeding on its hemolymph and fat body while also acting as a vector for various pathogenic viruses [9,10]. Miticide treatments are essential for honey bee colony survival [11], yet no remedy exists for the viruses, leaving colonies vulnerable to regular damage [12]. Consequently, mites and viruses stand as the primary culprits behind overwintering losses in honey bees, posing a significant economic threat [13,14]. The most important bee virus transmitted by Varroa mites is certainly deformed wing virus (DWV). Clinical signs of DWV infections closely correlate with Varroa infestation rates, serving as an indicator of subsequent damage or even colony collapse.

DWV belongs to the *Picornavirales* order, is grouped within the *Iflavirus* genus, and is classified within the *Iflaviridae* family [15]. Its non-enveloped virions have an icosahedral symmetry, measuring around 30 nm in diameter [16,17]. The genome, consisting of single-stranded RNA of positive polarity, harbors a single open reading frame (ORF) encoding a polyprotein spanning 2893 amino acids. The organization of the picornavirus-like polyprotein, which is post-translationally processed by a 3C-like protease, was first characterized by Lanzi et al. [15]. The first protein in the ORF is a so-called leader protein, which is followed by the viral structural proteins in the P1 cassette (VP1, VP2, VP3, and VP4). The typical replication enzymes in the P2 and P3 cassettes, such as helicase (2C-like), protease (3C-like), and RNA-dependent RNA polymerase (3D-like), have already been identified by Lanzi et al., along with their potential processing sites [15]. Only recently, Yuan et al. (N-terminus of 3C-like protease) and our group (N-terminus 3C-like protease and N-terminus 3D-like polymerase) uncovered the precise processing of the 3CL/3DL region [18,19].

Like other RNA viruses, the DWV genome is highly diversified, leading to the classification of four master variants (DWV-A, DWV-B, DWV-C, and DWV-D) [20,21,22]. DWV-B, also known as Varroa destructor virus-1 (VDV-1) [23], and DWV-D, initially termed Egypt bee virus, were re-named following this classification [22].

While DWV-A and -B are widespread in the field and usually prevalent with high titers, DWV-D has disappeared [24]. DWV-C infections, on the other hand, have been frequently observed in the UK and USA, generally presenting lower titers [25]. The master variants are all linked to disease and/or colony collapse. As a plus-strand RNA virus, DWV forms so-called quasi-species in the host, which, because of error-prone replication, present themselves as an array of diverse genomes clustered around a consensus sequence. At the same time, recombinants of the master variants DWV-A and DWV-B were described, whose transmission is favored by the Varroa mite, when the structural protein genes of DWV-B were recombined with the nonstructural protein genes of DWV-A [26,27]. The virulence of each master variant remains a hotly debated topic, with conflicting findings arising in different field and laboratory studies. In particular, the question of whether DWV-A or DWV-B is the more virulent variant has not yet been solved. Laboratory experiments with adult honey bees have suggested that DWV-B demonstrates greater virulence [28], while field observations and experimental data indicate that DWV-B outcompetes DWV-A, reaching higher titers [29,30]. However, when honey bee pupae, the stage where disease symptoms manifest, are experimentally inoculated, DWV-B appears to exhibit lower virulence and mortality compared to DWV-A, despite, again, higher RNA loads in DWV-B-infected pupae [31]. Interpreting data from field studies proves challenging due to the prevalence of multiple virus infections in bee colonies, with the individual pathogens’ influences often remaining ambiguous [32,33]. Moreover, laboratory studies encounter obstacles due to the lack of virus-free bee cell lines and defined virus-free “laboratory bees”, increasing the risk of contamination with both known and unknown pathogens when isolating viruses and producing virus stocks for experiments [34,35].

To address these challenges, we previously devised a reverse genetic system for DWV-A (strain 1414, GenBank KU847397), facilitating precise mono-infections and controlled laboratory experiments [36]. Additionally, in 2018, we developed a genetically tagged DWV expressing GFP as a reporter gene and filed a patent for DWV-A as a viral vector for transgene expression in honey bees (patent WO 2019063789A1, https://worldwide.espacenet.com/patent/search/family/060021911/publication/WO2019063789A1?q=WO%202019063789A1 (accessed on 17 May 2024)). Later, synthetic constructs for the master variants DWV-A (GenBank NC_004830.2) and DWV-B (GenBank AY251269.2), alongside DWV-A/-B recombinants (GenBank HM067438.1), were presented by another group [37]. These viruses were generated by molecular cloning techniques paired with in vitro gene synthesis using the established 5′-end UTR sequence of DWV-A, strain 1414. No significant differences in the replication rates or virulence among the two DWV variants or recombinants thereof were found when infecting bee pupae. Moreover, five independent molecular clones of DWV-A were generated through a nearly full-length RT-PCR in combination with the established 5′-end UTR sequence of strain 1414 [38]. Infectious DWV clones significantly advanced our understanding of DWV infections in honey bees, proving Koch’s postulates and shedding light on recombination events, the emergence of quasi-species post-infection, and DWV virulence. Since molecular analyses often rely on defined virus clones, we generated a typical DWV-B clone and compared it with our DWV-A clone. First, we selected a prevalent strain of DWV-B, closely related to the type strain VDV-1 (GenBank AY251269), and analyzed its 5′-end. We found that the authentic 5′-end sequence of DWV-B is indeed identical to that of our DWV-A clone and generated a molecular clone from a single viral RNA molecule via full-length RT-PCR. The comparative characterization of this virus clone confirms previous findings indicating higher RNA replication rates and lower virulence of the DWV-B clone in the pupal stage compared to the established DWV-A clone.

## 2. Materials and Methods

### 2.1. Honey Bees

The honey bees (*A. mellifera carnica*) utilized in this study were provided by the apiary of the Institute of Virology, Justus-Liebig University Giessen (Hesse, Germany), under the authorization of the owner. Experimental animals were only taken from healthy bee colonies. Brood samples were tested and found to be free of sacbrood virus (SBV), acute bee paralysis virus (ABPV), chronic bee paralysis virus (CBPV), and deformed wing virus (DWV), as previously described [39]. Uniform-age worker bee pupae were selected from individual combs for the experiments. Only pupae displaying characteristic violet to blue ocular pigmentation, indicative of a developmental stage between 13 and 15 days post-oviposition, were included in the studies. Each pupa was carefully transferred from its capped brood cell to a well within a sterile 24-well plate by employing sterile steel tweezers. A pre-screening procedure was implemented to ensure the exclusion of deceased or compromised individuals, involving an overnight incubation at 33 °C in a humidity-controlled environment, followed by microscopic examination before the start of the experiments. All necessary regulatory approvals were obtained for the genetic manipulation of the pathogens (IV44-53r 30.03 UGI109.12.01), and all infection studies were conducted in accordance with established biosafety and containment level 2 (BSL2/S2) protocols.

### 2.2. Bee Cell Culture

Primary bee cells were harvested from the thoracic segment of pink-eye pupae. Initially, the abdominal segment was opened and washed twice with 300 µL of Schneider’s Drosophila medium (Thermo Fisher; Waltham, MA, USA) for the removal of intestinal and other abdominal and thoracic tissues. The pupae were then incised at the thoracic segment. Viable cells suitable for cultivation were obtained from a final intensive rinse of this segment. The remains of the pupa were then utilized to prepare RNA, which was tested for DWV using RT-PCR. Cells were transferred to individual wells of 48-well cell culture plates and cultured in a bee cell medium containing 79% Schneider’s Drosophila medium, 10% FBS (Capricorn Scientific; Ebsdorfergrund, Germany), 1% Penicillin/Streptomycin (Thermo Fisher), and 10% pre-conditioned medium. The pre-conditioned medium was obtained from S2-cell cultures (Catalog number R690-07; Invitrogen—Life Technologies; Darmstadt, Germany) grown in Schneider’s Drosophila medium. The bee cells were cultivated in a humidified incubator at 33 °C. After 48 h of incubation, naïve cells from uninfected pupae and the DWV-infected cells from pupae transfected with in vitro-synthesized RNA were analyzed using a sensitive fluorescence microscope.

### 2.3. Viruses

Crippled emerging worker bees were collected in September 2022, with the owner’s consent, from a colony burdened with a high Varroa load at the University of Veterinary Medicine (Vienna, Austria). DWV infection was confirmed through an ELISA test, now available commercially as a lateral flow device (FASTest Bee 3T; MEGACOR; Lindau, Germany). The virus was isolated from the original homogenized sample pool of ten emerged bees (passage 0, P0) by injecting naïve bee pupae (passage 1, P1). The DWV strain was sequenced using RT-PCR amplicons and identified as DWV-B (GenBank PP418870). It was designated as DWV-B, strain Austria-SB22, named after the country of origin, the initials of the collector and identifier (Seitz and Barth), and the year of isolation. The virus stocks P0 and P1 were also tested for SBV, ABPV, and CBPV by RT-PCR, as previously described (39). It was found that the virus isolate P0 already contained SBV as a second pathogen, which limited its use for defined infection studies.

The infectious clonal virus was produced using the transfection of in vitro-transcribed RNA, just as described earlier [36]. Briefly, plasmid DNA was linearized for run-off transcription (NotI-HF; NEB; Ipswich, MA, USA), purified (Monarch PCR and DNA Cleanup Kit; NEB), and used as a template for in vitro transcription (SP6; NEB). We further purified the RNA (RNeasy; Qiagen; Hilden, Germany) and quantified it (Qubit; Life Technologies). A quantity of 1 µg of transcribed RNA in 1 µL of inoculum was used per transfection, which corresponds to approximately 1.73 × 10^11^ RNA molecules. However, it must be considered that, in addition to complete infectious genomes, short, prematurely terminated transcripts also occur. Sequence motifs similar to the SP6 RNA polymerase termination signal can cause this premature termination, which has been shown previously [36,40]. The transcribed RNA was injected into the lower part of the pupae’s thoracic segments before the pupae were further incubated at 33 °C. We have refined our transfection technique since the initial publication on DWV-A reverse genetics. We no longer use Hamilton pipettes for bee injections but have switched to gel-loading tips (04RD200, Kisker Biotech, Steinfurt, Germany) coupled with a piston-operated pipette. Utilizing tips provides three key advantages: decreasing injuries to experimental animals by removing the necessity for a metal needle, guaranteeing sterility, and enhancing injection volume control. After the indicated timepoints, the pupae were harvested by freezing and stored at −80 °C. For molecular analyses, the pupae were thawed and homogenized in PBS containing 0.5% Triton X-100 (Carl Roth, Karlsruhe, Germany). For virus passaging, we thawed and homogenized the pupae in PBS only to avoid the toxic effects of the tenside. All virus stocks were sterile-filtered (0.45 µm, Minisart RC4; Satorius, Göttingen, Germany) to exclude bacterial contamination.

### 2.4. RT-PCR and Genome Equivalent Quantification

RNA extraction was performed from whole pupae lysates using the QIAshredder and QIAamp viral RNA Mini Kit on a QIACube workstation (Qiagen). Diagnostic RT-PCR was conducted utilizing the OneTaq One-Step RT-PCR Kit (NEB) with the specified oligonucleotides. For quantitative RT-PCR, the Luna Universal qPCR Master Mix (NEB) was employed, along with the oligonucleotides DWV-B-qRT_fwd and _rev for DWV-B quantification (modified from [26]) and DWV-A-qRT_fwd and _rev for DWV-A quantification. Genome equivalents (GEs) were determined using the 7500 System SDS Software (v2.3, Applied Biosystems, Foster City, CA, USA) based on the standard curve of purified in vitro-transcribed RNA and cDNA. The DWV genome load per bee pupa was extrapolated using the proportion factors from the processing steps. The oligonucleotides utilized in this study are listed in Table 1.

### 2.5. The Generation of a Molecular Clone of DWV-B

To conduct full-genome-length RT-PCR, we determined the 5′-end sequence of the genome via a RACE-PCR assay. The oligonucleotides for the RACE were designed based on the DWV-B reference genome (VDV-1, GenBank AY251269). Following RNA purification, cDNA was generated using oligonucleotide DWV-B-5′-RACE-outer_rev and ProtoScript II Reverse Transcriptase (NEB). The resultant cDNA underwent purification (Monarch PCR and DNA Cleanup Kit; NEB), followed by poly-A tailing, facilitated by terminal transferase (NEB). After ethanol precipitation and drying, the nucleic acids served as templates for PCR. The oligonucleotides Adapter-dt and DWV-B-5′-RACE-outer_rev were employed alongside OneTaq polymerase Mastermix (NEB) to amplify the complete 5′-end sequence of about 840 bp. Subsequently, a nested PCR reaction was initiated using Adapter and DWV-B-5′-RACE-inner_rev oligonucleotides in a OneTaq reaction, yielding a 240 nt amplicon, which was directly sequenced using the reverse primer. The gel purification of this amplicon was also executed (Monarch DNA Gel extraction Kit, NEB), followed by cloning into a T-vector (pGem-T, Promega, Madison, WI, USA) and sequencing utilizing M13 and M13_rev primers at a commercial supplier facility (Microsynth, Balgach, Switzerland). The experiment was replicated using a C-tailing approach and matching oligonucleotides. The validated 5′-end sequence served as the foundation for designing a 5′-end oligonucleotide for genome-length RT-PCR (Figure S1). Additionally, the entire genome was subsequently amplified via RT-PCR using oligonucleotides DWV-B-Seq1 to 15, and a consensus sequence was determined through Sanger sequencing of these amplicons using assay oligonucleotides (Microsynth).

**Figure 1:**
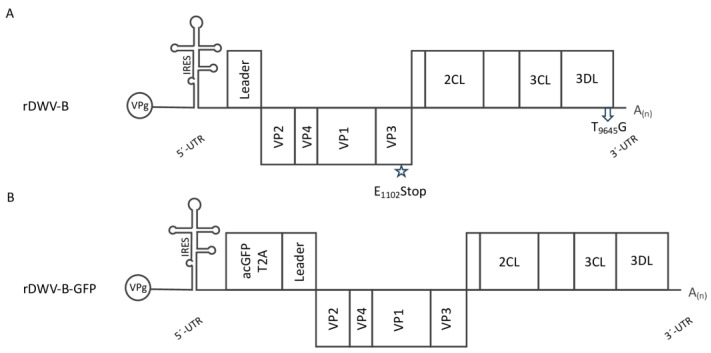
Molecular DWV-B clones used in this study. (**A**) The genome organization of rDWV-B is illustrated. Captions encompass the genome-linked protein (VPg), the 5′-UTR featuring an IRES element, the leader protein gene (Leader), the structural protein gene cassette (VP1-VP4), and the nonstructural protein gene cassette containing annotated functional enzymes (2CL, 3CL, and 3DL), along with so-far-uncharacterized gene products (represented by empty boxes), and the short 3′-UTR with the poly-A-tail (A_(n)_). A star highlights the mutation of one amino acid codon (G_4459_AG to T_4459_AG), resulting in the alteration of E_1102_ to a termination signal (E_1102_Stop). This mutation leads to an interruption of the reading frame, rendering the genome non-replicative. An arrow indicates the synonymous mutation T_9645_G, which disrupts an XhoI restriction enzyme cleavage site without altering the encoded amino acid (G_9643_CT to G_9643_CG). (**B**) The genome organization of rDWV-B-GFP follows a similar structure with corresponding captions. However, it incorporates an additional acGFP gene and a T2A peptide preceding the viral ORF.

Full-length genomic cDNAs of DWV-B were generated through a ProtoScript II reverse transcription reaction employing the oligonucleotide Adapter-dt. Following purification and concentration using a DNA Cleanup Kit (Monarch; NEB), this cDNA served as a template for a high-fidelity PCR reaction (Q5 Hot Start 2x Master Mix; NEB) employing the oligonucleotides Adapter and DWV-5′-end_fwd. A pBR322 vector backbone containing an SP6 promoter was modified by the oligonucleotides DWV-5′-SP6_rev and Adapter-NotI_fwd, providing complementary extensions for DNA recombination (NBBuilder; NEB). The resulting plasmid harboring the cDNA of DWV-B was termed prDWV-B. For validation, all plasmids generated in this study were sent for long-read single-molecule sequencing (Full PlasmidSeq; Microsynth). To provide a negative control, the plasmid prDWV-B was amplified with the oligonucleotides DWV-B-Stop_fwd and _rev. The purified PCR product was re-circularized via homologous recombination (NEBuilder; NEB). This plasmid, termed prDWV-B-E_1102_Stop, encodes a polyprotein interrupted by a termination codon within the VP3 coding region, thereby preventing the replication of the in vitro-transcribed RNA. An overview of the virus constructs generated and their genome organization is shown in a diagram (Figure 1).

### 2.6. Alignments and Phylogenetic Analyses

The nucleotide sequences of DWV-B strains Austria-SB22 (Austria 2022, PP418870), VDV-1 (Netherlands 2001, NC_006494), ER23 (USA 2018, OR361559), ER24 (USA 2018, OR361560), B11B (Austria 2018, OL803829), 341-1 (Slovenia 2019, ON648741), and VV144I (France 2019, MN565037) were aligned with three DWV-A master variant strains, namely, PA (USA 2003, AY292384), 1414 (Austria 2014, KU847397), and Kakugo (Japan 2015, AB070959). DWV master variants C and D were represented by the strains Devon (UK 2015, European Nucleotide Archive ER5657949) and Egypt bee virus (Egypt 1977, MT504363), respectively. Strain NT-12 (Australia 2014, MG995697) of Darwin bee virus 3 was included in the alignments as an outgroup due to its close relation and available full genomic sequence identified through a BLAST search (https://blast.ncbi.nlm.nih.gov/Blast.cgi (accessed on 13 June 2022)). Multiple sequence alignments were conducted using CLC Genomics Workbench (Version 7.7.1, Qiagen) with default parameters and checked for accuracy. However, all genome regions were aligned correctly without the need for adjustments, which is probably due to the close relationship of all these virus genomes (Figure S2). Evolutionary relationships were determined using the “unweighted pair-group method using arithmetic mean” (UPGMA) via a clustering approach, measuring nucleotide distance with the Jukes–Cantor method. Bootstrap analyses involving 1000 replicates determined the percentage of trees featuring respective nodes and groups, as shown in the figures. A distinct phylogenetic analysis was performed for the polyprotein sequences of the same DWV strains under similar settings. For the DWV-C sequence (ER5657949), a crucial adjustment had to be made—the insertion of a C at position 5327—to restore the open reading frame, which had been frame-shifted at this point. The resulting alignment was also manually checked for accuracy without the need for adjustments (Figure S3).

### 2.7. Genetic Marker and Reporter Gene Insertion in prDWV-B

To discern between the DWV-B clone and potential wild-type virus contamination possibly already present in experimental animals, a genetic marker was introduced. We disrupted an XhoI restriction enzyme site within the rDWV-B genome by mutating nucleotides T_9645_ to G_9645_. Mutagenesis was executed through the amplification of two PCR fragments from the prDWV-B plasmid, followed by subsequent DNA assembly. These fragments were generated via Q5 PCR (Qiagen) employing the oligonucleotides T_9645_G_rev and Amp_fwd, as well as T_9645_G_fwd and Amp_rev. The resulting plasmid was named prDWV-B-T_9645_G. The restriction-site deletion marker was analyzed following RT-PCR. After the transfection of bee pupae, incubation, and RNA preparation, the respective genome fragment was amplified using the oligonucleotides Marker-PCR_fwd and Marker-PCR_rev. An aliquot of the PCR products was digested in the reaction mixture by adding 1 µL of XhoI and incubating for 1 h at 37 °C. The RT-PCR products and their resistance against XhoI fragmentation were assessed through agarose gel electrophoresis, DNA staining (peqGREEN; VWR, Radnor, PE, USA), transillumination, and digital imaging.

To distinguish our viral clones from wild-type DWV without the need for molecular detection methods, we integrated a GFP gene as an additional reporter, as detailed earlier in our patent (WO 2019063789A1). In brief, we inserted the acGFP gene along with a T2A peptide gene sequence directly after the ATG start codon of the ORF. This reporter gene cassette was amplified using the oligonucleotides 5′-UTR-acGFP_fwd and ORF-T2A_rev. The plasmid prDWV-B was amplified using the oligonucleotides ORF-DWV-B_fwd and DWV-B-ATG_rev. Subsequently, the two PCR fragments were seamlessly assembled by recombination, yielding the plasmid prDWV-B-GFP. The GFP reporter protein signal in infected bees was assessed using a binocular microscope equipped for green fluorescence (SFA-GR; NIGHTSEA, Lexington, MA, USA) and digital imaging using the “Royal Blue wavelength set” (Excitation LED: 440–460nm; Emission filter: 500 nm longpass). Similarly, the reporter gene signal in infected bee cells was visualized using a fluorescence microscope (IX70; Olympus, Tokyo, Japan) and digital imaging.

### 2.8. Western Blotting

For sodium dodecyl sulfate–polyacrylamide gel electrophoresis (SDS-PAGE), the homogenized samples were mixed with SDS-PAGE loading buffer, incubated at 95 °C for 5 min, and loaded onto 7.5% polyacrylamide tricine gels. Identical quantities of the equally prepared samples were loaded into each of the gel pockets. Proteins were separated by electrophoresis (100 V for 1.5h) and transferred onto nitrocellulose membranes (Pall, Pensacola, FL, USA) through wet blotting (77 V for 1 h). After blotting, the membranes underwent washing and staining with Ponceau S solution to verify the transfer’s success and to ensure proper loading. Subsequently, membranes were blocked overnight at 4 °C using PBS containing 5% skim milk powder (Carl Roth; Karlsruhe, Germany) and 0.1% Tween-20 (Invitrogen; Karlsruhe, Germany). Following this, incubation with primary murine monoclonal antibodies (MAbs) and secondary goat anti-mouse HRPO conjugate (115-035-146; Dianova, Hamburg, Germany) occurred for 1 h at room temperature. Antibody binding was detected utilizing chemiluminescence (ECL-Prime; GE Healthcare; Chicago, IL, USA) and digital imaging (Odyssey M; Li-Cor; Lincoln, NE, USA). Mab VP1A1 against the VP1 of DWV [36], Mab 55A10 anti-Pol [19], and Mab B-2 anti-GFP (sc-9996; Santa Cruz; Dallas, TX, USA) were employed as specified.

## 3. Results

### 3.1. DWV-B Stocks for Molecular Cloning

**Figure 2:**
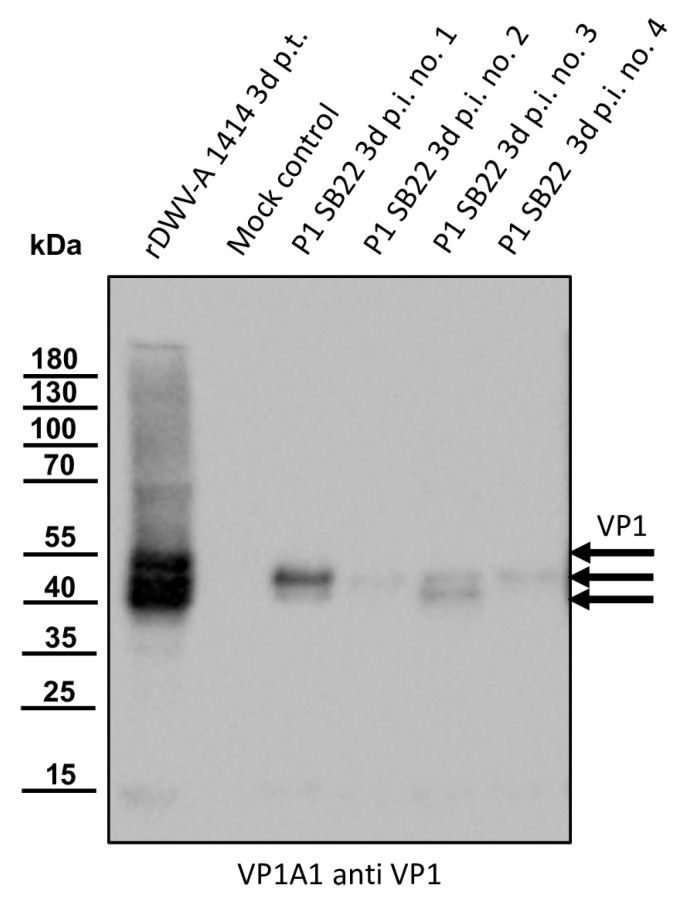
Western blot analyses of bee pupae infected with the isolate Austria-SB22. As controls, honey bee pupae were transfected with the in vitro-transcribed RNA of DWV strain 1414 (rDWV-A 1414 3d p.t.) or mock-transfected with PBS. Four pupae were injected with 1 µL of a DWV-B-positive bee lysate (P1 SB22 3d p.i. no. 1–4). After a three-day incubation period, the pupae were harvested and homogenized, and the total bee protein was resolved via SDS-PAGE. A typical VP1 pattern with bands at 47, 42, and 39 kDa appeared in the rDWV-A-transfected pupa. The mock-transfected pupa showed no signal, indicating no background infections in our experimental animal. All pupae inoculated with the original sample material of SB22 showed a protein band at 42 kDa. Pupa no. 1 displayed a strong signal, while signals in pupa nos. 2–4 were relatively weak. Pupa nos. 1 and 3 showed an additional signal at 39 kDa. Arrows indicate the protein bands of the characterized target proteins of the antibody used. The bands of a pre-stained molecular weight marker are indicated on the left.

The original sample material (P0) was injected into naïve bee pupae, which were then incubated for three days prior to harvest. As a positive control, the RNA of the established DWV-A clone rDWV-A 1414 was injected. Naïve pupae from the same comb were injected with PBS and served as a mock control. The VP1-specific Mab VP1A1 was employed to confirm successful infection and identify pupae with a high viral load of DWV-B in Western blot analyses (Figure 2).

Pupae injected with the RNA of rDWV-A 1414 exhibited strong signals at protein bands at 47, 42, and 39 kDa, representing the different VP1 species present in DWV-A-infected pupae. Weak additional signals at 68, 35, and 15 kDa were observed, possibly indicating incompletely processed polyprotein fragments or products of protein degradation. The mock-infected control bee displayed no protein reactivity with the antibody, indicating the absence of pre-existing DWV infections in this bee pupa. All four pupae injected with the sample material (P1) showed a protein band at about 42 kDa, indicating successful infection. Pupa no. 1 displayed a strong signal at 42 kDa, while signals in pupa nos. 2–4 were relatively weak. Notably, pupa nos. 1 and 3 showed an additional signal at 39 kDa, which was more intense in pupa no. 3 compared to no. 1. A very faint band at approximately 15 kDa was detected in pupa nos. 1 and 3. Due to the inability to quantitatively assess the cross-reactivity of the VP1 antibody, we concluded solely that pupa no. 1 has the strongest DWV-B protein expression for VP1, probably correlating with the highest DWV-B RNA yield. Hence, viral RNA extraction and RT-PCR typing using DWV-B-specific oligonucleotides were performed on material from pupa no. 1. Upon confirming the DWV strain as a DWV-B variant, reverse transcription was conducted using the oligonucleotide Adapter-dt, followed by PCR amplification of fragments covering the entire genome. DNA fragments of the desired nucleotide length were excised from agarose gels, purified using silica columns, and sent for Sanger sequencing. The genome sequence (PP418870) closely matched the VDV-1 sequence (AY251269), leading us to select strain DWV-B Austria-SB22 for the generation of a molecular virus clone.

### 3.2. RACE-PCR and Molecular Cloning of DWV-B Strain Austria-SB22

Following A-tailing RACE-PCR, the products were gel-purified and directly subjected to Sanger sequencing to obtain a 5′-end consensus sequence. After cloning into a T-vector backbone, three independent clones were sequenced to obtain clonal sequences. The entire experiment was then repeated, using C-tailing RACE-PCR to reliably determine the first nucleotide of DWV-B. Not only the consensus sequence of both reactions but also the three T-vector clones each showed an identical 5′-end sequence (Figure S1). This sequence is 34 nucleotides longer than the publicly available VDV-1 strain sequence (Genbank, AY251269) and exactly matches the 5′-end previously determined for DWV-A strain 1414. Since this 5′-end has already been successfully used by others to generate synthetic constructs for DWV-B, we were confident that it is the authentic end of strain SB22 [37]. With this knowledge, we were able to set up a full-length genomic PCR. Genomic double-stranded DNA was amplified by RT-PCR, gel-purified, and inserted into a pBR322 backbone, as described earlier [36]). The plasmid DNA of several independent clones was prepared and sequenced via full-plasmid pore-sequencing. We selected a clone named prDWV-B for subsequent studies, because it completely matched the RT-PCR-generated consensus sequence of strain Austria-SB22 (GenBank PP418870).

### 3.3. Phylogenetic Analyses

**Figure 3:**
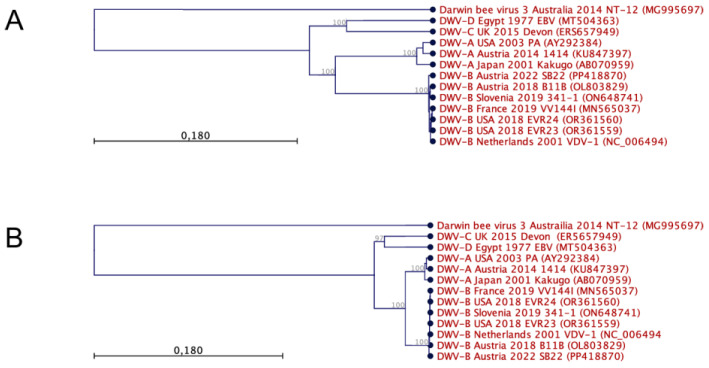
Phylogenetic analyses of DWV genomes and gene products were conducted using nucleotide (**A**) and polyprotein sequence (**B**) alignments employing UPGMA tree construction methods. Each virus is identified by its respective master variant, country of origin, year of isolation, strain designation, and accession number. The viruses were predominantly isolated from Apis mellifera, except for VDV-1, originating from Varroa destructor mites, and VV144I, obtained from Vespa velutina. The relationships between the species are illustrated in an unrooted increasing phylogram, with node numbers indicating bootstrap values derived from 1000 replicates in percentages. Scale bars represent the number of substitutions per site. Notably, the analysis reveals the distinct separation of individual master variants, close relationships among master variants, and a shared ancestry of DWVs.

Representative genome sequences of the DWV master variants DWV-A, DWV-B, DWV-C, and DWV-D were aligned to each other and to Darwin bee virus 3. This multiple sequence alignment (Figure S2) was then used to perform phylogenetic analyses of the evolutionary relations using a clustering approach (Figure 3A). The resulting phylogenetic tree separates Darwin bee virus 3 and proposes a common origin of all DWV strains. Furthermore, the DWV-A and -B strains constitute a common root, while the DWV-C and -D strains form a twin root. The individual master variants are clearly separated from each other before the tips of the branches separate the individual isolates.

Within the DWV-B group, it is noticeable that the relationships are very close or that the sequence divergences are very low (Table S1). The direct comparison of the DWV-B strain Austria-SB22 with strain VDV-1, for example, showed 10,029 of 10,110 matching nucleotides, excluding the very 5′-end. There were two small gaps in the VDV-1 sequence, which could represent sequencing errors in the first DWV-B sequence, as it was created using an older technology and as the sequence was not functionally tested. The Austria-SB22 strain exhibits the highest genetic similarity with two more recent DWV-B strain sequences deposited in GenBank: B11B (Austria 2018, OL803829) and 341-1 (Slovenia 2019, ON648741). Both strains have been sequenced in geographically close regions in recent years, so a direct relationship between the strains can be assumed.

In contrast, the nucleotide sequence divergence between DWV-B and DWV-A is considerable. When comparing the DWV-B strain Austria-SB22 with the first tested functional sequence of DWV-A (strain 1414), we found only 8571 of 10,167 nucleotides matching. Among these 1.574 mismatches, the majority stemmed from discrepancies in the third nucleotide of the codons (detailed below). Gaps of 19 nt in total occurred in the DWV-B sequence. Noteworthy is an 11 nt gap preceding the ORF start codon (T_1156_TGATTATTAA_1166_ in DWV-A strain 1414). This gap was consistently observed in all examined DWV-B genomes assigned conclusively to the master variant B (see also Figure S3 in [27]). In direct comparison with DWV-A, the DWV-C and -D genomes also exhibited a deletion in this region. The four nucleotides immediately preceding the DWV-B gap (A_1152_TAA_1155_ in DWV-A strain 1414) were missing in the DWV-C and -D genomes, indicating the genetic adaptability of this UTR region. Among each other, the DWV-A genomes displayed close relationships, with a comparison between the Kakugo virus and strain 1414 revealing 9828 of 10,147 nucleotides matching. Gaps of 20 nucleotides in total occurred, potentially, in some cases, attributable to sequencing errors. This close relationship is further evident in the comparison between strain PA and strain 1414.

Mutations of the third codon nucleotide often have no consequences for the gene product, since the genetic code exhibits redundancy, with multiple codons encoding the same amino acid. Such synonymous mutations are only subject to low functional selection pressure, as they might only affect the RNA structure or mildly de-optimize the codon-use-dependent translation efficiency. We investigated the amino acid sequence divergence of the polyproteins to identify functionally significant sequence differences (Figure S3). The evolutionary relationships between the viruses were again determined using a clustering algorithm, yielding a very similar tree (Figure 3B). Darwin bee virus 3 consistently emerged as a distinct outgroup, while the polyproteins of all DWV master variants shared a robust common root. This root bifurcated into two main branches, housing DWV-A and -B and DWV-C and -D groups, respectively. At the tips of these branches, there were clear demarcations that separated virus groups according to their respective master variants.

In a direct comparison, the DWV-B strains Austria-SB22 and VDV-1 showed an astonishing amino acid identity, with only 9 amino acids differing out of 2893 (Table S2). Among these exchanges, six were conservative substitutions, which typically have minimal effects on protein function. Only three observed radical substitutions are more likely to have functional effects on protein functions. Considering that only nine amino acid changes occurred following 81 nucleotide substitutions and considering the high degree of functional conservation observed in these substitutions, an extremely robust purifying selection pressure acts on the evolution of DWV-B. A direct comparison of DWV-B strain Austria-SB22 and DWV-A strain 1414 polyproteins reveals a lower identity, with 2754 out of 2893 amino acids matching. The high frequency of conservative substitutions is again remarkable, accounting for 88 out of 139 substitutions. Only a few clusters of radical replacements stand out, particularly located within the leader protein with T_54_VYDHT_59_, H_97_V_98_, and G_121_EFV_124_ alongside I_1795_EPSTSRPL_1803_ within the nonstructural protein region. Interestingly, very few substitutions occurred in the sequences of the capsid proteins, which usually represent a hotspot of sequence diversity in Picorna-like viruses.

### 3.4. An Infectious cDNA Clone of DWV-B Austria-SB22

**Figure 4:**
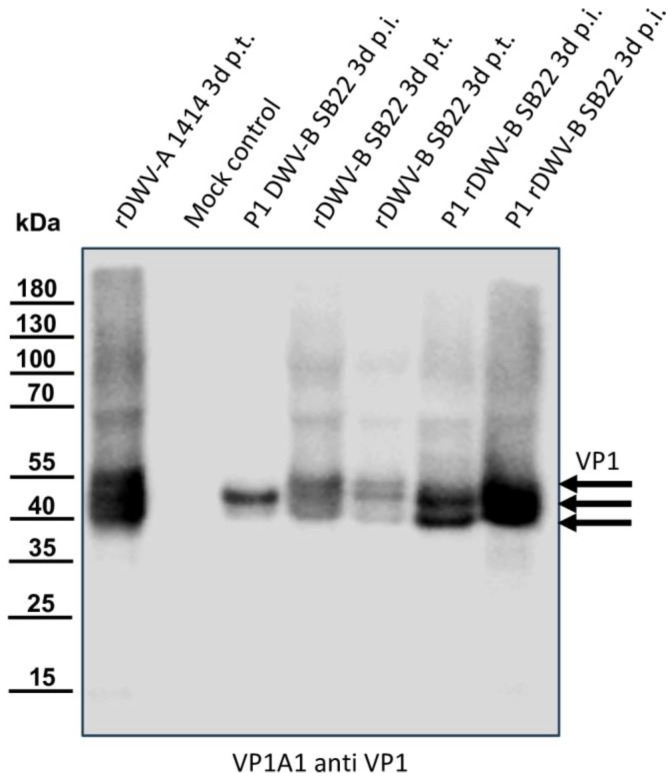
Western blot analyses of bee pupae transfected or infected with the DWV-B clone. Honey bee pupae were either transfected with the in vitro-transcribed RNA of DWV strain 1414 (rDWV-A 1414 3d p.t.) or mock-transfected (Mock control). One pupa was injected with 1 µL of a DWV-B-positive bee sample (P1 DWV-B SB22 3d p.i.), and two pupae were transfected with the in vitro-transcribed RNA of rDWV-B Austria-SB22 (rDWV-B SB22 3d p.t.). Subsequently, two naïve pupae were injected with 1 µL of lysate from rDWV-B-transfected pupae (P1 rDWV-B SB22 3d p.i.). Total bee protein was resolved via SDS-PAGE, blotted, and analyzed using a VP1-specific antibody. In the rDWV-A-transfected pupa, a typical VP1 pattern with bands at 47, 42, and 39 kDa was observed, whereas the mock-transfected pupa showed no signals. The pupa inoculated with the original DWV-B SB22 material displayed a strong protein band at 42 kDa and a faint band at 39 kDa. The two bee pupae transfected with in vitro-transcribed RNA displayed protein bands at 47, 42, and 39 kDa, similar to rDWV-A but less intense. However, pupae infected with lysates from these transfected animals exhibited very strong bands at 43 and 39 kDa, indicating further spread and growth of rDWV-B in the first passage. Arrows indicate the protein bands of the characterized target proteins of the antibody used.

In vitro-transcribed RNA was injected into honey bee pupae, followed by incubation, harvest, and homogenization. To assess productive infection and infectious particle production, 1 µL of the homogenate from these transfected pupae was injected into fresh naïve pupae. Samples from both transfected and infected pupae were then analyzed for VP1 expression alongside positive and negative controls (Figure 4). In the positive control (rDWV-A-transfected), strong characteristic VP1 bands at 47, 42, and 39 kDa were observed, while no reactivity was detected in the mock-infected pupa. Passage 1 of the DWV-B field virus (Austria-SB22) showed an intense signal solely at 42 kDa, as seen in Figure 2. However, bee pupae transfected with the in vitro-transcribed RNA of rDWV-B exhibited signals at 47, 42, and 39 kDa, akin to rDWV-A signals but with lower intensities. Upon passage, the virus clone yielded strong signals at 42 and 39 kDa in infected pupae, indicating further replication. Additionally, weaker signals corresponding to different VP1 species and degradation products (47 kDa and 15 kDa) were observed alongside the two main bands in rDWV-B passages.

The replication dynamics of in vitro-transcribed RNA from the rDWV-B clone were analyzed via qRT-PCR (Table 2). Employing a well-established DWV-B-specific qRT-PCR assay [26], we had to modify the reverse primer for optimal hybridization with strain Austria-SB22. DWV-A was quantified as described earlier [36]. To measure and compare viral RNA replication products against remnants of in vitro-transcribed RNA, bee pupae received 1 µg of RNA from the rDWV-B strain Austria-SB22 and the rDWV-A strain 1414, as well as from two non-replicative control genomes, namely, rDWV-B-E_1102_Stop and rDWV-A-Q_2118_A [19]. Further controls encompassed the field virus (wtDWV-B, SB22) and mock-transfected bee pupae. We also passaged the virus clone from the lysates of transfected bee pupae (P1 rDWV-B and P1 rDWV-A) to discern further virus growth. After three days of incubation, the pupae were harvested, total RNA was prepared, and qRT-PCR measurements were performed. DWV-B genomes were undetectable in mock-transfected pupae and in rDWV-A-transfected animals using the DWV-B-specific assay, demonstrating the test’s specificity and the absence of background infection in this experimental cohort. Approximately 1 × 10^11^ genome equivalents per bee (GEs/bee) were measured three days post-infection with the field virus from the original sample (wtDWV-B, SB22). Autonomous replication of rDWV-B RNA was evident when compared directly to the non-replicative control RNA (rDWV-B-E_1102_Stop). Notably, the in vitro-transcribed RNA of the non-replicative genome (rDWV-B-E_1102_Stop) underwent significant degradation during the incubation period, leaving only 7 × 10^4^ GEs/bee from the initially transfected 1.73 × 10^11^ RNA molecules. At the same time, the functional virus genome reached levels of about 2 × 10^10^ GEs/bee, indicating active replication. While the non-replicative genomes of rDWV-B-E_1102_Stop were either undetectable in the passage experiment (two of three animals) or only detected at the lower limit of detection (1.75 × 10^3^ GEs/bee in one animal), the rDWV-B values increased during the passage and reached values comparable to wtDWV-B with around 1 × 10^11^ GEs/bee (P1 rDWV-B). In direct comparison with the established clone of rDWV-A, which yielded 6.6 × 10^9^ GEs/bee after transfection (RNA rDWV-A) and 9.3 × 10^9^ GEs/bee in passage one (P1 rDWV-A), a difference of more than one log was observed in replication levels between DWV-A and DWV-B in the head-to-head experiment. Degradation of the RNA of our non-replicative rDWV-A-Q_2118_A mutant was also evident after transfection, and the RNA of rDWV-A-Q_2118_A could no longer be detected in a passage experiment. The result of a two-sided T-test shows a statistical significance for the differences between the replicative DWV-A and DWV-B genomes and the respective mutated non-replicative genomes (*p* < 0.05). However, the observed differences between the titers of the DWV-A and DWV-B clones in the passage experiment are not statistically significant (*p* > 0.05). The average GE/bee for the individual bee pupae is shown in the Supplemental Information of the manuscript (Table S3).

### 3.5. A Genetic Marker for rDWV-B

We eliminated an XhoI site from the cDNA of the rDWV-B clone to distinguish between wild-type field viruses and our virus clones. Bee pupae were injected with wtDWV-B Austria-SB22, PBS, or the in vitro-transcribed RNA of rDWV-B-T_9645_G. After three days of incubation, the pupae were homogenized and subjected to Western blot analysis for VP1 expression (Figure S4). The wild-type-virus-infected pupae exhibited a characteristic band at 42 kDa, with weaker signals at 47 and 39 kDa, while no reactive proteins were detected in the mock-infected pupa. Strong signals were observed in the rDWV-B-T_9645_G-transfected pupae at 47, 42, and 39 kDa, along with additional weak signals at 100, 68, and 15 kDa, representing unprocessed precursors or degradation products. The VP1 protein expression clearly indicated productive infection and protein expression following RNA transfection. RNA from these homogenates was extracted to assess the utility of the genetic marker. An RT-PCR assay targeting the respective genome region generating a product of 1531 bp yielded no band in the mock sample, while the expected products were obtained in wtDWV-B-infected and rDWV-B-T_9645_G-transfected bee pupae. Additional weaker bands at approximately 3000 and 1400 were observed, which did not appear in PCR tests using plasmid DNA. Hence, these additional bands likely resulted from less specific hybridization in the reverse transcription reaction. Digestion of the RT-PCR products with XhoI led to the complete fragmentation of the amplicon derived from the field virus genome into two smaller fragments of 1026 kb and 505 bp. The RT-PCR amplicon of the rDWV-B-T_9645_G-transfected pupae exhibited resistance against the activity of XhoI, still presenting one band at 1531 bp after incubation with XhoI. In conclusion, our RT-PCR and XhoI digestion system for detecting the DWV-B clone proved effective. Furthermore, the mutation of RNA at position T_9645_ was well tolerated by the virus, with no obvious pressure to revert this mutation, at least following RNA transfection.

### 3.6. Generation of a Reporter DWV-B

**Figure 5:**
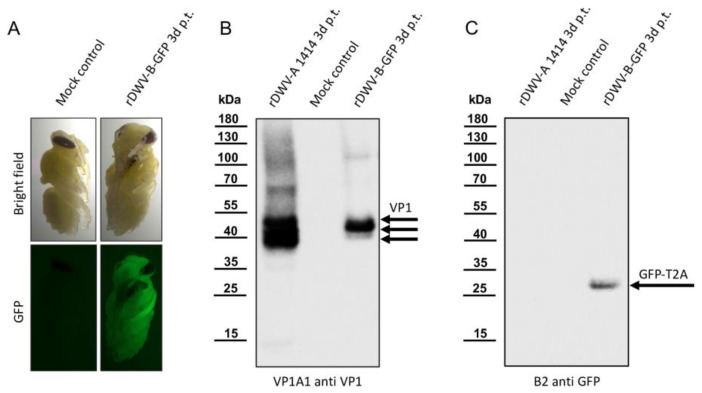
The employment of a GFP reporter in rDWV-B. (**A**) The fluorescence of rDWV-B-GFP-transfected bee pupae. The GFP fluorescence emanating from the rDWV-B-GFP-transfected pupa was remarkably intense and already easily visible through filter eyeglasses. Conversely, only negligible background fluorescence was detected in the mock-transfected pupa. (**B**) VP1 expression of rDWV-B-GFP. Pupae were transfected with either rDWV-A or rDWV-B-GFP or injected with PBS (Mock control) and harvested three days post-transfection. Whole-bee lysates were prepared and subjected to SDS-PAGE, followed by blotting and VP1 analysis. Typical VP1 bands were evident in the positive control (rDWV-A 1414), and a strong VP1 signal was observed in the rDWV-B-GFP-transfected pupa, with main bands appearing at 42 kDa and 39 kDa. No signals were detected in the mock-infected pupa. (**C**) Processing of the GFP-T2A reporter. Another Western blot was conducted using B2 anti-GFP. Only the rDWV-B-GFP-transfected bee exhibited a signal corresponding to the calculated weight of GFP-T2A at an apparent molecular weight of 29 kDa. The absence of additional bands suggests the effective separation of GFP and the leader protein by the T2A peptide.

We introduced a GFP-encoding gene upstream of the ORF of our rDWV-B clone. To ensure the production of a functional leader protein, we additionally inserted a T2A peptide sequence behind the GFP. Such 2A peptides are a class of 18–22 aa long peptides that induce ribosomal skipping during translation, ensuring proper processing of downstream proteins. The construction of the plasmid prDWV-B-GFP involved PCR and homologous recombination techniques. Bee pupae were then transfected with the in vitro-transcribed RNA of rDWV-B-GFP or mock-treated with PBS as a negative control. After 72 h of incubation, the pupae were examined using a fluorescence binocular microscope (Figure 5A).

While no fluorescence signal was observed in the mock-treated control bees, the rDWV-B-GFP-transfected bees exhibited visible green fluorescence. Notably, this signal was detectable throughout the body of the transfected bee, indicating systemic infection. To investigate whether the GFP protein was released as a free molecule, ensuring the maturation of functional leader proteins, we conducted Western blot analyses. By utilizing Mab VP1A1 for detecting DWV infection (Figure 5B) and B2 anti-GFP (Figure 5C) for detecting the reporter protein, we demonstrated infection and the complete separation of GFP from the leader protein. An rDWV-A-infected pupa served as a positive control, exhibiting typical intense VP1 signals at 47, 42, and 39 kDa, while no signals were detected in the mock-infected control animal. Like rDWV-B without a reporter gene, rDWV-B-GFP displayed an intense VP1 band at 42 kDa and a weaker band at 39 kDa, indicating productive infection. A signal for GFP-T2A (with a calculated molecular mass of 28.7 kDa) was obtained solely from the rDWV-B-GFP-transfected pupa, consistent with the expected molecular mass. Thus, it can be inferred that the T2A peptide is functionally active, inhibiting the formation of a regular peptide bond on the ribosome and consequently leading to the complete separation of GFP-T2A from the leader protein during translation. To document the spread and infection of rDWV-B-GFP at the cellular level, we isolated cells of infected honey bee pupae (Figure S5). While no fluorescence was detected in the cells of mock-transfected control animals, round suspension cells and adherent epithelial cells of the rDWV-B-GFP-transfected pupae showed an intense GFP signal. Passage of the infected cells was not possible. Future cell culture studies still have to show to what extent DWV-A and -B cause cytopathic effects.

### 3.7. rDWV-B Shows Reduced Virulence Compared to rDWV-A

Our original aim was to evaluate the virulence of the wild-type isolates (wtDWV-A and wtDWV-B) and to compare them with the clonal viruses (rDWV-A and rDWV-B). However, SBV was detected in our primary stock of DWV-B strain Austria-SB22 using RT-PCR. Serial dilution showed that the titer of the SBV contaminant was higher than that of DWV-B, so we were unable to grow a clean wtDWV-B stock. Since a comparison with a double infection is pointless, we had to abandon these analyses. However, major differences in the clinical presentation of emerging bees were already observed after the transfection of in vitro-transcribed RNAs. Hence, we decided to compare the virulence of rDWV-B and -A under controlled conditions using the transfection of in vitro-transcribed RNA, which eliminated the possibility of inoculum contamination.

**Figure 6:**
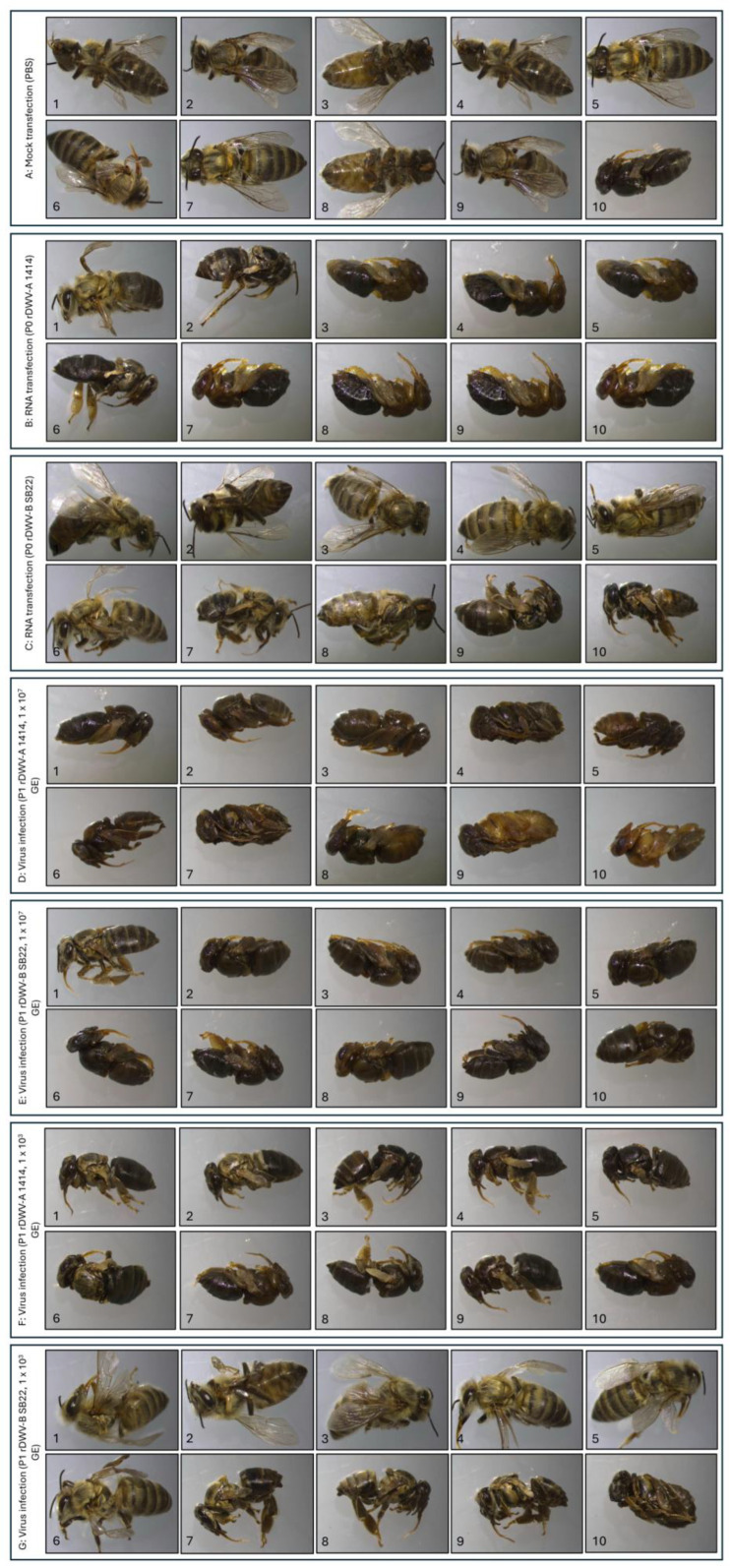
Ten bee pupae each were subjected to different treatments: mock transfection (**A**) or transfection with the RNA of rDWV-A (**B**) or rDWV-B (**C**). Additional groups were injected with either 1 × 10^7^ GEs of passaged rDWV-A (**D**) or rDWV-B (**E**) or 1 × 10^3^ GEs of passaged rDWV-A (**F**) or rDWV-B (**G**). All specimens were injected on day 13 of development and incubated until emergence. In the mock-transfected group (**A**), 90% of the pupae survived, with only a 10% mortality rate. Transfection with rDWV-A RNA (B) resulted in an 80% mortality rate during the pupal stage. The surviving 20% emerged as crippled adults but died shortly after. Conversely, 80% of the pupae transfected with rDWV-B RNA (**C**) transitioned to adults (mortality rate of 20%). Of these, five appeared healthy, one had slight wing deformities, and two had severe deformities. A high-titer infection with 1 × 10^7^ GEs of rDWV-A (**D**) resulted in 100% mortality during the pupal stage. Similarly, 1 × 10^7^ GEs of rDWV-B (**E**) led to a 90% mortality rate, with only one specimen surviving. In contrast, low-dose infection with 1 × 10^3^ GEs mirrored the RNA transfection results, with a 100% mortality rate in the case of rDWV-A (**F**) and a 20% mortality rate after rDWV-B infection (**G**). For rDWV-B, 50% of the pupae emerged without clinical signs of infection, 30% emerged with mild to severe malformations, and 20% died before emergence.

The bee pupae were transfected on development day 13 and incubated in a humidified environment at 33 °C until completing metamorphosis on day 21. Mock-transfected pupae served as a negative control, with 90% emerging as healthy adult bees (Figure 6A). However, 1 out of 10 pupae (Figure 6A, no. 10) died before metamorphosis. Consistent with previous results, pupae transfected with the rDWV-A strain 1414 had a high infection-related mortality rate of 80% during pupation (Figure 6B). The two surviving animals showed considerable deformities after emergence, including crippled legs and wings (Figure 6B, no. 1), as well as shortened abdomens and discoloration (Figure 6B, no. 2). In stark contrast, five bees emerged as healthy-appearing adults following transfection with rDWV-B (Figure 6C, nos. 1–5). Among the others, three bees displayed mild (Figure 6C, no. 6) to severe malformations (Figure 6C, nos. 7 and 8). Only two mortalities were recorded before emergence, resulting in a rate of 20% (Figure 6C, nos. 9 and 10). This experiment highlighted the markedly higher virulence of the rDWV-A strain 1414 compared to the rDWV-B strain Austria-SB22 after transfection of in vitro-transcribed RNA. Subsequent repetitions of the experiment using pupae from various bee colonies consistently yielded similar outcomes in terms of morbidity and mortality rates.

Driven by these clear results, we wanted to compare the effects of infections with the recombinant viruses. Therefore, a further series of experiments was carried out using lysates of the rDWV-B- and rDWV-A-transfected pupae. Naïve pupae were injected with 1 µL of sterile-filtered and diluted lysates, each containing approximately 1 × 10^7^ GEs/µL. With this high infection titer, both viruses caused similar high mortality rates (Figure 6D,E). All test animals died before metamorphosis, except for one pupa infected with rDWV-B (Figure 6E, no. 1), which emerged severely crippled. It should also be noted that pupa nos. 9 and 10 in the DWV-A infection experiment died before their body surface could turn dark during pupal development. It must therefore be assumed that both DWV-A and DWV-B are pathogenic viruses with considerable virulence. To explore the dose dependency of DWV virulence, the infection experiment was repeated with a low infectious dose of approximately 1 × 10^3^ GEs (1:10,000 dilution of the same P1 virus stock). All animals infected with a low dose of rDWV-A died before metamorphosis, whereas 50% of those infected with rDWV-B developed into clinically unaffected adult bees. Among the rDWV-B-infected bees, three showed crippling, and two died before metamorphosis. To confirm infection with rDWV-B at the low dose, VP1 expression was tested via Western blotting, yielding positive results for all rDWV-A- and rDWV-B-infected animals.

## 4. Discussion

In the northern hemisphere, honey bee colonies face a double threat to their productivity and reproduction: Varroa mites and the RNA viruses they transmit [4,41]. These parasites compromise the general health of honey bee colonies and, above all, their ability to survive the winter. Among the viruses transmitted by Varroa mites, DWV is the most devastating. The connection between Varroa mite infestation and DWV infection is so close that the appearance of clinical DWV symptoms alone indicates a considerable mite load in the bee colony. It is striking that in regions where no Varroa mites are present, the incidence of DWV is minimal. Historically, DWV could not be detected in the Australian bee population by the Australian Centre for Disease Preparedness [42]. As the mites have now been established in Australia, DWV will certainly soon be widespread [43]. In North America and Europe, the prevalence rates of DWV mirror those of Varroa mites, peaking in the fall when the number of mites in bee broods increases exponentially toward the end of the season, inducing colony losses [44,45,46,47]. Effective treatments against mites in autumn, followed by a brood-free winter period, result in significantly reduced DWV prevalence in spring [48]. Early studies by Ongus et al. and current investigations point to the active replication of both DWV variants, DWV-B and DWV-A, within the mites using strand-specific RT-PCR assays [23,49]. Recent advances, including in situ hybridization, have corroborated DWV-B’s replication in mite cells, though DWV-A’s replication was not detectable via the same methods [50]. Evidence for the active replication of DWV-A in the mites was also lacking in studies assessing the accumulation of viral RNA after the ingestion of viruses [51]. Since different experimental approaches have yielded different results, the question of active replication in mite tissue cannot be answered conclusively for DWV-A, yet. While the debate over DWV-A’s active replication in mite tissue persists, there is no disputing that both DWV variants are efficiently transmitted by the mites regardless of whether they are actively multiplied or not.

Recent studies indicate a significant shift in the predominant variant from DWV-A to DWV-B in both the USA and Europe over the past few decades [24,52,53]. However, there exist conflicting data regarding the implications of this master-variant shift, particularly regarding a potential difference in virulence. Understanding the epidemiology of DWV, including its virulence and transmission dynamics, is crucial for addressing the current challenges facing beekeeping and for developing potential intervention strategies. Given the extensive scope of research on DWV, it is understandable that this discussion may not cover every aspect, and reference must be made to earlier reports and reviews [24]. The knowledge about the virulence of the master variants -A and -B remains ambiguous, lacking clear conclusions. Assessing DWV’s impact in the field involves correlating colony mortality with master-variant prevalence. Studies in the USA and UK suggested that colony mortality correlated with DWV-A prevalence [54,55], while DWV-B prevalence showed no such association. However, other studies from Europe found the opposite pattern, suggesting a higher virulence of DWV-B [28,30,56], while a study from Turkey found an association of both variants with colony losses [57]. Initially, DWV-B was identified as a virus replicating in Varroa mites [23]. After DWV-B was detected a decade later in British bee colonies that had survived an uncontrolled Varroa infestation, a protective effect of DWV-B was suspected [55]. It was hypothesized that DWV-B’s lower virulence and higher RNA titers might induce superinfection exclusion for DWV-A, enhancing colony tolerance to mite infestation. At about the same time, isolates of the two master variants were compared in laboratory experiments, where a higher virulence of DWV-B was observed after the infection of adult bees. In line with these observations, slightly higher RNA titers of DWV-B and a tendency of the two master variants to recombine were observed in DWV-A/-B coinfection experiments [55]. Other studies used pupae infection models and found a lower virulence of DWV-B despite higher RNA titers compared to DWV-A [31]. Recent studies have even failed to detect differences in titer or virulence between DWV-A and -B in a very similar honey bee pupae model [58].

Assessing the virulence of honey bee viruses remains a challenge, given the complexity of field studies and the relative infancy of laboratory systems for honey bee viruses. The complexity of field observations arises from the fact that honey bee colonies must be considered dynamic populations that are exposed to the simultaneous influence of numerous pathogens, noxious and beneficial factors, and seasonal changes. Even focusing on dominant DWV variants through statistical analyses in extensive population studies proves difficult due to the prevalence of different strains and potential recombinants whose genotypes often remained uncharacterized. However, transferring the study to a more controllable laboratory environment poses several novel challenges. DWV exhibits variances in susceptibility and disease manifestation across different life stages of the bees (egg, larva, pupa, and adult), necessitating comprehensive investigations across all stages for general conclusions. However, given that mite reproduction occurs during the pupal stage and significant viral damage manifests post-pupation, evaluating DWV variant virulence in this stage proves particularly insightful. Thorough comparison requires the use of defined strains with known sequences and an inoculum that is as pure as possible. Yet, employing field bees for the propagation of “virus isolates” poses substantial risks, as the lack of virus-free “laboratory bees” requires constant vigilance against contamination by other viruses. In view of the large number of known bee viruses and the likelihood that undiscovered pathogens are still out there, even extensive stock testing cannot eliminate the contamination risk completely. Furthermore, each diagnostic assay used has a lower limit of detection. It is important to be aware that any pathogen present in the inoculum is significant in view of the pathogen’s ability to multiply and to interact with other pathogens. In summary, navigating the complexities of honey bee virus virulence assessments demands attention to detail and robust methodologies. To tackle some of these challenges, we have developed a reverse genetic system for a typical DWV-A (strain 1414, KU847397), facilitating controlled mono-infections with a known genotype within laboratory settings [36]. Here, we introduce a reverse genetic system for a representative DWV-B strain (Austria SB22, PP418870), enabling the direct comparison of both master variants within the same laboratory settings.

Examining the cloned genome of DWV-B, we found no differences in the 5′-end UTR sequence compared to that of DWV-A (strain 1414). The DWV-B clone displayed only 81 nucleotide substitutions resulting in nine amino acid changes compared to VDV-1. The genetic stability of DWV-B is notably high, with a substitution rate of 3.86 nucleotide changes per genome per year over the 21-year period separating the two strains. Typically, RNA viruses have mutation rates between 10^−6^ and 10^−4^ substitutions per nucleotide per cell infection, presenting an average mutation rate of 1.5 mutations per genome per replication round [59,60]. However, this dynamic is not observed in DWV-B genomes. We hypothesize that the entire DWV-B genome is under exceptionally strong purifying selection, which minimizes changes to both the nucleic acid and amino acid sequences. Others have found that DWV-A and DWV-B diverged approximately 308 years ago, predating the documented Varroa host switches [61]. Given the low substitution rate observed here, this estimate appears plausible. The comparison of DWV genomes highlighted some characteristic genetic features of the master variants. A gap of 11 nucleotides preceding the ORF start codon (T_1156_TGATTATTAA_1166_ in DWV-A strain 1414) is a hallmark of DWV-B genomes. DWV-C and DWV-D also exhibit a deletion of four nucleotides immediately preceding this site (A_1152_TAA_1155_ in DWV-A strain 1414), suggesting genetic flexibility in this region. The significance of these variations in the DWV UTRs remains undetermined, but they could impact the IRES-mediated translation of the polyprotein. Only a few small clusters of consecutive radical replacements were found when comparing the amino acid sequences of DWV-A and -B, namely, T_54_VYDHT_59_, H_97_V_98_, G_121_EFV_124_, and I_1795_EPSTSRPL_1803_ in DWV-B. Notably, functionally relevant substitutions within the capsid protein sequences are very rare. Mammalian picornaviruses often exhibit considerable sequence diversity in their capsid proteins, leading to the emergence of distinct serotypes evading the host immune pressure [62]. However, DWV appears optimized and prevents changes in gene products, likely due to the lack of selection pressure from an adaptive immune response.

The new DWV-B virus clone underwent functional analyses in vivo using a bee pupa model. Following the transfection of in vitro-transcribed RNA via injection, viral protein expression was detectable in bee pupae using a cross-reactive VP1-specific antibody. In addition, the numbers of viral RNAs after transfection of in vitro-transcribed RNA were measured, and the production of infectious virions was assessed. A comparison with a non-replicative control genome containing an artificially inserted stop codon revealed active viral RNA replication. These differences were statistically significant in two-sided T-tests. The subsequent passage of bee lysates into naïve bee pupae further demonstrated infection and genome replication, with virus titers exceeding 10^11^ genome equivalents per infected pupa, akin to the original DWV-B field isolate. A comparative analysis with the established DWV-A clone revealed substantial differences in RNA replication, exceeding a factor of 10. To unequivocally identify the replicative genome as the in vitro-generated virus clone and distinguish it from field viruses, an XhoI site in the genome was disrupted via mutagenesis. Viral protein expression was observed following RNA transfection of the virus containing the XhoI-site deletion marker. Amplification of the relevant genome fragment via RT-PCR and subsequent XhoI digestion confirmed the replication of the virus clone. We further constructed a DWV-B-GFP reporter virus based on previous work, in which a reporter virus for DWV-A was developed. As seen in DWV-A, the insertion of a GFP gene right behind the start codon of the ORF allowed the direct visualization of viral replication and protein expression. Very similar fluorescence intensities were observed in the case of DWV-B-GFP, allowing infected pupae to be identified macroscopically using blue illumination and a suitable optical filter. In addition, VP1 and GFP expression were detected in Western blot analyses, proving that a reporter virus could also be established for DWV-B. Cultures of primary bee pupal cells allowed DWV-B-GFP-infected cells to be identified under the microscope in live-cell-imaging applications, paving the way for future studies on the host cell range and cytopathogenicity of this virus.

We conducted an experimental comparison of the virulence of the two DWV clones in controlled laboratory settings. Upon the transfection of in vitro-transcribed RNA, which mimics a low-dose infection, clear differences in disease symptoms and mortality became apparent. While rDWV-A quickly killed or severely crippled all injected pupae, rDWV-B only led to noticeable damage or death in half of the subjects. However, when virus particles from the lysates of transfected animals were injected into naïve pupae, which corresponds to a high-titer infection with about a 10^7^ GE dose, the differences between the two virus variants were practically negligible. Since these lysates came from deceased animals that may have contained cytokines and toxins from lytic cells and protein degradation, the result is more difficult to interpret. The purification of virions for such experiments offers only limited advantages, because CsCl-purified material exhibits lower infectivity, while the presence of CsCl contamination may confound test results due to its inherent toxicity. Hence, we conducted an additional low-titer infection with diluted lysates containing approximately 10^3^ GEs. The results mirrored those from the RNA transfection. Despite the low titer, all rDWV-A-infected animals died before metamorphosis, whereas 80% of the rDWV-B-infected animals successfully emerged as adults. Half of the animals exhibited symptoms of disease, from mild to severe, including crippling and death. To rule out the possibility that the high dilution caused a lack of infection, we tested all animals for DWV protein expression and confirmed that all animals were infected with rDWV-A or rDWV-B. From these two experiments, it can be inferred that both DWV-A and DWV-B are pathogenic bee viruses exhibiting notable virulence. Moreover, it is evident that the DWV-B strain Austria-SB22 displays slightly reduced virulence compared to the DWV-A strain 1414.

The Varroa mite’s role as a vector is not addressed in this study. If DWV-A is not replicated within the Varroa and is only transmitted mechanically, infection doses should be significantly lower compared to DWV-B, since DWV-B amplifies in the mite [50]. The lower virulence of DWV-B might be attributed to its need not to inflict immediate damage on the host to avoid rapid host mortality, ensuring the successful transmission of the virus and mite. To deepen our comprehension of DWV’s characteristics and virulence, it is crucial to clone additional genomes from diverse strains or isolates and subject them to laboratory analyses, including tests with different stages of development and the mite vector. Previous studies on RNA viruses have demonstrated rapid virulence modulation, where even a single amino acid exchange altered the virus’s phenotype significantly [63]. Hence, caution must be exercised in generalizing the findings from the comparison of a sole DWV-A and a sole DWV-B strain, as presented here.

## Figures and Tables

**Table 1:** Oligonucleotides used in this study.

Designation	Sequence (5′-N × -3′)	Position in DWV-B (PP418870)
Adapter-dt	GGCCACGCGTCGACTAGTACTTTTTTTTTTTTTTTTT	nt10149-10173
Adapter	GGCCACGCGTCGACTAGTAC	-
Adapter-dg	GGCCACGCGTCGACTAGTACGGGGGGGGGGGGGGGGG	-
DWV-B-5′-RACE-outer_rev	CTTTCACACTTTCGCCTCATAC	nt796-817
DWV-B-5′-RACE-inner_rev	CCAATACAATTATCTCCAACTTGC	nt175-198
DWV-5′-end_fwd	TTTAAAATTCGCTATGGGAGGCGATTTATGC	nt1-31
Adapter-NotI_fwd	TAGTCGACGCGTGGCCGCGGCCGCTGCTACCTCACTAAC	-
DWV-5′-SP6_rev	CATAGCGAATTTTAAACTATAGTGTCACCTAAATCGCG	nt1-16
DWV-B-Seq1_fwd	GTGCGCGAGAAAGTTGTTAG	nt1585-1604
DWV-B-Seq2_fwd	CACGTATATCCATTCTTACC	nt2326-2345
DWV-B-Seq3_fwd	GTTATAGAATTGGAAGTC	nt4354-4371
DWV-B-Seq4_fwd	GATCTCATGGAAATGGGATCAAACCCATATATC	nt5503-5535
DWV-B-Seq5_fwd	GACGATTAGTATGTTACATCAGAG	nt6819-6842
DWV-B-Seq6_fwd	GCGGCATTACATTGAGTCGAC	nt7656-7676
DWV-B-Seq7_fwd	CTGGAATACTAGTGCTGGTTTTC	nt8628-8650
DWV-B-Seq8_rev	TTTTTACTATTATGGTTAAAACTATAC	nt10127-10153
DWV-B-Seq9_rev	CACTCTAACTCGTATTCACG	nt1717-1736
DWV-B-Seq10_rev	CTGCATACCATCGCCAATAC	nt4548-4559
DWV-B-Seq11_rev	TTTAGTCTCCTTCTGGCACC	nt6926-6945
DWV-B-Seq12_rev	GGCAATCTATGGATTCTAGG	nt7468-7487
DWV-B-Seq13_fwd	TAAGTATATTCATAATCAAGA	nt7710-7730
DWV-B-Seq14_fwd	CAGTTAGTGCATGCTATCATTG	nt4813-4834
DWV-B-Seq15_fwd	GTATGAGGCGAAAGTGTGAAAG	nt796-817
DWV-B-Stop1102_fwd	TAGATTTCGGTTGGTTTTCAAGCTAC	nt4456-4481
DWV-B-Stop1102_rev	ACCAACCGAAATCTATCCGAGCGAAACGGCATAAC	nt4436-4470
DWV-B-qRT_fwd	GCAAGTTGGAGATAATTGTA	nt175-194
DWV-B-qRT_rev	CGATACTTACGTTCTTCAAGAT	nt270-291
DWV-A-qRT_fwd	ATTGTGCCAGATTGGACTAC	-
DWV-A-qRT_rev	AGATGCAATGGANGATACAG	-
T_9645_G_rev	ACCCAATCCTCGCGCATGTGTCCAATTGGTTGTTCCTTC	nt9619-9657
Amp_fwd	GAATGAAGCCATACCAAACGAC	-
T_9645_G_fwd	GCGCGAGGATTGGGTCGTCGAGTAG	nt9643-9667
Amp_rev	GTCGTTTGGTATGGCTTCATTC	-
5′-UTR-acGFP_fwd	GTAAATATATATAAAAATGGTGAGCAAGGGCGCCGAGCTG	nt1137-1155
ORF-T2A_rev	CACAACTAAATGCCTAGGGCCTGGGTTTTCCTCAAC	nt1153-1168
ORF-DWV-B_fwd	ATGGCATTTAGTTGTGGAACTCTTTC	nt1153-1178
DWV-B-ATG_rev	CATTTTTATATATATTTACCTTC	nt1133-1155
Marker-PCR_fwd	CAATATCCTGGAATACTAGTGCTG	nt8621-8644
Marker-PCR_rev	TTTTTACTATTATGGTTAAAACTATAC	nt10127-10153

**Table 2:** Genome equivalents measured 3 days post-transfection/infection.

RNA or Virus	Average GE DWV-B/Bee (Average GE DWV-A/Bee)	Standard Deviation
Mock	n.d. * (n.d.)	-
wtDWV-B, SB22 (Virus)	1.4 × 10^11^ (n.d.)	3.8 × 10^10^ (-)
RNA rDWV-B-E_1102_Stop	4.5 × 10^4^ (n.d.)	6.7 × 10^4^ (-)
P1 rDWV-B-E_1102_Stop (Virus)	5.8 × 10^2^ (n.d.)	1.0 × 10^3^ (-)
RNA rDWV-B	1.8 × 10^10^ (n.d.)	1.0 × 10^10^ (-)
P1 rDWV-B (Virus)	1.7 × 10^11^ (n.d.)	2.1 × 10^11^ (-)
RNA rDWV-A-Q_2118_A	n.d. (1.2 × 10^4^ GE DWV-A)	- (1.1 × 10^4^)
P1 rDWV-A-Q_2118_A (Virus)	n.d. (n.d.)	- (-)
RNA rDWV-A	n.d. (6.6 × 10^9^ GE DWV-A)	- (3.0 × 10^9^)
P1 rDWV-A (Virus)	n.d. (9.3 × 10^9^ GE DWV-A)	- (1.4 × 10^10^)

Average titer of three individual bees, measured in three technical replicates. * n.d. means not detected.

## Data Availability

All data analyzed or generated during this study are included in the manuscript. Further information on the experimental protocols can be obtained from the corresponding author upon request. All materials used in this study that are not commercially available (e.g., antibodies) can be obtained from the corresponding author on request and for reasonable compensation.

## Supplementary Materials

The following supporting information can be downloaded at https://www.mdpi.com/article/10.3390/v16060980/s1, Figure S1. The 5′-terminus of DWV-B Austria-SB22; Figure S2. Genome alignment of DWVs; Figure S3. Polyprotein alignment of DWVs; Figure S4. Insertion of a molecular marker into the DWV-B genome; Figure S5. Primary culture of rDWV-B-GFP infected cells; Table S1. Divergence of DWV nucleotide sequences; Table S2. Divergence of DWV polyprotein sequences; Table S3. Genome equivalents of individual animals 3 days post transfection/infection.
