# Supplementary material for: Characterization of a Molecular Clone of Deformed Wing Virus B
Source: Viruses. 2024 Jun 18;16(6):980. doi: 10.3390/v16060980 (PMC11209315; doi:10.3390/v16060980)
Supplement: Supplementary file 1 [file viruses-16-00980-s001.zip › viruses-3018600-supplementary.pdf]

Figure S1. The 5′-terminus of DWV-B Austria-SB22 (A) The 5′-end sequence of the DWV-B strain was obtained through tailing RACE-PCR assays. The consensus sequence derived from Sanger sequencing of the PCR products is presented in the initial line. Both tailing reactions yielded identical consensus sequences and this sequence was also found in three cloned T-vectors each. Numbers denote the nucleotide position behind the VPg. (B) Sequence comparison of the 5′-terminus of DWVs. The 5′-terminus sequences of DWV-B strains VDV-1, Austria-SB22, and DWV-A strain 1414 are presented as an alignment. The exact 5'-terminus of DWV-B VDV-1 remained undetermined, rendering its available sequence 34 nucleotides shorter than that of DWV-B Austria-SB22 and DWV-A 1414. However, the 5'-end sequence of DWV-B strain SB22 perfectly matches that of DWV-A strain 1414, with no nucleotide discrepancies detected.

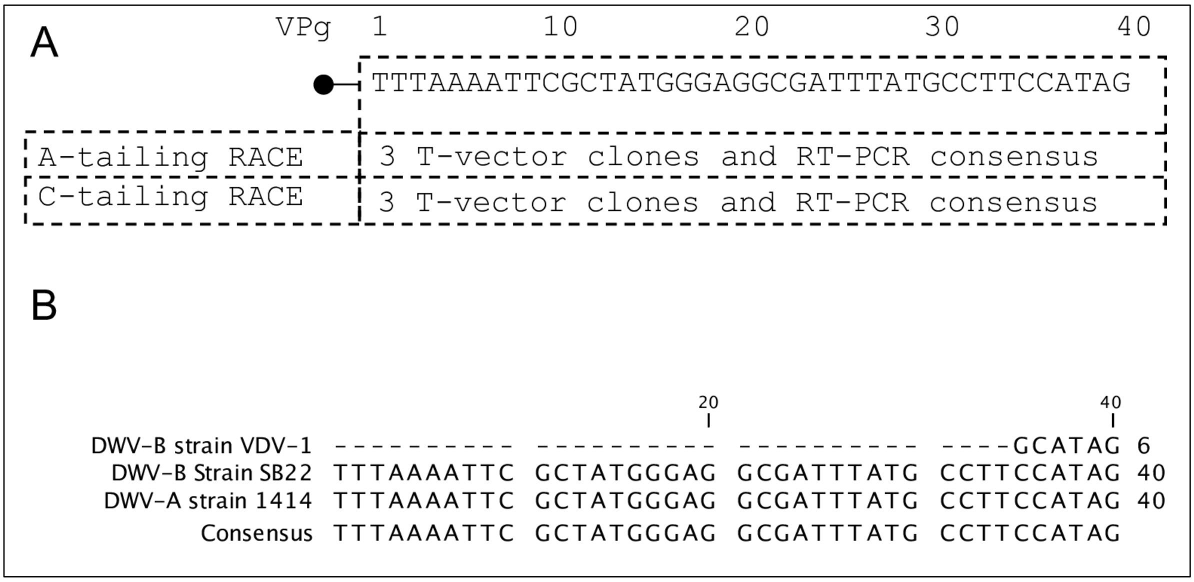

Figure S2. Genome alignment of DWVs. The nucleotide sequences of DWV-B strains Austria-SB22 (PP418870), VDV-1 (NC_006494), ER23 (OR361559), ER24 (OR361560), B11B (OL803829), 341-1 (ON648741), and VV144I (MN565037) were aligned with three DWV-A master variant strains, namely PA (AY292384), 1414 (KU847397), and Kakugo (AB070959). DWV master variants C and D were represented by strains Devon (European Nucleotide Archive ER5657949) and Egypt bee virus (MT504363), respectively. Strain NT-12 (MG995697) of Darwin bee virus 3 was included in the alignments as an outgroup due to its close relation. Multiple sequence alignments were conducted using CLC Genomics Workbench (Version 7.7.1, Qiagen) with default parameters.

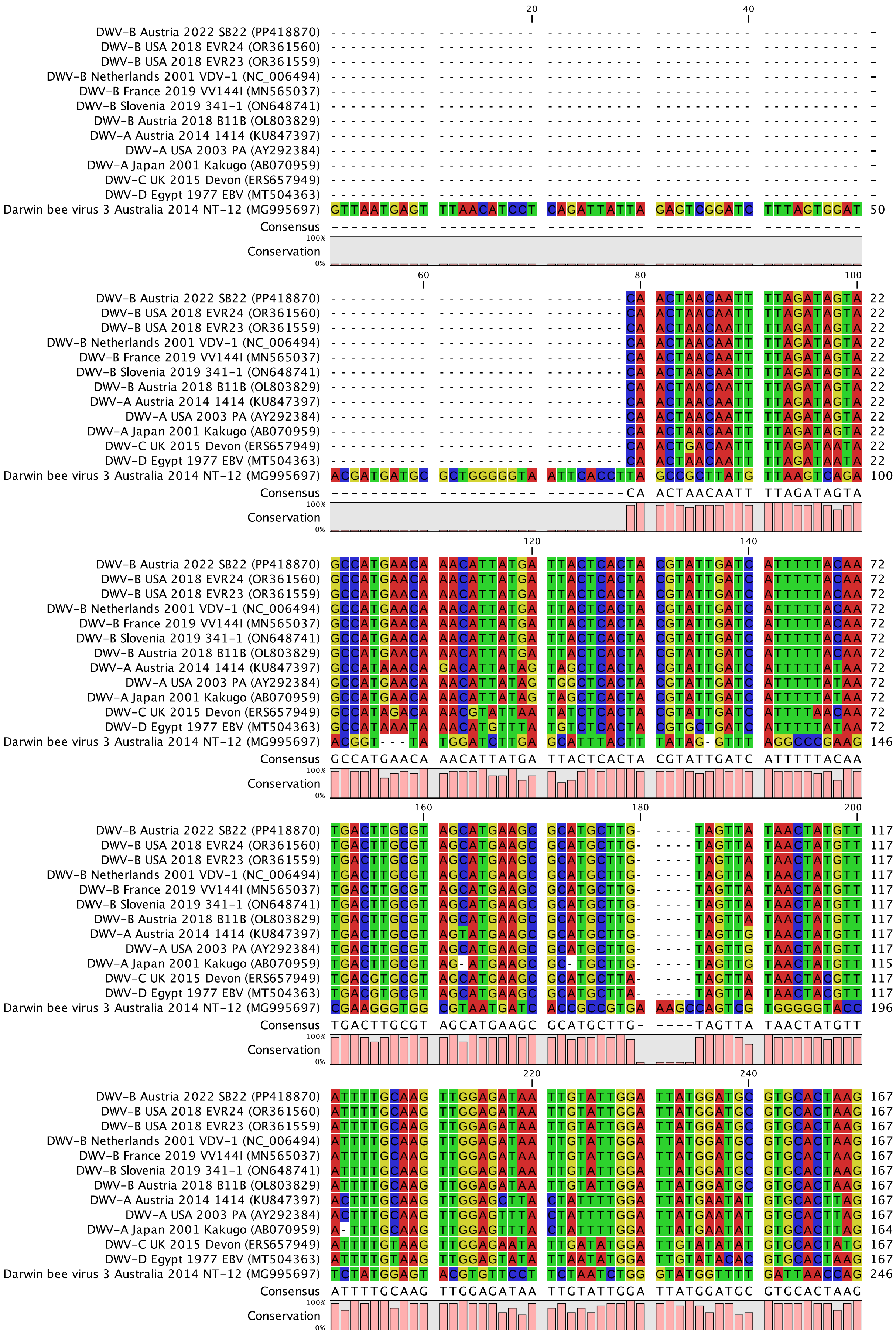
20 40
DWV-B Austria 2022 SB22 (PP418870) ---------- ---------- ---------- ---------- ---------- -
DWV-B USA 2018 EVR24 (OR361560) ---------- ---------- ---------- ---------- ---------- -
DWV-B USA 2018 EVR23 (OR361559) ---------- ---------- ---------- ---------- ---------- -
DWV-B Netherlands 2001 VDV-1 (NC_006494) ---------- ---------- ---------- ---------- ---------- -
DWV-B France 2019 VV144I (MN565037) ---------- ---------- ---------- ---------- ---------- -
DWV-B Slovenia 2019 341-1 (ON648741) ---------- ---------- ---------- ---------- ---------- -
DWV-B Austria 2018 B11B (OL803829) ---------- ---------- ---------- ---------- ---------- -
DWV-A Austria 2014 1414 (KU847397) ---------- ---------- ---------- ---------- ---------- -
DWV-A USA 2003 PA (AY292384) ---------- ---------- ---------- ---------- ---------- -
DWV-A Japan 2001 Kakugo (AB070959) ---------- ---------- ---------- ---------- ---------- -
DWV-C UK 2015 Devon (ERS657949) ---------- ---------- ---------- ---------- ---------- -
DWV-D Egypt 1977 EBV (MT504363) ---------- ---------- ---------- ---------- ---------- -
Darwin bee virus 3 Australia 2014 NT-12 (MG995697) GTTAATGAGT TTAACATCCT CAGATTATTA GAGTCGGATC TTTAGTGGAT 50
Consensus ---------- ---------- ---------- ---------- ----------
Conservation 100% 0%
60 80 100
DWV-B Austria 2022 SB22 (PP418870) ---------- ---------- --------CA ACTAACAATT TTAGATAGTA 22
DWV-B USA 2018 EVR24 (OR361560) ---------- ---------- --------CA ACTAACAATT TTAGATAGTA 22
DWV-B USA 2018 EVR23 (OR361559) ---------- ---------- --------CA ACTAACAATT TTAGATAGTA 22
DWV-B Netherlands 2001 VDV-1 (NC_006494) ---------- ---------- --------CA ACTAACAATT TTAGATAGTA 22
DWV-B France 2019 VV144I (MN565037) ---------- ---------- --------CA ACTAACAATT TTAGATAGTA 22
DWV-B Slovenia 2019 341-1 (ON648741) ---------- ---------- --------CA ACTAACAATT TTAGATAGTA 22
DWV-B Austria 2018 B11B (OL803829) ---------- ---------- --------CA ACTAACAATT TTAGATAGTA 22
DWV-A Austria 2014 1414 (KU847397) ---------- ---------- --------CA ACTAACAATT TTAGATAGTA 22
DWV-A USA 2003 PA (AY292384) ---------- ---------- --------CA ACTAACAATT TTAGATAGTA 22
DWV-A Japan 2001 Kakugo (AB070959) ---------- ---------- --------CA ACTAACAATT TTAGATAGTA 22
DWV-C UK 2015 Devon (ERS657949) ---------- ---------- --------CA ACTGACAATT TTAGATAATA 22
DWV-D Egypt 1977 EBV (MT504363) ---------- ---------- --------CA ACTAACAATT TTAGATAATA 22
Darwin bee virus 3 Australia 2014 NT-12 (MG995697) ACGATGATGC GCTGGGGGTA ATTCACCTTA GCCGCTTATG TTAAGTCAGA 100
Consensus ---------- ---------- --------CA ACTAACAATT TTAGATAGTA
Conservation 100% 0%
120 140
DWV-B Austria 2022 SB22 (PP418870) GCCATGAACA AACATTATGA TTACTCACTA CGTATTGATC ATTTTTACAA 72
DWV-B USA 2018 EVR24 (OR361560) GCCATGAACA AACATTATGA TTACTCACTA CGTATTGATC ATTTTTACAA 72
DWV-B USA 2018 EVR23 (OR361559) GCCATGAACA AACATTATGA TTACTCACTA CGTATTGATC ATTTTTACAA 72
DWV-B Netherlands 2001 VDV-1 (NC_006494) GCCATGAACA AACATTATGA TTACTCACTA CGTATTGATC ATTTTTACAA 72
DWV-B France 2019 VV144I (MN565037) GCCATGAACA AACATTATGA TTACTCACTA CGTATTGATC ATTTTTACAA 72
DWV-B Slovenia 2019 341-1 (ON648741) GCCATGAACA AACATTATGA TTACTCACTA CGTATTGATC ATTTTTACAA 72
DWV-B Austria 2018 B11B (OL803829) GCCATGAACA AACATTATGA TTACTCACTA CGTATTGATC ATTTTTACAA 72
DWV-A Austria 2014 1414 (KU847397) GCCATAAACA GACATTATAG TAGCTCACTA CGTATTGATC ATTTTTATAA 72
DWV-A USA 2003 PA (AY292384) GCCATGAACA AACATTATAG TGGCTCACTA CGTATTGATC ATTTTTATAA 72
DWV-A Japan 2001 Kakugo (AB070959) GCCATGAACA AACATTATAG TAGCTCACTA CGTATTGATC ATTTTTATAA 72
DWV-C UK 2015 Devon (ERS657949) GCCATAGACA AACGTATTAA TATCTCACTA CGTATTGATC ATTTTAACAA 72
DWV-D Egypt 1977 EBV (MT504363) GCCATAAATA AACATGTTTA TGTCTCACTA CGTGCTGATC ATTTTTATAA 72
Darwin bee virus 3 Australia 2014 NT-12 (MG995697) ACGGT---TA TGGATCTTGA GCATTTACTT TATAG-GTTT AGGCCCGAAG 146
Consensus GCCATGAACA AACATTATGA TTACTCACTA CGTATTGATC ATTTTTACAA
Conservation 100% 0%
160 180 200
DWV-B Austria 2022 SB22 (PP418870) TGACTTGCGT AGCATGAAGC GCATGCTTG- ----TAGTTA TAACTATGTT 117
DWV-B USA 2018 EVR24 (OR361560) TGACTTGCGT AGCATGAAGC GCATGCTTG- ----TAGTTA TAACTATGTT 117
DWV-B USA 2018 EVR23 (OR361559) TGACTTGCGT AGCATGAAGC GCATGCTTG- ----TAGTTA TAACTATGTT 117
DWV-B Netherlands 2001 VDV-1 (NC_006494) TGACTTGCGT AGCATGAAGC GCATGCTTG- ----TAGTTA TAACTATGTT 117
DWV-B France 2019 VV144I (MN565037) TGACTTGCGT AGCATGAAGC GCATGCTTG- ----TAGTTA TAACTATGTT 117
DWV-B Slovenia 2019 341-1 (ON648741) TGACTTGCGT AGCATGAAGC GCATGCTTG- ----TAGTTA TAACTATGTT 117
DWV-B Austria 2018 B11B (OL803829) TGACTTGCGT AGCATGAAGC GCATGCTTG- ----TAGTTA TAACTATGTT 117
DWV-A Austria 2014 1414 (KU847397) TGACTTGCGT AGTATGAAGC GCATGCTTG- ----TAGTTG TAACTATGTT 117
DWV-A USA 2003 PA (AY292384) TGACTTGCGT AGCATGAAGC GCATGCTTG- ----TAGTTG TAACTATGTT 117
DWV-A Japan 2001 Kakugo (AB070959) TGACTTGCGT AG-ATGAAGC GC-TGCTTG- ----TAGTTG TAACTATGTT 115
DWV-C UK 2015 Devon (ERS657949) TGACGTGCGT AGCATGAAGC GCATGCTTA- ----TAGTTA TAACTACGTT 117
DWV-D Egypt 1977 EBV (MT504363) TGACGTGCGT AGCATGAAGC GCATGCTTA- ----TAGTTA TAACTACGTT 117
Darwin bee virus 3 Australia 2014 NT-12 (MG995697) CGAAGGGTGG CGTAATGATC ACCGCCGTGA AAGCCAGTCG TGGGGGTACC 196
Consensus TGACTTGCGT AGCATGAAGC GCATGCTTG- ----TAGTTA TAACTATGTT
Conservation 100% 0%
220 240
DWV-B Austria 2022 SB22 (PP418870) ATTTTGCAAG TTGGAGATAA TTGTATTGGA TTATGGATGC GTGCACTAAG 167
DWV-B USA 2018 EVR24 (OR361560) ATTTTGCAAG TTGGAGATAA TTGTATTGGA TTATGGATGC GTGCACTAAG 167
DWV-B USA 2018 EVR23 (OR361559) ATTTTGCAAG TTGGAGATAA TTGTATTGGA TTATGGATGC GTGCACTAAG 167
DWV-B Netherlands 2001 VDV-1 (NC_006494) ATTTTGCAAG TTGGAGATAA TTGTATTGGA TTATGGATGC GTGCACTAAG 167
DWV-B France 2019 VV144I (MN565037) ATTTTGCAAG TTGGAGATAA TTGTATTGGA TTATGGATGC GTGCACTAAG 167
DWV-B Slovenia 2019 341-1 (ON648741) ATTTTGCAAG TTGGAGATAA TTGTATTGGA TTATGGATGC GTGCACTAAG 167
DWV-B Austria 2018 B11B (OL803829) ATTTTGCAAG TTGGAGATAA TTGTATTGGA TTATGGATGC GTGCACTAAG 167
DWV-A Austria 2014 1414 (KU847397) ACTTTGCAAG TTGGAGCTTA CTATTTTGGA TTATGAATAT GTGCACTTAG 167
DWV-A USA 2003 PA (AY292384) ACTTTGCAAG TTGGAGTTTA CTATTTTGGA TTATGAATAT GTGCACTTAG 167
DWV-A Japan 2001 Kakugo (AB070959) A-TTTGCAAG TTGGAGTTTA CTATTTTGGA TTATGAATAT GTGCACTTAG 164
DWV-C UK 2015 Devon (ERS657949) ATTTTGTAAG TTGGAGAATA TTGATATGGA TTGTATATAT GTGCACTATG 167
DWV-D Egypt 1977 EBV (MT504363) ATTTTGTAAG TTGGAGTATA TTAATATGGA TTGTATACAC GTGCACTAAG 167
Darwin bee virus 3 Australia 2014 NT-12 (MG995697) TCTATGGAGT ACGTGTTCCT TCTAATCTGG GTATGGTTTT GATTAACCAG 246
Consensus ATTTTGCAAG TTGGAGATAA TTGTATTGGA TTATGGATGC GTGCACTAAG
Conservation 100% 0%

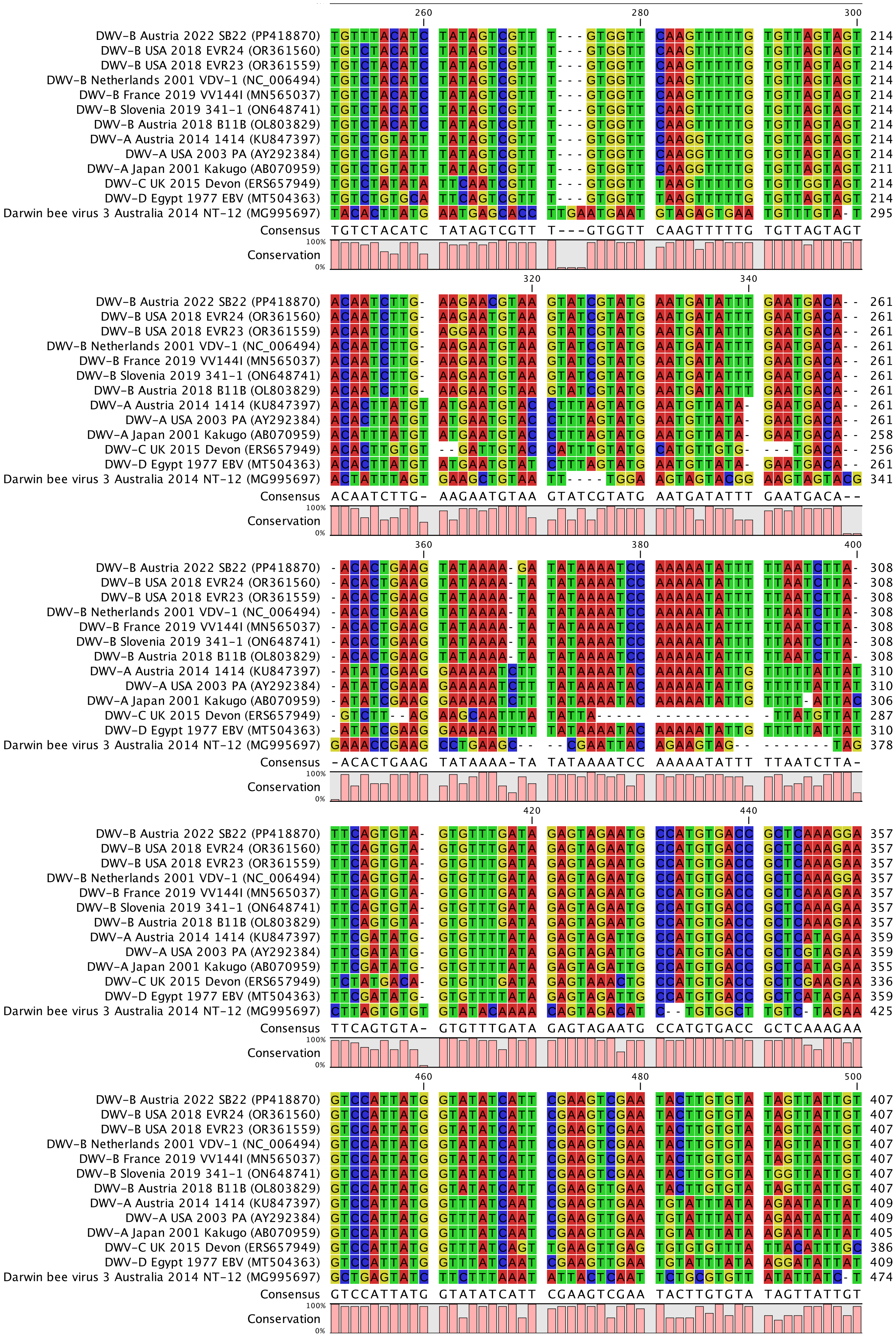
260 280 300
DWV-B Austria 2022 SB22 (PP418870) TGTTTACATC TATAGTCGTT T---GTGGTT CAAGTTTTTG TGTTAGTAGT 214
DWV-B USA 2018 EVR24 (OR361560) TGTCTACATC TATAGTCGTT T---GTGGTT CAAGTTTTTG TGTTAGTAGT 214
DWV-B USA 2018 EVR23 (OR361559) TGTCTACATC TATAGTCGTT T---GTGGTT CAAGTTTTTG TGTTAGTAGT 214
DWV-B Netherlands 2001 VDV-1 (NC_006494) TGTCTACATC TATAGTCGTT T---GTGGTT CAAGTTTTTG TGTTAGTAGT 214
DWV-B France 2019 VV144I (MN565037) TGTCTACATC TATAGTCGTT T---GTGGTT CAAGTTTTTG TGTTAGTAGT 214
DWV-B Slovenia 2019 341-1 (ON648741) TGTCTACATC TATAGTCGTT T---GTGGTT CAAGTTTTTG TGTTAGTAGT 214
DWV-B Austria 2018 B11B (OL803829) TGTCTACATC TATAGTCGTT T---GTGGTT CAAGTTTTTG TGTTAGTAGT 214
DWV-A Austria 2014 1414 (KU847397) TGTCTGTATT TATAGTCGTT T---GTGGTT CAAGGTTTTG TGTTAGTAGT 214
DWV-A USA 2003 PA (AY292384) TGTCTGTATT TATAGTCGTT T---GTGGTT CAAGGTTTTG TGTTAGTAGT 214
DWV-A Japan 2001 Kakugo (AB070959) TGTCTGTATT TATAGTCGTT T---GTGGTT CAAGGTTTTG TGTTAGTAGT 211
DWV-C UK 2015 Devon (ERS657949) TGTCTATATA TTCAATCGTT T---GTGGTT TAAGTTTTTG TGTTGGTAGT 214
DWV-D Egypt 1977 EBV (MT504363) TGTCTGTGCA TTCAGTCGTT T---GTGGTT TAAGTTTTTG TGTTAGTAGT 214
Darwin bee virus 3 Australia 2014 NT-12 (MG995697) TACACTTATG AATGAGCACC TTGAATGAAT GTAGAGTGAA TGTTTGTA-T 295
Consensus TGTCTACATC TATAGTCGTT T---GTGGTT CAAGTTTTTG TGTTAGTAGT
Conservation 100% 0%
320 340
DWV-B Austria 2022 SB22 (PP418870) ACAATCTTG- AAGAACGTAA GTATCGTATG AATGATATTT GAATGACA-- 261
DWV-B USA 2018 EVR24 (OR361560) ACAATCTTG- AAGAATGTAA GTATCGTATG AATGATATTT GAATGACA-- 261
DWV-B USA 2018 EVR23 (OR361559) ACAATCTTG- AGGAATGTAA GTATCGTATG AATGATATTT GAATGACA-- 261
DWV-B Netherlands 2001 VDV-1 (NC_006494) ACAATCTTG- AAGAATGTAA GTATCGTATG AATGATATTT GAATGACA-- 261
DWV-B France 2019 VV144I (MN565037) ACAATCTTG- AAGAATGTAA GTATCGTATG AATGATATTT GAATGACA-- 261
DWV-B Slovenia 2019 341-1 (ON648741) ACAATCTTG- AAGAATGTAA GTATCGTATG AATGATATTT GAATGACA-- 261
DWV-B Austria 2018 B11B (OL803829) ACAATCTTG- AAGAATGTAA GTATCGTATG AATGATATTT GAATGACA-- 261
DWV-A Austria 2014 1414 (KU847397) ACACTTATGT ATGAATGTAC CTTTAGTATG AATGTTATA- GAATGACA-- 261
DWV-A USA 2003 PA (AY292384) ACACTTATGT ATGAATGTAC CTTTAGTATG AATGTTATA- GAATGACA-- 261
DWV-A Japan 2001 Kakugo (AB070959) ACATTTATGT ATGAATGTAC CTTTAGTATG AATGTTATA- GAATGACA-- 258
DWV-C UK 2015 Devon (ERS657949) ACACTTGTGT --GATTGTAC CATTTGTATG CATGTTGTG- ---TGACA-- 256
DWV-D Egypt 1977 EBV (MT504363) ACACTTATGT ATGAATGTAT CTTTAGTATG AATGTTATA- GAATGACA-- 261
Darwin bee virus 3 Australia 2014 NT-12 (MG995697) ACTATTTAGT GAAGCTGTAA TT----TGGA AGTAGTACGG AAGTAGTACG 341
Consensus ACAATCTTG- AAGAATGTAA GTATCGTATG AATGATATTT GAATGACA--
Conservation 100% 0%
360 380 400
DWV-B Austria 2022 SB22 (PP418870) -ACACTGAAG TATAAAA-GA TATAAAATCC AAAAATATTT TTAATCTTA- 308
DWV-B USA 2018 EVR24 (OR361560) -ACACTGAAG TATAAAA-TA TATAAAATCC AAAAATATTT TTAATCTTA- 308
DWV-B USA 2018 EVR23 (OR361559) -ACACTGAAG TATAAAA-TA TATAAAATCC AAAAATATTT TTAATCTTA- 308
DWV-B Netherlands 2001 VDV-1 (NC_006494) -ACACTGAAG TATAAAA-TA TATAAAATCC AAAAATATTT TTAATCTTA- 308
DWV-B France 2019 VV144I (MN565037) -ACACTGAAG TATAAAA-TA TATAAAATCC AAAAATATTT TTAATCTTA- 308
DWV-B Slovenia 2019 341-1 (ON648741) -ACACTGAAG TATAAAA-TA TATAAAATCC AAAAATATTT TTAATCTTA- 308
DWV-B Austria 2018 B11B (OL803829) -ACACTGAAG TATAAAA-TA TATAAAATCC AAAAATATTT TTAATCTTA- 308
DWV-A Austria 2014 1414 (KU847397) -ATATCGAAG GAAAAATCTT TATAAAATAC AAAAATATTG TTTTTATTAT 310
DWV-A USA 2003 PA (AY292384) -ATATCGAAA GAAAAATCTT TATAAAATAC AAAAATATTG TTTTTATTAT 310
DWV-A Japan 2001 Kakugo (AB070959) -ATATCGAAG GAAAAATCTT TATAAAATAC AAAAATATTG TTTT-ATTAC 306
DWV-C UK 2015 Devon (ERS657949) -GTCTT--AG AAGCAATTTA TATTA----- ---------- -TTATGTTAT 287
DWV-D Egypt 1977 EBV (MT504363) -ATATCGAAG GAAAAATTTT TATAAAATAC AAAAATATTG TTTTTATTAT 310
Darwin bee virus 3 Australia 2014 NT-12 (MG995697) GAAACCGAAG CCTGAAGC-- --CGAATTAC AGAAGTAG-- -------TAG 378
Consensus -ACACTGAAG TATAAAA-TA TATAAAATCC AAAAATATTT TTAATCTTA-
Conservation 100% 0%
420 440
DWV-B Austria 2022 SB22 (PP418870) TTCAGTGTA- GTGTTTGATA GAGTAGAATG CCATGTGACC GCTCAAAGGA 357
DWV-B USA 2018 EVR24 (OR361560) TTCAGTGTA- GTGTTTGATA GAGTAGAATG CCATGTGACC GCTCAAAGAA 357
DWV-B USA 2018 EVR23 (OR361559) TTCAGTGTA- GTGTTTGATA GAGTAGAATG CCATGTGACC GCTCAAAGAA 357
DWV-B Netherlands 2001 VDV-1 (NC_006494) TTCAGTGTA- GTGTTTGATA GAGTAGAATG CCATGTGACC GCTCAAAGGA 357
DWV-B France 2019 VV144I (MN565037) TTCAGTGTA- GTGTTTGATA GAGTAGAATG CCATGTGACC GCTCAAAGAA 357
DWV-B Slovenia 2019 341-1 (ON648741) TTCAGTGTA- GTGTTTGATA GAGTAGAATG CCATGTGACC GCTCAAAGAA 357
DWV-B Austria 2018 B11B (OL803829) TTCAGTGTA- GTGTTTGATA GAGTAGAATG CCATGTGACC GCTCAAAGAA 357
DWV-A Austria 2014 1414 (KU847397) TTCGATATG- GTGTTTTATA GAGTAGATTG CCATGTGACC GCTCATAGAA 359
DWV-A USA 2003 PA (AY292384) TTCGATATG- GTGTTTTATA GAGTAGATTG CCATGTGACC GCTCGTAGAA 359
DWV-A Japan 2001 Kakugo (AB070959) TTCGATATG- GTGTTTTATA GAGTAGATTG CCATGTGACC GCTCATAGAA 355
DWV-C UK 2015 Devon (ERS657949) TCTATGACA- GTGTTTGATA GAGTAAACTG CCATGTGACC GCTCGAAGAA 336
DWV-D Egypt 1977 EBV (MT504363) TTCGATATG- GTGTTTTATA GAGTAGATTG CCATGTGACC GCTCATAGAA 359
Darwin bee virus 3 Australia 2014 NT-12 (MG995697) CTTAGTGTGT GTATACAAAA CAGTAGACAT C--TGTGGCT TGTC-TAGAA 425
Consensus TTCAGTGTA- GTGTTTGATA GAGTAGAATG CCATGTGACC GCTCAAAGAA
Conservation 100% 0%
460 480 500
DWV-B Austria 2022 SB22 (PP418870) GTCCATTATG GTATATCATT CGAAGTCGAA TACTTGTGTA TAGTTATTGT 407
DWV-B USA 2018 EVR24 (OR361560) GTCCATTATG GTATATCATT CGAAGTCGAA TACTTGTGTA TAGTTATTGT 407
DWV-B USA 2018 EVR23 (OR361559) GTCCATTATG GTATATCATT CGAAGTCGAA TACTTGTGTA TAGTTATTGT 407
DWV-B Netherlands 2001 VDV-1 (NC_006494) GTCCATTATG GTATATCATT CGAAGTCGAA TACTTGTGTA TAGTTATTGT 407
DWV-B France 2019 VV144I (MN565037) GTCCATTATG GTATATCATT CGAAGTCGAA TACTTGTGTA TAGTTATTGT 407
DWV-B Slovenia 2019 341-1 (ON648741) GTCCATTATG GTATATCATT CGAAGTCGAA TACTTGTGTA TGGTTATTGT 407
DWV-B Austria 2018 B11B (OL803829) GTCCATTATG GTATATCATT CGAAGTCGAA TACTTGTGTA TAGTTATTGT 407
DWV-A Austria 2014 1414 (KU847397) GTCCATTATG GTTTATCAAT CGAAGTTGAA TGTATTTATA AGAATATTAT 409
DWV-A USA 2003 PA (AY292384) GTCCATTATG GTTTATCAAT CGAAGTTGAA TGTATTTATA AGAATATTAT 409
DWV-A Japan 2001 Kakugo (AB070959) GTCCATTATG GTTTATCAAT CGAAGTTGAA TGTATTTATA AGAATATTAT 405
DWV-C UK 2015 Devon (ERS657949) GTCCATTATG GTTTATCAGT TGAAGTTGAG TGTGTGTTTA TTACATTTGC 386
DWV-D Egypt 1977 EBV (MT504363) GTCCATTATG GTTTATCAAT CGAAGTTGAA TGTATTTATA AGGATATTAT 409
Darwin bee virus 3 Australia 2014 NT-12 (MG995697) GCTGAGTATC TTCTTTAAAT ATTACTCAAT TCTGCGTGTT ATATTATC-T 474
Consensus GTCCATTATG GTATATCATT CGAAGTCGAA TACTTGTGTA TAGTTATTGT
Conservation 100% 0%

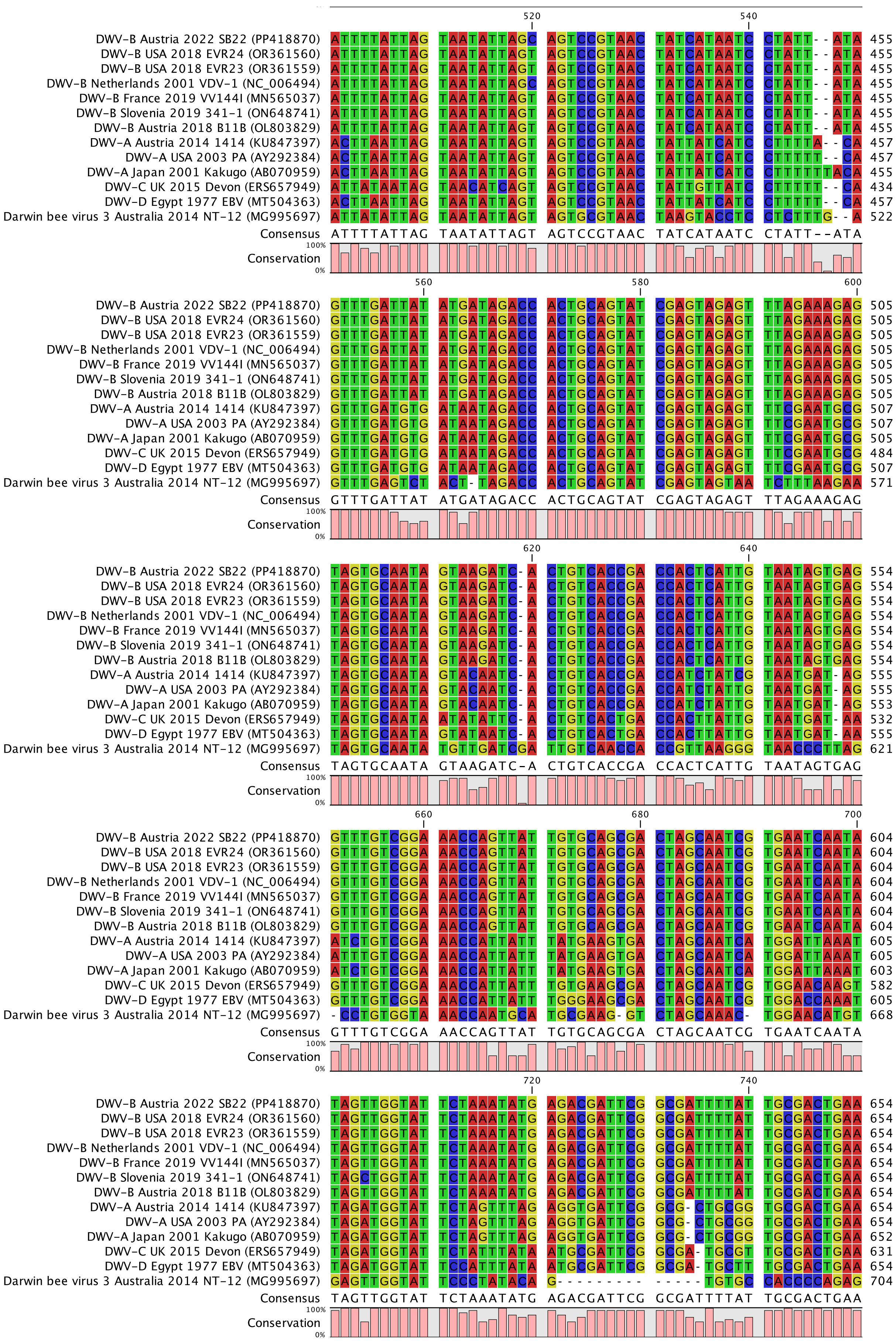

520
540
DWV-B Austria 2022 SB22 (PP418870) 455
DWV-B USA 2018 EVR24 (OR361560) 455
DWV-B USA 2018 EVR23 (OR361559) 455
DWV-B Netherlands 2001 VDV-1 (NC_006494) 455
DWV-B France 2019 VV1441 (MN565037) 455
DWV-B Slovenia 2019 341-1 (ON648741) 455
DWV-B Austria 2018 B11B (OL803829) 455
DWV-A Austria 2014 1414 (KU847397) 457
DWV-A USA 2003 PA (AY292384) 457
DWV-A Japan 2001 Kakugo (AB070959) 455
DWV-C UK 2015 Devon (ERS657949) 434
DWV-D Egypt 1977 EBV (MT504363) 457
Darwin bee virus 3 Australia 2014 NT-12 (MG995697) 522
Consensus ATTTTATTAG TAATATTAGT AGTCCGTAAC TATCATAATC CTATT--ATA
Conservation
560
580
600
Consensus GTTTGATTAT ATGATAGACC ACTGCAGTAT CGAGTAGAGT TTAGAAAGAG
620
640
Consensus TAGTGCAATA GTAAGATC-A CTGTCACCGA CCACTCATTG TAATAGTGAG
660
680
700
Consensus GTTTGTCGGA AACCAGTTAT TGTGCAGCGA CTAGCAATCG TGAATCAATA
720
740
Consensus TAGTTGGTAT TCTAAATATG AGACGATTCG GCGATTTTAT TGCGACTGAA

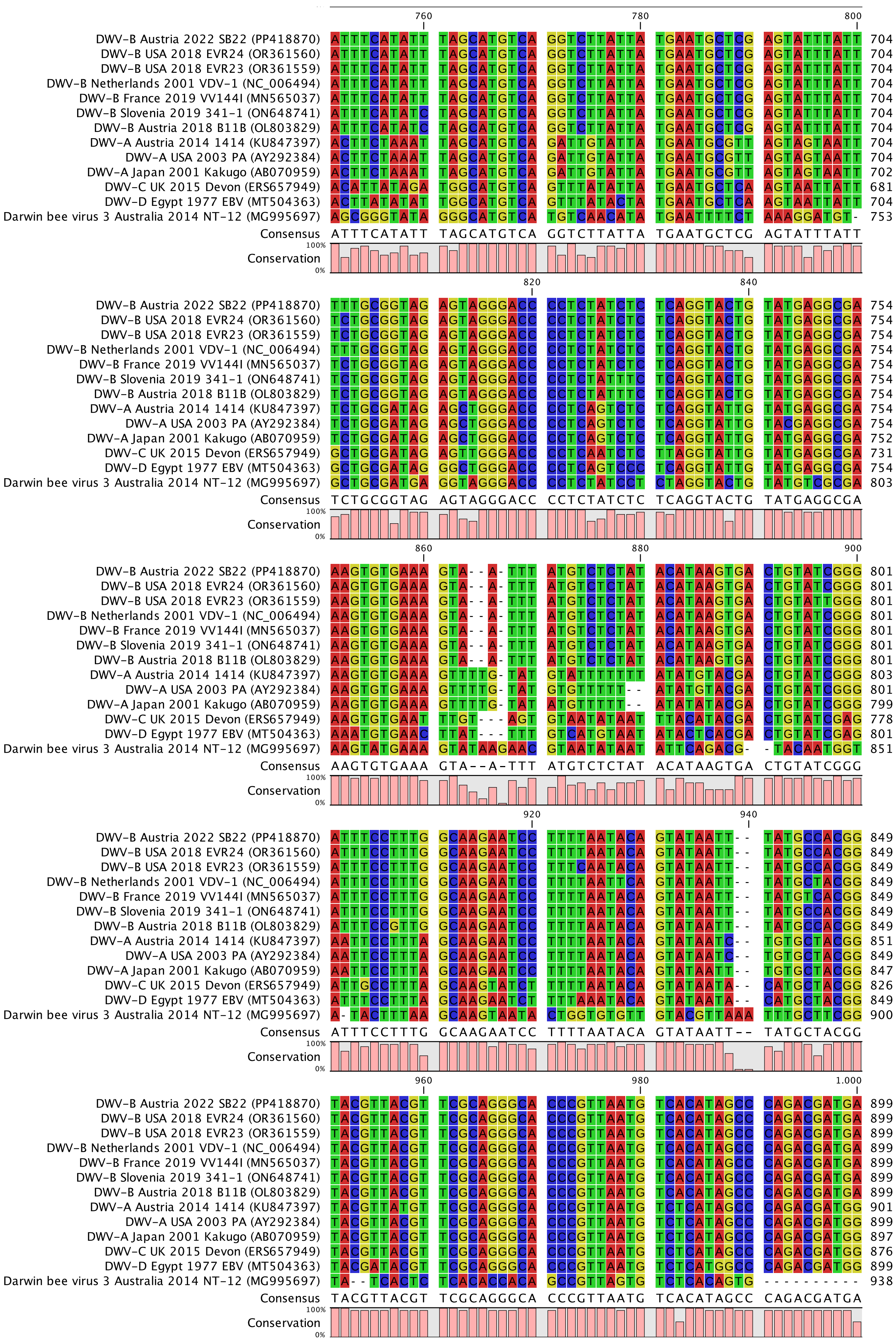
760 780 800
DWV-B Austria 2022 SB22 (PP418870) ATTTCATATT TAGCATGTCA GGTCTTATTA TGAATGCTCG AGTATTTATT 704
DWV-B USA 2018 EVR24 (OR361560) ATTTCATATT TAGCATGTCA GGTCTTATTA TGAATGCTCG AGTATTTATT 704
DWV-B USA 2018 EVR23 (OR361559) ATTTCATATT TAGCATGTCA GGTCTTATTA TGAATGCTCG AGTATTTATT 704
DWV-B Netherlands 2001 VDV-1 (NC_006494) ATTTCATATT TAGCATGTCA GGTCTTATTA TGAATGCTCG AGTATTTATT 704
DWV-B France 2019 VV144I (MN565037) ATTTCATATT TAGCATGTCA GGTCTTATTA TGAATGCTCG AGTATTTATT 704
DWV-B Slovenia 2019 341-1 (ON648741) ATTTCATATC TAGCATGTCA GGTCTTATTA TGAATGCTCG AGTATTTATT 704
DWV-B Austria 2018 B11B (OL803829) ATTTCATATC TAGCATGTCA GGTCTTATTA TGAATGCTCG AGTATTTATT 704
DWV-A Austria 2014 1414 (KU847397) ACTTCTAAAT TAGCATGTCA GATTGTATTA TGAATGCGTT AGTAGTAATT 704
DWV-A USA 2003 PA (AY292384) ACTTCTAAAT TAGCATGTCA GATTGTATTA TGAATGCGTT AGTAGTAATT 704
DWV-A Japan 2001 Kakugo (AB070959) ACTTCTAAAT TAGCATGTCA GATTGTATTA TGAATGCGTT AGTAGTAATT 702
DWV-C UK 2015 Devon (ERS657949) ACATTATAGA TGGCATGTCA GTTTATATTA TGAATGCTCA AGTAATTATT 681
DWV-D Egypt 1977 EBV (MT504363) ACTTATATAT TGGCATGTCA GTTTATACTA TGAATGCTCA AGTAATTATT 704
Darwin bee virus 3 Australia 2014 NT-12 (MG995697) AGCGGGTATA GGGCATGTCA TGTCAACATA TGAATTTTCT AAAGGATGT- 753
Consensus ATTTCATATT TAGCATGTCA GGTCTTATTA TGAATGCTCG AGTATTTATT
100%
Conservation
0%
820 840
DWV-B Austria 2022 SB22 (PP418870) TTTGCGGTAG AGTAGGGACC CCTCTATCTC TCAGGTACTG TATGAGGCGA 754
DWV-B USA 2018 EVR24 (OR361560) TCTGCGGTAG AGTAGGGACC CCTCTATCTC TCAGGTACTG TATGAGGCGA 754
DWV-B USA 2018 EVR23 (OR361559) TCTGCGGTAG AGTAGGGACC CCTCTATCTC TCAGGTACTG TATGAGGCGA 754
DWV-B Netherlands 2001 VDV-1 (NC_006494) TTTGCGGTAG AGTAGGGACC CCTCTATCTC TCAGGTACTG TATGAGGCGA 754
DWV-B France 2019 VV144I (MN565037) TCTGCGGTAG AGTAGGGACC CCTCTATCTC TCAGGTACTG TATGAGGCGA 754
DWV-B Slovenia 2019 341-1 (ON648741) TCTGCGGTAG AGTAGGGACC CCTCTATTTC TCAGGTACTG TATGAGGCGA 754
DWV-B Austria 2018 B11B (OL803829) TCTGCGGTAG AGTAGGGACC CCTCTATTTC TCAGGTACTG TATGAGGCGA 754
DWV-A Austria 2014 1414 (KU847397) TCTGCGATAG AGCTGGGACC CCTCAGTCTC TCAGGTATTG TATGAGGCGA 754
DWV-A USA 2003 PA (AY292384) TCTGCGATAG AGCTGGGACC CCTCAGTCTC TCAGGTATTG TACGAGGCGA 754
DWV-A Japan 2001 Kakugo (AB070959) TCTGCGATAG AGCTGGGACC CCTCAGTCTC TCAGGTATTG TATGAGGCGA 752
DWV-C UK 2015 Devon (ERS657949) GCTGCGATAG AGTTGGGACC CCTCAATCTC TTAGGTATTG TATGAGGCGA 731
DWV-D Egypt 1977 EBV (MT504363) GCTGCGATAG GGCTGGGACC CCTCAGTCCC TCAGGTATTG TATGAGGCGA 754
Darwin bee virus 3 Australia 2014 NT-12 (MG995697) GCTGCGATGA GGTAGGGACC CCTCTATCCT CTAGGTACTG TATGTCGCGA 803
Consensus TCTGCGGTAG AGTAGGGACC CCTCTATCTC TCAGGTACTG TATGAGGCGA
100%
Conservation
0%
860 880 900
DWV-B Austria 2022 SB22 (PP418870) AAGTGTGAAA GTA--A-TTT ATGTCTCTAT ACATAAGTGA CTGTATCGGG 801
DWV-B USA 2018 EVR24 (OR361560) AAGTGTGAAA GTA--A-TTT ATGTCTCTAT ACATAAGTGA CTGTATCGGG 801
DWV-B USA 2018 EVR23 (OR361559) AAGTGTGAAA GTA--A-TTT ATGTCTCTAT ACATAAGTGA CTGTATTGGG 801
DWV-B Netherlands 2001 VDV-1 (NC_006494) AAGTGTGAAA GTA--A-TTT ATGTCTCTAT ACATAAGTGA CTGTATCGGG 801
DWV-B France 2019 VV144I (MN565037) AAGTGTGAAA GTA--A-TTT ATGTCTCTAT ACATAAGTGA CTGTATCGGG 801
DWV-B Slovenia 2019 341-1 (ON648741) AAGTGTGAAA GTA--A-TTT ATGTCTCTAT ACATAAGTGA CTGTATCGGG 801
DWV-B Austria 2018 B11B (OL803829) AAGTGTGAAA GTA--A-TTT ATGTCTCTAT ACATAAGTGA CTGTATCGGG 801
DWV-A Austria 2014 1414 (KU847397) AAGTGTGAAA GTTTTG-TAT GTATTTTTTT ATATGTACGA CTGTATCGGG 803
DWV-A USA 2003 PA (AY292384) AAGTGTGAAA GTTTTG-TAT GTGTTTTT-- ATATGTACGA CTGTATCGGG 801
DWV-A Japan 2001 Kakugo (AB070959) AAGTGTGAAA GTTTTG-TAT ATGTTTTT-- ATATATACGA CTGTATCGGG 799
DWV-C UK 2015 Devon (ERS657949) AAGTGTGAAT TTGT---AGT GTAATATAAT TTACATACGA CTGTATCGAG 778
DWV-D Egypt 1977 EBV (MT504363) AAATGTGAAC TTAT---TTT GTCATGTAAT ATACTCACGA CTGTATCGAG 801
Darwin bee virus 3 Australia 2014 NT-12 (MG995697) AAGTATGAAA GTATAAGAAC GTAATATAAT ATTCAGACG- -TACAATGGT 851
Consensus AAGTGTGAAA GTA--A-TTT ATGTCTCTAT ACATAAGTGA CTGTATCGGG
100%
Conservation
0%
920 940
DWV-B Austria 2022 SB22 (PP418870) ATTTCCTTTG GCAAGAATCC TTTTAATACA GTATAATT-- TATGCCACGG 849
DWV-B USA 2018 EVR24 (OR361560) ATTTCCTTTG GCAAGAATCC TTTTAATACA GTATAATT-- TATGCCACGG 849
DWV-B USA 2018 EVR23 (OR361559) ATTTCCTTTG GCAAGAATCC TTTCAATACA GTATAATT-- TATGCCACGG 849
DWV-B Netherlands 2001 VDV-1 (NC_006494) ATTTCCTTTG GCAAGAATCC TTTTAATTCA GTATAATT-- TATGCTACGG 849
DWV-B France 2019 VV144I (MN565037) ATTTCCTTTG GCAAGAATCC TTTTAATACA GTATAATT-- TATGTCACGG 849
DWV-B Slovenia 2019 341-1 (ON648741) ATTTCCTTTG GCAAGAATCC TTTTAATACA GTATAATT-- TATGCCACGG 849
DWV-B Austria 2018 B11B (OL803829) ATTTCCGTTG GCAAGAATCC TTTTAATACA GTATAATT-- TATGCCACGG 849
DWV-A Austria 2014 1414 (KU847397) AATTCCTTTA GCAAGAATCC TTTTAATACA GTATAATC-- TGTGCTACGG 851
DWV-A USA 2003 PA (AY292384) AATTCCTTTA GCAAGAATCC TTTTAATACA GTATAATC-- TGTGCTACGG 849
DWV-A Japan 2001 Kakugo (AB070959) AATTCCTTTA GCAAGAATCC TTTTAATACA GTATAATT-- TGTGCTACGG 847
DWV-C UK 2015 Devon (ERS657949) ATTGCCTTTA GCAAGTATCT TTTTAATACA GTATAATA-- CATGCTACGG 826
DWV-D Egypt 1977 EBV (MT504363) ATTTCCTTTA GCAAGAATCT TTTAAATACA GTATAATA-- CATGCTACGG 849
Darwin bee virus 3 Australia 2014 NT-12 (MG995697) A-TACTTTAA GCAAGTAATA CTGGTGTGTT GTACGTTAAA TTTGCTTCGG 900
Consensus ATTTCCTTTG GCAAGAATCC TTTTAATACA GTATAATT-- TATGCTACGG
100%
Conservation
0%
960 980 1.000
DWV-B Austria 2022 SB22 (PP418870) TACGTTACGT TCGCAGGGCA CCCGTTAATG TCACATAGCC CAGACGATGA 899
DWV-B USA 2018 EVR24 (OR361560) TACGTTACGT TCGCAGGGCA CCCGTTAATG TCACATAGCC CAGACGATGA 899
DWV-B USA 2018 EVR23 (OR361559) TACGTTACGT TCGCAGGGCA CCCGTTAATG TCACATAGCC CAGACGATGA 899
DWV-B Netherlands 2001 VDV-1 (NC_006494) TACGTTACGT TCGCAGGGCA CCCGTTAATG TCACATAGCC CAGACGATGA 899
DWV-B France 2019 VV144I (MN565037) TACGTTACGT TCGCAGGGCA CCCGTTAATG TCACATAGCC CAGACGATGA 899
DWV-B Slovenia 2019 341-1 (ON648741) TACGTTACGT TCGCAGGGCA CCCGTTAATG TCACATAGCC CAGACGATGA 899
DWV-B Austria 2018 B11B (OL803829) TACGTTACGT TCGCAGGGCA CCCGTTAATG TCACATAGCC CAGACGATGA 899
DWV-A Austria 2014 1414 (KU847397) TACGTTATGT TCGCAGGGCA CCCGTTAATG TCTCATAGCC CAGACGATGG 901
DWV-A USA 2003 PA (AY292384) TACGTTACGT TCGCAGGGCA CCCGTTAATG TCTCATAGCC CAGACGATGG 899
DWV-A Japan 2001 Kakugo (AB070959) TACGTTACGT TCGCAGGGCA CCCGTTAATG TCTCATAGCC CAGACGATGG 897
DWV-C UK 2015 Devon (ERS657949) TACGTTACGT TCGCAGGGCA CCCGTTAATG TCTCATAGCC CAGACGATGG 876
DWV-D Egypt 1977 EBV (MT504363) TACGATACGT TCGCAGGGCA CCCGTTAATG TCTCATGGCC CAGACGATGG 899
Darwin bee virus 3 Australia 2014 NT-12 (MG995697) TA--TCACTC TCACACCACA GCCGTTAGTG TCTCACAGTG ---------- 938
Consensus TACGTTACGT TCGCAGGGCA CCCGTTAATG TCACATAGCC CAGACGATGA
100%
Conservation
0%

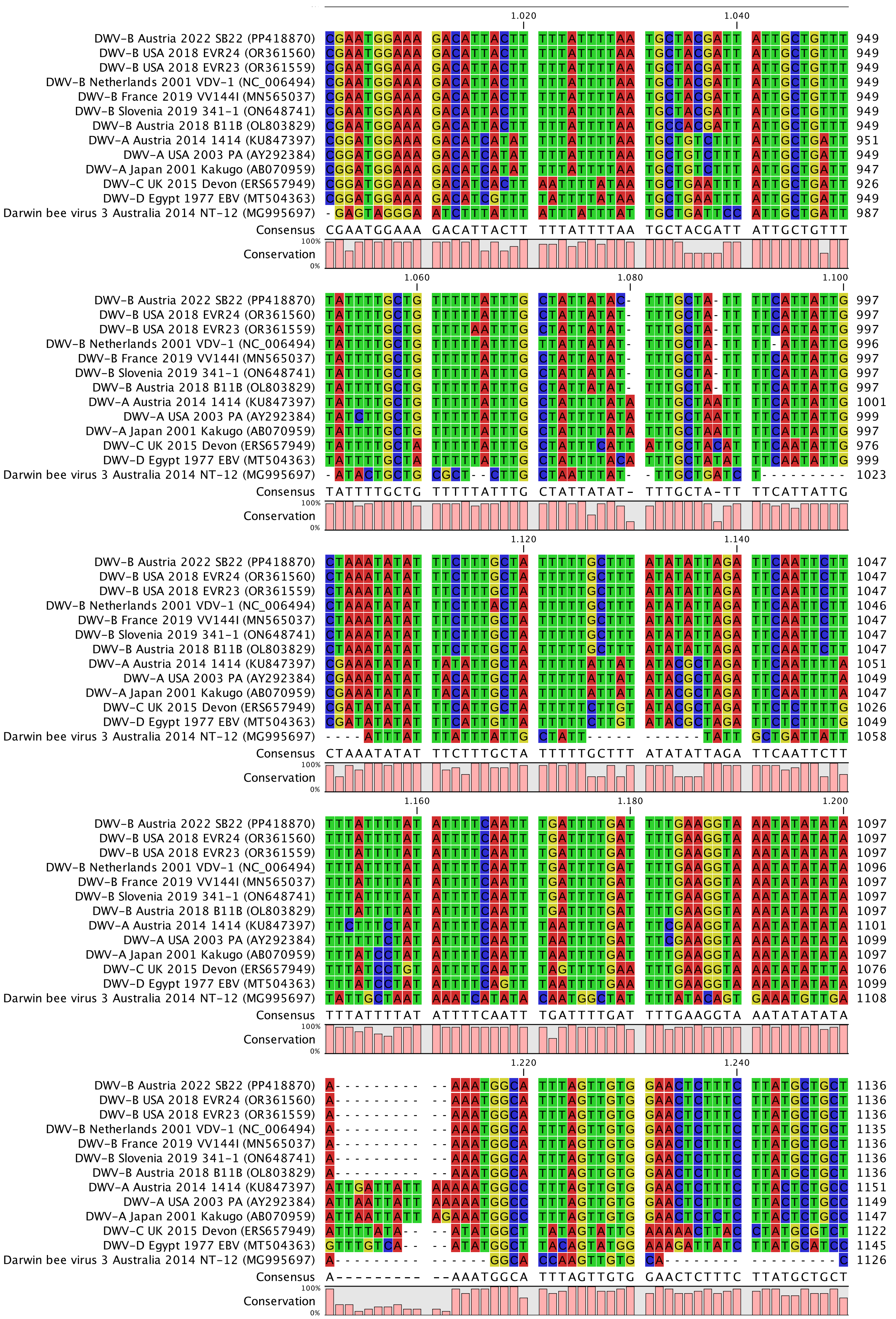
1.020
1.040
1.060
1.080
1.100
1.120
1.140
1.160
1.180
1.200
1.220
1.240
DWV-B Austria 2022 SB22 (PP418870)
DWV-B USA 2018 EVR24 (OR361560)
DWV-B USA 2018 EVR23 (OR361559)
DWV-B Netherlands 2001 VDV-1 (NC_006494)
DWV-B France 2019 VV144I (MN565037)
DWV-B Slovenia 2019 341-1 (ON648741)
DWV-B Austria 2018 B11B (OL803829)
DWV-A Austria 2014 1414 (KU847397)
DWV-A USA 2003 PA (AY292384)
DWV-A Japan 2001 Kakugo (AB070959)
DWV-C UK 2015 Devon (ERS657949)
DWV-D Egypt 1977 EBV (MT504363)
Darwin bee virus 3 Australia 2014 NT-12 (MG995697)
Consensus CGAATGGAAA GACATTACTT TTTATTTTAA TGCTACGATT ATTGCTGTTT
Consensus TATTTTGCTG TTTTTATTTG CTATTATAT- TTTGCTA-TT TTCATTATTG
Consensus CTAAATATAT TTCTTTGCTA TTTTTGCTTT ATATATTAGA TTCAATTCTT
Consensus TTTATTTTAT ATTTTCAATT TGATTTTGAT TTTGAAGGTA AATATATATA
Consensus A--------- --AAATGGCA TTTAGTTGTG GAACTCTTTC TTATGCTGCT
Conservation
100%
0%

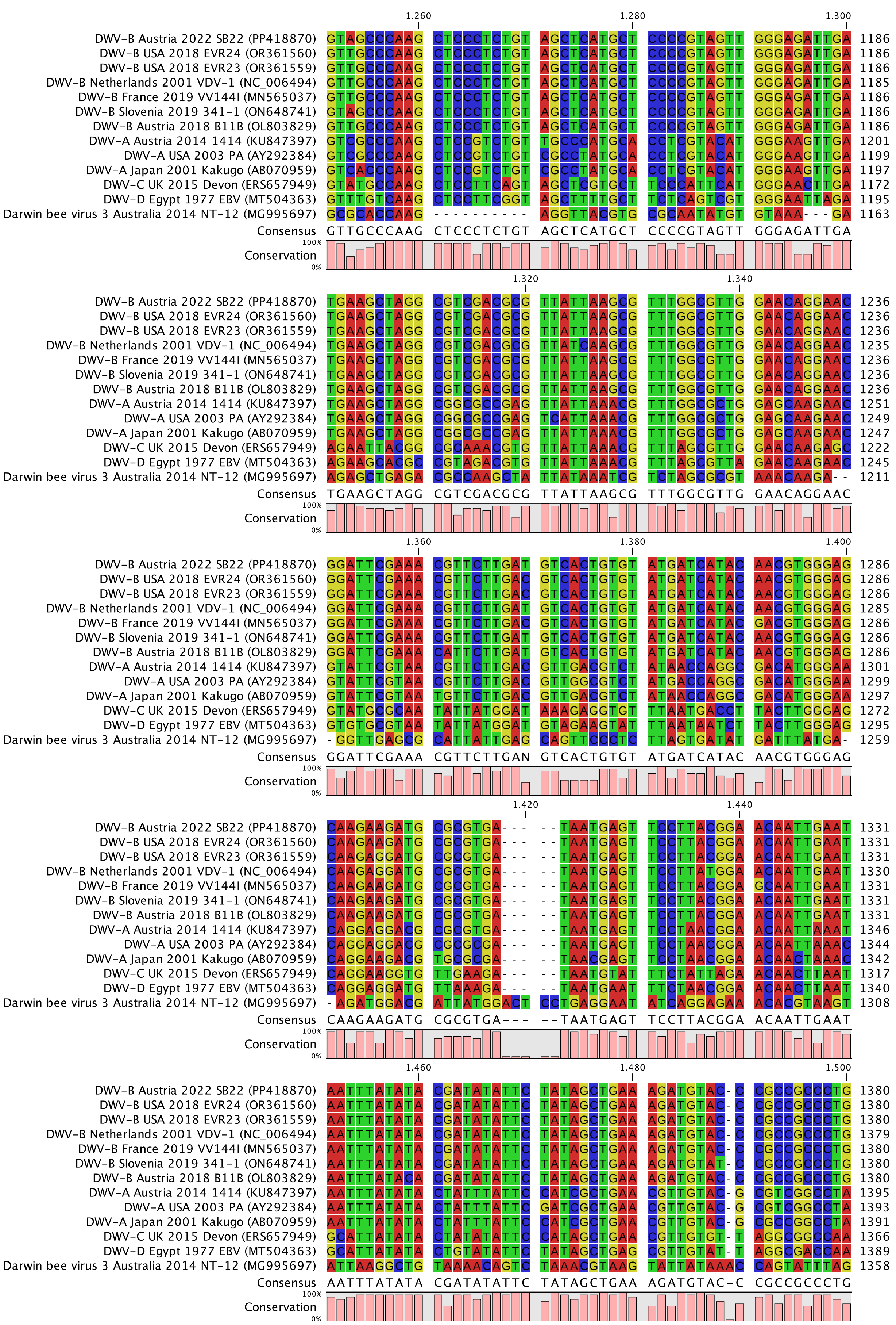

1.260 1.280 1.300
DWV-B Austria 2022 SB22 (PP418870) GTAGCCCAAG CTCCCTCTGT AGCTCATGCT CCCCGTAGTT GGGAGATTGA 1186
DWV-B USA 2018 EVR24 (OR361560) GTTGCCCAAG CTCCCTCTGT AGCTCATGCT CCCCGTAGTT GGGAGATTGA 1186
DWV-B USA 2018 EVR23 (OR361559) GTTGCCCAAG CTCCCTCTGT AGCTCATGCT CCCCGTAGTT GGGAGATTGA 1186
DWV-B Netherlands 2001 VDV-1 (NC_006494) GTTGCCCAAG CTCCCTCTGT AGCTCATGCT CCCCGTAGTT GGGAGATTGA 1185
DWV-B France 2019 VV144I (MN565037) GTTGCCCAAG CTCCCTCTGT AGCTCATGCT CCCCGTAGTT GGGAGATTGA 1186
DWV-B Slovenia 2019 341-1 (ON648741) GTAGCCCAAG CTCCCTCTGT AGCTCATGCT CCCCGTAGTT GGGAGATTGA 1186
DWV-B Austria 2018 B11B (OL803829) GTTGCCCAAG CTCCCTCTGT AGCTCATGCT CCCCGTAGTT GGGAGATTGA 1186
DWV-A Austria 2014 1414 (KU847397) GTCGCCCAAG CTCCGTCTGT TGCCCATGCA CCTCGTACAT GGGAAGTTGA 1201
DWV-A USA 2003 PA (AY292384) GTCGCCCAAG CTCCGTCTGT CGCCTATGCA CCTCGTACAT GGGAAGTTGA 1199
DWV-A Japan 2001 Kakugo (AB070959) GTCACCCAAG CTCCGTCTGT CGCCTATGCA CCTCGTACAT GGGAAGTTGA 1197
DWV-C UK 2015 Devon (ERS657949) GTATGCCAAG CTCCTTCAGT AGCTCGTGCT TCCCATTCAT GGGAACTTGA 1172
DWV-D Egypt 1977 EBV (MT504363) GTTTGTCAAG CTCCTTCGGT AGCTTTTGCT TCTCAGTCGT GGGAATTAGA 1195
Darwin bee virus 3 Australia 2014 NT-12 (MG995697) GCGCACCAAG ---------- AGGTTACGTG CGCAATATGT GTAAA---GA 1163
Consensus GTTGCCCAAG CTCCCTCTGT AGCTCATGCT CCCCGTAGTT GGGAGATTGA
100%
Conservation
0%
1.320 1.340
DWV-B Austria 2022 SB22 (PP418870) TGAAGCTAGG CGTCGACGCG TTATTAAGCG TTTGGCGTTG GAACAGGAAC 1236
DWV-B USA 2018 EVR24 (OR361560) TGAAGCTAGG CGTCGACGCG TTATTAAGCG TTTGGCGTTG GAACAGGAAC 1236
DWV-B USA 2018 EVR23 (OR361559) TGAAGCTAGG CGTCGACGCG TTATTAAGCG TTTGGCGTTG GAACAGGAAC 1236
DWV-B Netherlands 2001 VDV-1 (NC_006494) TGAAGCTAGG CGTCGACGCG TTATCAAGCG TTTGGCGTTG GAACAGGAAC 1235
DWV-B France 2019 VV144I (MN565037) TGAAGCTAGG CGTCGACGCG TTATTAAGCG TTTGGCGTTG GAACAGGAAC 1236
DWV-B Slovenia 2019 341-1 (ON648741) TGAAGCTAGG CGTCGACGCG TTATTAAGCG TTTGGCGTTG GAACAGGAAC 1236
DWV-B Austria 2018 B11B (OL803829) TGAAGCTAGG CGTCGACGCG TTATTAAGCG TTTGGCGTTG GAACAGGAAC 1236
DWV-A Austria 2014 1414 (KU847397) TGAAGCTAGG CGGCGCCGAG TTATTAAACG TTTGGCGCTG GAGCAAGAAC 1251
DWV-A USA 2003 PA (AY292384) TGAAGCTAGG CGGCGCCGAG TCATTAAACG TTTGGCGCTG GAGCAAGAAC 1249
DWV-A Japan 2001 Kakugo (AB070959) TGAAGCTAGG CGGCGCCGAG TTATTAAACG TTTGGCGCTG GAGCAAGAAC 1247
DWV-C UK 2015 Devon (ERS657949) AGAATTACGG CGCAAACGTG TTATTAAACG TTTAGCGTTG GAACAAGAGC 1222
DWV-D Egypt 1977 EBV (MT504363) AGAAGCACGC CGTAGACGTG TTATTAAACG TTTAGCGTTA GAACAAGAAC 1245
Darwin bee virus 3 Australia 2014 NT-12 (MG995697) AGAGCTGAGA CGCCAAGCTA TTATAAATCG TCTAGCGCGT AAACAAGA-- 1211
Consensus TGAAGCTAGG CGTCGACGCG TTATTAAGCG TTTGGCGTTG GAACAGGAAC
100%
Conservation
0%
1.360 1.380 1.400
DWV-B Austria 2022 SB22 (PP418870) GGATTCGAAA CGTTCTTGAT GTCACTGTGT ATGATCATAC AACGTGGGAG 1286
DWV-B USA 2018 EVR24 (OR361560) GGATTCGAAA CGTTCTTGAC GTCACTGTGT ATGATCATAC AACGTGGGAG 1286
DWV-B USA 2018 EVR23 (OR361559) GGATTCGAAA CGTTCTTGAC GTCACTGTGT ATGATCATAC AACGTGGGAG 1286
DWV-B Netherlands 2001 VDV-1 (NC_006494) GGATTCGAAA CGTTCTTGAT GTCACTGTGT ATGATCATAC AACGTGGGAG 1285
DWV-B France 2019 VV144I (MN565037) GGATTCGAAA CGTTCTTGAC GTCACTGTGT ATGATCATAC GACGTGGGAG 1286
DWV-B Slovenia 2019 341-1 (ON648741) GGATTCGAAA CGTTCTTGAT GTCACTGTGT ATGATCATAC AACGTGGGAG 1286
DWV-B Austria 2018 B11B (OL803829) GGATTCGAAA CATTCTTGAT GTCACTGTGT ATGATCATAC AACGTGGGAG 1286
DWV-A Austria 2014 1414 (KU847397) GTATTCGTAA CGTTCTTGAC GTTGACGTCT ATAACCAGGC GACATGGGAA 1301
DWV-A USA 2003 PA (AY292384) GTATTCGTAA CGTTCTTGAC GTTGGCGTCT ATGACCAGGC GACATGGGAA 1299
DWV-A Japan 2001 Kakugo (AB070959) GTATTCGTAA TGTTCTTGAC GTTGACGTCT ATAACCAGGC GACATGGGAA 1297
DWV-C UK 2015 Devon (ERS657949) GTATGCGCAA TATTATGGAT AAAGAGGTGT TTAATGACCT TACTTGGGAG 1272
DWV-D Egypt 1977 EBV (MT504363) GTGTGCGTAA TATTATGGAT GTAGAAGTAT TTAATAATCT TACTTGGGAG 1295
Darwin bee virus 3 Australia 2014 NT-12 (MG995697) -GGTTGAGCG CATTATTGAG CAGTTCCCTC TTAGTGATAT GATTTATGA- 1259
Consensus GGATTCGAAA CGTTCTTGAN GTCACTGTGT ATGATCATAC AACGTGGGAG
100%
Conservation
0%
1.420 1.440
DWV-B Austria 2022 SB22 (PP418870) CAAGAAGATG CGCGTGA--- --TAATGAGT TCCTTACGGA ACAATTGAAT 1331
DWV-B USA 2018 EVR24 (OR361560) CAAGAAGATG CGCGTGA--- --TAATGAGT TCCTTACGGA ACAATTGAAT 1331
DWV-B USA 2018 EVR23 (OR361559) CAAGAGGATG CGCGTGA--- --TAATGAGT TCCTTACGGA ACAATTGAAT 1331
DWV-B Netherlands 2001 VDV-1 (NC_006494) CAAGAGGATG CGCGTGA--- --TAATGAGT TCCTTATGGA ACAATTGAAT 1330
DWV-B France 2019 VV144I (MN565037) CAAGAAGATG CGCGTGA--- --TAATGAGT TCCTTACGGA GCAATTGAAT 1331
DWV-B Slovenia 2019 341-1 (ON648741) CAAGAAGATG CGCGTGA--- --TAATGAGT TCCTTACGGA ACAATTGAAT 1331
DWV-B Austria 2018 B11B (OL803829) CAAGAAGATG CGCGTGA--- --TAATGAGT TCCTTACGGA ACAATTGAAT 1331
DWV-A Austria 2014 1414 (KU847397) CAGGAGGACG CGCGTGA--- --TAATGAGT TCCTAACGGA ACAATTAAAT 1346
DWV-A USA 2003 PA (AY292384) CAGGAGGACG CGCGCGA--- --TAATGAGT TCCTAACGGA ACAATTAAAC 1344
DWV-A Japan 2001 Kakugo (AB070959) CAGGAAGACG TGCGCGA--- --TAACGAGT TCCTAACGGA ACAATTAAAC 1342
DWV-C UK 2015 Devon (ERS657949) CAGGAAGGTG TTGAAGA--- --TAATGTAT TTCTATTAGA ACAACTTAAT 1317
DWV-D Egypt 1977 EBV (MT504363) CAGGAGGATG TTAAAGA--- --TAATGAAT TTCTAACGGA ACAACTTAAT 1340
Darwin bee virus 3 Australia 2014 NT-12 (MG995697) -AGATGGACG ATTATGGACT CCTGAGGAAT ATCAGGAGAA ACACGTAAGT 1308
Consensus CAAGAAGATG CGCGTGA--- --TAATGAGT TCCTTACGGA ACAATTGAAT
100%
Conservation
0%
1.460 1.480 1.500
DWV-B Austria 2022 SB22 (PP418870) AATTTATATA CGATATATTC TATAGCTGAA AGATGTAC-C CGCCGCCCTG 1380
DWV-B USA 2018 EVR24 (OR361560) AATTTATATA CGATATATTC TATAGCTGAA AGATGTAC-C CGCCGCCCTG 1380
DWV-B USA 2018 EVR23 (OR361559) AATTTATATA CGATATATTC TATAGCTGAA AGATGTAC-C CGCCGCCCTG 1380
DWV-B Netherlands 2001 VDV-1 (NC_006494) AATTTATATA CGATATATTC TATAGCTGAA AGATGTAC-C CGCCGCCCTG 1379
DWV-B France 2019 VV144I (MN565037) AATTTATATA CGATATATTC TATAGCTGAA AGATGTAC-C CGCCGCCCTG 1380
DWV-B Slovenia 2019 341-1 (ON648741) AATTTATATA CGATATATTC TATAGCTGAA AGATGTAT-C CGCCGCCCTG 1380
DWV-B Austria 2018 B11B (OL803829) AATTTATACA CGATATATTC TATAGCTGAA AGATGTAC-C CGCCGCCCTG 1380
DWV-A Austria 2014 1414 (KU847397) AATTTATATA CTATTTATTC CATCGCTGAA CGTTGTAC-G CGTCGGCCTA 1395
DWV-A USA 2003 PA (AY292384) AATTTATATA CTATTTATTC GATCGCTGAA CGTTGTAC-G CGTCGGCCTA 1393
DWV-A Japan 2001 Kakugo (AB070959) AATTTATATA CTATTTATTC CATCGCTGAA CGTTGTAC-G CGCCGGCCTA 1391
DWV-C UK 2015 Devon (ERS657949) GCATTATATA CTATATATTC CATAGCTGAA CGTTGTGT-T AGGCGGCCAA 1366
DWV-D Egypt 1977 EBV (MT504363) GCATTATATA CTGTATATTC TATAGCTGAG CGTTGTAT-T AGGCGACCAA 1389
Darwin bee virus 3 Australia 2014 NT-12 (MG995697) ATTAAGGCTG TAAAACAGTC TAAACGTAAG TATTATAAAC CAGTATTTAG 1358
Consensus AATTTATATA CGATATATTC TATAGCTGAA AGATGTAC-C CGCCGCCCTG
100%
Conservation
0%

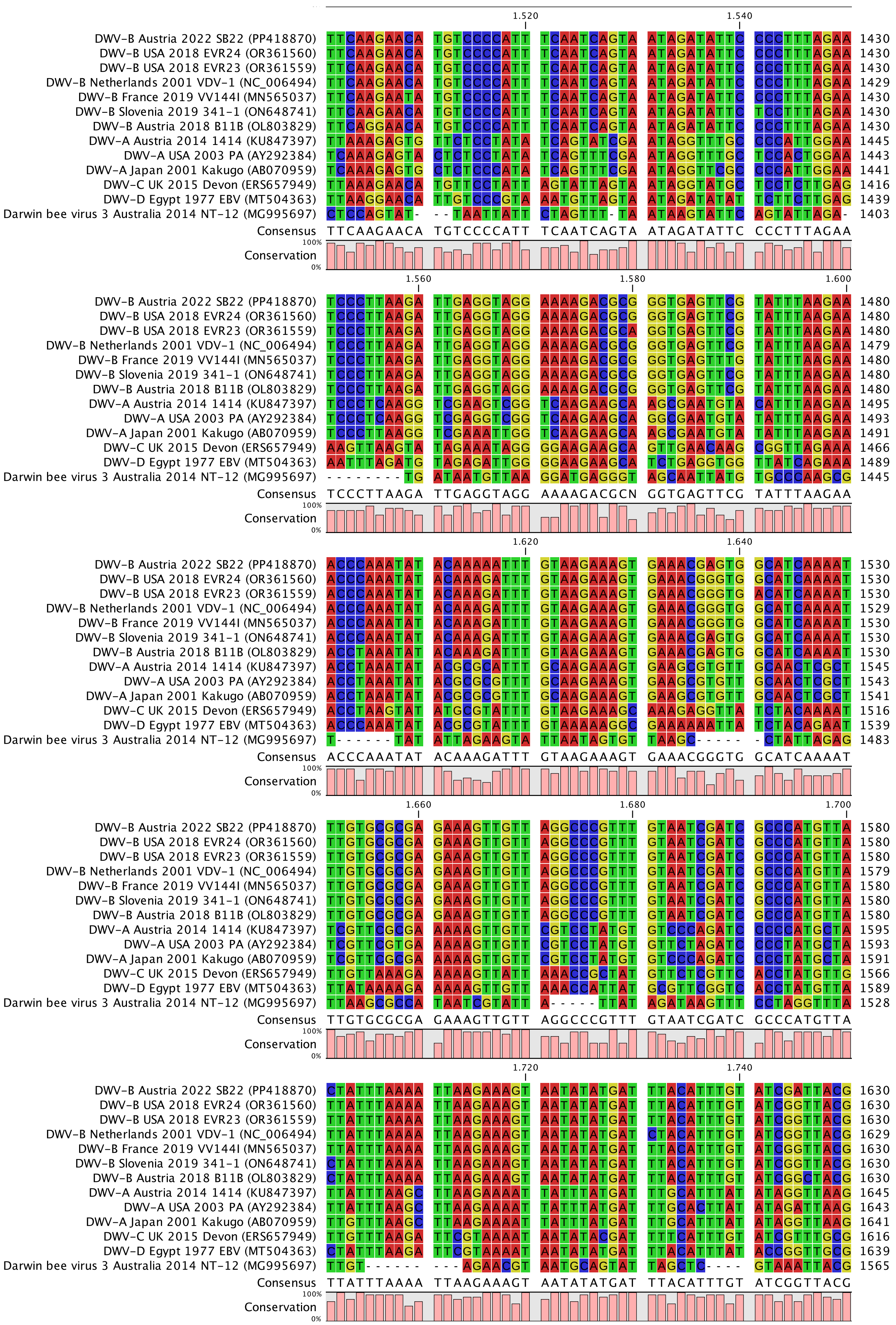
1.520 1.540
DWV-B Austria 2022 SB22 (PP418870) TTCAAGAACA TGTCCCCATT TCAATCAGTA ATAGATATTC CCCTTTAGAA 1430
DWV-B USA 2018 EVR24 (OR361560) TTCAAGAACA TGTCCCCATT TCAATCAGTA ATAGATATTC CCCTTTAGAA 1430
DWV-B USA 2018 EVR23 (OR361559) TTCAAGAACA TGTCCCCATT TCAATCAGTA ATAGATATTC CCCTTTAGAA 1430
DWV-B Netherlands 2001 VDV-1 (NC_006494) TTCAAGAACA TGTCCCCATT TCAATCAGTA ATAGATATTC CCCTTTAGAA 1429
DWV-B France 2019 VV144I (MN565037) TTCAAGAATA TGTCCCCATT TCAATCAGTA ATAGATATTC CCCTTTAGAA 1430
DWV-B Slovenia 2019 341-1 (ON648741) TTCAAGAACA TGTCCCCATT TCAATCAGTA ATAGATATTC TCCTTTAGAA 1430
DWV-B Austria 2018 B11B (OL803829) TTCAGGAACA TGTCCCCATT TCAATCAGTA ATAGATATTC CCCTTTAGAA 1430
DWV-A Austria 2014 1414 (KU847397) TTAAAGAGTG TTCTCCTATA TCAGTATCGA ATAGGTTTGC CCCATTGGAA 1445
DWV-A USA 2003 PA (AY292384) TCAAAGAGTA CTCTCCTATA TCAGTTTCGA ATAGGTTTGC TCCACTGGAA 1443
DWV-A Japan 2001 Kakugo (AB070959) TCAAAGAGTG CTCTCCCATA TCAGTTTCGA ATAGGTTCGC CCCATTGGAA 1441
DWV-C UK 2015 Devon (ERS657949) TTAAAGAACA TGTTCCTATT AGTATTAGTA ATAGGTATGC TCCTCTTGAG 1416
DWV-D Egypt 1977 EBV (MT504363) TTAAGGAACA TTGTCCCGTA AATGTTAGTA ATAGATATAT TCTTCTTGAG 1439
Darwin bee virus 3 Australia 2014 NT-12 (MG995697) CTCCAGTAT- --TAATTATT CTAGTTT-TA ATAAGTATTC AGTATTAGA- 1403
Consensus TTCAAGAACA TGTCCCCATT TCAATCAGTA ATAGATATTC CCCTTTAGAA
100%
Conservation
0%
1.560 1.580 1.600
DWV-B Austria 2022 SB22 (PP418870) TCCCTTAAGA TTGAGGTAGG AAAAGACGCG GGTGAGTTCG TATTTAAGAA 1480
DWV-B USA 2018 EVR24 (OR361560) TCCCTTAAGA TTGAGGTAGG AAAAGACGCG GGTGAGTTCG TATTTAAGAA 1480
DWV-B USA 2018 EVR23 (OR361559) TCCCTTAAGA TTGAGGTAGG AAAAGACGCA GGTGAGTTCG TATTTAAGAA 1480
DWV-B Netherlands 2001 VDV-1 (NC_006494) TCCCTTAAGA TTGAGGTAGG AAAAGACGCG GGTGAGTTCG TATTTAAGAA 1479
DWV-B France 2019 VV144I (MN565037) TCCCTTAAGA TTGAGGTAGG AAAAGACGCG GGTGAGTTTG TATTTAAGAA 1480
DWV-B Slovenia 2019 341-1 (ON648741) TCCCTTAAGA TTGAGGTAGG AAAAGACGCG GGTGAGTTCG TATTTAAGAA 1480
DWV-B Austria 2018 B11B (OL803829) TCCCTTAAGA TTGAGGTAGG AAAAGACGCG GGTGAGTTCG TATTTAAGAA 1480
DWV-A Austria 2014 1414 (KU847397) TCCCTCAAGG TCGAAGTCGG TCAAGAAGCA AGCGAATGTA CATTTAAGAA 1495
DWV-A USA 2003 PA (AY292384) TCCCTCAAGG TCGAGGTCGG TCAAGAAGCA GGCGAATGTA TATTTAAGAA 1493
DWV-A Japan 2001 Kakugo (AB070959) TCCCTTAAGG TCGAAATTGG TCAAGAAGCA AGCGAATGTA TATTTAAGAA 1491
DWV-C UK 2015 Devon (ERS657949) AAGTTAAGTA TAGAAATAGG GGAAGAAGCA GTTGAACAAG CGGTTAGAAA 1466
DWV-D Egypt 1977 EBV (MT504363) AATTTAGATG TAGAGATTGG GGAAGAAGCA TCTGAGGTGG TTATCAGAAA 1489
Darwin bee virus 3 Australia 2014 NT-12 (MG995697) --------TG ATAATGTTAA GGATGAGGGT AGCAATTATG TGCCCAAGCG 1445
Consensus TCCCTTAAGA TTGAGGTAGG AAAAGACGCN GGTGAGTTCG TATTTAAGAA
100%
Conservation
0%
1.620 1.640
DWV-B Austria 2022 SB22 (PP418870) ACCCAAATAT ACAAAAATTT GTAAGAAAGT GAAACGAGTG GCATCAAAAT 1530
DWV-B USA 2018 EVR24 (OR361560) ACCCAAATAT ACAAAGATTT GTAAGAAAGT GAAACGGGTG GCATCAAAAT 1530
DWV-B USA 2018 EVR23 (OR361559) ACCCAAATAT ACAAAGATTT GTAAGAAAGT GAAACGGGTG ACATCAAAAT 1530
DWV-B Netherlands 2001 VDV-1 (NC_006494) ACCCAAATAT ACAAAGATTT GTAAGAAAGT GAAACGGGTG GCATCAAAAT 1529
DWV-B France 2019 VV144I (MN565037) ACCCAAATAT ACAAAGATTT GTAAGAAAGT GAAACGGGTG GCATCAAAAT 1530
DWV-B Slovenia 2019 341-1 (ON648741) ACCCAAATAT ACAAAGATTT GTAAGAAAGT GAAACGAGTG GCATCAAAAT 1530
DWV-B Austria 2018 B11B (OL803829) ACCTAAATAT ACAAAGATTT GTAAGAAAGT GAAACGAGTG GCATCAAAAT 1530
DWV-A Austria 2014 1414 (KU847397) ACCTAAATAT ACGCGCATTT GCAAGAAAGT GAAGCGTGTT GCAACTCGCT 1545
DWV-A USA 2003 PA (AY292384) ACCTAAATAT ACGCGCGTTT GCAAGAAAGT GAAGCGTGTT GCAACTCGCT 1543
DWV-A Japan 2001 Kakugo (AB070959) ACCTAAATAT ACGCGCGTTT GCAAGAAAGT GAAGCGTGTT GCAACTCGCT 1541
DWV-C UK 2015 Devon (ERS657949) ACCTAAGTAT ATGCGTATTT GTAAGAAAGC AAAGAGGTTA TCTACAAAAT 1516
DWV-D Egypt 1977 EBV (MT504363) ACCCAAATAT ACGCGTATTT GTAAAAAGGC GAAAAAATTA TCTACAGAAT 1539
Darwin bee virus 3 Australia 2014 NT-12 (MG995697) T------TAT ATTAGAAGTA TTAATAGTGT TAAGC----- -CTATTAGAG 1483
Consensus ACCCAAATAT ACAAAGATTT GTAAGAAAGT GAAACGGGTG GCATCAAAAT
100%
Conservation
0%
1.660 1.680 1.700
DWV-B Austria 2022 SB22 (PP418870) TTGTGCGCGA GAAAGTTGTT AGGCCCGTTT GTAATCGATC GCCCATGTTA 1580
DWV-B USA 2018 EVR24 (OR361560) TTGTGCGCGA GAAAGTTGTT AGGCCCGTTT GTAATCGATC GCCCATGTTA 1580
DWV-B USA 2018 EVR23 (OR361559) TTGTGCGCGA GAAAGTTGTT AGGCCCGTTT GTAATCGATC GCCCATGTTA 1580
DWV-B Netherlands 2001 VDV-1 (NC_006494) TTGTGCGCGA GAAAGTTGTT AGGCCCGTTT GTAATCGATC GCCCATGTTA 1579
DWV-B France 2019 VV144I (MN565037) TTGTGCGCGA GAAAGTTGTT AGGCCCGTTT GTAATCGATC GCCCATGTTA 1580
DWV-B Slovenia 2019 341-1 (ON648741) TTGTGCGCGA GAAAGTTGTT AGGCCCGTTT GTAATCGATC GCCCATGTTA 1580
DWV-B Austria 2018 B11B (OL803829) TTGTGCGCGA GAAAGTTGTT AGGCCCGTTT GTAATCGATC GCCCATGTTA 1580
DWV-A Austria 2014 1414 (KU847397) TCGTTCGCGA AAAAGTTGTT CGTCCTATGT GTCCCAGATC CCCCATGCTA 1595
DWV-A USA 2003 PA (AY292384) TCGTTCGTGA AAAAGTTGTT CGTCCTATGT GTTCTAGATC CCCTATGCTA 1593
DWV-A Japan 2001 Kakugo (AB070959) TCGTTCGCGA AAAAGTTGTT CGTCCTATGT GTCCCAGATC CCCTATGCTA 1591
DWV-C UK 2015 Devon (ERS657949) TTGTTAAAGA AAAAGTTATT AAACCGCTAT GTTGTCGTTC ACCTATGTTG 1566
DWV-D Egypt 1977 EBV (MT504363) TTATAAAAGA AAAAGTTGTT AAACCATTAT GCGTTCGGTC ACCTATGTTA 1589
Darwin bee virus 3 Australia 2014 NT-12 (MG995697) TTAAGCGCCA TAATCGTATT A-----TTAT AGATAAGTTT CCTAGGTTTA 1528
Consensus TTGTGCGCGA GAAAGTTGTT AGGCCCGTTT GTAATCGATC GCCCATGTTA
100%
Conservation
0%
1.720 1.740
DWV-B Austria 2022 SB22 (PP418870) CTATTTAAAA TTAAGAAAGT AATATATGAT TTACATTTGT ATCGATTACG 1630
DWV-B USA 2018 EVR24 (OR361560) TTATTTAAAA TTAAGAAAGT AATATATGAT TTACATTTGT ATCGGTTACG 1630
DWV-B USA 2018 EVR23 (OR361559) TTATTTAAAA TTAAGAAAGT AATATATGAT TTACATTTGT ATCGGTTACG 1630
DWV-B Netherlands 2001 VDV-1 (NC_006494) TTATTTAAAA TTAAGAAAGT AATATATGAT CTACATTTGT ATCGGTTACG 1629
DWV-B France 2019 VV144I (MN565037) TTATTTAAAA TTAAGAAAGT AATATATGAT TTACATTTGT ATCGGTTACG 1630
DWV-B Slovenia 2019 341-1 (ON648741) CTATTTAAAA TTAAGAAAGT AATATATGAT TTACATTTGT ATCGGTTACG 1630
DWV-B Austria 2018 B11B (OL803829) CTATTTAAAA TTAAGAAAGT AATATATGAT TTACATTTGT ATCGGCTACG 1630
DWV-A Austria 2014 1414 (KU847397) TTATTTAAGC TTAAGAAAAT TATTTATGAT TTGCATTTAT ATAGGTTAAG 1645
DWV-A USA 2003 PA (AY292384) TTATTTAAGC TTAAGAAAAT TATTTATGAT TTGCACTTAT ATAGATTAAG 1643
DWV-A Japan 2001 Kakugo (AB070959) TTGTTTAAGC TTAAGAAAAT TATTTACGAT TTGCATTTAT ATAGGTTAAG 1641
DWV-C UK 2015 Devon (ERS657949) TTGTTTAAGA TTCGTAAAAT AATATACGAT TTTCATTTGT ATCGTTTGCG 1616
DWV-D Egypt 1977 EBV (MT504363) CTATTTAAGA TTCGTAAAAT AATATATGAT TTACATTTAT ACCGGTTGCG 1639
Darwin bee virus 3 Australia 2014 NT-12 (MG995697) TTGT------ ---AGAACGT AATGCAGTAT TAGCTC---- GTAAATTACG 1565
Consensus TTATTTAAAA TTAAGAAAGT AATATATGAT TTACATTTGT ATCGGTTACG
100%
Conservation
0%

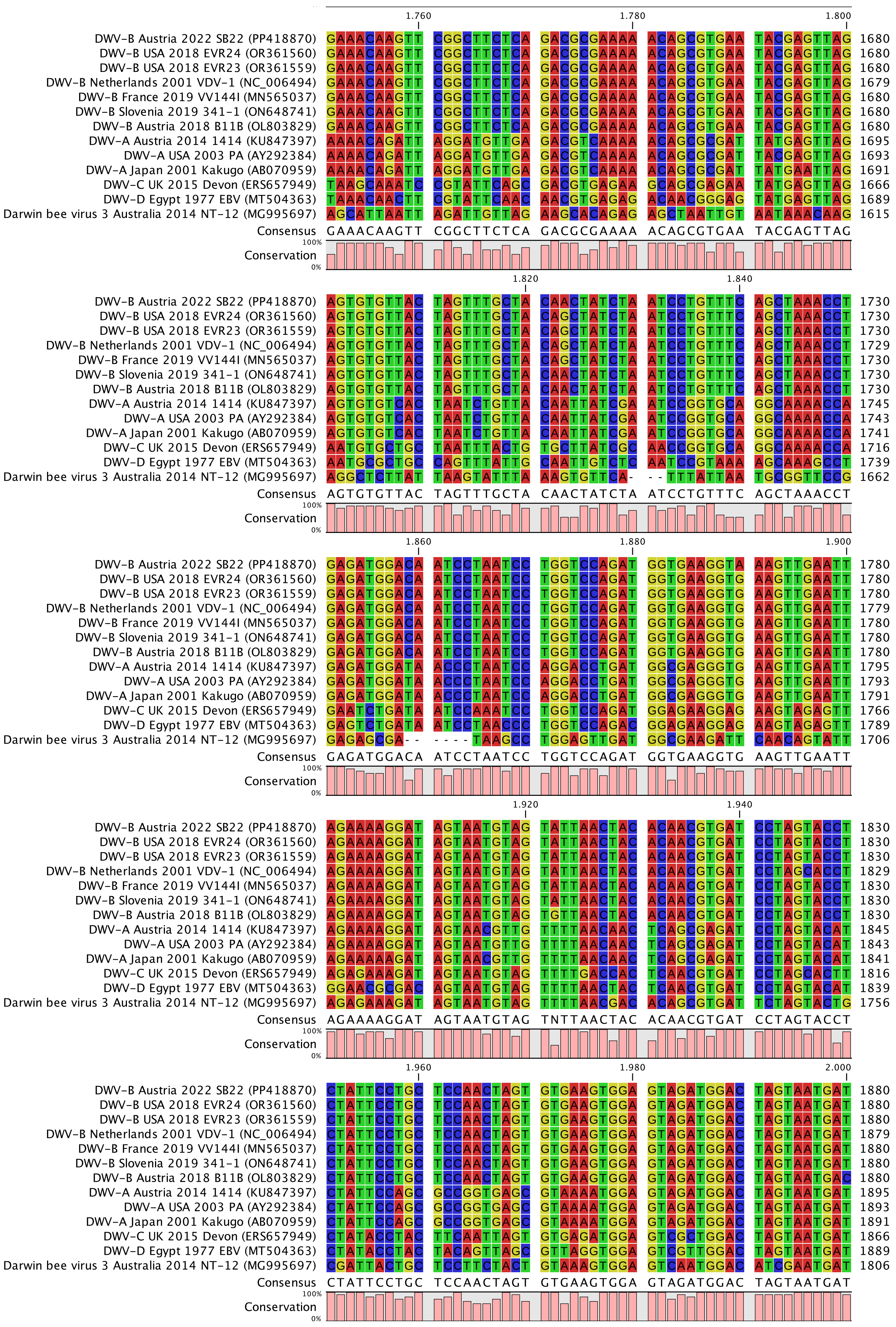
1.760 1.780 1.800
DWV-B Austria 2022 SB22 (PP418870) GAAACAAGTT CGGCTTCTCA GACGCGAAAA ACAGCGTGAA TACGAGTTAG 1680
DWV-B USA 2018 EVR24 (OR361560) GAAACAAGTT CGGCTTCTCA GACGCGAAAA ACAGCGTGAA TACGAGTTAG 1680
DWV-B USA 2018 EVR23 (OR361559) GAAACAAGTT CGGCTTCTCA GACGCGAAAA ACAGCGTGAA TACGAGTTAG 1680
DWV-B Netherlands 2001 VDV-1 (NC_006494) GAAACAAGTT CGGCTTCTCA GACGCGAAAA ACAGCGTGAA TACGAGTTAG 1679
DWV-B France 2019 VV144I (MN565037) GAAACAAGTT CGGCTTCTCA GACGCGAAAA ACAGCGTGAA TACGAGTTAG 1680
DWV-B Slovenia 2019 341-1 (ON648741) GAAACAAGTT CGGCTTCTCA GACGCGAAAA ACAGCGTGAA TACGAGTTAG 1680
DWV-B Austria 2018 B11B (OL803829) GAAACAAGTT CGGCTTCTCA GACGCGAAAA ACAGCGTGAA TACGAGTTAG 1680
DWV-A Austria 2014 1414 (KU847397) AAAACAGATT AGGATGTTGA GACGTCAAAA ACAGCGCGAT TATGAGTTAG 1695
DWV-A USA 2003 PA (AY292384) AAAACAGATT AGGATGTTGA GACGTCAAAA ACAGCGCGAT TACGAGTTAG 1693
DWV-A Japan 2001 Kakugo (AB070959) AAAACAGATT AGGATGTTGA GACGTCAAAA ACAGCGCGAT TATGAATTAG 1691
DWV-C UK 2015 Devon (ERS657949) TAAGCAAATC CGTATTCAGC GACGTGAGAA GCAGCGAGAA TATGAGTTAG 1666
DWV-D Egypt 1977 EBV (MT504363) TAAACAACTT CGTATTCAAC AACGTGAGAG ACAACGGGAG TATGAGTTAG 1689
Darwin bee virus 3 Australia 2014 NT-12 (MG995697) AGCATTAATT AGATTGTTAG AAGCACAGAG AGCTAATTGT AATAAACAAG 1615
Consensus GAAACAAGTT CGGCTTCTCA GACGCGAAAA ACAGCGTGAA TACGAGTTAG
100% Conservation 0%
1.820 1.840
DWV-B Austria 2022 SB22 (PP418870) AGTGTGTTAC TAGTTTGCTA CAACTATCTA ATCCTGTTTC AGCTAAACCT 1730
DWV-B USA 2018 EVR24 (OR361560) AGTGTGTTAC TAGTTTGCTA CAGCTATCTA ATCCTGTTTC AGCTAAACCT 1730
DWV-B USA 2018 EVR23 (OR361559) AGTGTGTTAC TAGTTTGCTA CAGCTATCTA ATCCTGTTTC AGCTAAACCT 1730
DWV-B Netherlands 2001 VDV-1 (NC_006494) AGTGTGTTAC TAGTTTGCTA CAGCTATCTA ATCCTGTTTC AGCTAAACCT 1729
DWV-B France 2019 VV144I (MN565037) AGTGTGTTAC TAGTTTGCTA CAGCTATCTA ATCCTGTTTC AGCTAAACCT 1730
DWV-B Slovenia 2019 341-1 (ON648741) AGTGTGTTAC TAGTTTGCTA CAACTATCTA ATCCTGTTTC AGCTAAACCT 1730
DWV-B Austria 2018 B11B (OL803829) AGTGTGTTAC TAGTTTGCTA CAACTATCTA ATCCTGTTTC AGCTAAACCT 1730
DWV-A Austria 2014 1414 (KU847397) AGTGTGTCAC TAATCTGTTA CAATTATCGA ATCCGGTGCA GGCAAAACCA 1745
DWV-A USA 2003 PA (AY292384) AGTGTGTCAC TAATCTGTTA CAATTATCGA ATCCGGTGCA GGCAAAACCA 1743
DWV-A Japan 2001 Kakugo (AB070959) AGTGTGTCAC TAATCTGTTA CAATTATCGA ATCCGGTGCA GGCAAAACCA 1741
DWV-C UK 2015 Devon (ERS657949) AATGTGCTGC TAATTTACTG TGCTTATCGC AACCGGTGCA GGCAAAACCA 1716
DWV-D Egypt 1977 EBV (MT504363) AATGCGCTGC CAGTTTATTG CAATTGTCTC AATCCGTAAA AGCAAAGCCT 1739
Darwin bee virus 3 Australia 2014 NT-12 (MG995697) AGGCTCTTAT TAAGTATTTA AAGTGTTCA- --TTTATTAA TGCGGTTCCG 1662
Consensus AGTGTGTTAC TAGTTTGCTA CAACTATCTA ATCCTGTTTC AGCTAAACCT
100% Conservation 0%
1.860 1.880 1.900
DWV-B Austria 2022 SB22 (PP418870) GAGATGGACA ATCCTAATCC TGGTCCAGAT GGTGAAGGTA AAGTTGAATT 1780
DWV-B USA 2018 EVR24 (OR361560) GAGATGGACA ATCCTAATCC TGGTCCAGAT GGTGAAGGTG AAGTTGAATT 1780
DWV-B USA 2018 EVR23 (OR361559) GAGATGGACA ATCCTAATCC TGGTCCAGAT GGTGAAGGTG AAGTTGAATT 1780
DWV-B Netherlands 2001 VDV-1 (NC_006494) GAGATGGACA ATCCTAATCC TGGTCCAGAT GGTGAAGGTG AAGTTGAATT 1779
DWV-B France 2019 VV144I (MN565037) GAGATGGACA ATCCTAATCC TGGTCCAGAT GGTGAAGGTG AAGTTGAATT 1780
DWV-B Slovenia 2019 341-1 (ON648741) GAGATGGACA ATCCTAATCC TGGTCCAGAT GGTGAAGGTG AAGTTGAATT 1780
DWV-B Austria 2018 B11B (OL803829) GAGATGGACA ATCCTAATCC TGGTCCAGAT GGTGAAGGTG AAGTTGAATT 1780
DWV-A Austria 2014 1414 (KU847397) GAGATGGATA ACCCTAATCC AGGACCTGAT GGCGAGGGTG AAGTTGAATT 1795
DWV-A USA 2003 PA (AY292384) GAGATGGATA ACCCTAATCC AGGACCTGAT GGCGAGGGTG AAGTTGAATT 1793
DWV-A Japan 2001 Kakugo (AB070959) GAGATGGATA ACCCTAATCC AGGACCTGAT GGCGAGGGTG AAGTTGAATT 1791
DWV-C UK 2015 Devon (ERS657949) GAATCTGATA ATCCAAATCC TGGTCCAGAT GGAGAAGGAG AAGTAGAGTT 1766
DWV-D Egypt 1977 EBV (MT504363) GAGTCTGATA ATCCTAACCC TGGTCCAGAC GGAGAAGGAG AAGTAGAGTT 1789
Darwin bee virus 3 Australia 2014 NT-12 (MG995697) GAGAGCGA-- ----TAAGCC TGGAGTTGAT GGCGAAGATT CAACAGTATT 1706
Consensus GAGATGGACA ATCCTAATCC TGGTCCAGAT GGTGAAGGTG AAGTTGAATT
100% Conservation 0%
1.920 1.940
DWV-B Austria 2022 SB22 (PP418870) AGAAAAGGAT AGTAATGTAG TATTAACTAC ACAACGTGAT CCTAGTACCT 1830
DWV-B USA 2018 EVR24 (OR361560) AGAAAAGGAT AGTAATGTAG TATTAACTAC ACAACGTGAT CCTAGTACCT 1830
DWV-B USA 2018 EVR23 (OR361559) AGAAAAGGAT AGTAATGTAG TATTAACTAC ACAACGTGAT CCTAGTACCT 1830
DWV-B Netherlands 2001 VDV-1 (NC_006494) AGAAAAGGAT AGTAATGTAG TATTAACTAC ACAACGTGAT CCTAGCACCT 1829
DWV-B France 2019 VV144I (MN565037) AGAAAAGGAT AGTAATGTAG TATTAACTAC ACAACGTGAT CCTAGTACCT 1830
DWV-B Slovenia 2019 341-1 (ON648741) AGAAAAGGAT AGTAATGTAG TATTAACTAC ACAACGTGAT CCTAGTACCT 1830
DWV-B Austria 2018 B11B (OL803829) AGAAAAGGAT AGTAATGTAG TGTTAACTAC ACAACGTGAT CCTAGTACCT 1830
DWV-A Austria 2014 1414 (KU847397) AGAAAAGGAT AGTAACGTTG TTTTAACAAC TCAGCGAGAT CCTAGTACAT 1845
DWV-A USA 2003 PA (AY292384) AGAAAAGGAT AGTAATGTTG TTTTAACAAC TCAGCGAGAT CCTAGTACAT 1843
DWV-A Japan 2001 Kakugo (AB070959) AGAAAAGGAT AGTAACGTTG TTTTAACAAC TCAGCGAGAT CCTAGTACAT 1841
DWV-C UK 2015 Devon (ERS657949) AGAGAAAGAT AGTAATGTAG TTTTGACCAC TCAACGTGAT CCTAGCACTT 1816
DWV-D Egypt 1977 EBV (MT504363) GGAACGCGAC AGTAATGTAG TTTTAACTAC TCAACGTGAT CCTAGTACAT 1839
Darwin bee virus 3 Australia 2014 NT-12 (MG995697) AGAGAAAGAT AGTAATGTAG TTTTAACGAC ACAGCGTGAT TCTAGTACTG 1756
Consensus AGAAAAGGAT AGTAATGTAG TNTTAACTAC ACAACGTGAT CCTAGTACCT
100% Conservation 0%
1.960 1.980 2.000
DWV-B Austria 2022 SB22 (PP418870) CTATTCCTGC TCCAACTAGT GTGAAGTGGA GTAGATGGAC TAGTAATGAT 1880
DWV-B USA 2018 EVR24 (OR361560) CTATTCCTGC TCCAACTAGT GTGAAGTGGA GTAGATGGAC TAGTAATGAT 1880
DWV-B USA 2018 EVR23 (OR361559) CTATTCCTGC TCCAACTAGT GTGAAGTGGA GTAGATGGAC TAGTAATGAT 1880
DWV-B Netherlands 2001 VDV-1 (NC_006494) CTATTCCTGC TCCAACTAGT GTGAAGTGGA GTAGATGGAC TAGTAATGAT 1879
DWV-B France 2019 VV144I (MN565037) CTATTCCTGC TCCAACTAGT GTGAAGTGGA GTAGATGGAC TAGTAATGAT 1880
DWV-B Slovenia 2019 341-1 (ON648741) CTATTCCTGC TCCAACTAGT GTGAAGTGGA GTAGATGGAC TAGTAATGAT 1880
DWV-B Austria 2018 B11B (OL803829) CTATTCCTGC TCCAACTAGT GTGAAGTGGA GTAGATGGAC TAGTAATGAC 1880
DWV-A Austria 2014 1414 (KU847397) CTATTCCAGC GCCGGTGAGC GTAAAATGGA GTAGATGGAC TAGTAATGAT 1895
DWV-A USA 2003 PA (AY292384) CTATTCCAGC GCCGGTGAGC GTAAAATGGA GTAGATGGAC TAGTAATGAT 1893
DWV-A Japan 2001 Kakugo (AB070959) CTATTCCAGC GCCGGTGAGC GTAAAATGGA GTAGATGGAC TAGTAATGAT 1891
DWV-C UK 2015 Devon (ERS657949) CTATACCTAC TTCAATTAGT GTGAGATGGA GTCGCTGGAC TAGTAATGAT 1866
DWV-D Egypt 1977 EBV (MT504363) CTATACCTAC TACAGTTAGC GTTAGGTGGA GTCGTTGGAC TAGTAATGAT 1889
Darwin bee virus 3 Australia 2014 NT-12 (MG995697) CGATTACTGC TCCTTCTACT GTAAAGTGGA GTCAATGGAC ATCGAATGAT 1806
Consensus CTATTCCTGC TCCAACTAGT GTGAAGTGGA GTAGATGGAC TAGTAATGAT
100% Conservation 0%

		2.020		2.040		
DWV-B Austria 2022 SB22 (PP418870)	GTTGTGGATG	ATTATGCCAC	TATAACTTCG	CGTTGGTATC	AAATTGCCGA	1930
DWV-B USA 2018 EVR24 (OR361560)	GTTGTGGATG	ATTATGCCAC	TATAACTTCG	CGTTGGTATC	AAATTGCCGA	1930
DWV-B USA 2018 EVR23 (OR361559)	GTTGTGGATG	ATTATGCCAC	TATAACTTCG	CGTTGGTATC	AAATTGCCGA	1930
DWV-B Netherlands 2001 VDV-1 (NC_006494)	GTTGTGGATG	ATTATGCCAC	TATAACTTCG	CGTTGGTATC	AGATTGCCGA	1929
DWV-B France 2019 VV144I (MN565037)	GTTGTGGATG	ATTATGCCAC	TATAACTTCG	CGTTGGTATC	AAATTGCCGA	1930
DWV-B Slovenia 2019 341-1 (ON648741)	GTTGTGGATG	ATTATGCCAC	TATAACTTCG	CGTTGGTATC	AAATTGCCGA	1930
DWV-B Austria 2018 B11B (OL803829)	GTTGTGGATG	ATTATGCCAC	TATAACTTCG	CGTTGGTATC	AAATTGCCGA	1930
DWV-A Austria 2014 1414 (KU847397)	GTAGTAGATG	ATTACGCCAC	AATTACTTCT	CGATGGTATC	AGATTGCTGA	1945
DWV-A USA 2003 PA (AY292384)	GTAGTAGATG	ATTACGCCAC	AATCACATCT	CGATGGTATC	AGATTGCTGA	1943
DWV-A Japan 2001 Kakugo (AB070959)	GTAGTAGATG	ATTACGCCAC	AATCACATCT	CGATGGTATC	AGATTGCTGA	1941
DWV-C UK 2015 Devon (ERS657949)	GTGGTAGATG	ATTATGCTAC	TATTACTTCT	CGTTGGTATC	AAATTACAGA	1916
DWV-D Egypt 1977 EBV (MT504363)	GTAGTAGATG	ATTATGCTAC	TATTACATCG	CGCTGGTATC	AGATTGCAGA	1939
Darwin bee virus 3 Australia 2014 NT-12 (MG995697)	ATAGTGGATA	GTTATGCGTC	GATAACGGAT	CGTTGGTATC	AAATTAATGA	1856
Consensus	GTTGTGGATG	ATTATGCCAC	TATAACTTCG	CGTTGGTATC	AAATTGCCGA	
Conservation						

	2.060		2.080		2.100	
DWV-B Austria 2022 SB22 (PP418870)	ATTTGTATGG	TCAAAGGATG	ATCCATTTGA	TAAGGAATTG	GCGCGCTTAA	1980
DWV-B USA 2018 EVR24 (OR361560)	ATTTGTATGG	TCAAAGGATG	ATCCATTTGA	TAAGGAATTG	GCGCGCTTAA	1980
DWV-B USA 2018 EVR23 (OR361559)	ATTTGTATGG	TCAAAGGATG	ATCCATTTGA	TAAGGAATTG	GCGCGCTTAA	1980
DWV-B Netherlands 2001 VDV-1 (NC_006494)	ATTTGTGTGG	TCAAAGGATG	ATCCATTTGA	TAAGGAATTG	GCGCGCTTAA	1979
DWV-B France 2019 VV144I (MN565037)	ATTTGTATGG	TCAAAGGATG	ATCCATTTGA	TAAGGAATTG	GCGCGCTTAA	1980
DWV-B Slovenia 2019 341-1 (ON648741)	ATTTGTATGG	TCAAAGGATG	ATCCATTTGA	TAAGGAATTG	GCGCGCTTAA	1980
DWV-B Austria 2018 B11B (OL803829)	ATTTGTATGG	TCAAAGGATG	ATCCATTTGA	TAAGGAATTG	GCGCGCTTAA	1980
DWV-A Austria 2014 1414 (KU847397)	ATTTGTTTGG	TCGAAGGATG	ATCCATTTGA	TAAGGAGTTA	GCACGCTTAA	1995
DWV-A USA 2003 PA (AY292384)	ATTTGTTTGG	TCGAAGGATG	ATCCATTTGA	TAAGGAGTTA	GCACGTTTAA	1993
DWV-A Japan 2001 Kakugo (AB070959)	ATTTGTTTGG	TCGAAGGATG	ATCCATTTGA	TAAGGAGTTA	GCACGTTTAA	1991
DWV-C UK 2015 Devon (ERS657949)	ATTTGTGTGG	TCGAAAGATG	ATCCATTTGA	TAAAGAATTA	ACTAGACTTA	1966
DWV-D Egypt 1977 EBV (MT504363)	ATTTGTTTGG	TCGAAAGATG	ATCCCTTCGA	TAAAGAATTA	ACTAGACTAA	1989
Darwin bee virus 3 Australia 2014 NT-12 (MG995697)	AGTGAAGTGG	ACGAAAGATC	ATAAATTTGA	TACTGAATTA	TACAGATTAG	1906
Consensus	ATTTGTATGG	TCAAAGGATG	ATCCATTTGA	TAAGGAATTG	GCGCGCTTAA	
Conservation						

		2.120		2.140		
DWV-B Austria 2022 SB22 (PP418870)	TTTTACCTCG	AGCTTTGTTA	TCTAGTATTG	AGGCTAATTC	TGACGCTATT	2030
DWV-B USA 2018 EVR24 (OR361560)	TTTTACCTCG	AGCTTTGTTA	TCTAGTATTG	AGGCTAATTC	TGACGCTATT	2030
DWV-B USA 2018 EVR23 (OR361559)	TTTTACCTCG	AGCTTTGTTA	TCTAGTATTG	AGGCTAATTC	TGACGCTATT	2030
DWV-B Netherlands 2001 VDV-1 (NC_006494)	TTTTACCTCG	AGCTTTGTTA	TCTAGTATTG	AGGCTAATTC	TGACGCTATT	2029
DWV-B France 2019 VV144I (MN565037)	TTTTACCTCG	AGCTTTGTTA	TCTAGTATTG	AGGCTAATTC	TGACGCTATT	2030
DWV-B Slovenia 2019 341-1 (ON648741)	TTTTACCTCG	AGCTTTGTTA	TCTAGTATTG	AGGCTAATTC	TGACGCTATT	2030
DWV-B Austria 2018 B11B (OL803829)	TTTTACCTCG	AGCTTTATTA	TCTAGTATTG	AGGCTAATTC	TGACGCTATT	2030
DWV-A Austria 2014 1414 (KU847397)	TTTTGCCTCG	TGCTTTGTTA	TCTAGTATAG	AGGCTAATTC	TGATGCTATA	2045
DWV-A USA 2003 PA (AY292384)	TTTTGCCTCG	TGCTTTGTTA	TCTAGTATAG	AGGCTAATTC	TGATGCTATA	2043
DWV-A Japan 2001 Kakugo (AB070959)	TTTTGCCTCG	TGCTTTGTTA	TCTAGTATAG	AGGCTAATTC	TGATGCTATA	2041
DWV-C UK 2015 Devon (ERS657949)	TTTTACCACG	AGCATTGTTG	TCTAGTATTG	AAGCTAATAC	AGATGCAATT	2016
DWV-D Egypt 1977 EBV (MT504363)	TTTTACCGCG	AGCATTATTG	TCCAGTATTG	AATCTAATAC	TGACGCGATT	2039
Darwin bee virus 3 Australia 2014 NT-12 (MG995697)	TGTTGCCTAG	AGAATTATTG	GCATTTATTG	AAAATAATCA	TGATGATATT	1956
Consensus	TTTTACCTCG	AGCTTTGTTA	TCTAGTATTG	AGGCTAATTC	TGACGCTATT	
Conservation						

	2.160		2.180		2.200	
DWV-B Austria 2022 SB22 (PP418870)	TGTGATGTAC	CTAATACTAT	TCCGTTTAAG	GTACATGCAT	ATTGGCGTGG	2080
DWV-B USA 2018 EVR24 (OR361560)	TGTGATGTAC	CTAATACTAT	TCCGTTTAAG	GTACATGCAT	ATTGGCGTGG	2080
DWV-B USA 2018 EVR23 (OR361559)	TGTGATGTAC	CTAATACTAT	TCCGTTTAAG	GTACATGCAT	ATTGGCGTGG	2080
DWV-B Netherlands 2001 VDV-1 (NC_006494)	TGTGATGTAC	CTAATACTAT	TCCGTTTAAG	GTACATGCAT	ATTGGCGTGG	2079
DWV-B France 2019 VV144I (MN565037)	TGTGATGTAC	CTAATACTAT	TCCGTTTAAG	GTACATGCAT	ATTGGCGTGG	2080
DWV-B Slovenia 2019 341-1 (ON648741)	TGTGATGTAC	CTAATACTAT	TCCGTTTAAG	GTACATGCAT	ATTGGCGTGG	2080
DWV-B Austria 2018 B11B (OL803829)	TGTGATGTAC	CTAATACTAT	TCCGTTTAAG	GTACATGCAT	ATTGGCGTGG	2080
DWV-A Austria 2014 1414 (KU847397)	TGTGATGTGC	CTAATACTAT	CCCATTCAAG	GTACACGCAT	ATTGGCGAGG	2095
DWV-A USA 2003 PA (AY292384)	TGTGATGTGC	CTAATACTAT	CCCATTTAAG	GTACACGCAT	ATTGGCGAGG	2093
DWV-A Japan 2001 Kakugo (AB070959)	TGTGATGTGC	CTAATACTAT	TCCATTTAAG	GTACCCGCAT	ATTGGCGAGG	2091
DWV-C UK 2015 Devon (ERS657949)	TGTGATGTTC	CTAATACTAT	ACCTTTCAAA	GTACATGCTT	ATTGGCGGGG	2066
DWV-D Egypt 1977 EBV (MT504363)	TGTGATGTTC	CTAATACTAT	ACCTTTTAAA	GTCCATGCAT	ATTGGAGAGG	2089
Darwin bee virus 3 Australia 2014 NT-12 (MG995697)	GGTAATGTAC	CTAATACAAT	ACCATTTCGT	GTTCATGCTT	ATTGGAGAGG	2006
Consensus	TGTGATGTAC	CTAATACTAT	TCCGTTTAAG	GTACATGCAT	ATTGGCGTGG	
Conservation						

		2.220		2.240		
DWV-B Austria 2022 SB22 (PP418870)	AGATATGGAA	GTTCGAGTGC	AGATTAACTC	GAATAAATTC	CAGGTTGGTC	2130
DWV-B USA 2018 EVR24 (OR361560)	AGATATGGAA	GTTCGAGTGC	AGATTAACTC	GAATAAATTC	CAGGTTGGTC	2130
DWV-B USA 2018 EVR23 (OR361559)	AGATATGGAA	GTTCGAGTGC	AGATTAACTC	GAATAAATTC	CAGGTTGGTC	2130
DWV-B Netherlands 2001 VDV-1 (NC_006494)	AGATATGGAA	GTTCGAGTGC	AGATTAACTC	GAATAAATTC	CAGGTTGGTC	2129
DWV-B France 2019 VV144I (MN565037)	AGATATGGAA	GTTCGAGTGC	AGATTAACTC	GAATAAATTC	CAGGTTGGTC	2130
DWV-B Slovenia 2019 341-1 (ON648741)	AGATATGGAA	GTTCGAGTGC	AGATTAACTC	GAATAAATTC	CAGGTTGGTC	2130
DWV-B Austria 2018 B11B (OL803829)	AGATATGGAA	GTTCGAGTGC	AGATTAACTC	GAATAAATTC	CAGGTTGGTC	2130
DWV-A Austria 2014 1414 (KU847397)	CGATATGGAA	GTTAGAGTTC	AGATTAATTC	AAATAAATTC	CAAGTTGGTC	2145
DWV-A USA 2003 PA (AY292384)	CGATATGGAA	GTTAGAGTTC	AAATTAATTC	AAATAAATTC	CAAGTTGGTC	2143
DWV-A Japan 2001 Kakugo (AB070959)	CGATATGGAA	GTTAGAGTTC	AAATTAGTTC	TAATAAATTC	CAAGTTGGTC	2141
DWV-C UK 2015 Devon (ERS657949)	AGATATGGAA	GTCCGTGTTC	AAATTAATTC	TAATAAGTTC	CAAGTTGGTC	2116
DWV-D Egypt 1977 EBV (MT504363)	AGATATGGAG	GTTCGTGTGC	AGATTAACTC	TAACAAATTC	CAAGTTGGCC	2139
Darwin bee virus 3 Australia 2014 NT-12 (MG995697)	TGATATGGAG	ATTAAAATAC	AAATTAACTC	CAACAAATTC	CAAGTCGGCC	2056
Consensus	AGATATGGAA	GTTCGAGTGC	AGATTAACTC	GAATAAATTC	CAGGTTGGTC	
Conservation						

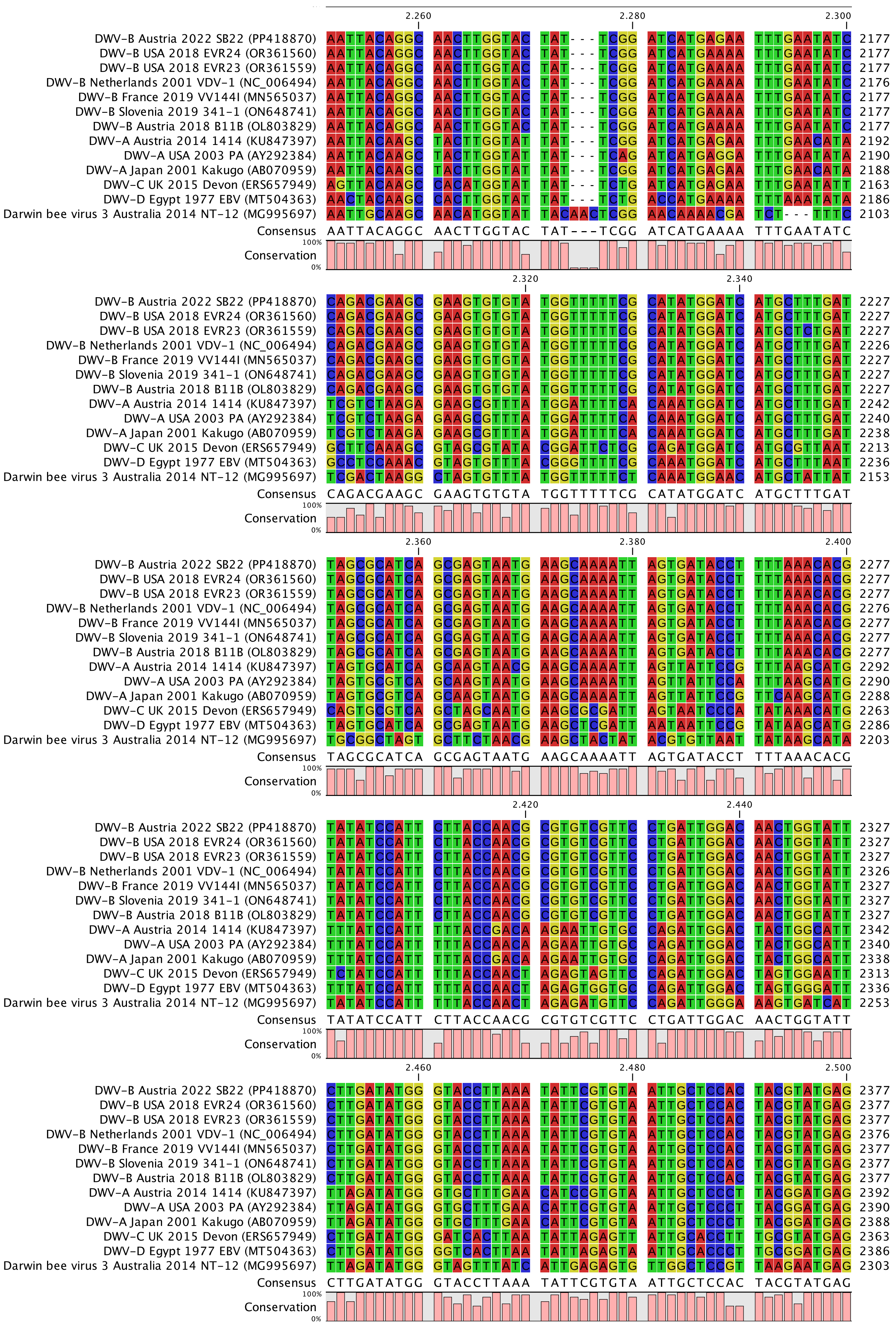

2.260 2.280 2.300
DWV-B Austria 2022 SB22 (PP418870) 2177
DWV-B USA 2018 EVR24 (OR361560) 2177
DWV-B USA 2018 EVR23 (OR361559) 2177
DWV-B Netherlands 2001 VDV-1 (NC_006494) 2176
DWV-B France 2019 VV1441 (MN565037) 2177
DWV-B Slovenia 2019 341-1 (ON648741) 2177
DWV-B Austria 2018 B11B (OL803829) 2177
DWV-A Austria 2014 1414 (KU847397) 2192
DWV-A USA 2003 PA (AY292384) 2190
DWV-A Japan 2001 Kakugo (AB070959) 2188
DWV-C UK 2015 Devon (ERS657949) 2163
DWV-D Egypt 1977 EBV (MT504363) 2186
Darwin bee virus 3 Australia 2014 NT-12 (MG995697) 2103
Consensus AATTACAGGC AACTTGGTAC TAT---TCGG ATCATGAAAA TTTGAATATC
Conservation 100% 0%
2.320 2.340
DWV-B Austria 2022 SB22 (PP418870) 2227
DWV-B USA 2018 EVR24 (OR361560) 2227
DWV-B USA 2018 EVR23 (OR361559) 2227
DWV-B Netherlands 2001 VDV-1 (NC_006494) 2226
DWV-B France 2019 VV1441 (MN565037) 2227
DWV-B Slovenia 2019 341-1 (ON648741) 2227
DWV-B Austria 2018 B11B (OL803829) 2227
DWV-A Austria 2014 1414 (KU847397) 2242
DWV-A USA 2003 PA (AY292384) 2240
DWV-A Japan 2001 Kakugo (AB070959) 2238
DWV-C UK 2015 Devon (ERS657949) 2213
DWV-D Egypt 1977 EBV (MT504363) 2236
Darwin bee virus 3 Australia 2014 NT-12 (MG995697) 2153
Consensus CAGACGAAGC GAAGTGTGTA TGGTTTTTCG CATATGGATC ATGCTTTGAT
Conservation 100% 0%
2.360 2.380 2.400
DWV-B Austria 2022 SB22 (PP418870) 2277
DWV-B USA 2018 EVR24 (OR361560) 2277
DWV-B USA 2018 EVR23 (OR361559) 2277
DWV-B Netherlands 2001 VDV-1 (NC_006494) 2276
DWV-B France 2019 VV1441 (MN565037) 2277
DWV-B Slovenia 2019 341-1 (ON648741) 2277
DWV-B Austria 2018 B11B (OL803829) 2277
DWV-A Austria 2014 1414 (KU847397) 2292
DWV-A USA 2003 PA (AY292384) 2290
DWV-A Japan 2001 Kakugo (AB070959) 2288
DWV-C UK 2015 Devon (ERS657949) 2263
DWV-D Egypt 1977 EBV (MT504363) 2286
Darwin bee virus 3 Australia 2014 NT-12 (MG995697) 2203
Consensus TAGCGCATCA GCGAGTAATG AAGCAAAATT AGTGATACCT TTTAAACACG
Conservation 100% 0%
2.420 2.440
DWV-B Austria 2022 SB22 (PP418870) 2327
DWV-B USA 2018 EVR24 (OR361560) 2327
DWV-B USA 2018 EVR23 (OR361559) 2327
DWV-B Netherlands 2001 VDV-1 (NC_006494) 2326
DWV-B France 2019 VV1441 (MN565037) 2327
DWV-B Slovenia 2019 341-1 (ON648741) 2327
DWV-B Austria 2018 B11B (OL803829) 2327
DWV-A Austria 2014 1414 (KU847397) 2342
DWV-A USA 2003 PA (AY292384) 2340
DWV-A Japan 2001 Kakugo (AB070959) 2338
DWV-C UK 2015 Devon (ERS657949) 2313
DWV-D Egypt 1977 EBV (MT504363) 2336
Darwin bee virus 3 Australia 2014 NT-12 (MG995697) 2253
Consensus TATATCCATT CTTACCAACG CGTGTCGTTC CTGATTGGAC AACTGGTATT
Conservation 100% 0%
2.460 2.480 2.500
DWV-B Austria 2022 SB22 (PP418870) 2377
DWV-B USA 2018 EVR24 (OR361560) 2377
DWV-B USA 2018 EVR23 (OR361559) 2377
DWV-B Netherlands 2001 VDV-1 (NC_006494) 2376
DWV-B France 2019 VV1441 (MN565037) 2377
DWV-B Slovenia 2019 341-1 (ON648741) 2377
DWV-B Austria 2018 B11B (OL803829) 2377
DWV-A Austria 2014 1414 (KU847397) 2392
DWV-A USA 2003 PA (AY292384) 2390
DWV-A Japan 2001 Kakugo (AB070959) 2388
DWV-C UK 2015 Devon (ERS657949) 2363
DWV-D Egypt 1977 EBV (MT504363) 2386
Darwin bee virus 3 Australia 2014 NT-12 (MG995697) 2303
Consensus CTTGATATGG GTACCTTAAA TATTCGTGTA ATTGCTCCAC TACGTATGAG
Conservation 100% 0%

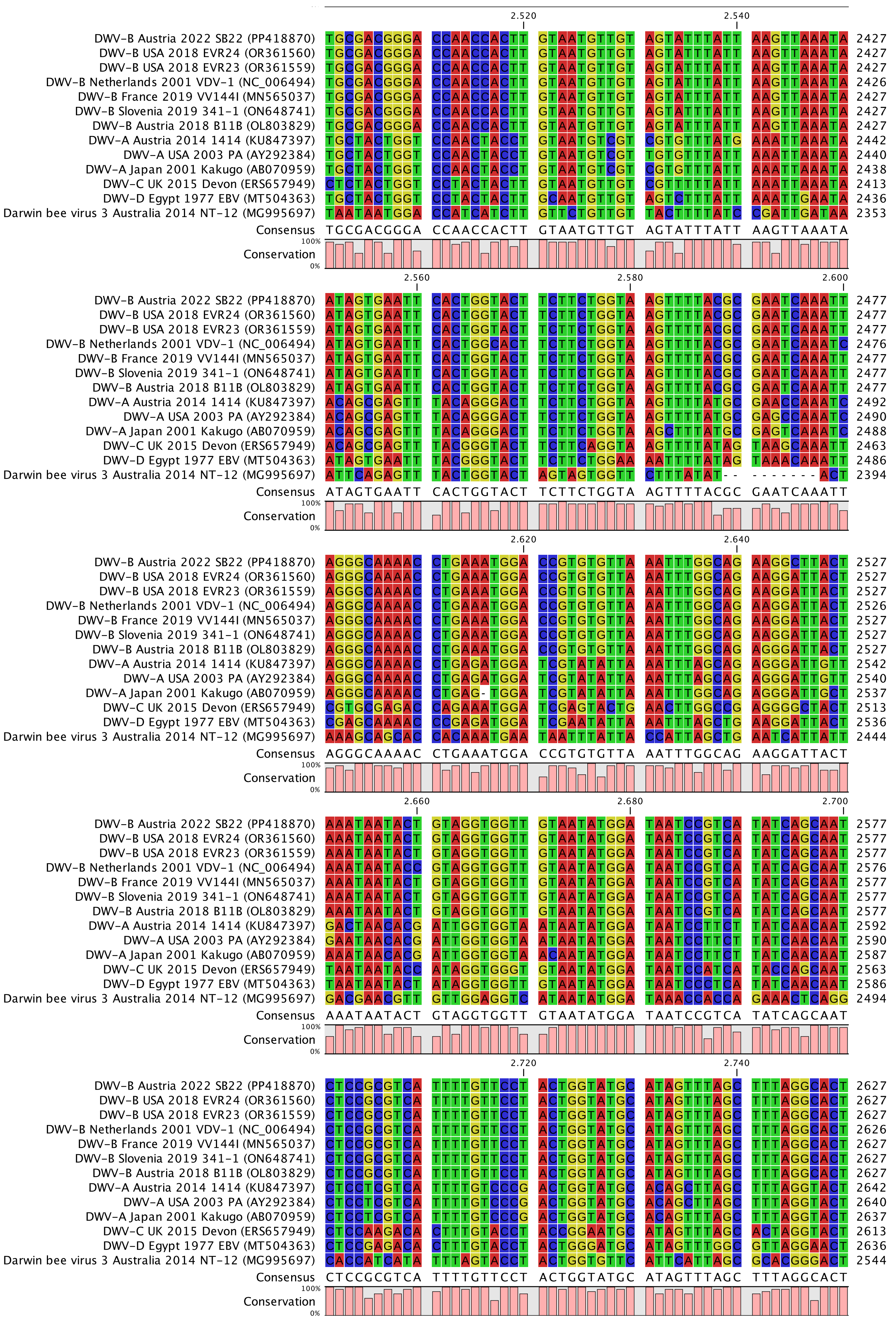
2.520
2.540
DWV-B Austria 2022 SB22 (PP418870) TGCGACGGGA CCAACCACTT GTAATGTTGT AGTATTTATT AAGTTAAATA 2427
DWV-B USA 2018 EVR24 (OR361560) TGCGACGGGA CCAACCACTT GTAATGTTGT AGTATTTATT AAGTTAAATA 2427
DWV-B USA 2018 EVR23 (OR361559) TGCGACGGGA CCAACCACTT GTAATGTTGT AGTATTTATT AAGTTAAATA 2427
DWV-B Netherlands 2001 VDV-1 (NC_006494) TGCGACGGGA CCAACCACTT GTAATGTTGT AGTATTTATT AAGTTAAATA 2426
DWV-B France 2019 VV144I (MN565037) TGCGACGGGA CCAACCACTT GTAATGTTGT AGTATTTATT AAGTTAAATA 2427
DWV-B Slovenia 2019 341-1 (ON648741) TGCGACGGGA CCAACCACTT GTAATGTTGT AGTATTTATT AAGTTAAATA 2427
DWV-B Austria 2018 B11B (OL803829) TGCGACGGGA CCAACCACTT GTAATGTTGT AGTATTTATT AAGTTAAATA 2427
DWV-A Austria 2014 1414 (KU847397) TGCTACTGGT CCAACTACCT GTAATGTCGT CGTGTTTATG AAATTAAATA 2442
DWV-A USA 2003 PA (AY292384) TGCTACTGGT CCAACTACCT GTAATGTCGT TGTGTTTATT AAATTAAATA 2440
DWV-A Japan 2001 Kakugo (AB070959) TGCTACTGGT CCAACTACCT GTAATGTCGT CGTGTTTATT AAATTAAATA 2438
DWV-C UK 2015 Devon (ERS657949) CTCTACTGGT CCTACTACTT GTAATGTTGT CGTTTTTATT AAATTAAATA 2413
DWV-D Egypt 1977 EBV (MT504363) TGCTACTGGT CCTACTACTT GCAATGTTGT AGTCTTTATT AAATTGAATA 2436
Darwin bee virus 3 Australia 2014 NT-12 (MG995697) TAATAATGGA CCATCATCTT GTTCTGTTGT TACTTTTATC CGATTGATAA 2353
Consensus TGCGACGGGA CCAACCACTT GTAATGTTGT AGTATTTATT AAGTTAAATA
100%
Conservation
0%
2.560
2.580
2.600
DWV-B Austria 2022 SB22 (PP418870) ATAGTGAATT CACTGGTACT TCTTCTGGTA AGTTTTACGC GAATCAAATT 2477
DWV-B USA 2018 EVR24 (OR361560) ATAGTGAATT CACTGGTACT TCTTCTGGTA AGTTTTACGC GAATCAAATT 2477
DWV-B USA 2018 EVR23 (OR361559) ATAGTGAATT CACTGGTACT TCTTCTGGTA AGTTTTACGC GAATCAAATT 2477
DWV-B Netherlands 2001 VDV-1 (NC_006494) ATAGTGAATT CACTGGCACT TCTTCTGGTA AGTTTTACGC GAATCAAATC 2476
DWV-B France 2019 VV144I (MN565037) ATAGTGAATT CACTGGTACT TCTTCTGGTA AGTTTTACGC GAATCAAATT 2477
DWV-B Slovenia 2019 341-1 (ON648741) ATAGTGAATT CACTGGTACT TCTTCTGGTA AGTTTTACGC GAATCAAATT 2477
DWV-B Austria 2018 B11B (OL803829) ATAGTGAATT CACTGGTACT TCTTCTGGTA AGTTTTACGC GAATCAAATT 2477
DWV-A Austria 2014 1414 (KU847397) ACAGCGAGTT TACAGGGACT TCTTCTGGTA AGTTTTATGC GAACCAAATC 2492
DWV-A USA 2003 PA (AY292384) ACAGCGAGTT TACAGGGACT TCTTCTGGTA AGTTTTATGC GAGCCAAATC 2490
DWV-A Japan 2001 Kakugo (AB070959) ACAGCGAGTT TACAGGGACT TCTTCTGGTA AGCTTTATGC GAGTCAAATC 2488
DWV-C UK 2015 Devon (ERS657949) ACAGCGAGTT TACGGGTACT TCTTCAGGTA AGTTTTATAG TAAGCAAATT 2463
DWV-D Egypt 1977 EBV (MT504363) ATAGTGAATT TACGGGTACT TCTTCTGGAA AATTTTATAG TAAACAAATT 2486
Darwin bee virus 3 Australia 2014 NT-12 (MG995697) ATTCAGAGTT TACTGGTACT AGTAGTGGTT CTTTATAT-- -------ACT 2394
Consensus ATAGTGAATT CACTGGTACT TCTTCTGGTA AGTTTTACGC GAATCAAATT
100%
Conservation
0%
2.620
2.640
DWV-B Austria 2022 SB22 (PP418870) AGGGCAAAAC CTGAAATGGA CCGTGTGTTA AATTTGGCAG AAGGCTTACT 2527
DWV-B USA 2018 EVR24 (OR361560) AGGGCAAAAC CTGAAATGGA CCGTGTGTTA AATTTGGCAG AAGGATTACT 2527
DWV-B USA 2018 EVR23 (OR361559) AGGGCAAAAC CTGAAATGGA CCGTGTGTTA AATTTGGCAG AAGGATTACT 2527
DWV-B Netherlands 2001 VDV-1 (NC_006494) AGGGCAAAAC CTGAAATGGA CCGTGTGTTA AATTTGGCAG AAGGATTACT 2526
DWV-B France 2019 VV144I (MN565037) AGGGCAAAAC CTGAAATGGA CCGTGTGTTA AATTTGGCAG AAGGATTACT 2527
DWV-B Slovenia 2019 341-1 (ON648741) AGGGCAAAAC CTGAAATGGA CCGTGTGTTA AATTTGGCAG AAGGATTACT 2527
DWV-B Austria 2018 B11B (OL803829) AGGGCAAAAC CTGAAATGGA CCGTGTGTTA AATTTGGCAG AGGGATTACT 2527
DWV-A Austria 2014 1414 (KU847397) AGGGCAAAAC CTGAGATGGA TCGTATATTA AATTTAGCAG AGGGATTGTT 2542
DWV-A USA 2003 PA (AY292384) AGGGCAAAAC CTGAGATGGA TCGTATATTA AATTTAGCAG AGGGATTGTT 2540
DWV-A Japan 2001 Kakugo (AB070959) AGGGCAAAAC CTGAG-TGGA TCGTATATTA AATTTGGCAG AGGGATTGCT 2537
DWV-C UK 2015 Devon (ERS657949) CGTGCGAGAC CAGAAATGGA TCGAGTACTG AAGTTGGCGG AGGGGCTACT 2513
DWV-D Egypt 1977 EBV (MT504363) CGAGCAAAAC CCGAGATGGA TCGAATATTA AATTTAGCTG AAGGATTACT 2536
Darwin bee virus 3 Australia 2014 NT-12 (MG995697) AAAGCAGCAC CACAAATGAA TAATTTATTA CCATTAGCTG AATCATTATT 2444
Consensus AGGGCAAAAC CTGAAATGGA CCGTGTGTTA AATTTGGCAG AAGGATTACT
100%
Conservation
0%
2.660
2.680
2.700
DWV-B Austria 2022 SB22 (PP418870) AAATAATACT GTAGGTGGTT GTAATATGGA TAATCCGTCA TATCAGCAAT 2577
DWV-B USA 2018 EVR24 (OR361560) AAATAATACT GTAGGTGGTT GTAATATGGA TAATCCGTCA TATCAGCAAT 2577
DWV-B USA 2018 EVR23 (OR361559) AAATAATACT GTAGGTGGTT GTAATATGGA TAATCCGTCA TATCAGCAAT 2577
DWV-B Netherlands 2001 VDV-1 (NC_006494) AAATAATACC GTAGGTGGTT GTAATATGGA TAATCCGTCA TATCAGCAAT 2576
DWV-B France 2019 VV144I (MN565037) AAATAATACT GTAGGTGGTT GTAATATGGA TAATCCGTCA TATCAGCAAT 2577
DWV-B Slovenia 2019 341-1 (ON648741) AAATAATACT GTAGGTGGTT GTAATATGGA TAATCCGTCA TATCAGCAAT 2577
DWV-B Austria 2018 B11B (OL803829) AAATAATACT GTAGGTGGTT GTAATATGGA TAATCCGTCA TATCAGCAAT 2577
DWV-A Austria 2014 1414 (KU847397) GACTAACACG ATTGGTGGTA ATAATATGGA TAATCCTTCT TATCAACAAT 2592
DWV-A USA 2003 PA (AY292384) GAATAACACG ATTGGTGGTA ATAATATGGA TAATCCTTCT TATCAACAAT 2590
DWV-A Japan 2001 Kakugo (AB070959) AAATAACACG ATTGGTGGTA ACAATATGGA TAATCCTTCT TATCAACAAT 2587
DWV-C UK 2015 Devon (ERS657949) TAATAATACC ATAGGTGGGT GTAATATGGA TAATCCATCA TACCAGCAAT 2563
DWV-D Egypt 1977 EBV (MT504363) TAATAATACT ATAGGTGGTT GTAATATGGA TAATCCCTCA TATCAACAAT 2586
Darwin bee virus 3 Australia 2014 NT-12 (MG995697) GACGAACGTT GTTGGAGGTC ATAATATGGA TAAACCACCA GAAACTCAGG 2494
Consensus AAATAATACT GTAGGTGGTT GTAATATGGA TAATCCGTCA TATCAGCAAT
100%
Conservation
0%
2.720
2.740
DWV-B Austria 2022 SB22 (PP418870) CTCCGCGTCA TTTTGTTCCT ACTGGTATGC ATAGTTTAGC TTTAGGCACT 2627
DWV-B USA 2018 EVR24 (OR361560) CTCCGCGTCA TTTTGTTCCT ACTGGTATGC ATAGTTTAGC TTTAGGCACT 2627
DWV-B USA 2018 EVR23 (OR361559) CTCCGCGTCA TTTTGTTCCT ACTGGTATGC ATAGTTTAGC TTTAGGCACT 2627
DWV-B Netherlands 2001 VDV-1 (NC_006494) CTCCGCGTCA TTTTGTTCCT ACTGGTATGC ATAGTTTAGC TTTAGGCACT 2626
DWV-B France 2019 VV144I (MN565037) CTCCGCGTCA TTTTGTTCCT ACTGGTATGC ATAGTTTAGC TTTAGGCACT 2627
DWV-B Slovenia 2019 341-1 (ON648741) CTCCGCGTCA TTTTGTTCCT ACTGGTATGC ATAGTTTAGC TTTAGGCACT 2627
DWV-B Austria 2018 B11B (OL803829) CTCCGCGTCA TTTTGTTCCT ACTGGTATGC ATAGTTTAGC TTTAGGCACT 2627
DWV-A Austria 2014 1414 (KU847397) CTCCTCGTCA TTTTGTCCCG ACTGGTATGC ACAGCTTAGC TTTAGGTACT 2642
DWV-A USA 2003 PA (AY292384) CTCCTCGTCA TTTTGTCCCG ACTGGTATGC ACAGCTTAGC TTTAGGTACT 2640
DWV-A Japan 2001 Kakugo (AB070959) CTCCTCGTCA TTTTGTCCCG ACTGGTATGC ACAGTTTAGC TTTAGGTACT 2637
DWV-C UK 2015 Devon (ERS657949) CTCCAAGACA CTTTGTACCT ACCGGAATGC ATAGTTTAGC ACTAGGTACT 2613
DWV-D Egypt 1977 EBV (MT504363) CTCCGAGACA CTTTGTACCT ACTGGGATGC ATAGTTTGGC GTTAGGAACT 2636
Darwin bee virus 3 Australia 2014 NT-12 (MG995697) CACCATCATA TTTAGTACCT ACTGGTGTTC ATTCATTAGC GCACGGGACT 2544
Consensus CTCCGCGTCA TTTTGTTCCT ACTGGTATGC ATAGTTTAGC TTTAGGCACT
100%
Conservation
0%

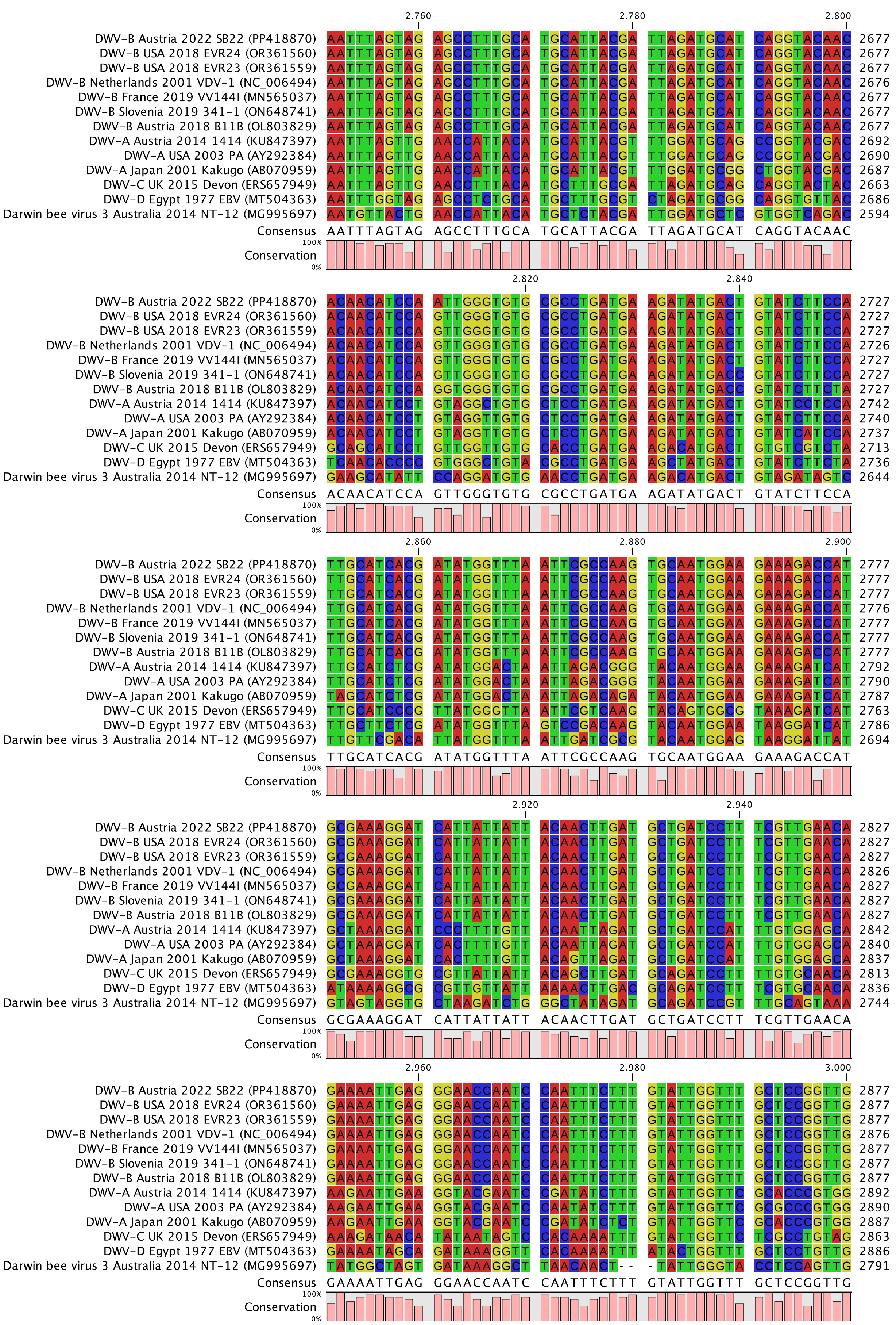
2.760 2.780 2.800
DWV-B Austria 2022 SB22 (PP418870) AATTTAGTAG AGCCTTTGCA TGCATTACGA TTAGATGCAT CAGGTACAAC 2677
DWV-B USA 2018 EVR24 (OR361560) AATTTAGTAG AGCCTTTGCA TGCATTACGA TTAGATGCAT CAGGTACAAC 2677
DWV-B USA 2018 EVR23 (OR361559) AATTTAGTAG AGCCTTTGCA TGCATTACGA TTAGATGCAT CAGGTACAAC 2677
DWV-B Netherlands 2001 VDV-1 (NC_006494) AATTTAGTAG AGCCTTTGCA TGCATTACGA TTAGATGCAT CAGGTACAAC 2676
DWV-B France 2019 VV144I (MN565037) AATTTAGTAG AGCCTTTGCA TGCATTACGA TTAGATGCAT CAGGTACAAC 2677
DWV-B Slovenia 2019 341-1 (ON648741) AATTTAGTAG AGCCTTTGCA TGCATTACGA TTAGATGCAT CAGGTACAAC 2677
DWV-B Austria 2018 B11B (OL803829) AATTTAGTAG AGCCTTTGCA TGCATTACGA TTAGATGCAT CAGGTACAAC 2677
DWV-A Austria 2014 1414 (KU847397) AATTTAGTTG AACCATTACA TGCATTACGT TTGGATGCAG CCGGTACGAC 2692
DWV-A USA 2003 PA (AY292384) AATTTAGTTG AACCATTACA TGCATTACGT TTGGATGCAG CCGGTACGAC 2690
DWV-A Japan 2001 Kakugo (AB070959) AATTTAGTTG AACCATTACA TGCATTACGT TTGGATGCGG CTGGTACGAC 2687
DWV-C UK 2015 Devon (ERS657949) AATTTAGTTG AACCTTTACA TGCTTTGCGA TTAGATGCAG CAGGTACTAC 2663
DWV-D Egypt 1977 EBV (MT504363) AATTTGGTAG AGCCTCTGCA TGCTTTGCGT CTAGATGCGG CAGGTGTTAC 2686
Darwin bee virus 3 Australia 2014 NT-12 (MG995697) AATGTTACTG AACCATTACA TGCTCTACGA TTGGATGCTC GTGGTCAGAC 2594
Consensus AATTTAGTAG AGCCTTTGCA TGCATTACGA TTAGATGCAT CAGGTACAAC
100%
Conservation
0%
2.820 2.840
DWV-B Austria 2022 SB22 (PP418870) ACAACATCCA ATTGGGTGTG CGCCTGATGA AGATATGACT GTATCTTCCA 2727
DWV-B USA 2018 EVR24 (OR361560) ACAACATCCA GTTGGGTGTG CGCCTGATGA AGATATGACT GTATCTTCCA 2727
DWV-B USA 2018 EVR23 (OR361559) ACAACATCCA GTTGGGTGTG CGCCTGATGA AGATATGACT GTATCTTCCA 2727
DWV-B Netherlands 2001 VDV-1 (NC_006494) ACAACATCCA GTTGGGTGTG CGCCTGATGA AGATATGACT GTATCTTCCA 2726
DWV-B France 2019 VV144I (MN565037) ACAACATCCA GTTGGGTGTG CGCCTGATGA AGATATGACT GTATCTTCCA 2727
DWV-B Slovenia 2019 341-1 (ON648741) ACAACATCCA GTTGGGTGTG CGCCTGATGA AGATATGACC GTATCTTCCA 2727
DWV-B Austria 2018 B11B (OL803829) ACAACATCCA GGTGGGTGTG CGCCTGATGA AGATATGACC GTATCTTCTA 2727
DWV-A Austria 2014 1414 (KU847397) ACAACATCCT GTAGGCTGTG CTCCTGATGA AGATATGACT GTATCCTCCA 2742
DWV-A USA 2003 PA (AY292384) ACAACATCCT GTAGGTTGTG CTCCTGATGA AGATATGACT GTATCTTCCA 2740
DWV-A Japan 2001 Kakugo (AB070959) ACAACATCCT GTAGGTTGTG CTCCTGATGA AGATATGACT GTATCATCCA 2737
DWV-C UK 2015 Devon (ERS657949) GCAGCATCCT GTTGGTTGTG CACCTGATGA AGACATGACT GTGTCGTCTA 2713
DWV-D Egypt 1977 EBV (MT504363) TCAACACCCC GTGGGCTGTA CGCCTGATGA AGCTATGACT GTATCTTCTA 2736
Darwin bee virus 3 Australia 2014 NT-12 (MG995697) GAAGCATATT CCAGGATGTG AACCTGATGA AGACATGACT GTAGATAGTC 2644
Consensus ACAACATCCA GTTGGGTGTG CGCCTGATGA AGATATGACT GTATCTTCCA
100%
Conservation
0%
2.860 2.880 2.900
DWV-B Austria 2022 SB22 (PP418870) TTGCATCACG ATATGGTTTA ATTCGCCAAG TGCAATGGAA GAAAGACCAT 2777
DWV-B USA 2018 EVR24 (OR361560) TTGCATCACG ATATGGTTTA ATTCGCCAAG TGCAATGGAA GAAAGACCAT 2777
DWV-B USA 2018 EVR23 (OR361559) TTGCATCACG ATATGGTTTA ATTCGCCAAG TGCAATGGAA GAAAGACCAT 2777
DWV-B Netherlands 2001 VDV-1 (NC_006494) TTGCATCACG ATATGGTTTA ATTCGCCAAG TGCAATGGAA GAAAGACCAT 2776
DWV-B France 2019 VV144I (MN565037) TTGCATCACG ATATGGTTTA ATTCGCCAAG TGCAATGGAA GAAAGACCAT 2777
DWV-B Slovenia 2019 341-1 (ON648741) TTGCATCACG ATATGGTTTA ATTCGCCAAG TGCAATGGAA GAAAGACCAT 2777
DWV-B Austria 2018 B11B (OL803829) TTGCATCACG ATATGGTTTA ATTCGCCAAG TGCAATGGAA GAAAGACCAT 2777
DWV-A Austria 2014 1414 (KU847397) TTGCATCTCG ATATGGACTA ATTAGACGGG TACAATGGAA GAAAGATCAT 2792
DWV-A USA 2003 PA (AY292384) TTGCATCTCG ATATGGACTA ATTAGACGGG TACAATGGAA GAAAGATCAT 2790
DWV-A Japan 2001 Kakugo (AB070959) TAGCATCTCG ATATGGACTA ATTAGACAGA TACAATGGAA GAAAGATCAT 2787
DWV-C UK 2015 Devon (ERS657949) TTGCATCCCG TTATGGGTTA ATTCGTCAAG TACAGTGGCG TAAAGATCAT 2763
DWV-D Egypt 1977 EBV (MT504363) TTGCTTCTCG ATATGGTTTA GTCCGACAAG TACAATGGAA TAAGGATCAT 2786
Darwin bee virus 3 Australia 2014 NT-12 (MG995697) TTGTTCGACA TTATGGTTTA ATTGATCGCG TACAATGGAG TAAGGATTAT 2694
Consensus TTGCATCACG ATATGGTTTA ATTCGCCAAG TGCAATGGAA GAAAGACCAT
100%
Conservation
0%
2.920 2.940
DWV-B Austria 2022 SB22 (PP418870) GCGAAAGGAT CATTATTATT ACAACTTGAT GCTGATCCTT TCGTTGAACA 2827
DWV-B USA 2018 EVR24 (OR361560) GCGAAAGGAT CATTATTATT ACAACTTGAT GCTGATCCTT TCGTTGAACA 2827
DWV-B USA 2018 EVR23 (OR361559) GCGAAAGGAT CATTATTATT ACAACTTGAT GCTGATCCTT TCGTTGAACA 2827
DWV-B Netherlands 2001 VDV-1 (NC_006494) GCGAAAGGAT CATTATTATT ACAACTTGAT GCTGATCCTT TCGTTGAACA 2826
DWV-B France 2019 VV144I (MN565037) GCGAAAGGAT CATTATTATT ACAACTTGAT GCTGATCCTT TCGTTGAACA 2827
DWV-B Slovenia 2019 341-1 (ON648741) GCGAAAGGAT CATTATTATT ACAACTTGAT GCTGATCCTT TCGTTGAACA 2827
DWV-B Austria 2018 B11B (OL803829) GCGAAAGGAT CATTATTATT ACAACTTGAT GCTGATCCTT TCGTTGAACA 2827
DWV-A Austria 2014 1414 (KU847397) GCTAAAGGAT CCCTTTTGTT ACAATTAGAT GCTGATCCAT TTGTGGAGCA 2842
DWV-A USA 2003 PA (AY292384) GCTAAAGGAT CACTTTTGTT ACAATTAGAT GCTGATCCAT TTGTGGAGCA 2840
DWV-A Japan 2001 Kakugo (AB070959) GCTAAAGGAT CACTTTTGTT ACAGTTAGAT GCTGATCCAT TTGTGGAGCA 2837
DWV-C UK 2015 Devon (ERS657949) GCGAAAGGTG CGTTATTATT ACAGCTTGAT GCAGATCCTT TTGTGCAACA 2813
DWV-D Egypt 1977 EBV (MT504363) ATAAAAGGCG CGTTGTTATT AAAACTTGAC GCAGATCCTT TCGTGCAACA 2836
Darwin bee virus 3 Australia 2014 NT-12 (MG995697) GTAGTAGGTG CTAAGATCTG GGCTATAGAT GCAGATCCGT TTGCAGTAAA 2744
Consensus GCGAAAGGAT CATTATTATT ACAACTTGAT GCTGATCCTT TCGTTGAACA
100%
Conservation
0%
2.960 2.980 3.000
DWV-B Austria 2022 SB22 (PP418870) GAAAATTGAG GGAACCAATC CAATTTCTTT GTATTGGTTT GCTCCGGTTG 2877
DWV-B USA 2018 EVR24 (OR361560) GAAAATTGAG GGAACCAATC CAATTTCTTT GTATTGGTTT GCTCCGGTTG 2877
DWV-B USA 2018 EVR23 (OR361559) GAAAATTGAG GGAACCAATC CAATTTCTTT GTATTGGTTT GCTCCGGTTG 2877
DWV-B Netherlands 2001 VDV-1 (NC_006494) GAAAATTGAG GGAACCAATC CAATTTCTTT GTATTGGTTT GCTCCGGTTG 2876
DWV-B France 2019 VV144I (MN565037) GAAAATTGAG GGAACCAATC CAATTTCTTT GTATTGGTTT GCTCCGGTTG 2877
DWV-B Slovenia 2019 341-1 (ON648741) GAAAATTGAG GGAACCAATC CAATTTCTTT GTATTGGTTT GCTCCGGTTG 2877
DWV-B Austria 2018 B11B (OL803829) GAAAATTGAG GGAACCAATC CAATTTCTTT GTATTGGTTT GCTCCGGTTG 2877
DWV-A Austria 2014 1414 (KU847397) AAGAATTGAA GGTACGAATC CGATATCTTT GTATTGGTTC GCACCCGTGG 2892
DWV-A USA 2003 PA (AY292384) AAGAATTGAA GGTACGAATC CAATATCTTT GTATTGGTTC GCGCCCGTGG 2890
DWV-A Japan 2001 Kakugo (AB070959) AAGAATTGAA GGTACGAATC CGATATCTCT GTATTGGTTC GCACCCGTGG 2887
DWV-C UK 2015 Devon (ERS657949) AAAGATAACA TATAATAGTC CACAAAATTT GTATTGGTTC TCGCCTGTAG 2863
DWV-D Egypt 1977 EBV (MT504363) GAAAATAGCA GATAAAGGTT CACAAAATTT ATACTGGTTT GCTCCTGTTG 2886
Darwin bee virus 3 Australia 2014 NT-12 (MG995697) TATGGCTAGT GATAAAGGCT TAACAACT-- -TATTGGGTA GCTCCAGTTG 2791
Consensus GAAAATTGAG GGAACCAATC CAATTTCTTT GTATTGGTTT GCTCCGGTTG
100%
Conservation
0%

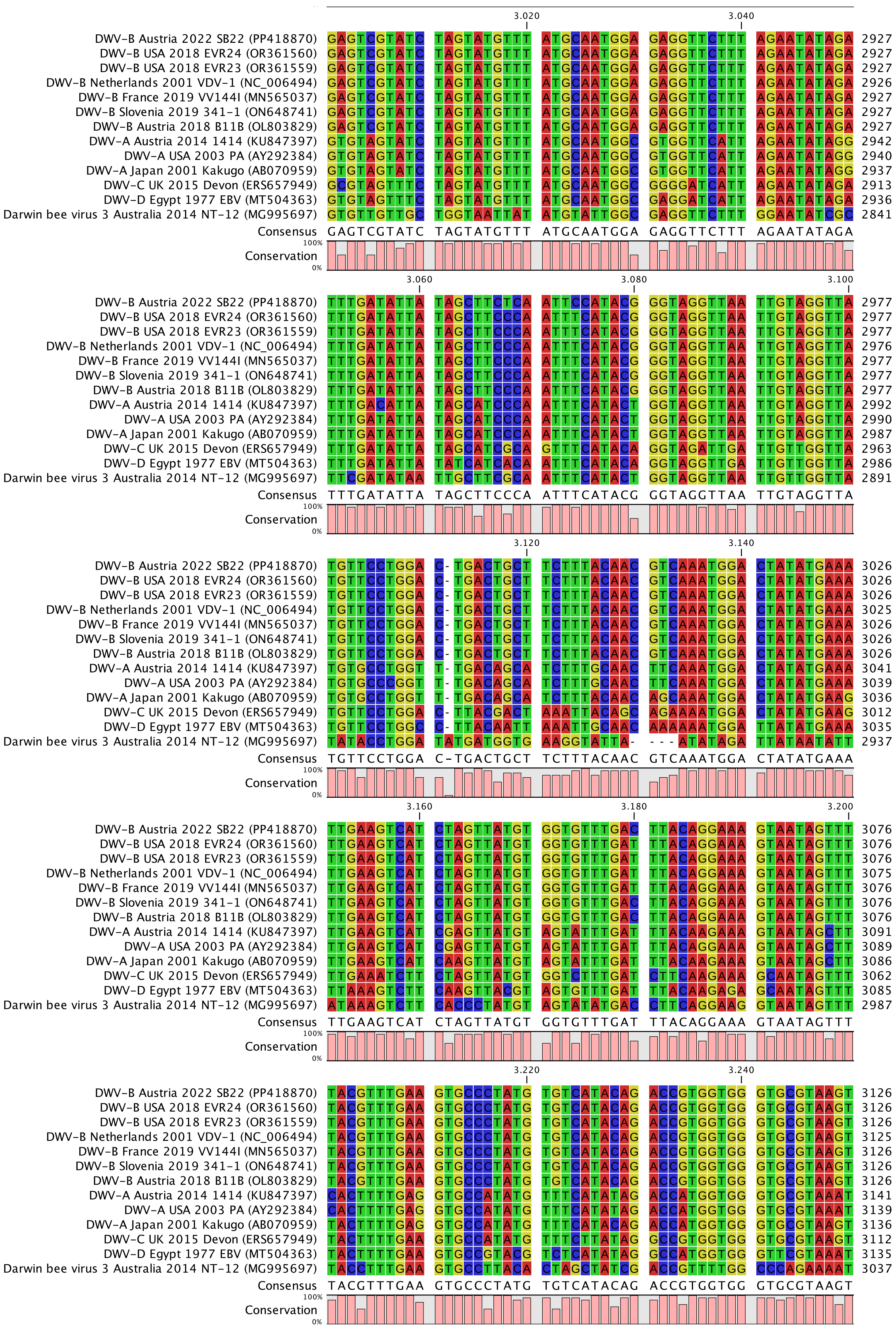

3.020 3.040
DWV-B Austria 2022 SB22 (PP418870) GAGTCGTATC TAGTATGTTT ATGCAATGGA GAGGTTCTTT AGAATATAGA 2927
DWV-B USA 2018 EVR24 (OR361560) GAGTCGTATC TAGTATGTTT ATGCAATGGA GAGGTTCTTT AGAATATAGA 2927
DWV-B USA 2018 EVR23 (OR361559) GAGTCGTATC TAGTATGTTT ATGCAATGGA GAGGTTCTTT AGAATATAGA 2927
DWV-B Netherlands 2001 VDV-1 (NC_006494) GAGTCGTATC TAGTATGTTT ATGCAATGGA GAGGTTCTTT AGAATATAGA 2926
DWV-B France 2019 VV144I (MN565037) GAGTCGTATC TAGTATGTTT ATGCAATGGA GAGGTTCTTT AGAATATAGA 2927
DWV-B Slovenia 2019 341-1 (ON648741) GAGTCGTATC TAGTATGTTT ATGCAATGGA GAGGTTCTTT AGAATATAGA 2927
DWV-B Austria 2018 B11B (OL803829) GAGTCGTATC TAGTATGTTT ATGCAATGGA GAGGTTCTTT AGAATATAGA 2927
DWV-A Austria 2014 1414 (KU847397) GTGTAGTATC TAGTATGTTT ATGCAATGGC GTGGTTCATT AGAATATAGG 2942
DWV-A USA 2003 PA (AY292384) GTGTAGTATC TAGTATGTTT ATGCAATGGC GTGGTTCATT AGAATATAGG 2940
DWV-A Japan 2001 Kakugo (AB070959) GTGTAGTATC TAGTATGTTT ATGCAATGGC GTGGTTCATT AGAATATAGG 2937
DWV-C UK 2015 Devon (ERS657949) GCGTAGTTTC TAGTATGTTT ATGCAATGGC GGGGATCATT AGAATATAGA 2913
DWV-D Egypt 1977 EBV (MT504363) GTGTAGTTTC TAGTATGTTT ATGCAATGGC GAGGATCATT AGAATATAGA 2936
Darwin bee virus 3 Australia 2014 NT-12 (MG995697) GTGTTGTTGC TGGTAATTAT ATGTATTGGC GAGGTTCTTT GGAATATCGC 2841
Consensus GAGTCGTATC TAGTATGTTT ATGCAATGGA GAGGTTCTTT AGAATATAGA
100%
Conservation
0%
3.060 3.080 3.100
DWV-B Austria 2022 SB22 (PP418870) TTTGATATTA TAGCTTCTCA ATTCCATACG GGTAGGTTAA TTGTAGGTTA 2977
DWV-B USA 2018 EVR24 (OR361560) TTTGATATTA TAGCTTCCCA ATTTCATACG GGTAGGTTAA TTGTAGGTTA 2977
DWV-B USA 2018 EVR23 (OR361559) TTTGATATTA TAGCTTCCCA ATTTCATACG GGTAGGTTAA TTGTAGGTTA 2977
DWV-B Netherlands 2001 VDV-1 (NC_006494) TTTGATATTA TAGCTTCCCA ATTTCATACG GGTAGGTTAA TTGTAGGTTA 2976
DWV-B France 2019 VV144I (MN565037) TTTGATATTA TAGCTTCCCA ATTTCATACG GGTAGGTTAA TTGTAGGTTA 2977
DWV-B Slovenia 2019 341-1 (ON648741) TTTGATATTA TAGCTTCCCA ATTTCATACG GGTAGGTTAA TTGTAGGTTA 2977
DWV-B Austria 2018 B11B (OL803829) TTTGATATTA TAGCTTCCCA ATTTCATACG GGTAGGTTAA TTGTAGGTTA 2977
DWV-A Austria 2014 1414 (KU847397) TTTGACATTA TAGCATCCCA ATTTCATACT GGTAGGTTAA TTGTAGGTTA 2992
DWV-A USA 2003 PA (AY292384) TTTGATATTA TAGCATCCCA ATTTCATACT GGTAGGTTAA TTGTAGGTTA 2990
DWV-A Japan 2001 Kakugo (AB070959) TTTGATATTA TAGCATCCCA ATTTCATACT GGTAGGTTAA TTGTAGGTTA 2987
DWV-C UK 2015 Devon (ERS657949) TTTGATATTA TAGCATCGCA GTTTCATACA GGTAGATTGA TTGTTGGTTA 2963
DWV-D Egypt 1977 EBV (MT504363) TTTGATATTA TATCATCACA ATTTCATACA GGTAGGTTGA TTGTTGGTTA 2986
Darwin bee virus 3 Australia 2014 NT-12 (MG995697) TTCGATATAA TTGCTTCGCA ATTTCATACT GGTAGGTTAA TTGTTGGTTA 2891
Consensus TTTGATATTA TAGCTTCCCA ATTTCATACG GGTAGGTTAA TTGTAGGTTA
100%
Conservation
0%
3.120 3.140
DWV-B Austria 2022 SB22 (PP418870) TGTTCCTGGA C-TGACTGCT TCTTTACAAC GTCAAATGGA CTATATGAAA 3026
DWV-B USA 2018 EVR24 (OR361560) TGTTCCTGGA C-TGACTGCT TCTTTACAAC GTCAAATGGA CTATATGAAA 3026
DWV-B USA 2018 EVR23 (OR361559) TGTTCCTGGA C-TGACTGCT TCTTTACAAC GTCAAATGGA CTATATGAAA 3026
DWV-B Netherlands 2001 VDV-1 (NC_006494) TGTTCCTGGA C-TGACTGCT TCTTTACAAC GTCAAATGGA CTATATGAAA 3025
DWV-B France 2019 VV144I (MN565037) TGTTCCTGGA C-TGACTGCT TCTTTACAAC GTCAAATGGA CTATATGAAA 3026
DWV-B Slovenia 2019 341-1 (ON648741) TGTTCCTGGA C-TGACTGCT TCTTTACAAC GTCAAATGGA CTATATGAAA 3026
DWV-B Austria 2018 B11B (OL803829) TGTTCCTGGA C-TGACTGCT TCTTTACAAC GTCAAATGGA CTATATGAAA 3026
DWV-A Austria 2014 1414 (KU847397) TGTGCCTGGT T-TGACAGCA TCTTTGCAAC TTCAAATGGA CTATATGAAA 3041
DWV-A USA 2003 PA (AY292384) TGTGCCCGGT T-TGACAGCA TCTTTGCAAC TTCAAATGGA CTATATGAAA 3039
DWV-A Japan 2001 Kakugo (AB070959) TGTGCCTGGT T-TGACAGCA TCTTTGCAAC AGCAAATGGA CTATATGAAG 3036
DWV-C UK 2015 Devon (ERS657949) TGTTCCTGGA C-TTACGACT AAATTACAGC AGAAAATGGA CTATATGAAG 3012
DWV-D Egypt 1977 EBV (MT504363) TGTTCCTGGC C-TTACAATT AAATTGCAAC AAAAAATGGA TTATATGAAA 3035
Darwin bee virus 3 Australia 2014 NT-12 (MG995697) TATACCTGGA TATGATGGTG AAGGTATTA- ---ATATAGA TTATAATATT 2937
Consensus TGTTCCTGGA C-TGACTGCT TCTTTACAAC GTCAAATGGA CTATATGAAA
100%
Conservation
0%
3.160 3.180 3.200
DWV-B Austria 2022 SB22 (PP418870) TTGAAGTCAT CTAGTTATGT GGTGTTTGAC TTACAGGAAA GTAATAGTTT 3076
DWV-B USA 2018 EVR24 (OR361560) TTGAAGTCAT CTAGTTATGT GGTGTTTGAT TTACAGGAAA GTAATAGTTT 3076
DWV-B USA 2018 EVR23 (OR361559) TTGAAGTCAT CTAGTTATGT GGTGTTTGAT TTACAGGAAA GTAATAGTTT 3076
DWV-B Netherlands 2001 VDV-1 (NC_006494) TTGAAGTCAT CTAGTTATGT GGTGTTTGAT TTACAGGAAA GTAATAGTTT 3075
DWV-B France 2019 VV144I (MN565037) TTGAAGTCAT CTAGTTATGT GGTGTTTGAT TTACAGGAAA GTAATAGTTT 3076
DWV-B Slovenia 2019 341-1 (ON648741) TTGAAGTCAT CTAGTTATGT GGTGTTTGAC TTACAGGAAA GTAATAGTTT 3076
DWV-B Austria 2018 B11B (OL803829) TTGAAGTCAT CTAGTTATGT GGTGTTTGAC TTACAGGAAA GTAATAGTTT 3076
DWV-A Austria 2014 1414 (KU847397) TTGAAGTCAT CGAGTTATGT AGTATTTGAT TTACAAGAAA GTAATAGCTT 3091
DWV-A USA 2003 PA (AY292384) TTGAAGTCAT CGAGTTATGT AGTATTTGAT TTACAGGAAA GTAATAGCTT 3089
DWV-A Japan 2001 Kakugo (AB070959) TTGAAGTCAT CAAGTTATGT AGTATTTGAT TTACAAGAAA GTAATAGCTT 3086
DWV-C UK 2015 Devon (ERS657949) TTGAAATCAT CTAGTTATGT GGTCTTTGAT CTTCAAGAAA GCAATAGTTT 3062
DWV-D Egypt 1977 EBV (MT504363) TTAAAGTCTT CAAGTTACGT AGTGTTTGAT TTACAAGAGA GCAATAGTTT 3085
Darwin bee virus 3 Australia 2014 NT-12 (MG995697) ATAAAGTCTT CACCCTATGT AGTATATGAC CTTCAGGAAG GTAATAGTTT 2987
Consensus TTGAAGTCAT CTAGTTATGT GGTGTTTGAT TTACAGGAAA GTAATAGTTT
100%
Conservation
0%
3.220 3.240
DWV-B Austria 2022 SB22 (PP418870) TACGTTTGAA GTGCCCTATG TGTCATACAG ACCGTGGTGG GTGCGTAAGT 3126
DWV-B USA 2018 EVR24 (OR361560) TACGTTTGAA GTGCCCTATG TGTCATACAG ACCGTGGTGG GTGCGTAAGT 3126
DWV-B USA 2018 EVR23 (OR361559) TACGTTTGAA GTGCCCTATG TGTCATACAG ACCGTGGTGG GTGCGTAAGT 3126
DWV-B Netherlands 2001 VDV-1 (NC_006494) TACGTTTGAA GTGCCCTATG TGTCATACAG ACCGTGGTGG GTGCGTAAGT 3125
DWV-B France 2019 VV144I (MN565037) TACGTTTGAA GTGCCCTATG TGTCATACAG ACCGTGGTGG GTGCGTAAGT 3126
DWV-B Slovenia 2019 341-1 (ON648741) TACGTTTGAA GTGCCCTATG TGTCATACAG ACCGTGGTGG GTGCGTAAGT 3126
DWV-B Austria 2018 B11B (OL803829) TACGTTTGAA GTGCCCTATG TGTCATACAG ACCGTGGTGG GTGCGTAAGT 3126
DWV-A Austria 2014 1414 (KU847397) CACTTTTGAG GTGCCATATG TTTCATATAG ACCATGGTGG GTGCGTAAAT 3141
DWV-A USA 2003 PA (AY292384) CACTTTTGAG GTGCCATATG TTTCATATAG ACCATGGTGG GTGCGTAAAT 3139
DWV-A Japan 2001 Kakugo (AB070959) TACTTTTGAG GTGCCATATG TTTCATACAG ACCATGGTGG GTGCGTAAGT 3136
DWV-C UK 2015 Devon (ERS657949) TACTTTTGAA GTGCCATATG TTTCTTATAG GCCGTGGTGG GTGCGTAAGT 3112
DWV-D Egypt 1977 EBV (MT504363) TACTTTTGAA GTGCCGTACG TCTCATATAG GCCATGGTGG GTTCGTAAAT 3135
Darwin bee virus 3 Australia 2014 NT-12 (MG995697) TACGTTTGAA GTGCCTTACA CTAGCTATCG ACCGTTTTGG CCCAGAAAAT 3037
Consensus TACGTTTGAA GTGCCCTATG TGTCATACAG ACCGTGGTGG GTGCGTAAGT
100%
Conservation
0%

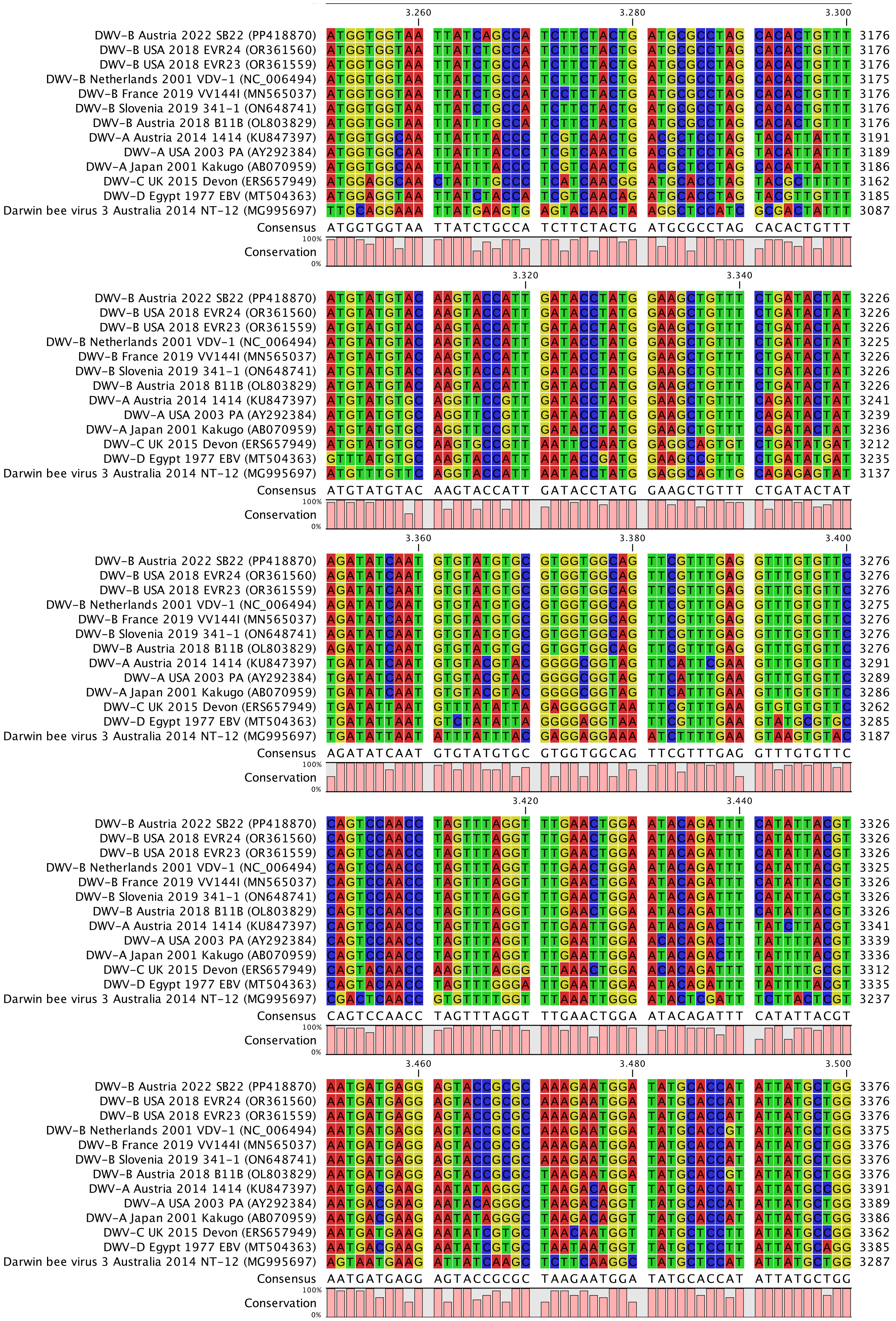
3.260 3.280 3.300
3.320 3.340
3.360 3.380 3.400
3.420 3.440
3.460 3.480 3.500
DWV-B Austria 2022 SB22 (PP418870)
DWV-B USA 2018 EVR24 (OR361560)
DWV-B USA 2018 EVR23 (OR361559)
DWV-B Netherlands 2001 VDV-1 (NC_006494)
DWV-B France 2019 VV144I (MN565037)
DWV-B Slovenia 2019 341-1 (ON648741)
DWV-B Austria 2018 B11B (OL803829)
DWV-A Austria 2014 1414 (KU847397)
DWV-A USA 2003 PA (AY292384)
DWV-A Japan 2001 Kakugo (AB070959)
DWV-C UK 2015 Devon (ERS657949)
DWV-D Egypt 1977 EBV (MT504363)
Darwin bee virus 3 Australia 2014 NT-12 (MG995697)
Consensus ATGGTGGTAA TTATCTGCCA TCTTCTACTG ATGCGCCTAG CACACTGTTT
Consensus ATGTATGTAC AAGTACCATT GATACCTATG GAAGCTGTTT CTGATACTAT
Consensus AGATATCAAT GTGTATGTGC GTGGTGGCAG TTCGTTTGAG GTTTGTGTTC
Consensus CAGTCCAACC TAGTTTAGGT TTGAACTGGA ATACAGATTT CATATTACGT
Consensus AATGATGAGG AGTACCGCGC TAAGAATGGA TATGCACCAT ATTATGCTGG
Conservation
100%
0%
3176 3176 3176 3175 3176 3176 3176 3191 3189 3186 3162 3185 3087
3226 3226 3226 3225 3226 3226 3226 3241 3239 3236 3212 3235 3137
3276 3276 3276 3275 3276 3276 3276 3291 3289 3286 3262 3285 3187
3326 3326 3326 3325 3326 3326 3326 3341 3339 3336 3312 3335 3237
3376 3376 3376 3375 3376 3376 3376 3391 3389 3386 3362 3385 3287

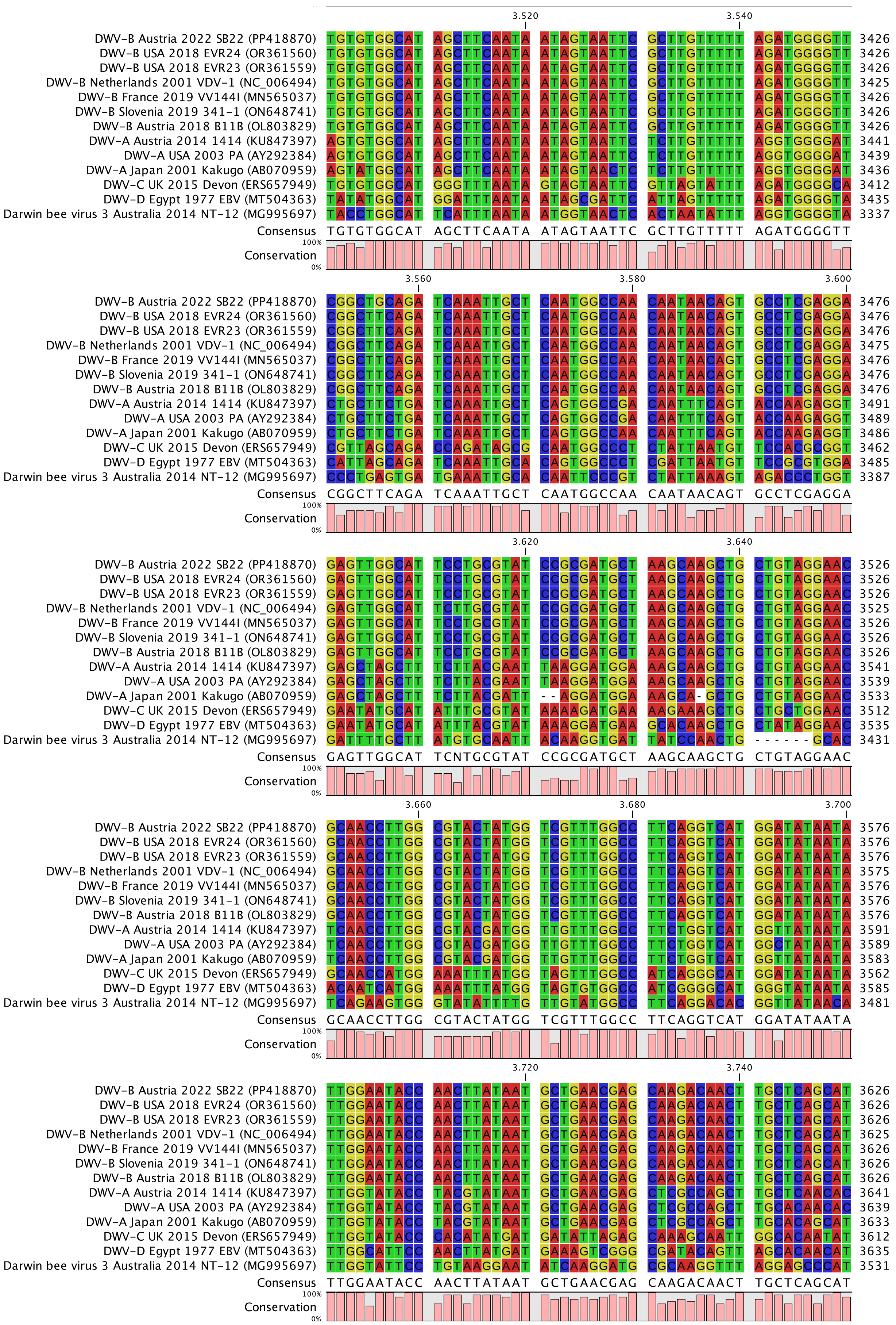
3.520 3.540
DWV-B Austria 2022 SB22 (PP418870) TGTGTGGCAT AGCTTCAATA ATAGTAATTC GCTTGTTTTT AGATGGGGTT 3426
DWV-B USA 2018 EVR24 (OR361560) TGTGTGGCAT AGCTTCAATA ATAGTAATTC GCTTGTTTTT AGATGGGGTT 3426
DWV-B USA 2018 EVR23 (OR361559) TGTGTGGCAT AGCTTCAATA ATAGTAATTC GCTTGTTTTT AGATGGGGTT 3426
DWV-B Netherlands 2001 VDV-1 (NC_006494) TGTGTGGCAT AGCTTCAATA ATAGTAATTC GCTTGTTTTT AGATGGGGTT 3425
DWV-B France 2019 VV1441 (MN565037) TGTGTGGCAT AGCTTCAATA ATAGTAATTC GCTTGTTTTT AGATGGGGTT 3426
DWV-B Slovenia 2019 341-1 (ON648741) TGTGTGGCAT AGCTTCAATA ATAGTAATTC GCTTGTTTTT AGATGGGGTT 3426
DWV-B Austria 2018 B11B (OL803829) TGTGTGGCAT AGCTTCAATA ATAGTAATTC GCTTGTTTTT AGATGGGGTT 3426
DWV-A Austria 2014 1414 (KU847397) AGTGTGGCAT AGCTTCAATA ATAGTAATTC TCTTGTTTTT AGGTGGGGAT 3441
DWV-A USA 2003 PA (AY292384) AGTGTGGCAT AGCTTCAATA ATAGTAATTC TCTTGTTTTT AGGTGGGGAT 3439
DWV-A Japan 2001 Kakugo (AB070959) AGTATGGCAT AGCTTCAATA ATAGTAACTC TCTTGTTTTT AGGTGGGGAT 3436
DWV-C UK 2015 Devon (ERS657949) TGTGTGGCAT GGGTTTAATA GTAGTAATTC GTTAGTATTT AGATGGGGCA 3412
DWV-D Egypt 1977 EBV (MT504363) TATATGGCAT GGATTTAATA ATAGCGATTC ATTAGTTTTT AGATGGGGTA 3435
Darwin bee virus 3 Australia 2014 NT-12 (MG995697) TACCTGGCAT TCATTTAATA ATGGTAACTC ACTAATATTT AGGTGGGGTA 3337
Consensus TGTGTGGCAT AGCTTCAATA ATAGTAATTC GCTTGTTTTT AGATGGGGTT
100%
Conservation
0%
3.560 3.580 3.600
DWV-B Austria 2022 SB22 (PP418870) CGGCTGCAGA TCAAATTGCT CAATGGCCAA CAATAACAGT GCCTCGAGGA 3476
DWV-B USA 2018 EVR24 (OR361560) CGGCTTCAGA TCAAATTGCT CAATGGCCAA CAATAACAGT GCCTCGAGGA 3476
DWV-B USA 2018 EVR23 (OR361559) CGGCTTCAGA TCAAATTGCT CAATGGCCAA CAATAACAGT GCCTCGAGGA 3476
DWV-B Netherlands 2001 VDV-1 (NC_006494) CGGCTTCAGA TCAAATTGCT CAATGGCCAA CAATAACAGT GCCTCGAGGA 3475
DWV-B France 2019 VV1441 (MN565037) CGGCTTCAGA TCAAATTGCT CAATGGCCAA CAATAACAGT GCCTCGAGGA 3476
DWV-B Slovenia 2019 341-1 (ON648741) CGGCTTCAGA TCAAATTGCT CAATGGCCAA CAATAACAGT GCCTCGAGGA 3476
DWV-B Austria 2018 B11B (OL803829) CGGCTTCAGA TCAAATTGCT CAATGGCCAA CAATAACAGT GCCTCGAGGA 3476
DWV-A Austria 2014 1414 (KU847397) CTGCTTCTGA TCAAATTGCT CAGTGGCCGA CAATTTCAGT ACCAAGAGGT 3491
DWV-A USA 2003 PA (AY292384) CTGCTTCTGA TCAAATTGCT CAGTGGCCGA CAATTTCAGT ACCAAGAGGT 3489
DWV-A Japan 2001 Kakugo (AB070959) CTGCTTCTGA TCAAATTGCT CAGTGGCCAA CAATTTCAGT ACCAAGAGGT 3486
DWV-C UK 2015 Devon (ERS657949) CGTTAGCAGA CCAGATAGCG CAATGGCCCT CTATTAATGT TCCACGCGGT 3462
DWV-D Egypt 1977 EBV (MT504363) CATTAGCAGA TCAAATTGCA CAGTGGCCCT CGATTAATGT TCCGCGTGGA 3485
Darwin bee virus 3 Australia 2014 NT-12 (MG995697) CCCTGAGTGA TGAAATTGCA CAATTCCCGT CTATTAAAGT AGACCCTGGT 3387
Consensus CGGCTTCAGA TCAAATTGCT CAATGGCCAA CAATAACAGT GCCTCGAGGA
100%
Conservation
0%
3.620 3.640
DWV-B Austria 2022 SB22 (PP418870) GAGTTGGCAT TCCTGCGTAT CCGCGATGCT AAGCAAGCTG CTGTAGGAAC 3526
DWV-B USA 2018 EVR24 (OR361560) GAGTTGGCAT TCCTGCGTAT CCGCGATGCT AAGCAAGCTG CTGTAGGAAC 3526
DWV-B USA 2018 EVR23 (OR361559) GAGTTGGCAT TCCTGCGTAT CCGCGATGCT AAGCAAGCTG CTGTAGGAAC 3526
DWV-B Netherlands 2001 VDV-1 (NC_006494) GAGTTGGCAT TCTTGCGTAT CCGCGATGCT AAGCAAGCTG CTGTAGGAAC 3525
DWV-B France 2019 VV1441 (MN565037) GAGTTGGCAT TCCTGCGTAT CCGCGATGCT AAGCAAGCTG CTGTAGGAAC 3526
DWV-B Slovenia 2019 341-1 (ON648741) GAGTTGGCAT TCCTGCGTAT CCGCGATGCT AAGCAAGCTG CTGTAGGAAC 3526
DWV-B Austria 2018 B11B (OL803829) GAGTTGGCAT TCCTGCGTAT CCGCGATGCT AAGCAAGCTG CTGTAGGAAC 3526
DWV-A Austria 2014 1414 (KU847397) GAGCTAGCTT TCTTACGAAT TAAGGATGGA AAGCAAGCTG CTGTAGGAAC 3541
DWV-A USA 2003 PA (AY292384) GAGCTAGCTT TCTTACGAAT TAAGGATGGA AAGCAAGCTG CTGTAGGAAC 3539
DWV-A Japan 2001 Kakugo (AB070959) GAGCTAGCTT TCTTACGATT --AGGATGGA AAGCA-GCTG CTGTAGGAAC 3533
DWV-C UK 2015 Devon (ERS657949) GAATATGCAT ATTTGCGTAT AAAAGATGAA AAGAAAGCTG CTGCTGGAAC 3512
DWV-D Egypt 1977 EBV (MT504363) GAATATGCAT ATTTACGTAT AAAGGATGAA GCACAAGCTG CTATAGGAAC 3535
Darwin bee virus 3 Australia 2014 NT-12 (MG995697) GATTTTGCTT ATGTGCAATT ACAAGGTGAT TATCCAACTG ------GCAC 3431
Consensus GAGTTGGCAT TCNTGCGTAT CCGCGATGCT AAGCAAGCTG CTGTAGGAAC
100%
Conservation
0%
3.660 3.680 3.700
DWV-B Austria 2022 SB22 (PP418870) GCAACCTTGG CGTACTATGG TCGTTTGGCC TTCAGGTCAT GGATATAATA 3576
DWV-B USA 2018 EVR24 (OR361560) GCAACCTTGG CGTACTATGG TCGTTTGGCC TTCAGGTCAT GGATATAATA 3576
DWV-B USA 2018 EVR23 (OR361559) GCAACCTTGG CGTACTATGG TCGTTTGGCC TTCAGGTCAT GGATATAATA 3576
DWV-B Netherlands 2001 VDV-1 (NC_006494) GCAACCTTGG CGTACTATGG TCGTTTGGCC TTCAGGTCAT GGATATAATA 3575
DWV-B France 2019 VV1441 (MN565037) GCAACCTTGG CGTACTATGG TCGTTTGGCC TTCAGGTCAT GGATATAATA 3576
DWV-B Slovenia 2019 341-1 (ON648741) GCAACCTTGG CGTACTATGG TCGTTTGGCC TTCAGGTCAT GGATATAATA 3576
DWV-B Austria 2018 B11B (OL803829) GCAACCTTGG CGTACTATGG TCGTTTGGCC TTCAGGTCAT GGATATAATA 3576
DWV-A Austria 2014 1414 (KU847397) TCAACCTTGG CGTACGATGG TTGTTTGGCC TTCTGGTCAT GGTTATAATA 3591
DWV-A USA 2003 PA (AY292384) TCAACCTTGG CGTACGATGG TTGTTTGGCC TTCTGGTCAT GGCTATAATA 3589
DWV-A Japan 2001 Kakugo (AB070959) TCAACCTTGG CGTACGATGG TTGTTTGGCC TTCTGGTCAT GGTTATAATA 3583
DWV-C UK 2015 Devon (ERS657949) GCAACCATGG AAATTTATGG TAGTTTGGCC ATCAGGGCAT GGATATAATA 3562
DWV-D Egypt 1977 EBV (MT504363) ACAATCATGG AAATTTATGG TAGTGTGGCC ATCGGGGCAT GGGTATAATA 3585
Darwin bee virus 3 Australia 2014 NT-12 (MG995697) TCAGAAGTGG GTATATTTTG TTGTATGGCC TTCAGGACAC GGTTATAACA 3481
Consensus GCAACCTTGG CGTACTATGG TCGTTTGGCC TTCAGGTCAT GGATATAATA
100%
Conservation
0%
3.720 3.740
DWV-B Austria 2022 SB22 (PP418870) TTGGAATACC AACTTATAAT GCTGAACGAG CAAGACAACT TGCTCAGCAT 3626
DWV-B USA 2018 EVR24 (OR361560) TTGGAATACC AACTTATAAT GCTGAACGAG CAAGACAACT TGCTCAGCAT 3626
DWV-B USA 2018 EVR23 (OR361559) TTGGAATACC AACTTATAAT GCTGAACGAG CAAGACAACT TGCTCAGCAT 3626
DWV-B Netherlands 2001 VDV-1 (NC_006494) TTGGAATACC AACTTATAAT GCTGAACGAG CAAGACAACT TGCTCAGCAT 3625
DWV-B France 2019 VV1441 (MN565037) TTGGAATACC AACTTATAAT GCTGAACGAG CAAGACAACT TGCTCAGCAT 3626
DWV-B Slovenia 2019 341-1 (ON648741) TTGGAATACC AACTTATAAT GCTGAACGAG CAAGACAACT TGCTCAGCAT 3626
DWV-B Austria 2018 B11B (OL803829) TTGGAATACC AACTTATAAT GCTGAACGAG CAAGACAACT TGCTCAGCAT 3626
DWV-A Austria 2014 1414 (KU847397) TTGGTATACC TACGTATAAT GCTGAACGAG CTCGCCAGCT TGCTCAACAC 3641
DWV-A USA 2003 PA (AY292384) TTGGTATACC TACGTATAAT GCTGAACGAG CTCGCCAGCT TGCACAACAC 3639
DWV-A Japan 2001 Kakugo (AB070959) TTGGTATACC TACGTATAAT GCTGAACGAG CTCGCCAGCT TGCACAGCAT 3633
DWV-C UK 2015 Devon (ERS657949) TTGGTATACC CACATATGAT GATATTAGAG CAAAGCAATT GGCACAATAT 3612
DWV-D Egypt 1977 EBV (MT504363) TTGGCATTCC AACTTATGAT GAAAGTCGGG CGATACAGTT AGCACAACAT 3635
Darwin bee virus 3 Australia 2014 NT-12 (MG995697) TTGGTATTCC TGTAAGGAAT ATCAAGGATG GGCAAGGTTT AGGAGCCCAT 3531
Consensus TTGGAATACC AACTTATAAT GCTGAACGAG CAAGACAACT TGCTCAGCAT
100%
Conservation
0%

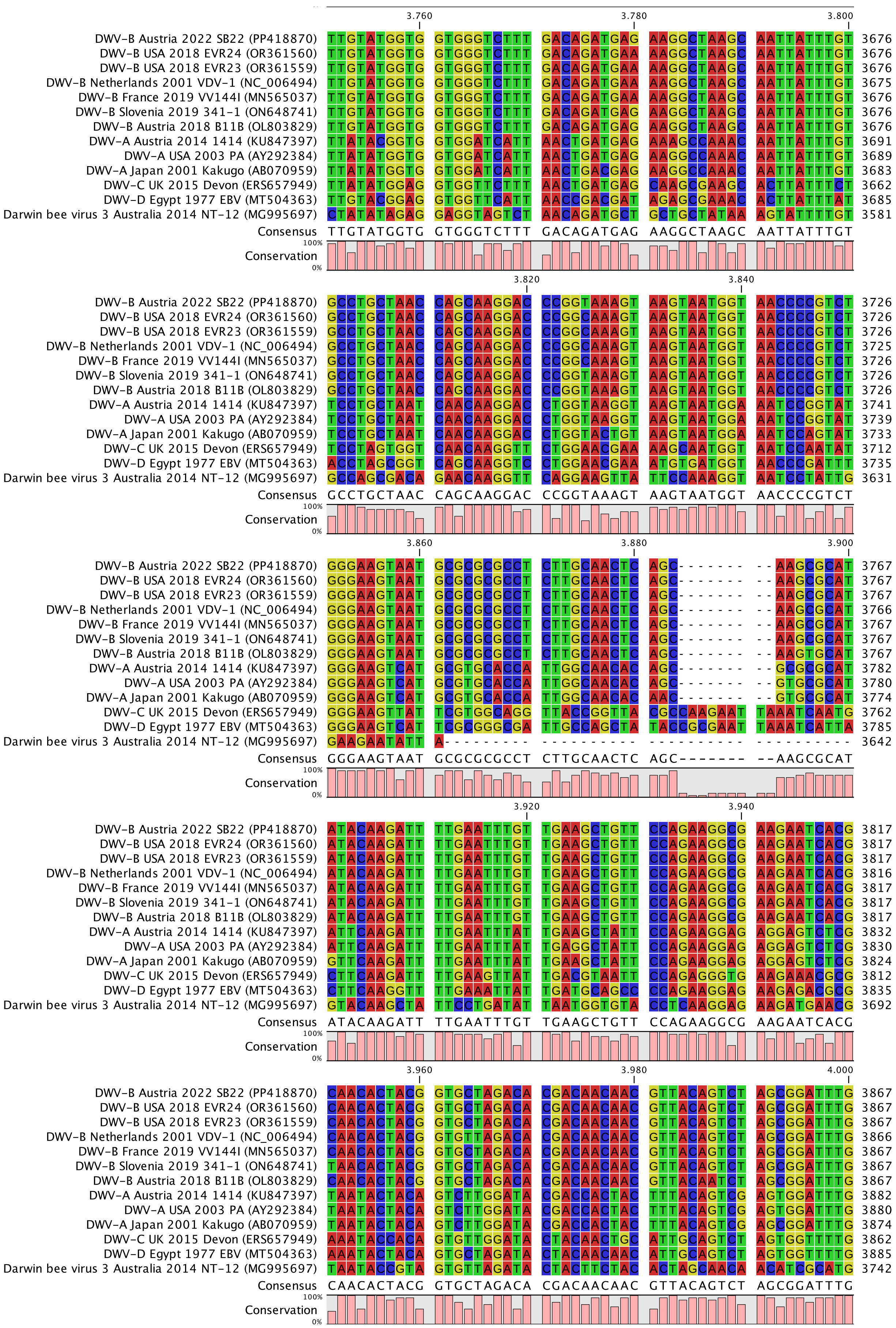
3.760 3.780 3.800
DWV-B Austria 2022 SB22 (PP418870) TTGTATGGTG GTGGGTCTTT GACAGATGAG AAGGCTAAGC AATTATTTGT 3676
DWV-B USA 2018 EVR24 (OR361560) TTGTATGGTG GTGGGTCTTT GACAGATGAA AAGGCTAAGC AATTATTTGT 3676
DWV-B USA 2018 EVR23 (OR361559) TTGTATGGTG GTGGGTCTTT GACAGATGAA AAGGCTAAGC AATTATTTGT 3676
DWV-B Netherlands 2001 VDV-1 (NC_006494) TTGTATGGTG GTGGGTCTTT GACAGATGAA AAGGCTAAGC AATTATTTGT 3675
DWV-B France 2019 VV144I (MN565037) TTGTATGGTG GTGGGTCTTT GACAGATGAA AAGGCTAAGC AATTATTTGT 3676
DWV-B Slovenia 2019 341-1 (ON648741) TTGTATGGTG GTGGGTCTTT GACAGATGAG AAGGCTAAGC AATTATTTGT 3676
DWV-B Austria 2018 B11B (OL803829) TTGTATGGTG GTGGGTCTTT GACAGATGAG AAGGCTAAGC AATTATTTGT 3676
DWV-A Austria 2014 1414 (KU847397) TTATACGGTG GTGGATCATT AACTGATGAG AAAGCCAAAC AATTATTTGT 3691
DWV-A USA 2003 PA (AY292384) TTATATGGTG GTGGATCATT AACTGATGAG AAGGCCAAAC AATTATTTGT 3689
DWV-A Japan 2001 Kakugo (AB070959) TTATATGGTG GTGGATCATT AACTGACGAG AAGGCCAAAC AATTATTTGT 3683
DWV-C UK 2015 Devon (ERS657949) TTATATGGAG GTGGTTCTTT AACTGATGAG CAAGCGAAGC ACTTATTTCT 3662
DWV-D Egypt 1977 EBV (MT504363) TTGTACGGAG GTGGTTCATT AACCGACGAT AGAGCGAAAC ACTTATTTAT 3685
Darwin bee virus 3 Australia 2014 NT-12 (MG995697) CTATATAGAG GAGGTAGTCT AACAGATGCT GCTGCTATAA AGTATTTTGT 3581
Consensus TTGTATGGTG GTGGGTCTTT GACAGATGAG AAGGCTAAGC AATTATTTGT
100%
Conservation
0%
3.820 3.840
DWV-B Austria 2022 SB22 (PP418870) GCCTGCTAAC CAGCAAGGAC CCGGTAAAGT AAGTAATGGT AACCCCGTCT 3726
DWV-B USA 2018 EVR24 (OR361560) GCCTGCTAAC CAGCAAGGAC CCGGCAAAGT AAGTAATGGT AACCCCGTCT 3726
DWV-B USA 2018 EVR23 (OR361559) GCCTGCTAAC CAGCAAGGAC CCGGCAAAGT AAGTAATGGT AACCCCGTCT 3726
DWV-B Netherlands 2001 VDV-1 (NC_006494) GCCTGCTAAC CAGCAAGGAC CCGGCAAAGT AAGTAATGGT AACCCCGTCT 3725
DWV-B France 2019 VV144I (MN565037) GCCTGCTAAC CAGCAAGGAC CCGGCAAAGT AAGTAATGGT AACCCCGTCT 3726
DWV-B Slovenia 2019 341-1 (ON648741) GCCTGCTAAC CAGCAAGGAC CCGGTAAAGT AAGTAATGGT AACCCCGTCT 3726
DWV-B Austria 2018 B11B (OL803829) GCCTGCTAAC CAGCAAGGAC CCGGTAAAGT AAGTAATGGT AACCCCGTCT 3726
DWV-A Austria 2014 1414 (KU847397) TCCTGCTAAT CAACAAGGAC CTGGTAAGGT AAGTAATGGA AATCCGGTAT 3741
DWV-A USA 2003 PA (AY292384) TCCTGCTAAT CAACAAGGAC CTGGTAAGGT AAGTAATGGA AATCCGGTAT 3739
DWV-A Japan 2001 Kakugo (AB070959) TCCTGCTAAT CAACAAGGAC CTGGTACTGT AAGTAATGGA AATCCAGTAT 3733
DWV-C UK 2015 Devon (ERS657949) TCCTAGTGGT CAACAAGGTT CTGGAACGAA AAGCAATGGT AATCCAATAT 3712
DWV-D Egypt 1977 EBV (MT504363) ACCTAGCGGT CAGCAAGGTC CTGGAACGAA ATGTGATGGT AACCCGATTT 3735
Darwin bee virus 3 Australia 2014 NT-12 (MG995697) GCCAGCGACA GAACAAGGTT CAGGAAGTTA TTCCAAAGGT AATCCTATTG 3631
Consensus GCCTGCTAAC CAGCAAGGAC CCGGTAAAGT AAGTAATGGT AACCCCGTCT
100%
Conservation
0%
3.860 3.880 3.900
DWV-B Austria 2022 SB22 (PP418870) GGGAAGTAAT GCGCGCGCCT CTTGCAACTC AGC------- --AAGCGCAT 3767
DWV-B USA 2018 EVR24 (OR361560) GGGAAGTAAT GCGCGCGCCT CTTGCAACTC AGC------- --AAGCGCAT 3767
DWV-B USA 2018 EVR23 (OR361559) GGGAAGTAAT GCGCGCGCCT CTTGCAACTC AGC------- --AAGCGCAT 3767
DWV-B Netherlands 2001 VDV-1 (NC_006494) GGGAAGTAAT GCGCGCGCCT CTTGCAACTC AGC------- --AAGCGCAT 3766
DWV-B France 2019 VV144I (MN565037) GGGAAGTAAT GCGCGCGCCT CTTGCAACTC AGC------- --AAGCGCAT 3767
DWV-B Slovenia 2019 341-1 (ON648741) GGGAAGTAAT GCGCGCGCCT CTTGCAACTC AGC------- --AAGCGCAT 3767
DWV-B Austria 2018 B11B (OL803829) GGGAAGTAAT GCGCGCGCCT CTTGCAACTC AGC------- --AAGTGCAT 3767
DWV-A Austria 2014 1414 (KU847397) GGGAAGTCAT GCGTGCACCA TTGGCAACAC AGC------- --GCGCGCAT 3782
DWV-A USA 2003 PA (AY292384) GGGAAGTCAT GCGTGCACCA TTGGCAACAC AGC------- --GTGCGCAT 3780
DWV-A Japan 2001 Kakugo (AB070959) GGGAAGTCAT GCGTGCACCA TTGGCAACAC AAC------- --GTGCGCAT 3774
DWV-C UK 2015 Devon (ERS657949) GGGAAGTTAT TCGTGGCAGG TTACCGGTTA CGCCAAGAAT TAAATCAATG 3762
DWV-D Egypt 1977 EBV (MT504363) GGGAAGTCAT TCGCGGGCGA TTGCCAGCTA TACCGCGAAT TAAATCATTA 3785
Darwin bee virus 3 Australia 2014 NT-12 (MG995697) GAAGAATATT A--------- ---------- ---------- ---------- 3642
Consensus GGGAAGTAAT GCGCGCGCCT CTTGCAACTC AGC------- --AAGCGCAT
100%
Conservation
0%
3.920 3.940
DWV-B Austria 2022 SB22 (PP418870) ATACAAGATT TTGAATTTGT TGAAGCTGTT CCAGAAGGCG AAGAATCACG 3817
DWV-B USA 2018 EVR24 (OR361560) ATACAAGATT TTGAATTTGT TGAAGCTGTT CCAGAAGGCG AAGAATCACG 3817
DWV-B USA 2018 EVR23 (OR361559) ATACAAGATT TTGAATTTGT TGAAGCTGTT CCAGAAGGCG AAGAATCACG 3817
DWV-B Netherlands 2001 VDV-1 (NC_006494) ATACAAGATT TTGAATTTGT TGAAGCTGTT CCAGAAGGCG AAGAATCACG 3816
DWV-B France 2019 VV144I (MN565037) ATACAAGATT TTGAATTTGT TGAAGCTGTT CCAGAAGGCG AAGAATCACG 3817
DWV-B Slovenia 2019 341-1 (ON648741) ATACAAGATT TTGAATTTGT TGAAGCTGTT CCAGAAGGCG AAGAATCACG 3817
DWV-B Austria 2018 B11B (OL803829) ATACAAGATT TTGAATTTGT TGAAGCTGTT CCAGAAGGCG AAGAATCACG 3817
DWV-A Austria 2014 1414 (KU847397) ATTCAAGATT TTGAATTTAT TGAAGCTATT CCAGAAGGAG AGGAGTCTCG 3832
DWV-A USA 2003 PA (AY292384) ATTCAAGATT TTGAATTTAT TGAGGCTATT CCAGAAGGAG AGGAGTCTCG 3830
DWV-A Japan 2001 Kakugo (AB070959) GTTCAAGATT TTGAATTTAT TGAAGCTATT CCAGAAGGAG AGGAGTCTCG 3824
DWV-C UK 2015 Devon (ERS657949) CTTCAAGATT TTGAAGTTAT TGACGTAATT CCAGAGGGTG AAGAAACGCG 3812
DWV-D Egypt 1977 EBV (MT504363) CTTCAAGGTT TTGAAATTAT TGATGCAGCC CCAGAAGGAG AAGAGACGCG 3835
Darwin bee virus 3 Australia 2014 NT-12 (MG995697) GTACAAGCTA TTCCTGATAT TAATGGTGTA CCTCAAGGAG AAGATGAACG 3692
Consensus ATACAAGATT TTGAATTTGT TGAAGCTGTT CCAGAAGGCG AAGAATCACG
100%
Conservation
0%
3.960 3.980 4.000
DWV-B Austria 2022 SB22 (PP418870) CAACACTACG GTGCTAGACA CGACAACAAC GTTACAGTCT AGCGGATTTG 3867
DWV-B USA 2018 EVR24 (OR361560) CAACACTACG GTGCTAGACA CGACAACAAC GTTACAGTCT AGCGGATTTG 3867
DWV-B USA 2018 EVR23 (OR361559) CAACACTACG GTGCTAGACA CGACAACAAC GTTACAGTCT AGCGGATTTG 3867
DWV-B Netherlands 2001 VDV-1 (NC_006494) CAACACTACG GTGTTAGACA CGACAACAAC GTTACAGTCT AGCGGATTTG 3866
DWV-B France 2019 VV144I (MN565037) CAACACTACG GTGCTAGACA CGACAACAAC GTTACAGTCT AGCGGATTTG 3867
DWV-B Slovenia 2019 341-1 (ON648741) TAACACTACG GTGCTAGACA CGACAACAAC GTTACAGTCT AGCGGATTTG 3867
DWV-B Austria 2018 B11B (OL803829) CAACACTACG GTGCTAGACA CGACAACAAC GTTACAATCT AGCGGATTTG 3867
DWV-A Austria 2014 1414 (KU847397) TAATACTACA GTCTTGGATA CGACCACTAC TTTACAGTCG AGTGGATTTG 3882
DWV-A USA 2003 PA (AY292384) TAATACTACA GTCTTGGATA CGACCACTAC TTTACAGTCG AGTGGATTTG 3880
DWV-A Japan 2001 Kakugo (AB070959) TAATACTACA GTCTTGGATA CGACCACTAC TTTACAGTCG AGCGGATTTG 3874
DWV-C UK 2015 Devon (ERS657949) AAATACCACA GTGTTGGATA CTACAACTGC ATTGCAGTCT AGTGGTTTTG 3862
DWV-D Egypt 1977 EBV (MT504363) AAATACTACA GTGCTAGATA CTACAACAAC ATTGCAGTCT AGTGGTTTTG 3885
Darwin bee virus 3 Australia 2014 NT-12 (MG995697) TAATACCGTA GTGTTAGATA CTACTTCTAC ACTAGCAACA ACATCGCATG 3742
Consensus CAACACTACG GTGCTAGACA CGACAACAAC GTTACAGTCT AGCGGATTTG
100%
Conservation
0%

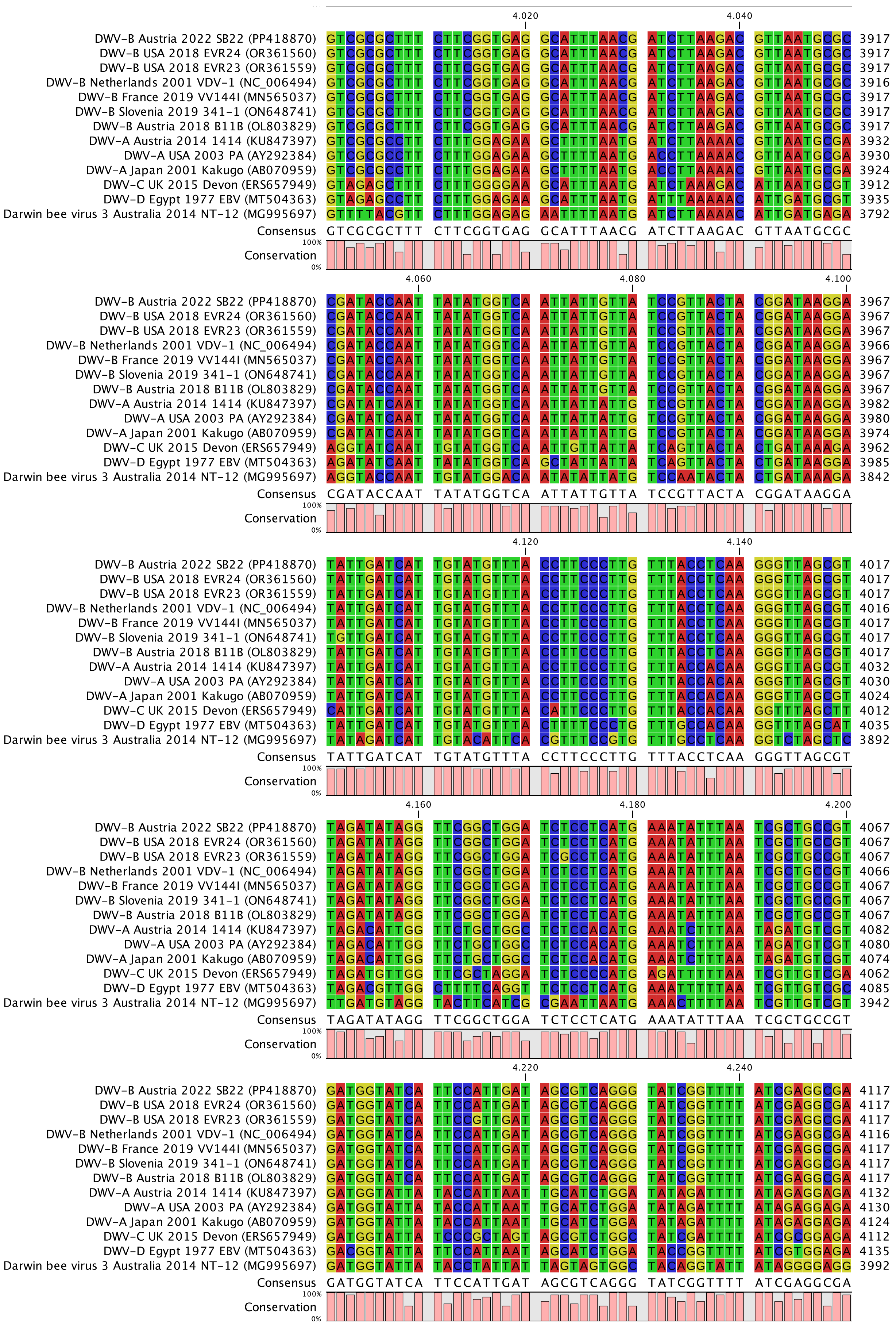
DWV-B Austria 2022 SB22 (PP418870)
DWV-B USA 2018 EVR24 (OR361560)
DWV-B USA 2018 EVR23 (OR361559)
DWV-B Netherlands 2001 VDV-1 (NC_006494)
DWV-B France 2019 VV144I (MN565037)
DWV-B Slovenia 2019 341-1 (ON648741)
DWV-B Austria 2018 B11B (OL803829)
DWV-A Austria 2014 1414 (KU847397)
DWV-A USA 2003 PA (AY292384)
DWV-A Japan 2001 Kakugo (AB070959)
DWV-C UK 2015 Devon (ERS657949)
DWV-D Egypt 1977 EBV (MT504363)
Darwin bee virus 3 Australia 2014 NT-12 (MG995697)
Consensus GTCGCGCTTT CTTCGGTGAG GCATTTAACG ATCTTAAGAC GTTAATGCGC
Consensus CGATACCAAT TATATGGTCA ATTATTGTTA TCCGTTACTA CGGATAAGGA
Consensus TATTGATCAT TGTATGTTTA CCTTCCCTTG TTTACCTCAA GGGTTAGCGT
Consensus TAGATATAGG TTCGGCTGGA TCTCCTCATG AAATATTTAA TCGCTGCCGT
Consensus GATGGTATCA TTCCATTGAT AGCGTCAGGG TATCGGTTTT ATCGAGGCGA
Conservation
100%
0%
4.020
4.040
4.060
4.080
4.100
4.120
4.140
4.160
4.180
4.200
4.220
4.240

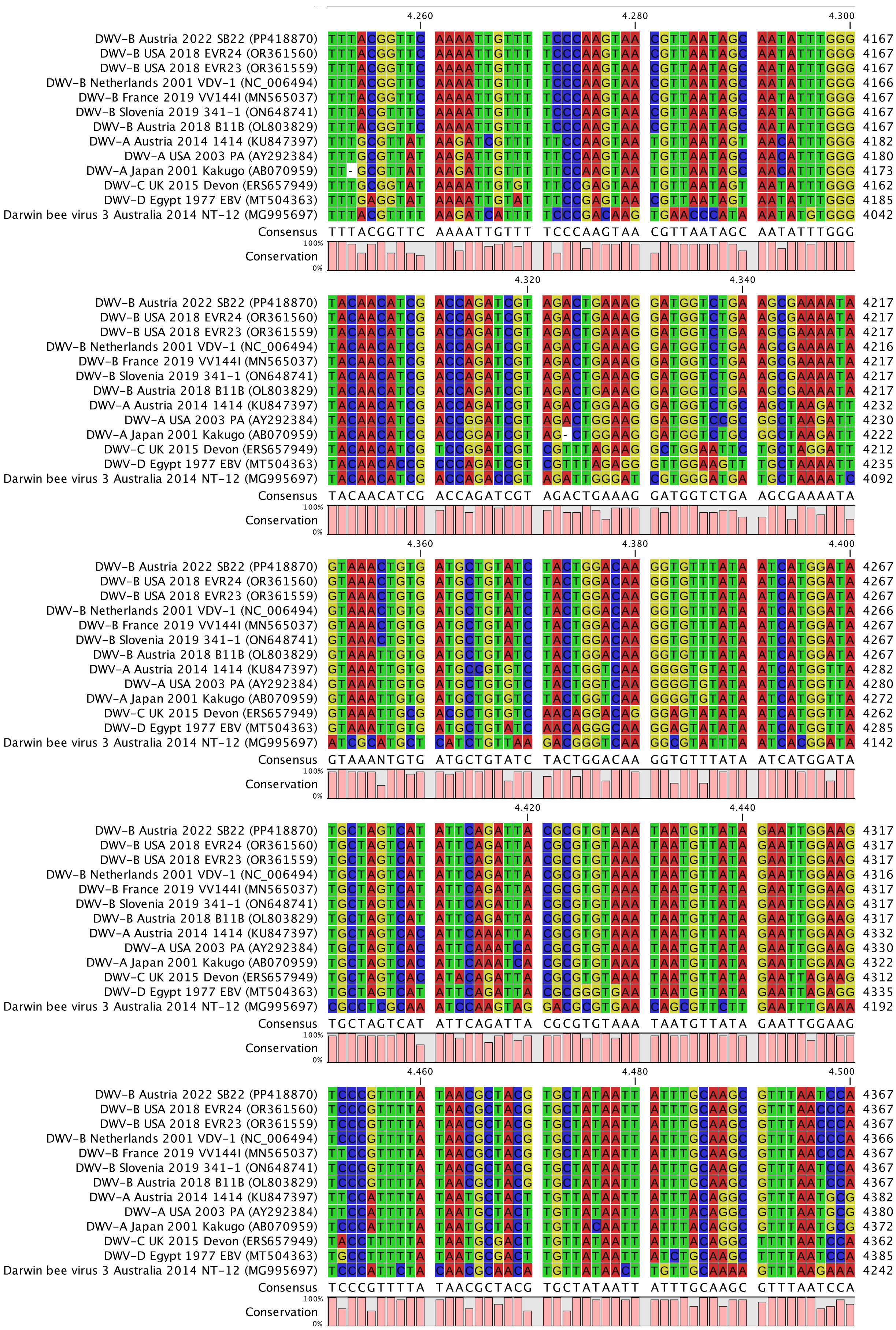
DWV-B Austria 2022 SB22 (PP418870)
DWV-B USA 2018 EVR24 (OR361560)
DWV-B USA 2018 EVR23 (OR361559)
DWV-B Netherlands 2001 VDV-1 (NC_006494)
DWV-B France 2019 VV1441 (MN565037)
DWV-B Slovenia 2019 341-1 (ON648741)
DWV-B Austria 2018 B11B (OL803829)
DWV-A Austria 2014 1414 (KU847397)
DWV-A USA 2003 PA (AY292384)
DWV-A Japan 2001 Kakugo (AB070959)
DWV-C UK 2015 Devon (ERS657949)
DWV-D Egypt 1977 EBV (MT504363)
Darwin bee virus 3 Australia 2014 NT-12 (MG995697)
Consensus TTTACGGTTC AAAATTGTTT TCCCAAGTAA CGTTAATAGC AATATTTGGG
Consensus TACAACATCG ACCAGATCGT AGACTGAAAG GATGGTCTGA AGCGAAAATA
Consensus GTAAANTGTG ATGCTGTATC TACTGGACAA GGTGTTTATA ATCATGGATA
Consensus TGCTAGTCAT ATTCAGATTA CGCGTGTAAA TAATGTTATA GAATTGGAAG
Consensus TCCCGTTTTA TAACGCTACG TGCTATAATT ATTTGCAAGC GTTTAATCCA
Conservation

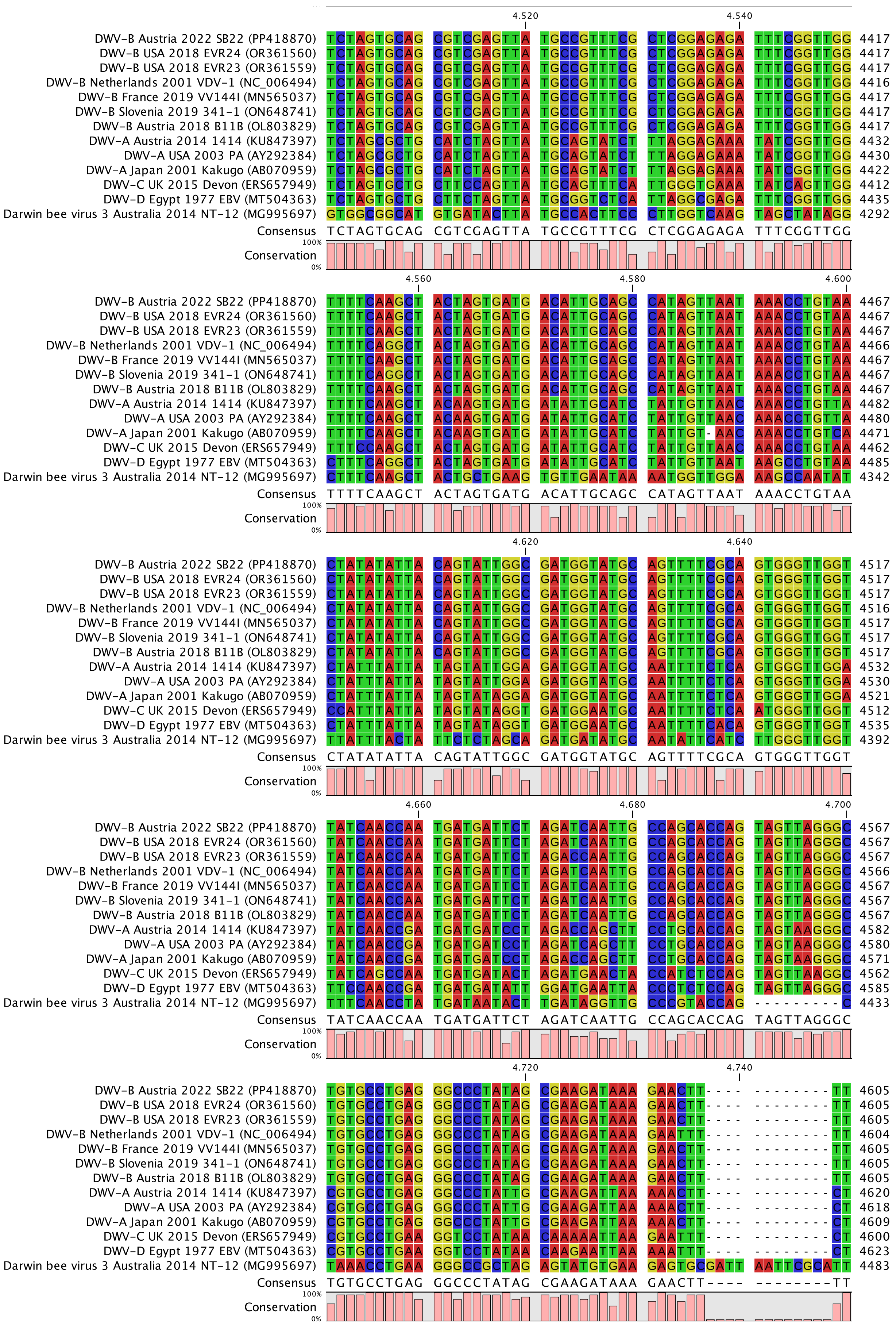
DWV-B Austria 2022 SB22 (PP418870)
DWV-B USA 2018 EVR24 (OR361560)
DWV-B USA 2018 EVR23 (OR361559)
DWV-B Netherlands 2001 VDV-1 (NC_006494)
DWV-B France 2019 VV1441 (MN565037)
DWV-B Slovenia 2019 341-1 (ON648741)
DWV-B Austria 2018 B11B (OL803829)
DWV-A Austria 2014 1414 (KU847397)
DWV-A USA 2003 PA (AY292384)
DWV-A Japan 2001 Kakugo (AB070959)
DWV-C UK 2015 Devon (ERS657949)
DWV-D Egypt 1977 EBV (MT504363)
Darwin bee virus 3 Australia 2014 NT-12 (MG995697)
Consensus
Conservation
100%
0%
4.520
4.540
TCTAGTGCAG CGTCGAGTTA TGCCGTTTCG CTCGGAGAGA TTTCGGTTGG
4.560
4.580
4.600
TTTTCAAGCT ACTAGTGATG ACATTGCAGC CATAGTTAAT AAACCTGTAA
4.620
4.640
CTATATATTA CAGTATTGGC GATGGTATGC AGTTTTCGCA GTGGGTTGGT
4.660
4.680
4.700
TATCAACCAA TGATGATTCT AGATCAATTG CCAGCACCAG TAGTTAGGGC
4.720
4.740
TGTGCCTGAG GGCCCTATAG CGAAGATAAA GAACTT---- --------TT

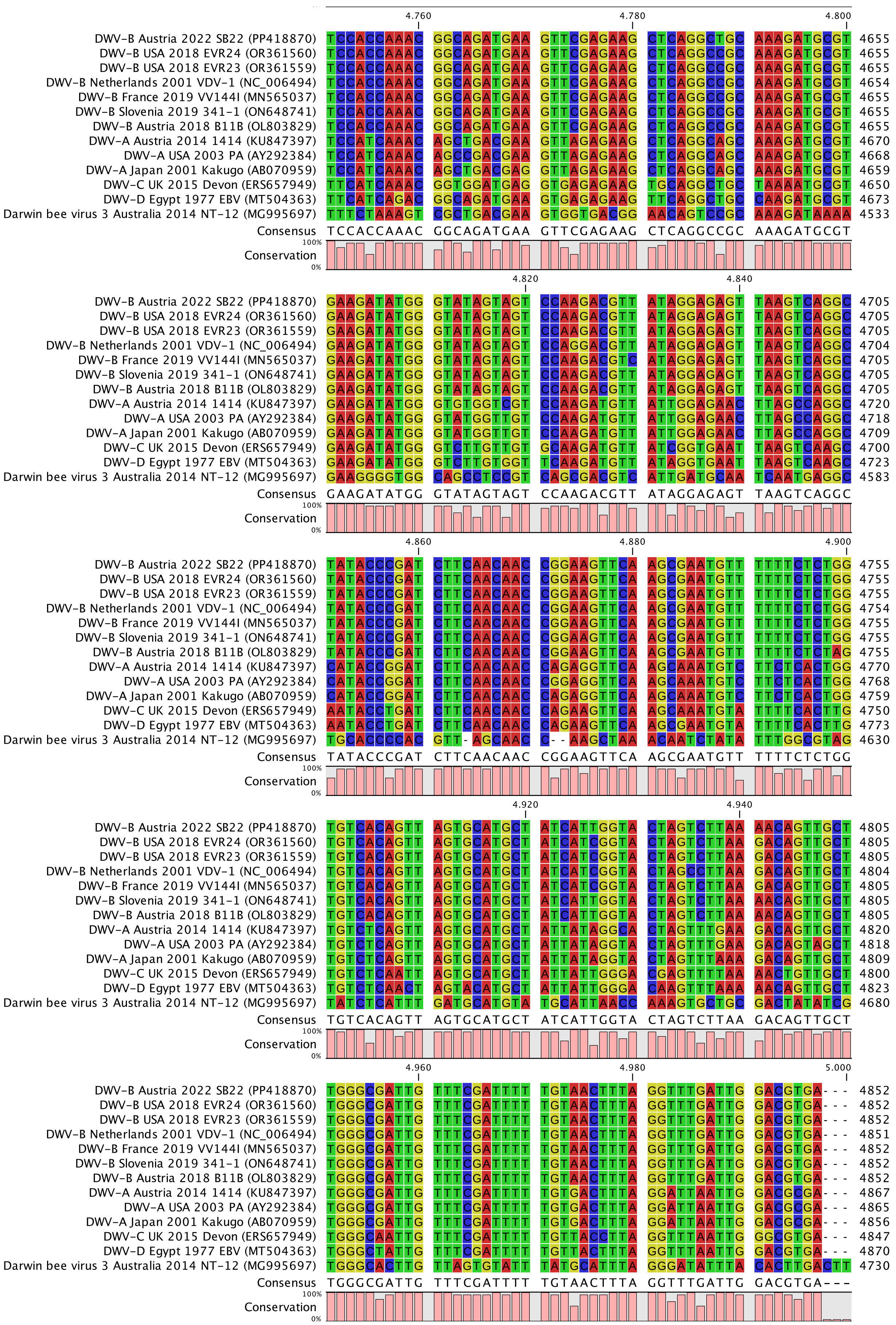
DWV-B Austria 2022 SB22 (PP418870)
DWV-B USA 2018 EVR24 (OR361560)
DWV-B USA 2018 EVR23 (OR361559)
DWV-B Netherlands 2001 VDV-1 (NC_006494)
DWV-B France 2019 VV144I (MN565037)
DWV-B Slovenia 2019 341-1 (ON648741)
DWV-B Austria 2018 B11B (OL803829)
DWV-A Austria 2014 1414 (KU847397)
DWV-A USA 2003 PA (AY292384)
DWV-A Japan 2001 Kakugo (AB070959)
DWV-C UK 2015 Devon (ERS657949)
DWV-D Egypt 1977 EBV (MT504363)
Darwin bee virus 3 Australia 2014 NT-12 (MG995697)
4.760
4.780
4.800
Consensus TCCACCAAAC GGCAGATGAA GTTCGAGAAG CTCAGGCCGC AAAGATGCGT
4.820
4.840
Consensus GAAGATATGG GTATAGTAGT CCAAGACGTT ATAGGAGAGT TAAGTCAGGC
4.860
4.880
4.900
Consensus TATACCCGAT CTTCAACAAC CGGAAGTTCA AGCGAATGTT TTTTCTCTGG
4.920
4.940
Consensus TGTCACAGTT AGTGCATGCT ATCATTGGTA CTAGTCTTAA GACAGTTGCT
4.960
4.980
5.000
Consensus TGGGCGATTG TTTCGATTTT TGTAACTTTA GGTTTGATTG GACGTGA---
Conservation
100%
0%

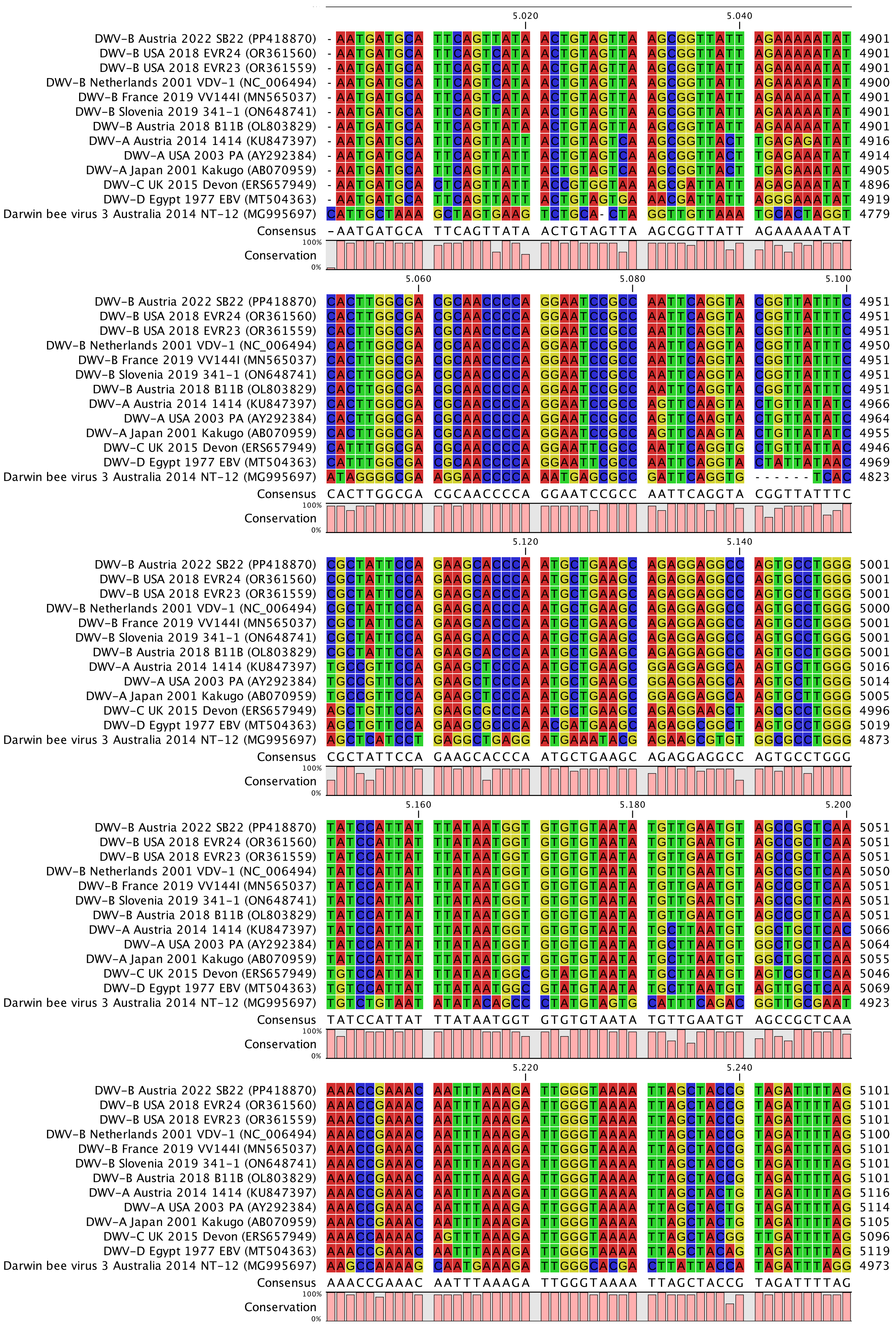
DWV-B Austria 2022 SB22 (PP418870)
DWV-B USA 2018 EVR24 (OR361560)
DWV-B USA 2018 EVR23 (OR361559)
DWV-B Netherlands 2001 VDV-1 (NC_006494)
DWV-B France 2019 VV144I (MN565037)
DWV-B Slovenia 2019 341-1 (ON648741)
DWV-B Austria 2018 B11B (OL803829)
DWV-A Austria 2014 1414 (KU847397)
DWV-A USA 2003 PA (AY292384)
DWV-A Japan 2001 Kakugo (AB070959)
DWV-C UK 2015 Devon (ERS657949)
DWV-D Egypt 1977 EBV (MT504363)
Darwin bee virus 3 Australia 2014 NT-12 (MG995697)
Consensus
Conservation
100%
0%
5.020
5.040
-AATGATGCA TTCAGTTATA ACTGTAGTTA AGCGGTTATT AGAAAAATAT
4901 4901 4901 4900 4901 4901 4901 4916 4914 4905 4896 4919 4779
5.060
5.080
5.100
CACTTGGCGA CGCAACCCCA GGAATCCGCC AATTCAGGTA CGGTTATTTC
4951 4951 4951 4950 4951 4951 4951 4966 4964 4955 4946 4969 4823
5.120
5.140
CGCTATTCCA GAAGCACCCA ATGCTGAAGC AGAGGAGGCC AGTGCCTGGG
5001 5001 5001 5000 5001 5001 5001 5016 5014 5005 4996 5019 4873
5.160
5.180
5.200
TATCCATTAT TTATAATGGT GTGTGTAATA TGTTGAATGT AGCCGCTCAA
5051 5051 5051 5050 5051 5051 5051 5066 5064 5055 5046 5069 4923
5.220
5.240
AAACCGAAAC AATTTAAAGA TTGGGTAAAA TTAGCTACCG TAGATTTTAG
5101 5101 5101 5100 5101 5101 5101 5116 5114 5105 5096 5119 4973

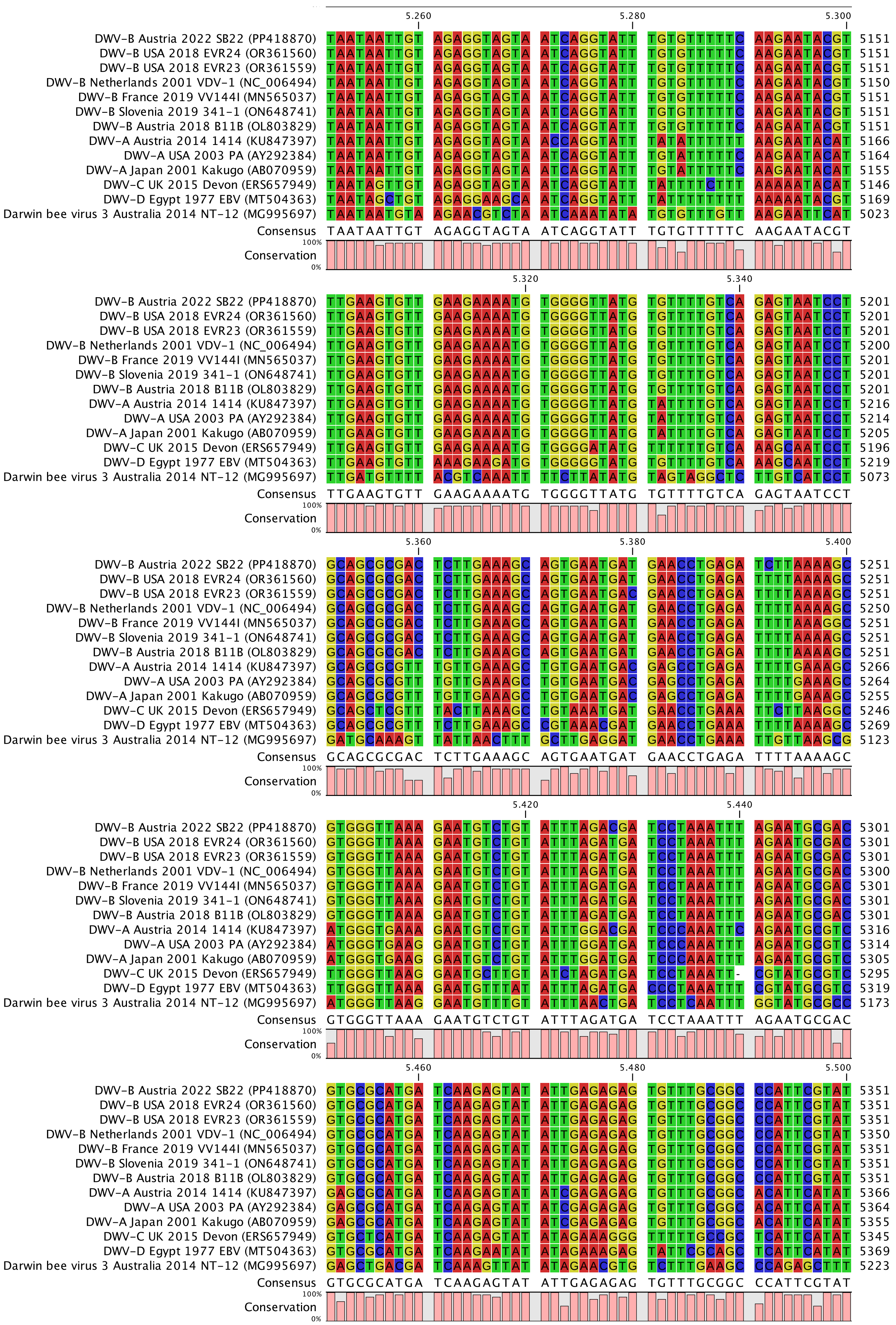
5.260
5.280
5.300
DWV-B Austria 2022 SB22 (PP418870)
DWV-B USA 2018 EVR24 (OR361560)
DWV-B USA 2018 EVR23 (OR361559)
DWV-B Netherlands 2001 VDV-1 (NC_006494)
DWV-B France 2019 VV1441 (MN565037)
DWV-B Slovenia 2019 341-1 (ON648741)
DWV-B Austria 2018 B11B (OL803829)
DWV-A Austria 2014 1414 (KU847397)
DWV-A USA 2003 PA (AY292384)
DWV-A Japan 2001 Kakugo (AB070959)
DWV-C UK 2015 Devon (ERS657949)
DWV-D Egypt 1977 EBV (MT504363)
Darwin bee virus 3 Australia 2014 NT-12 (MG995697)
Consensus TAATAATTGT AGAGGTAGTA ATCAGGTATT TGTGTTTTTC AAGAATACGT
Conservation
5.320
5.340
Consensus TTGAAGTGTT GAAGAAAATG TGGGGTTATG TGTTTTGTCA GAGTAATCCT
5.360
5.380
5.400
Consensus GCAGCGCGAC TCTTGAAAGC AGTGAATGAT GAACCTGAGA TTTTAAAAGC
5.420
5.440
Consensus GTGGGTTAAA GAATGTCTGT ATTTAGATGA TCCTAAATTT AGAATGCGAC
5.460
5.480
5.500
Consensus GTGCGCATGA TCAAGAGTAT ATTGAGAGAG TGTTTGCGGC CCATTCGTAT

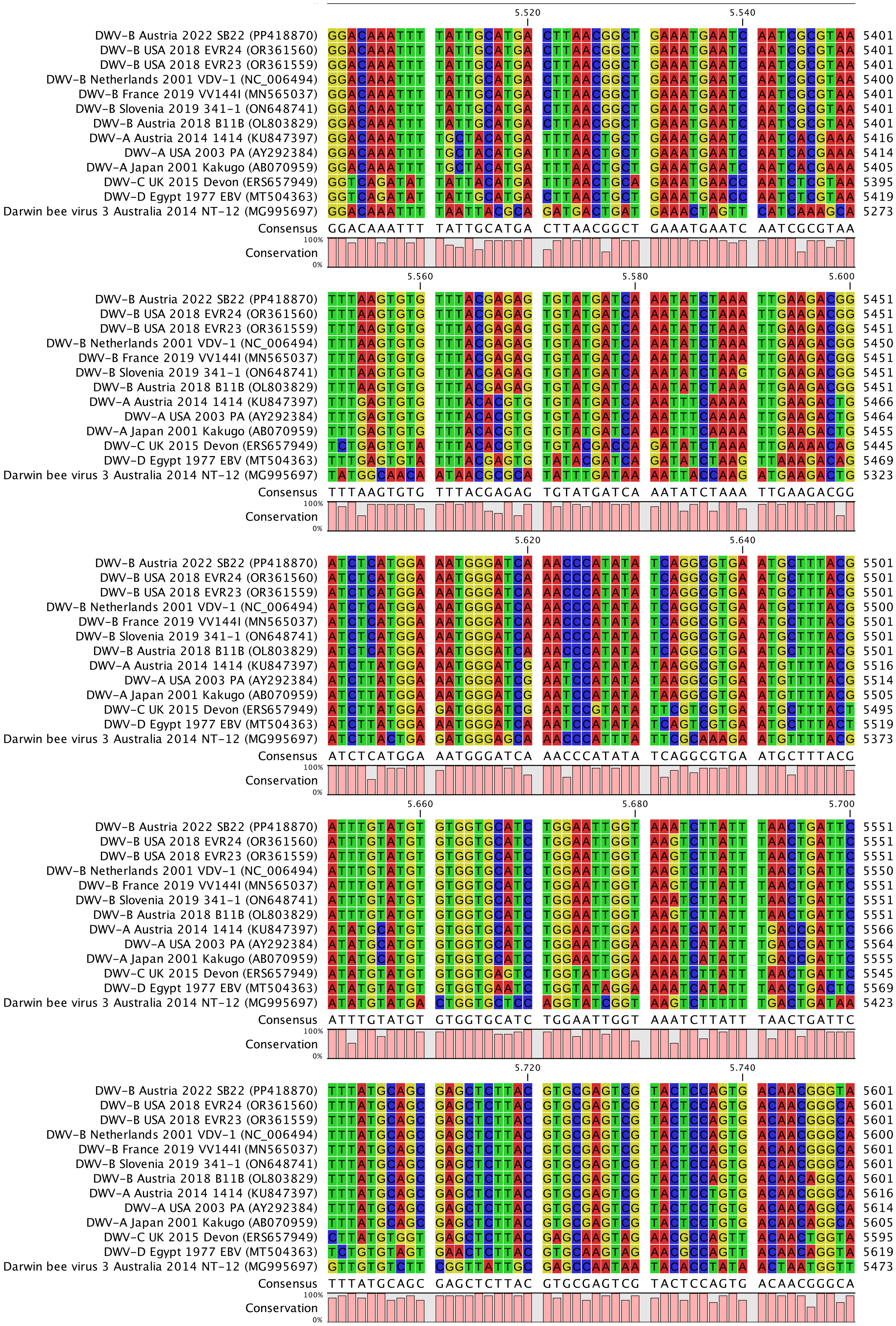
5.520 5.540
DWV-B Austria 2022 SB22 (PP418870) GGACAAATTT TATTGCATGA CTTAACGGCT GAAATGAATC AATCGCGTAA 5401
DWV-B USA 2018 EVR24 (OR361560) GGACAAATTT TATTGCATGA CTTAACGGCT GAAATGAATC AATCGCGTAA 5401
DWV-B USA 2018 EVR23 (OR361559) GGACAAATTT TATTGCATGA CTTAACGGCT GAAATGAATC AATCGCGTAA 5401
DWV-B Netherlands 2001 VDV-1 (NC_006494) GGACAAATTT TATTGCATGA CTTAACGGCT GAAATGAATC AATCGCGTAA 5400
DWV-B France 2019 VV144I (MN565037) GGACAAATTT TATTGCATGA CTTAACGGCT GAAATGAATC AATCGCGTAA 5401
DWV-B Slovenia 2019 341-1 (ON648741) GGACAAATTT TATTGCATGA CTTAACGGCT GAAATGAATC AATCGCGTAA 5401
DWV-B Austria 2018 B11B (OL803829) GGACAAATTT TATTGCATGA CTTAACGGCT GAAATGAATC AATCGCGTAA 5401
DWV-A Austria 2014 1414 (KU847397) GGACAAATTT TGCTACATGA TTTAACTGCT GAAATGAATC AATCACGAAA 5416
DWV-A USA 2003 PA (AY292384) GGACAAATTT TGCTACATGA TTTAACTGCT GAAATGAATC AATCACGAAA 5414
DWV-A Japan 2001 Kakugo (AB070959) GGACAAATTT TGCTACATGA TTTAACTGCT GAAATGAATC AATCACGAAA 5405
DWV-C UK 2015 Devon (ERS657949) GGTCAGATAT TATTACATGA TTTAACTGCA GAAATGAACC AATCTCGTAA 5395
DWV-D Egypt 1977 EBV (MT504363) GGTCAGATAT TATTGCATGA CTTAACTGCT GAAATGAACC AATCTCGTAA 5419
Darwin bee virus 3 Australia 2014 NT-12 (MG995697) GGACAAATTT TAATTACGCA GATGACTGAT GAAACTAGTT CATCAAAGCA 5273
Consensus GGACAAATTT TATTGCATGA CTTAACGGCT GAAATGAATC AATCGCGTAA
100% Conservation 0%
5.560 5.580 5.600
DWV-B Austria 2022 SB22 (PP418870) TTTAAGTGTG TTTACGAGAG TGTATGATCA AATATCTAAA TTGAAGACGG 5451
DWV-B USA 2018 EVR24 (OR361560) TTTAAGTGTG TTTACGAGAG TGTATGATCA AATATCTAAA TTGAAGACGG 5451
DWV-B USA 2018 EVR23 (OR361559) TTTAAGTGTG TTTACGAGAG TGTATGATCA AATATCTAAA TTGAAGACGG 5451
DWV-B Netherlands 2001 VDV-1 (NC_006494) TTTAAGTGTG TTTACGAGAG TGTATGATCA AATATCTAAA TTGAAGACGG 5450
DWV-B France 2019 VV144I (MN565037) TTTAAGTGTG TTTACGAGAG TGTATGATCA AATATCTAAA TTGAAGACGG 5451
DWV-B Slovenia 2019 341-1 (ON648741) TTTAAGTGTG TTTACGAGAG TGTATGATCA AATATCTAAG TTGAAGACGG 5451
DWV-B Austria 2018 B11B (OL803829) TTTAAGTGTG TTTACGAGAG TGTATGATCA AATATCTAAA TTGAAGACGG 5451
DWV-A Austria 2014 1414 (KU847397) TTTGAGTGTG TTTACACGTG TGTATGATCA AATTTCAAAA TTGAAGACTG 5466
DWV-A USA 2003 PA (AY292384) TTTGAGTGTG TTTACACGTG TGTATGATCA AATTTCAAAA TTGAAGACTG 5464
DWV-A Japan 2001 Kakugo (AB070959) TTTGAGTGTG TTTACACGTG TGTATGATCA AATTTCAAAA TTGAAGACTG 5455
DWV-C UK 2015 Devon (ERS657949) TCTGAGTGTA TTTACACGTG TGTACGACCA GATATCTAAA TTGAAAACAG 5445
DWV-D Egypt 1977 EBV (MT504363) TTTGAGTGTA TTTACGAGTG TATACGATCA GATATCTAAG TTAAAGACAG 5469
Darwin bee virus 3 Australia 2014 NT-12 (MG995697) TTTAAGTGTG TTTACGAGAG TGTATGATCA AATTACCAAG ATGAAGACTG 5323
Consensus TTTAAGTGTG TTTACGAGAG TGTATGATCA AATATCTAAA TTGAAGACGG
100% Conservation 0%
5.620 5.640
DWV-B Austria 2022 SB22 (PP418870) ATCTCATGGA AATGGGATCA AACCCATATA TCAGGCGTGA ATGCTTTACG 5501
DWV-B USA 2018 EVR24 (OR361560) ATCTCATGGA AATGGGATCA AACCCATATA TCAGGCGTGA ATGCTTTACG 5501
DWV-B USA 2018 EVR23 (OR361559) ATCTCATGGA AATGGGATCA AACCCATATA TCAGGCGTGA ATGCTTTACG 5501
DWV-B Netherlands 2001 VDV-1 (NC_006494) ATCTCATGGA AATGGGATCA AACCCATATA TCAGGCGTGA ATGCTTTACG 5500
DWV-B France 2019 VV144I (MN565037) ATCTCATGGA AATGGGATCA AACCCATATA TCAGGCGTGA ATGCTTTACG 5501
DWV-B Slovenia 2019 341-1 (ON648741) ATCTCATGGA AATGGGATCA AACCCATATA TCAGGCGTGA ATGCTTTACG 5501
DWV-B Austria 2018 B11B (OL803829) ATCTCATGGA AATGGGATCA AACCCATATA TCAGGCGTGA ATGCTTTACG 5501
DWV-A Austria 2014 1414 (KU847397) ATCTTATGGA AATGGGATCG AATCCATATA TAAGGCGTGA ATGTTTTACG 5516
DWV-A USA 2003 PA (AY292384) ATCTTATGGA AATGGGATCG AATCCATATA TAAGGCGTGA ATGTTTTACG 5514
DWV-A Japan 2001 Kakugo (AB070959) ATCTTATGGA AATGGGATCG AATCCATATA TAAGGCGTGA ATGTTTTACG 5505
DWV-C UK 2015 Devon (ERS657949) ATCTTATGGA GATGGGATCG AATCCGTATA TTCGTCGTGA ATGCTTTACT 5495
DWV-D Egypt 1977 EBV (MT504363) ATCTTATGGA AATGGGATCA AATCCATATA TCAGTCGTGA ATGCTTTACT 5519
Darwin bee virus 3 Australia 2014 NT-12 (MG995697) ATCTTACTGA GATGGGAGCA AACCCATTTA TTCGCAAAGA ATGTTTTACG 5373
Consensus ATCTCATGGA AATGGGATCA AACCCATATA TCAGGCGTGA ATGCTTTACG
100% Conservation 0%
5.660 5.680 5.700
DWV-B Austria 2022 SB22 (PP418870) ATTTGTATGT GTGGTGCATC TGGAATTGGT AAATCTTATT TAACTGATTC 5551
DWV-B USA 2018 EVR24 (OR361560) ATTTGTATGT GTGGTGCATC TGGAATTGGT AAGTCTTATT TAACTGATTC 5551
DWV-B USA 2018 EVR23 (OR361559) ATTTGTATGT GTGGTGCATC TGGAATTGGT AAGTCTTATT TAACTGATTC 5551
DWV-B Netherlands 2001 VDV-1 (NC_006494) ATTTGTATGT GTGGTGCATC TGGAATTGGT AAGTCTTATT TAACTGATTC 5550
DWV-B France 2019 VV144I (MN565037) ATTTGTATGT GTGGTGCATC TGGAATTGGT AAGTCTTATT TAACTGATTC 5551
DWV-B Slovenia 2019 341-1 (ON648741) ATTTGTATGT GTGGTGCATC TGGAATTGGT AAATCTTATT TAACTGATTC 5551
DWV-B Austria 2018 B11B (OL803829) ATTTGTATGT GTGGTGCATC TGGAATTGGT AAGTCTTATT TAACTGATTC 5551
DWV-A Austria 2014 1414 (KU847397) ATATGCATGT GTGGTGCATC TGGAATTGGA AAATCATATT TGACCGATTC 5566
DWV-A USA 2003 PA (AY292384) ATATGCATGT GTGGTGCATC TGGAATTGGA AAATCATATT TGACCGATTC 5564
DWV-A Japan 2001 Kakugo (AB070959) ATATGCATGT GTGGTGCATC TGGAATTGGA AAATCATATT TGACCGATTC 5555
DWV-C UK 2015 Devon (ERS657949) ATATGTATGT GTGGTGAGTC TGGTATTGGA AAATCTTATT TAACTGATTC 5545
DWV-D Egypt 1977 EBV (MT504363) ATATGTATGT GTGGTGAATC TGGTATAGGA AAATCATATT TAACTGACTC 5569
Darwin bee virus 3 Australia 2014 NT-12 (MG995697) ATATGTATGA CTGGTGCTCC AGGTATCGGT AAGTCTTTTT TGACTGATAA 5423
Consensus ATTTGTATGT GTGGTGCATC TGGAATTGGT AAATCTTATT TAACTGATTC
100% Conservation 0%
5.720 5.740
DWV-B Austria 2022 SB22 (PP418870) TTTATGCAGC GAGCTCTTAC GTGCGAGTCG TACTCCAGTG ACAACGGGTA 5601
DWV-B USA 2018 EVR24 (OR361560) TTTATGCAGC GAGCTCTTAC GTGCGAGTCG TACTCCAGTG ACAACGGGCA 5601
DWV-B USA 2018 EVR23 (OR361559) TTTATGCAGC GAGCTCTTAC GTGCGAGTCG TACTCCAGTG ACAACGGGCA 5601
DWV-B Netherlands 2001 VDV-1 (NC_006494) TTTATGCAGC GAGCTCTTAC GTGCGAGTCG TACTCCAGTG ACAACGGGCA 5600
DWV-B France 2019 VV144I (MN565037) TTTATGCAGC GAGCTCTTAC GTGCGAGTCG TACTCCAGTG ACAACGGGCA 5601
DWV-B Slovenia 2019 341-1 (ON648741) TTTATGCAGC GAGCTCTTAC GTGCGAGTCG TACTCCAGTG ACAACGGGCA 5601
DWV-B Austria 2018 B11B (OL803829) TTTATGCAGC GAGCTCTTAC GTGCGAGTCG TACTCCAGTG ACAACAGGCA 5601
DWV-A Austria 2014 1414 (KU847397) TTTATGCAGC GAGCTCTTAC GTGCGAGTCG TACTCCTGTG ACAACGGGCA 5616
DWV-A USA 2003 PA (AY292384) TTTATGCAGC GAGCTCTTAC GTGCGAGTCG TACTCCTGTG ACAACAGGCA 5614
DWV-A Japan 2001 Kakugo (AB070959) TTTATGCAGC GAGCTCTTAC GTGCGAGTCG TACTCCTGTG ACAACAGGCA 5605
DWV-C UK 2015 Devon (ERS657949) CTTATGTGGT GAGCTCTTAC GAGCAAGTAG AACGCCAGTT ACAACTGGTA 5595
DWV-D Egypt 1977 EBV (MT504363) TCTGTGTAGT GAACTCTTAC GTGCAAGTAG AACGCCAGTT ACAACAGGTA 5619
Darwin bee virus 3 Australia 2014 NT-12 (MG995697) GTTGTGTCTT CGGTTATTGC GAGCCAATAA TACACCTATA ACTAATGGTT 5473
Consensus TTTATGCAGC GAGCTCTTAC GTGCGAGTCG TACTCCAGTG ACAACGGGCA
100% Conservation 0%

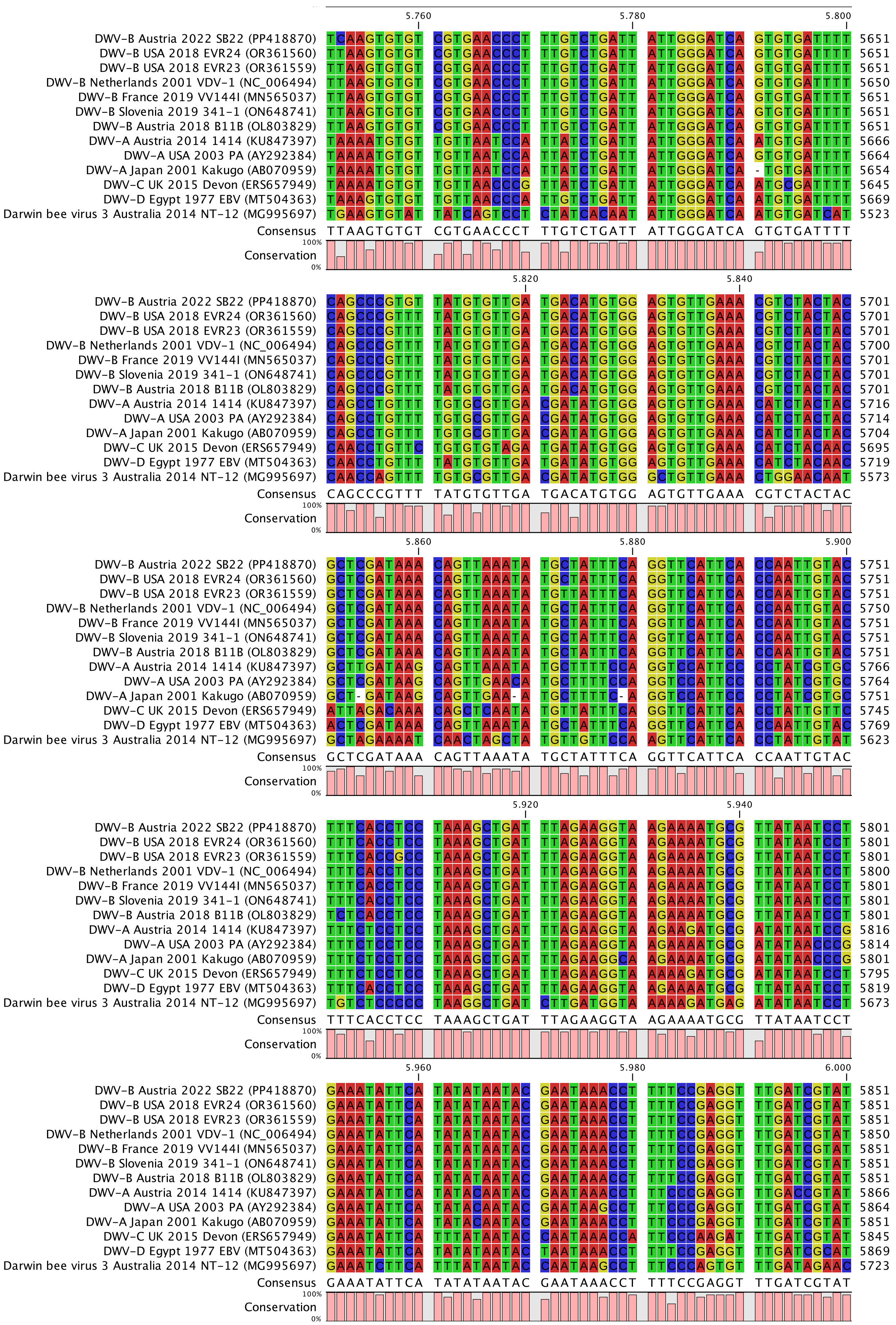
5.760
5.780
5.800
5.820
5.840
5.860
5.880
5.900
5.920
5.940
5.960
5.980
6.000
DWV-B Austria 2022 SB22 (PP418870)
DWV-B USA 2018 EVR24 (OR361560)
DWV-B USA 2018 EVR23 (OR361559)
DWV-B Netherlands 2001 VDV-1 (NC_006494)
DWV-B France 2019 VV1441 (MN565037)
DWV-B Slovenia 2019 341-1 (ON648741)
DWV-B Austria 2018 B11B (OL803829)
DWV-A Austria 2014 1414 (KU847397)
DWV-A USA 2003 PA (AY292384)
DWV-A Japan 2001 Kakugo (AB070959)
DWV-C UK 2015 Devon (ERS657949)
DWV-D Egypt 1977 EBV (MT504363)
Darwin bee virus 3 Australia 2014 NT-12 (MG995697)
Consensus TTAAGTGTGT CGTGAACCCT TTGTCTGATT ATTGGGATCA GTGTGATTTT
Consensus CAGCCCGTTT TATGTGTTGA TGACATGTGG AGTGTTGAAA CGTCTACTAC
Consensus GCTCGATAAA CAGTTAAATA TGCTATTTCA GGTTCATTCA CCAATTGTAC
Consensus TTTCACCTCC TAAAGCTGAT TTAGAAGGTA AGAAAATGCG TTATAATCCT
Consensus GAAATATTCA TATATAATAC GAATAAACCT TTTCCGAGGT TTGATCGTAT
Conservation
100%
0%

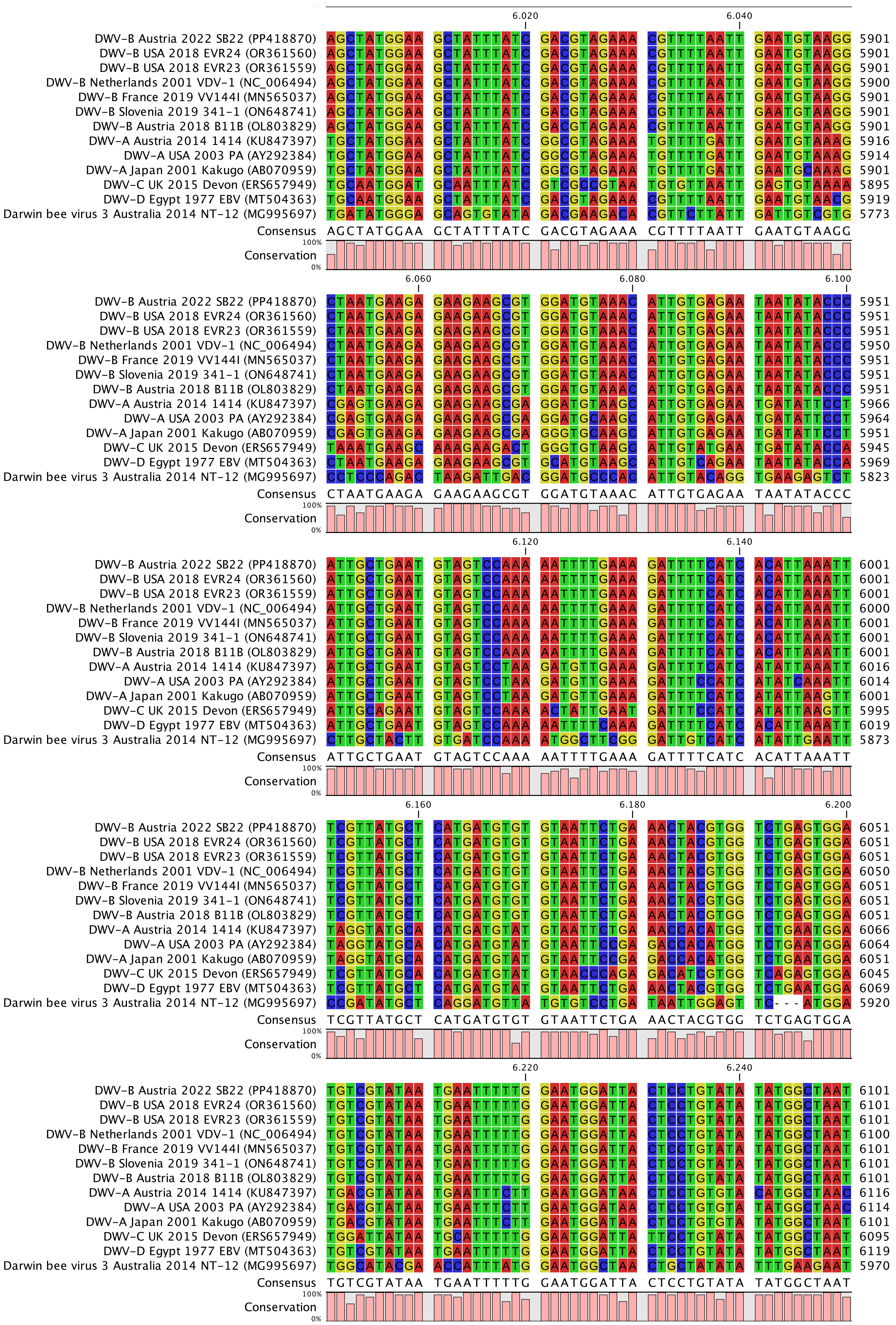
6.020 6.040
DWV-B Austria 2022 SB22 (PP418870) AGCTATGGAA GCTATTTATC GACGTAGAAA CGTTTTAATT GAATGTAAGG 5901
DWV-B USA 2018 EVR24 (OR361560) AGCTATGGAA GCTATTTATC GACGTAGAAA CGTTTTAATT GAATGTAAGG 5901
DWV-B USA 2018 EVR23 (OR361559) AGCTATGGAA GCTATTTATC GACGTAGAAA CGTTTTAATT GAATGTAAGG 5901
DWV-B Netherlands 2001 VDV-1 (NC_006494) AGCTATGGAA GCTATTTATC GACGTAGAAA CGTTTTAATT GAATGTAAGG 5900
DWV-B France 2019 VV1441 (MN565037) AGCTATGGAA GCTATTTATC GACGTAGAAA CGTTTTAATT GAATGTAAGG 5901
DWV-B Slovenia 2019 341-1 (ON648741) AGCTATGGAA GCTATTTATC GACGTAGAAA CGTTTTAATT GAATGTAAGG 5901
DWV-B Austria 2018 B11B (OL803829) AGCTATGGAA GCTATTTATC GACGTAGAAA CGTTTTAATT GAATGTAAGG 5901
DWV-A Austria 2014 1414 (KU847397) TGCTATGGAA GCTATTTATC GGCGTAGAAA TGTTTTGATT GAATGTAAAG 5916
DWV-A USA 2003 PA (AY292384) TGCTATGGAA GCTATTTATC GGCGTAGAAA TGTTTTGATT GAATGTAAAG 5914
DWV-A Japan 2001 Kakugo (AB070959) TGCTATGGAA GCTATTTATC GGCGTAGAAA TGTTTTGATT GAATGCAAAG 5901
DWV-C UK 2015 Devon (ERS657949) TGCAATGGAT GCAATTTATC GTCGCCGTAA TGTGTTAATT GAGTGTAAAA 5895
DWV-D Egypt 1977 EBV (MT504363) TGCAATGGAA GCTATTTATC GACGTAGAAA CGTTTTAATT GAATGTAACG 5919
Darwin bee virus 3 Australia 2014 NT-12 (MG995697) TGATATGGGA GCAGTGTATA GACGAAGACA CGTTCTTATT GATTGTCGTG 5773
Consensus AGCTATGGAA GCTATTTATC GACGTAGAAA CGTTTTAATT GAATGTAAGG
100%
Conservation
0%
6.060 6.080 6.100
DWV-B Austria 2022 SB22 (PP418870) CTAATGAAGA GAAGAAGCGT GGATGTAAAC ATTGTGAGAA TAATATACCC 5951
DWV-B USA 2018 EVR24 (OR361560) CTAATGAAGA GAAGAAGCGT GGATGTAAAC ATTGTGAGAA TAATATACCC 5951
DWV-B USA 2018 EVR23 (OR361559) CTAATGAAGA GAAGAAGCGT GGATGTAAAC ATTGTGAGAA TAATATACCC 5951
DWV-B Netherlands 2001 VDV-1 (NC_006494) CTAATGAAGA GAAGAAGCGT GGATGTAAAC ATTGTGAGAA TAATATACCC 5950
DWV-B France 2019 VV1441 (MN565037) CTAATGAAGA GAAGAAGCGT GGATGTAAAC ATTGTGAGAA TAATATACCC 5951
DWV-B Slovenia 2019 341-1 (ON648741) CTAATGAAGA GAAGAAGCGT GGATGTAAAC ATTGTGAGAA TAATATACCC 5951
DWV-B Austria 2018 B11B (OL803829) CTAATGAAGA GAAGAAGCGT GGATGTAAAC ATTGTGAGAA TAATATACCC 5951
DWV-A Austria 2014 1414 (KU847397) CGAGTGAAGA GAAGAAGCGA GGATGTAAGC ATTGTGAGAA TGATATTCCT 5966
DWV-A USA 2003 PA (AY292384) CGAGTGAAGA GAAGAAGCGA GGATGCAAGC ATTGTGAGAA TGATATTCCT 5964
DWV-A Japan 2001 Kakugo (AB070959) CGAGTGAAGA GAAGAAGCGA GGGTGCAAGC ATTGTGAGAA TGATATTCCT 5951
DWV-C UK 2015 Devon (ERS657949) TAAATGAAGC AAAGAAGACT GGGTGTAAGC ATTGTATGAA TGATATACCA 5945
DWV-D Egypt 1977 EBV (MT504363) CTAATGAAGA GAAGAAGCGT GCATGTAAGC ATTGTCAGAA TAATATACCA 5969
Darwin bee virus 3 Australia 2014 NT-12 (MG995697) CCTCCCAGAC TAAGATTGAC GGATGCCCAC ATTGTACAGG TGAAGAGTCT 5823
Consensus CTAATGAAGA GAAGAAGCGT GGATGTAAAC ATTGTGAGAA TAATATACCC
100%
Conservation
0%
6.120 6.140
DWV-B Austria 2022 SB22 (PP418870) ATTGCTGAAT GTAGTCCAAA AATTTTGAAA GATTTTCATC ACATTAAATT 6001
DWV-B USA 2018 EVR24 (OR361560) ATTGCTGAAT GTAGTCCAAA AATTTTGAAA GATTTTCATC ACATTAAATT 6001
DWV-B USA 2018 EVR23 (OR361559) ATTGCTGAAT GTAGTCCAAA AATTTTGAAA GATTTTCATC ACATTAAATT 6001
DWV-B Netherlands 2001 VDV-1 (NC_006494) ATTGCTGAAT GTAGTCCAAA AATTTTGAAA GATTTTCATC ACATTAAATT 6000
DWV-B France 2019 VV1441 (MN565037) ATTGCTGAAT GTAGTCCAAA AATTTTGAAA GATTTTCATC ACATTAAATT 6001
DWV-B Slovenia 2019 341-1 (ON648741) ATTGCTGAAT GTAGTCCAAA AATTTTGAAA GATTTTCATC ACATTAAATT 6001
DWV-B Austria 2018 B11B (OL803829) ATTGCTGAAT GTAGTCCAAA AATTTTGAAA GATTTTCATC ACATTAAATT 6001
DWV-A Austria 2014 1414 (KU847397) ATTGCTGAAT GTAGTCCTAA GATGTTGAAA GATTTTCATC ATATTAAATT 6016
DWV-A USA 2003 PA (AY292384) ATTGCTGAAT GTAGTCCTAA GATGTTGAAA GATTTCCATC ATATCAAATT 6014
DWV-A Japan 2001 Kakugo (AB070959) ATTGCTGAAT GTAGTCCTAA GATGTTGAAA GATTTTCATC ATATTAAGTT 6001
DWV-C UK 2015 Devon (ERS657949) ATTGCAGAAT GTAGTCCAAA ACTATTGAAT GATTTCCATC ATATTAAGTT 5995
DWV-D Egypt 1977 EBV (MT504363) ATTGCTGAAT GTAGTCCAAA AATTTTCAAA GATTTTCATC ACATTAAATT 6019
Darwin bee virus 3 Australia 2014 NT-12 (MG995697) CTTGCTACTT GTGATCCAAA ATGGCTTCGG GATTGTCATC ATATTGAATT 5873
Consensus ATTGCTGAAT GTAGTCCAAA AATTTTGAAA GATTTTCATC ACATTAAATT
100%
Conservation
0%
6.160 6.180 6.200
DWV-B Austria 2022 SB22 (PP418870) TCGTTATGCT CATGATGTGT GTAATTCTGA AACTACGTGG TCTGAGTGGA 6051
DWV-B USA 2018 EVR24 (OR361560) TCGTTATGCT CATGATGTGT GTAATTCTGA AACTACGTGG TCTGAGTGGA 6051
DWV-B USA 2018 EVR23 (OR361559) TCGTTATGCT CATGATGTGT GTAATTCTGA AACTACGTGG TCTGAGTGGA 6051
DWV-B Netherlands 2001 VDV-1 (NC_006494) TCGTTATGCT CATGATGTGT GTAATTCTGA AACTACGTGG TCTGAGTGGA 6050
DWV-B France 2019 VV1441 (MN565037) TCGTTATGCT CATGATGTGT GTAATTCTGA AACTACGTGG TCTGAGTGGA 6051
DWV-B Slovenia 2019 341-1 (ON648741) TCGTTATGCT CATGATGTGT GTAATTCTGA AACTACGTGG TCTGAGTGGA 6051
DWV-B Austria 2018 B11B (OL803829) TCGTTATGCT CATGATGTGT GTAATTCTGA AACTACGTGG TCTGAGTGGA 6051
DWV-A Austria 2014 1414 (KU847397) TAGGTATGCA CATGATGTAT GTAATTCTGA AACCACATGG TCTGAATGGA 6066
DWV-A USA 2003 PA (AY292384) TAGGTATGCA CATGATGTAT GTAATTCCGA GACCACATGG TCTGAATGGA 6064
DWV-A Japan 2001 Kakugo (AB070959) TAGGTATGCA CATGATGTAT GTAATTCCGA GACCACATGG TCTGAATGGA 6051
DWV-C UK 2015 Devon (ERS657949) TCGTTATGCA CATGATGTAT GTAACCCAGA GACATCGTGG TCAGAGTGGA 6045
DWV-D Egypt 1977 EBV (MT504363) TCGTTATGCT CATGATGTAT GTAATTCTGA AACTACGTGG TCTGAATGGA 6069
Darwin bee virus 3 Australia 2014 NT-12 (MG995697) CCGATATGCT CAGGATGTTA TGTGTCCTGA TAATTGGAGT TC---ATGGA 5920
Consensus TCGTTATGCT CATGATGTGT GTAATTCTGA AACTACGTGG TCTGAGTGGA
100%
Conservation
0%
6.220 6.240
DWV-B Austria 2022 SB22 (PP418870) TGTCGTATAA TGAATTTTTG GAATGGATTA CTCCTGTATA TATGGCTAAT 6101
DWV-B USA 2018 EVR24 (OR361560) TGTCGTATAA TGAATTTTTG GAATGGATTA CTCCTGTATA TATGGCTAAT 6101
DWV-B USA 2018 EVR23 (OR361559) TGTCGTATAA TGAATTTTTG GAATGGATTA CTCCTGTATA TATGGCTAAT 6101
DWV-B Netherlands 2001 VDV-1 (NC_006494) TGTCGTATAA TGAATTTTTG GAATGGATTA CTCCTGTATA TATGGCTAAT 6100
DWV-B France 2019 VV1441 (MN565037) TGTCGTATAA TGAATTTTTG GAATGGATTA CTCCTGTATA TATGGCTAAT 6101
DWV-B Slovenia 2019 341-1 (ON648741) TGTCGTATAA TGAATTTTTG GAATGGATTA CTCCTGTATA TATGGCTAAT 6101
DWV-B Austria 2018 B11B (OL803829) TGTCGTATAA TGAATTTTTG GAATGGATTA CTCCTGTATA TATGGCTAAT 6101
DWV-A Austria 2014 1414 (KU847397) TGACGTATAA TGAATTTCTT GAATGGATAA CTCCTGTGTA CATGGCTAAC 6116
DWV-A USA 2003 PA (AY292384) TGACGTATAA TGAATTTCTT GAATGGATAA CTCCTGTGTA TATGGCTAAC 6114
DWV-A Japan 2001 Kakugo (AB070959) TGACGTATAA TGAATTTCTT GAATGGATAA CTCCTGTGTA TATGGCTAAT 6101
DWV-C UK 2015 Devon (ERS657949) TGGATTATAA TGCATTTTTG GAATGGATTA TTCCTGTATA TATGGCTAAT 6095
DWV-D Egypt 1977 EBV (MT504363) TGTCGTATAA TGAATTTTTG GAATGGATTA CTCCTGTATA TATGGCTAAT 6119
Darwin bee virus 3 Australia 2014 NT-12 (MG995697) TGGCATACGA ACCATTTATG GAATGGCTAA CTGCTATATA TTTGAAGAAT 5970
Consensus TGTCGTATAA TGAATTTTTG GAATGGATTA CTCCTGTATA TATGGCTAAT
100%
Conservation
0%

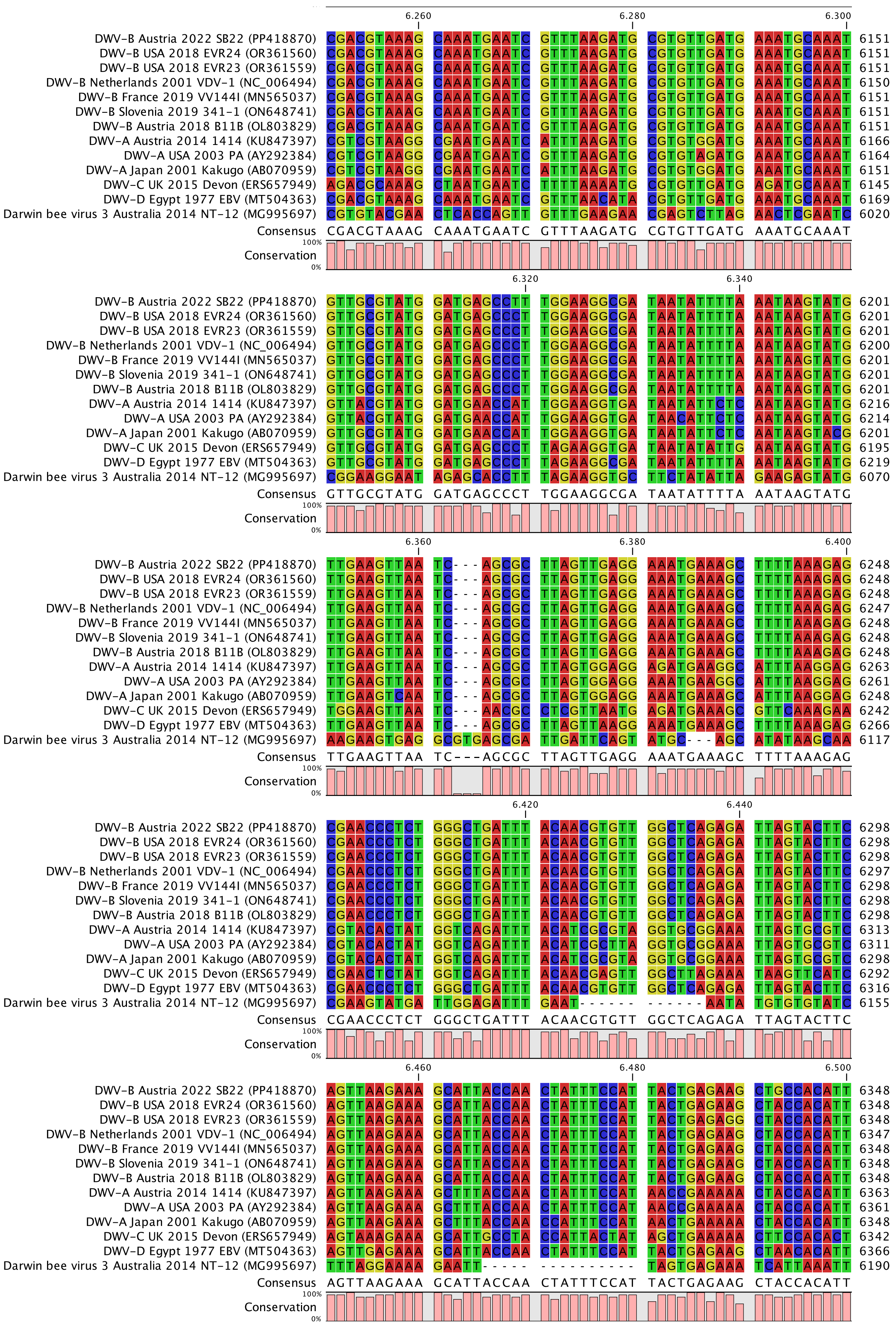
DWV-B Austria 2022 SB22 (PP418870)
DWV-B USA 2018 EVR24 (OR361560)
DWV-B USA 2018 EVR23 (OR361559)
DWV-B Netherlands 2001 VDV-1 (NC_006494)
DWV-B France 2019 VV1441 (MN565037)
DWV-B Slovenia 2019 341-1 (ON648741)
DWV-B Austria 2018 B11B (OL803829)
DWV-A Austria 2014 1414 (KU847397)
DWV-A USA 2003 PA (AY292384)
DWV-A Japan 2001 Kakugo (AB070959)
DWV-C UK 2015 Devon (ERS657949)
DWV-D Egypt 1977 EBV (MT504363)
Darwin bee virus 3 Australia 2014 NT-12 (MG995697)
Consensus
Conservation
100%
0%
6.260
6.280
6.300
CGACGTAAAG CAAATGAATC GTTTAAGATG CGTGTTGATG AAATGCAAAT
6.320
6.340
GTTGCGTATG GATGAGCCCT TGGAAGGCGA TAATATTTTA AATAAGTATG
6.360
6.380
6.400
TTGAAGTTAA TC---AGCGC TTAGTTGAGG AAATGAAAGC TTTTAAAGAG
6.420
6.440
CGAACCCTCT GGGCTGATTT ACAACGTGTT GGCTCAGAGA TTAGTACTTC
6.460
6.480
6.500
AGTTAAGAAA GCATTACCAA CTATTTCCAT TACTGAGAAG CTACCACATT

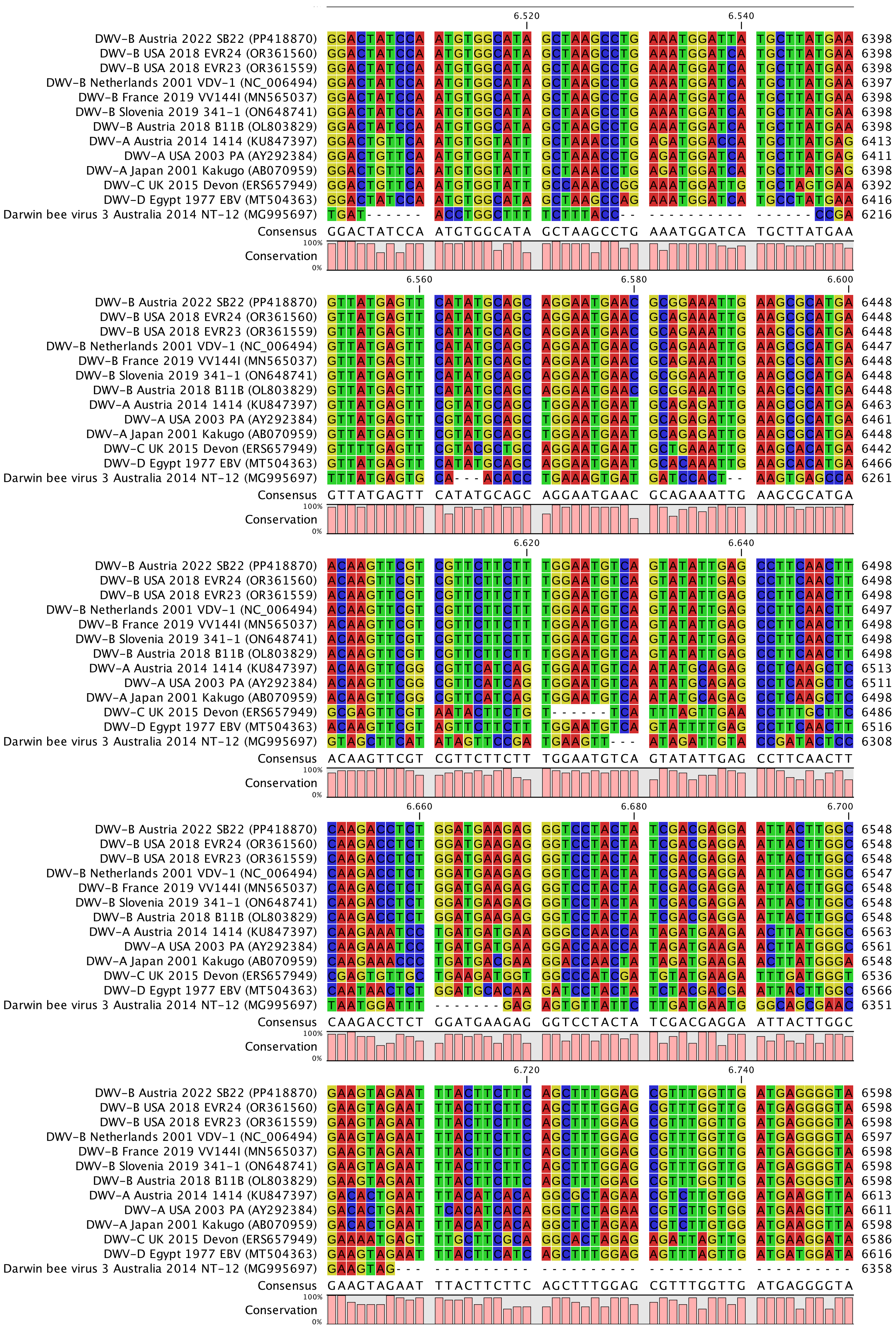
6.520
6.540
DWV-B Austria 2022 SB22 (PP418870) GGACTATCCA ATGTGGCATA GCTAAGCCTG AAATGGATTA TGCTTATGAA 6398
DWV-B USA 2018 EVR24 (OR361560) GGACTATCCA ATGTGGCATA GCTAAGCCTG AAATGGATCA TGCTTATGAA 6398
DWV-B USA 2018 EVR23 (OR361559) GGACTATCCA ATGTGGCATA GCTAAGCCTG AAATGGATCA TGCTTATGAA 6398
DWV-B Netherlands 2001 VDV-1 (NC_006494) GGACTATCCA ATGTGGCATA GCTAAGCCTG AAATGGATCA TGCTTATGAA 6397
DWV-B France 2019 VV1441 (MN565037) GGACTATCCA ATGTGGCATA GCTAAGCCTG AAATGGATCA TGCTTATGAA 6398
DWV-B Slovenia 2019 341-1 (ON648741) GGACTATCCA ATGTGGCATA GCTAAGCCTG AAATGGATCA TGCTTATGAA 6398
DWV-B Austria 2018 B11B (OL803829) GGACTATCCA ATGTGGCATA GCTAAGCCTG AAATGGATCA TGCTTATGAA 6398
DWV-A Austria 2014 1414 (KU847397) GGACTGTTCA ATGTGGTATT GCTAAACCTG AGATGGACCA TGCTTATGAG 6413
DWV-A USA 2003 PA (AY292384) GGACTGTTCA ATGTGGTATT GCTAAACCTG AGATGGATCA TGCTTATGAG 6411
DWV-A Japan 2001 Kakugo (AB070959) GGACTGTTCA ATGTGGTATT GCTAAACCTG AGATGGATCA TGCTTATGAG 6398
DWV-C UK 2015 Devon (ERS657949) GGACTGTTCA ATGTGGTATT GCCAAACCGG AAATGGATTG TGCTAGTGAA 6392
DWV-D Egypt 1977 EBV (MT504363) GGACTATCCA ATGTGGCATA GCTAAGCCAG AAATGGATCA TGCCTATGAA 6416
Darwin bee virus 3 Australia 2014 NT-12 (MG995697) TGAT------ ACCTGGCTTT TCTTTACC-- ---------- ------CCGA 6216
Consensus GGACTATCCA ATGTGGCATA GCTAAGCCTG AAATGGATCA TGCTTATGAA
100%
Conservation
0%
6.560
6.580
6.600
DWV-B Austria 2022 SB22 (PP418870) GTTATGAGTT CATATGCAGC AGGAATGAAC GCGGAAATTG AAGCGCATGA 6448
DWV-B USA 2018 EVR24 (OR361560) GTTATGAGTT CATATGCAGC AGGAATGAAC GCAGAAATTG AAGCGCATGA 6448
DWV-B USA 2018 EVR23 (OR361559) GTTATGAGTT CATATGCAGC AGGAATGAAC GCAGAAATTG AAGCGCATGA 6448
DWV-B Netherlands 2001 VDV-1 (NC_006494) GTTATGAGTT CATATGCAGC AGGAATGAAC GCAGAAATTG AAGCGCATGA 6447
DWV-B France 2019 VV1441 (MN565037) GTTATGAGTT CATATGCAGC AGGAATGAAC GCAGAAATTG AAGCGCATGA 6448
DWV-B Slovenia 2019 341-1 (ON648741) GTTATGAGTT CATATGCAGC AGGAATGAAC GCGGAAATTG AAGCGCATGA 6448
DWV-B Austria 2018 B11B (OL803829) GTTATGAGTT CATATGCAGC AGGAATGAAC GCGGAAATTG AAGCGCATGA 6448
DWV-A Austria 2014 1414 (KU847397) GTTATGAGTT CGTATGCAGC TGGAATGAAT GCAGAGATTG AAGCGCATGA 6463
DWV-A USA 2003 PA (AY292384) GTTATGAGTT CGTATGCAGC TGGAATGAAT GCAGAGATTG AAGCGCATGA 6461
DWV-A Japan 2001 Kakugo (AB070959) GTTATGAGTT CGTATGCAGC TGGAATGAAT GCAGAGATTG AAGCGCATGA 6448
DWV-C UK 2015 Devon (ERS657949) GTTTTGAGTT CGTACGCTGC AGGAATGAAT GCTGAAATTG AAGCACATGA 6442
DWV-D Egypt 1977 EBV (MT504363) GTTATGAGTT CATATGCAGC AGGAATGAAT GCACAAATTG AAGCACATGA 6466
Darwin bee virus 3 Australia 2014 NT-12 (MG995697) TTTATGAGTG CA---ACACC TGAAAGTGAT GATCCACT-- AAGTGAGCCA 6261
Consensus GTTATGAGTT CATATGCAGC AGGAATGAAC GCAGAAATTG AAGCGCATGA
100%
Conservation
0%
6.620
6.640
DWV-B Austria 2022 SB22 (PP418870) ACAAGTTCGT CGTTCTTCTT TGGAATGTCA GTATATTGAG CCTTCAACTT 6498
DWV-B USA 2018 EVR24 (OR361560) ACAAGTTCGT CGTTCTTCTT TGGAATGTCA GTATATTGAG CCTTCAACTT 6498
DWV-B USA 2018 EVR23 (OR361559) ACAAGTTCGT CGTTCTTCTT TGGAATGTCA GTATATTGAG CCTTCAACTT 6498
DWV-B Netherlands 2001 VDV-1 (NC_006494) ACAAGTTCGT CGTTCTTCTT TGGAATGTCA GTATATTGAG CCTTCAACTT 6497
DWV-B France 2019 VV1441 (MN565037) ACAAGTTCGT CGTTCTTCTT TGGAATGTCA GTATATTGAG CCTTCAACTT 6498
DWV-B Slovenia 2019 341-1 (ON648741) ACAAGTTCGT CGTTCTTCTT TGGAATGTCA GTATATTGAG CCTTCAACTT 6498
DWV-B Austria 2018 B11B (OL803829) ACAAGTTCGT CGTTCTTCTT TGGAATGTCA GTATATTGAG CCTTCAACTT 6498
DWV-A Austria 2014 1414 (KU847397) ACAAGTTCGG CGTTCATCAG TGGAATGTCA ATATGCAGAG CCTCAAGCTC 6513
DWV-A USA 2003 PA (AY292384) ACAAGTTCGG CGTTCATCAG TGGAATGTCA ATATGCAGAG CCTCAAGCTC 6511
DWV-A Japan 2001 Kakugo (AB070959) ACAAGTTCGG CGTTCATCAG TGGAATGTCA ATATGCAGAG CCTCAAGCTC 6498
DWV-C UK 2015 Devon (ERS657949) GCGAGTTCGT AATACTTCTG T------TCA TTTAGTTGAA CCTTTGCTTC 6486
DWV-D Egypt 1977 EBV (MT504363) ACAAGTTCGT AGTTCTTCTT TGGAATGTCA GTATTTTGAG CCTTCAACTT 6516
Darwin bee virus 3 Australia 2014 NT-12 (MG995697) GTAGCTTCAT ATAGTTCCGA TGAAGTT--- ATAGATTGTA CCGATACTCC 6308
Consensus ACAAGTTCGT CGTTCTTCTT TGGAATGTCA GTATATTGAG CCTTCAACTT
100%
Conservation
0%
6.660
6.680
6.700
DWV-B Austria 2022 SB22 (PP418870) CAAGACCTCT GGATGAAGAG GGTCCTACTA TCGACGAGGA ATTACTTGGC 6548
DWV-B USA 2018 EVR24 (OR361560) CAAGACCTCT GGATGAAGAG GGTCCTACTA TCGACGAGGA ATTACTTGGC 6548
DWV-B USA 2018 EVR23 (OR361559) CAAGACCTCT GGATGAAGAG GGTCCTACTA TCGACGAGGA ATTACTTGGC 6548
DWV-B Netherlands 2001 VDV-1 (NC_006494) CAAGACCTCT GGATGAAGAG GGTCCTACTA TCGACGAGGA ATTACTTGGC 6547
DWV-B France 2019 VV1441 (MN565037) CAAGACCTCT GGATGAAGAG GGTCCTACTA TCGACGAGGA ATTACTTGGC 6548
DWV-B Slovenia 2019 341-1 (ON648741) CAAGACCTCT GGATGAAGAG GGTCCTACTA TCGACGAGGA ATTACTTGGC 6548
DWV-B Austria 2018 B11B (OL803829) CAAGACCTCT GGATGAAGAG GGTCCTACTA TCGACGAGGA ATTACTTGGC 6548
DWV-A Austria 2014 1414 (KU847397) CAAGAAATCC TGATGATGAA GGGCCAACCA TAGATGAAGA ACTTATGGGC 6563
DWV-A USA 2003 PA (AY292384) CAAGAAATCC TGATGATGAA GGACCAACCA TAGATGAAGA ACTTATGGGC 6561
DWV-A Japan 2001 Kakugo (AB070959) CAAGAAACCC TGATGACGAA GGACCAACTA TAGATGAAGA ACTTATGGGC 6548
DWV-C UK 2015 Devon (ERS657949) CGAGTGTTGC TGAAGATGGT GGCCCATCGA TGTATGAAGA TTTGATGGGT 6536
DWV-D Egypt 1977 EBV (MT504363) CAATAACTCT GGATGCACAA GATCCTACTA TCTACGACGA ATTACTTGGC 6566
Darwin bee virus 3 Australia 2014 NT-12 (MG995697) TAATGGATTT -------GAG AGTGTTATTC TTGATGAATG GGCAGCGAAC 6351
Consensus CAAGACCTCT GGATGAAGAG GGTCCTACTA TCGACGAGGA ATTACTTGGC
100%
Conservation
0%
6.720
6.740
DWV-B Austria 2022 SB22 (PP418870) GAAGTAGAAT TTACTTCTTC AGCTTTGGAG CGTTTGGTTG ATGAGGGGTA 6598
DWV-B USA 2018 EVR24 (OR361560) GAAGTAGAAT TTACTTCTTC AGCTTTGGAG CGTTTGGTTG ATGAGGGGTA 6598
DWV-B USA 2018 EVR23 (OR361559) GAAGTAGAAT TTACTTCTTC AGCTTTGGAG CGTTTGGTTG ATGAGGGGTA 6598
DWV-B Netherlands 2001 VDV-1 (NC_006494) GAAGTAGAAT TTACTTCTTC AGCTTTGGAG CGTTTGGTTG ATGAGGGGTA 6597
DWV-B France 2019 VV1441 (MN565037) GAAGTAGAAT TTACTTCTTC AGCTTTGGAG CGTTTGGTTG ATGAGGGGTA 6598
DWV-B Slovenia 2019 341-1 (ON648741) GAAGTAGAAT TTACTTCTTC AGCTTTGGAG CGTTTGGTTG ATGAGGGGTA 6598
DWV-B Austria 2018 B11B (OL803829) GAAGTAGAAT TTACTTCTTC AGCTTTGGAG CGTTTGGTTG ATGAGGGGTA 6598
DWV-A Austria 2014 1414 (KU847397) GACACTGAAT TTACATCACA GGCGCTAGAA CGTCTTGTGG ATGAAGGTTA 6613
DWV-A USA 2003 PA (AY292384) GACACTGAAT TCACATCACA GGCTCTAGAA CGTCTTGTGG ATGAAGGTTA 6611
DWV-A Japan 2001 Kakugo (AB070959) GACACTGAAT TTACATCACA GGCTCTAGAA CGTCTTGTGG ATGAAGGTTA 6598
DWV-C UK 2015 Devon (ERS657949) GAAAATGAGT TTGCTTCGCA GGCACTAGAG AGATTAGTTG ATGAAGGATA 6586
DWV-D Egypt 1977 EBV (MT504363) GAAGTAGAAT TTACTTCATC AGCTTTGGAG AGTTTAGTTG ATGATGGATA 6616
Darwin bee virus 3 Australia 2014 NT-12 (MG995697) GAAGTAG--- ---------- ---------- ---------- ---------- 6358
Consensus GAAGTAGAAT TTACTTCTTC AGCTTTGGAG CGTTTGGTTG ATGAGGGGTA
100%
Conservation
0%

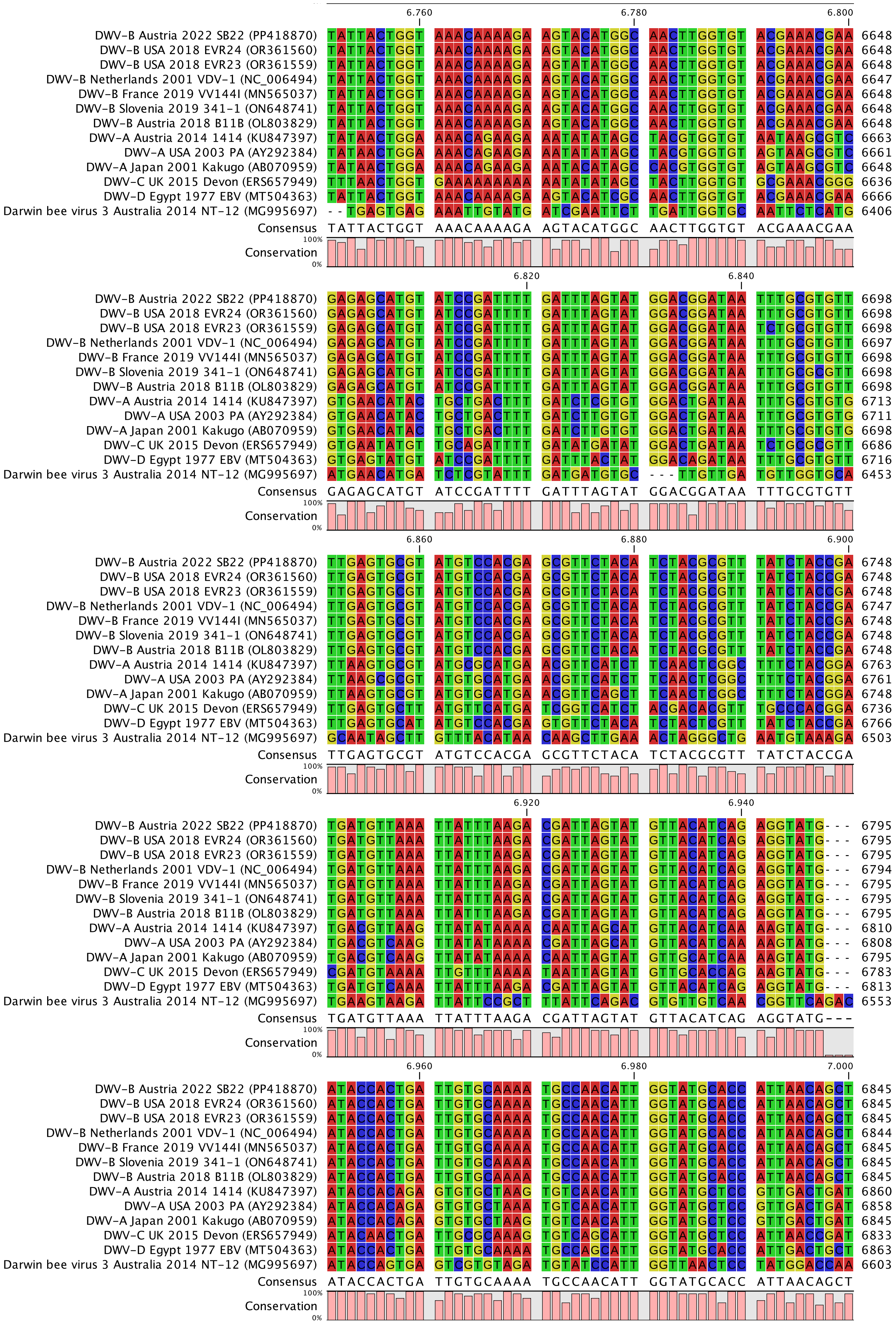
6.760 6.780 6.800
DWV-B Austria 2022 SB22 (PP418870) TATTACTGGT AAACAAAAGA AGTACATGGC AACTTGGTGT ACGAAACGAA 6648
DWV-B USA 2018 EVR24 (OR361560) TATTACTGGT AAACAAAAGA AGTACATGGC AACTTGGTGT ACGAAACGAA 6648
DWV-B USA 2018 EVR23 (OR361559) TATTACTGGT AAACAAAAGA AGTATATGGC AACTTGGTGT ACGAAACGAA 6648
DWV-B Netherlands 2001 VDV-1 (NC_006494) TATTACTGGT AAACAAAAGA AGTACATGGC AACTTGGTGT ACGAAACGAA 6647
DWV-B France 2019 VV144I (MN565037) TATTACTGGT AAACAAAAGA AGTACATGGC AACTTGGTGT ACGAAACGAA 6648
DWV-B Slovenia 2019 341-1 (ON648741) TATTACTGGT AAACAAAAGA AGTACATGGC AACTTGGTGT ACGAAACGAA 6648
DWV-B Austria 2018 B11B (OL803829) TATTACTGGT AAACAAAAGA AGTACATGGC AACTTGGTGT ACGAAACGAA 6648
DWV-A Austria 2014 1414 (KU847397) TATAACTGGA AAACAGAAGA AATATATAGC TACGTGGTGT AATAAGCGTC 6663
DWV-A USA 2003 PA (AY292384) TATAACTGGA AAACAGAAGA AATATATAGC TACGTGGTGT AGTAAGCGTC 6661
DWV-A Japan 2001 Kakugo (AB070959) TATAACTGGA AAACAGAAGA AATACATAGC CACGTGGTGT AGTAAGCGTC 6648
DWV-C UK 2015 Devon (ERS657949) TTTAACTGGT GAAAAAAAAA AATATATAGC TACTTGGTGT GCGAAACGGG 6636
DWV-D Egypt 1977 EBV (MT504363) TATTACTGGT AAACAAAAGA AGTACATCGC AACTTGGTGT ACGAAACGAA 6666
Darwin bee virus 3 Australia 2014 NT-12 (MG995697) - - TGAGTGAG AAATTGTATG ATCGAATTCT TGATTGGTGC AATTCTCATG 6406
Consensus TATTACTGGT AAACAAAAGA AGTACATGGC AACTTGGTGT ACGAAACGAA
100%
Conservation
0%
6.820 6.840
DWV-B Austria 2022 SB22 (PP418870) GAGAGCATGT ATCCGATTTT GATTTAGTAT GGACGGATAA TTTGCGTGTT 6698
DWV-B USA 2018 EVR24 (OR361560) GAGAGCATGT ATCCGATTTT GATTTAGTAT GGACGGATAA TTTGCGTGTT 6698
DWV-B USA 2018 EVR23 (OR361559) GAGAGCATGT ATCCGATTTT GATTTAGTAT GGACGGATAA TCTGCGTGTT 6698
DWV-B Netherlands 2001 VDV-1 (NC_006494) GAGAGCATGT ATCCGATTTT GATTTAGTAT GGACGGATAA TTTGCGTGTT 6697
DWV-B France 2019 VV144I (MN565037) GAGAGCATGT ATCCGATTTT GATTTAGTAT GGACGGATAA TTTGCGTGTT 6698
DWV-B Slovenia 2019 341-1 (ON648741) GAGAGCATGT ATCCGATTTT GATTTAGTAT GGACGGATAA TTTGCGCGTT 6698
DWV-B Austria 2018 B11B (OL803829) GAGAGCATGT ATCCGATTTT GATTTAGTAT GGACGGATAA TTTGCGTGTT 6698
DWV-A Austria 2014 1414 (KU847397) GTGAACATAC TGCTGACTTT GATCTCGTGT GGACTGATAA TTTGCGTGTG 6713
DWV-A USA 2003 PA (AY292384) GTGAACATAC TGCTGACTTT GATCTTGTGT GGACTGATAA TTTGCGTGTG 6711
DWV-A Japan 2001 Kakugo (AB070959) GTGAACATAC TGCTGACTTT GATCTTGTGT GGACTGATAA TTTGCGTGTG 6698
DWV-C UK 2015 Devon (ERS657949) GTGAATATGT TGCAGATTTT GATATGATAT GGACTGATAA TCTGCGCGTT 6686
DWV-D Egypt 1977 EBV (MT504363) GTGAGTATGT ATCCGATTTT GATTTACTAT GGACAGATAA TTTGCGTGTT 6716
Darwin bee virus 3 Australia 2014 NT-12 (MG995697) ATGAACATGA TCTCGTATTT GATGATGTGC - - - TTGTTGA TGTTGGTGCA 6453
Consensus GAGAGCATGT ATCCGATTTT GATTTAGTAT GGACGGATAA TTTGCGTGTT
100%
Conservation
0%
6.860 6.880 6.900
DWV-B Austria 2022 SB22 (PP418870) TTGAGTGCGT ATGTCCACGA GCGTTCTACA TCTACGCGTT TATCTACCGA 6748
DWV-B USA 2018 EVR24 (OR361560) TTGAGTGCGT ATGTCCACGA GCGTTCTACA TCTACGCGTT TATCTACCGA 6748
DWV-B USA 2018 EVR23 (OR361559) TTGAGTGCGT ATGTCCACGA GCGTTCTACA TCTACGCGTT TATCTACCGA 6748
DWV-B Netherlands 2001 VDV-1 (NC_006494) TTGAGTGCGT ATGTCCACGA GCGTTCTACA TCTACGCGTT TATCTACCGA 6747
DWV-B France 2019 VV144I (MN565037) TTGAGTGCGT ATGTCCACGA GCGTTCTACA TCTACGCGTT TATCTACCGA 6748
DWV-B Slovenia 2019 341-1 (ON648741) TTGAGTGCGT ATGTCCACGA GCGTTCTACA TCTACGCGTT TATCTACCGA 6748
DWV-B Austria 2018 B11B (OL803829) TTGAGTGCGT ATGTCCACGA GCGTTCTACA TCTACGCGTT TATCTACCGA 6748
DWV-A Austria 2014 1414 (KU847397) TTAAGTGCGT ATGCGCATGA ACGTTCATCT TCAACTCGGC TTTCTACGGA 6763
DWV-A USA 2003 PA (AY292384) TTAAGCGCGT ATGTGCATGA ACGTTCATCT TCAACTCGGC TTTCTACGGA 6761
DWV-A Japan 2001 Kakugo (AB070959) TTAAGTGCGT ATGTGCATGA ACGTTCAGCT TCAACTCGGC TTTCTACGGA 6748
DWV-C UK 2015 Devon (ERS657949) TTGAGTGCTT ATGTTCATGA TCGGTCATCT ACGACACGTT TGCCCACGGA 6736
DWV-D Egypt 1977 EBV (MT504363) TTGAGTGCAT ATGTCCACGA GTGTTCTACA TGTACTCGTT TATCTACCGA 6766
Darwin bee virus 3 Australia 2014 NT-12 (MG995697) GCAATAGCTT GTTTACATAA CAAGCTTGAA ACTAGGGCTG AATGTAAAGA 6503
Consensus TTGAGTGCGT ATGTCCACGA GCGTTCTACA TCTACGCGTT TATCTACCGA
100%
Conservation
0%
6.920 6.940
DWV-B Austria 2022 SB22 (PP418870) TGATGTTAAA TTATTTAAGA CGATTAGTAT GTTACATCAG AGGTATG- - - 6795
DWV-B USA 2018 EVR24 (OR361560) TGATGTTAAA TTATTTAAGA CGATTAGTAT GTTACATCAG AGGTATG- - - 6795
DWV-B USA 2018 EVR23 (OR361559) TGATGTTAAA TTATTTAAGA CGATTAGTAT GTTACATCAG AGGTATG- - - 6795
DWV-B Netherlands 2001 VDV-1 (NC_006494) TGATGTTAAA TTATTTAAGA CGATTAGTAT GTTACATCAG AGGTATG- - - 6794
DWV-B France 2019 VV144I (MN565037) TGATGTTAAA TTATTTAAGA CGATTAGTAT GTTACATCAG AGGTATG- - - 6795
DWV-B Slovenia 2019 341-1 (ON648741) TGATGTTAAA TTATTTAAGA CGATTAGTAT GTTACATCAG AGGTATG- - - 6795
DWV-B Austria 2018 B11B (OL803829) TGATGTTAAA TTATTTAAGA CGATTAGTAT GTTACATCAG AGGTATG- - - 6795
DWV-A Austria 2014 1414 (KU847397) TGACGTTAAG TTATATAAAA CAATTAGCAT GTTACATCAA AAGTATG- - - 6810
DWV-A USA 2003 PA (AY292384) TGACGTCAAG TTATATAAAA CGATTAGCAT GTTACATCAA AAGTATG- - - 6808
DWV-A Japan 2001 Kakugo (AB070959) TGACGTCAAG TTATATAAAA CAATTAGTAT GTTGCATCAA AAGTATG- - - 6795
DWV-C UK 2015 Devon (ERS657949) CGATGTAAAA TTGTTTAAAA TAATTAGTAT GTTGCACCAG AAGTATG- - - 6783
DWV-D Egypt 1977 EBV (MT504363) TGATGTCAAA TTATTTAAGA CGATTAGTAT GTTACATCAG AGGTATG- - - 6813
Darwin bee virus 3 Australia 2014 NT-12 (MG995697) TGAAGTAAGA TTATTCCGCT TTATTCAGAC GTGTTGTCAA CGGTTCAGAC 6553
Consensus TGATGTTAAA TTATTTAAGA CGATTAGTAT GTTACATCAG AGGTATG- - -
100%
Conservation
0%
6.960 6.980 7.000
DWV-B Austria 2022 SB22 (PP418870) ATACCACTGA TTGTGCAAAA TGCCAACATT GGTATGCACC ATTAACAGCT 6845
DWV-B USA 2018 EVR24 (OR361560) ATACCACTGA TTGTGCAAAA TGCCAACATT GGTATGCACC ATTAACAGCT 6845
DWV-B USA 2018 EVR23 (OR361559) ATACCACTGA TTGTGCAAAA TGCCAACATT GGTATGCACC ATTAACAGCT 6845
DWV-B Netherlands 2001 VDV-1 (NC_006494) ATACCACTGA TTGTGCAAAA TGCCAACATT GGTATGCACC ATTAACAGCT 6844
DWV-B France 2019 VV144I (MN565037) ATACCACTGA TTGTGCAAAA TGCCAACATT GGTATGCACC ATTAACAGCT 6845
DWV-B Slovenia 2019 341-1 (ON648741) ATACCACTGA TTGTGCAAAA TGCCAACATT GGTATGCACC ATTAACAGCT 6845
DWV-B Austria 2018 B11B (OL803829) ATACCACTGA TTGTGCAAAA TGCCAACATT GGTATGCACC ATTAACAGCT 6845
DWV-A Austria 2014 1414 (KU847397) ATACCACAGA GTGTGCTAAG TGTCAACATT GGTATGCTCC GTTGACTGAT 6860
DWV-A USA 2003 PA (AY292384) ATACCACAGA GTGTGCTAAA TGTCAACATT GGTATGCTCC GTTGACTGAT 6858
DWV-A Japan 2001 Kakugo (AB070959) ATACCACAGA GTGTGCTAAG TGTCAACATT GGTATGCTCC GTTGACTGAT 6845
DWV-C UK 2015 Devon (ERS657949) ATACAACTGA TTGCGCAAAG TGTCAGCATT GGTATGCTCC ATTAACCGAT 6833
DWV-D Egypt 1977 EBV (MT504363) ATACCACTGA TTGTGCAAAA TGCCAGCATT GGTATGCACC ATTGACTGCT 6863
Darwin bee virus 3 Australia 2014 NT-12 (MG995697) ATACCAGTGA GTCGTGTAGA TGTATCCATT GGTTAACTCC TATGGACCAA 6603
Consensus ATACCACTGA TTGTGCAAAA TGCCAACATT GGTATGCACC ATTAACAGCT
100%
Conservation
0%

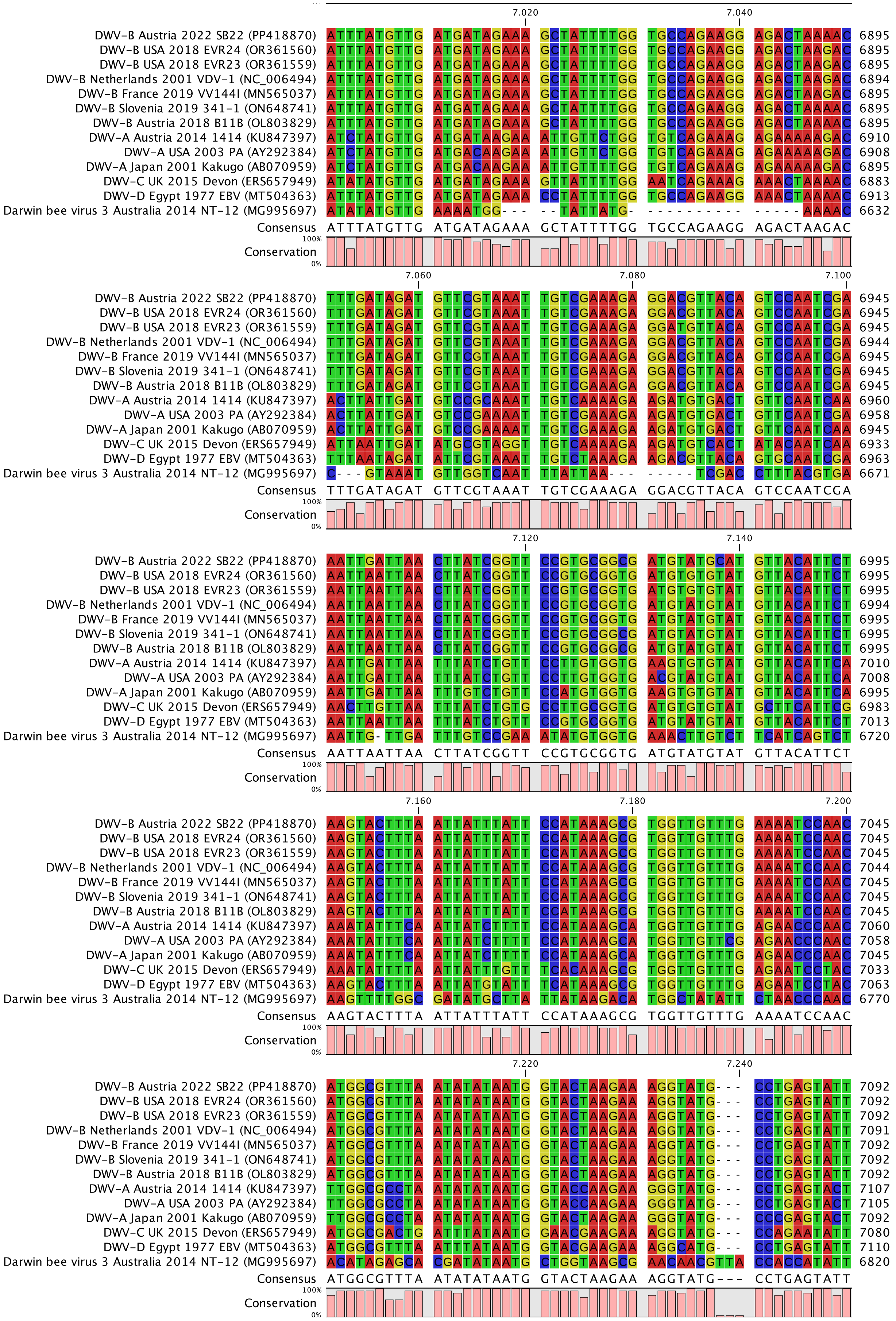

7.020
7.040
DWV-B Austria 2022 SB22 (PP418870)
DWV-B USA 2018 EVR24 (OR361560)
DWV-B USA 2018 EVR23 (OR361559)
DWV-B Netherlands 2001 VDV-1 (NC_006494)
DWV-B France 2019 VV1441 (MN565037)
DWV-B Slovenia 2019 341-1 (ON648741)
DWV-B Austria 2018 B11B (OL803829)
DWV-A Austria 2014 1414 (KU847397)
DWV-A USA 2003 PA (AY292384)
DWV-A Japan 2001 Kakugo (AB070959)
DWV-C UK 2015 Devon (ERS657949)
DWV-D Egypt 1977 EBV (MT504363)
Darwin bee virus 3 Australia 2014 NT-12 (MG995697)
Consensus ATTTATGTTG ATGATAGAAA GCTATTTTGG TGCCAGAAGG AGACTAAGAC
Conservation
7.060
7.080
7.100
Consensus TTTGATAGAT GTTCGTAAAT TGTCGAAAGA GGACGTTACA GTCCAATCGA
7.120
7.140
Consensus AATTAATTAA CTTATCGGTT CCGTGCGGTG ATGTATGTAT GTTACATTCT
7.160
7.180
7.200
Consensus AAGTACTTTA ATTATTTATT CCATAAAGCG TGGTTGTTTG AAAATCCAAC
7.220
7.240
Consensus ATGGCGTTTA ATATATAATG GTACTAAGAA AGGTATG--- CCTGAGTATT

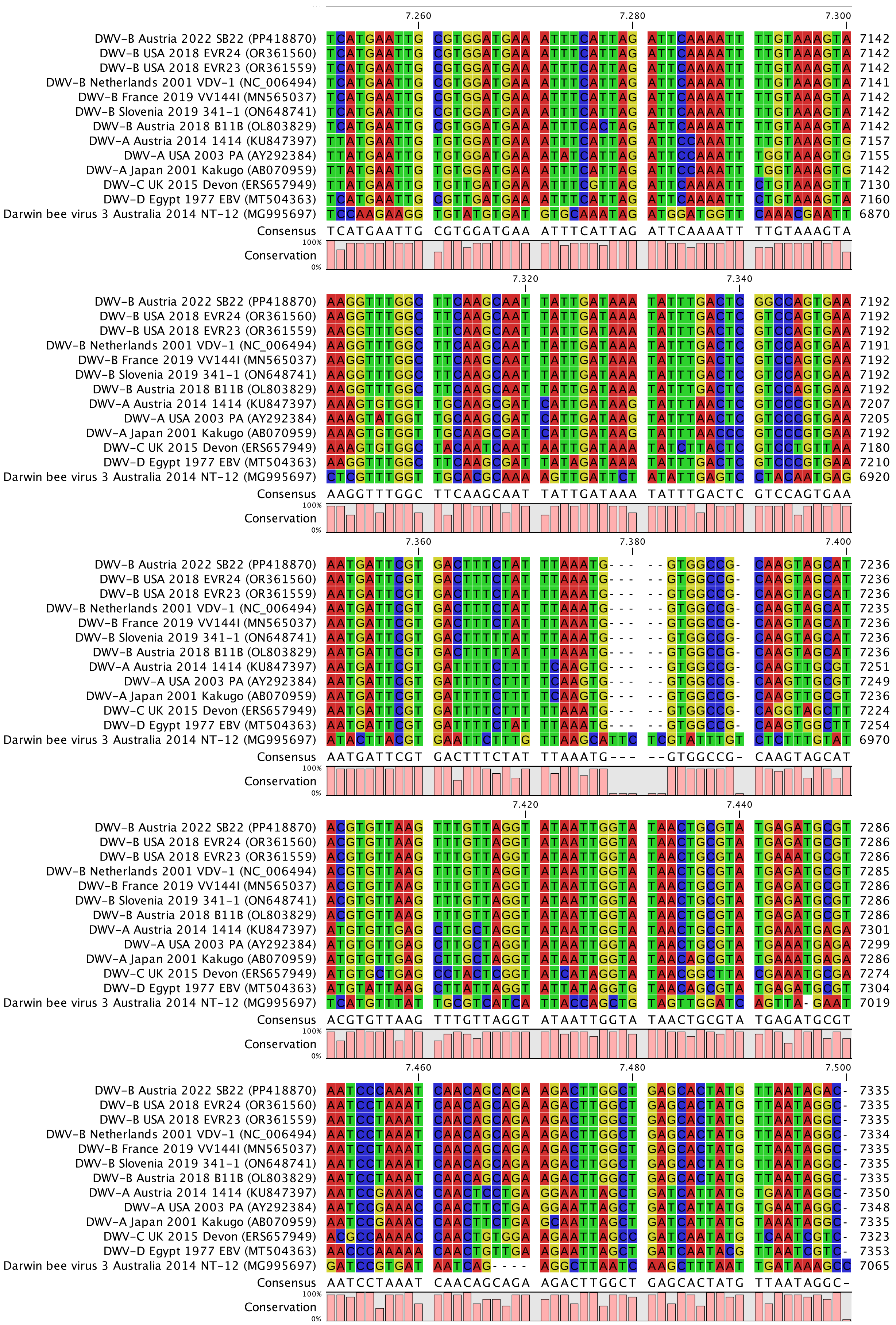
7.260 7.280 7.300
DWV-B Austria 2022 SB22 (PP418870)
DWV-B USA 2018 EVR24 (OR361560)
DWV-B USA 2018 EVR23 (OR361559)
DWV-B Netherlands 2001 VDV-1 (NC_006494)
DWV-B France 2019 VV144I (MN565037)
DWV-B Slovenia 2019 341-1 (ON648741)
DWV-B Austria 2018 B11B (OL803829)
DWV-A Austria 2014 1414 (KU847397)
DWV-A USA 2003 PA (AY292384)
DWV-A Japan 2001 Kakugo (AB070959)
DWV-C UK 2015 Devon (ERS657949)
DWV-D Egypt 1977 EBV (MT504363)
Darwin bee virus 3 Australia 2014 NT-12 (MG995697)
Consensus TCATGAATTG CGTGGATGAA ATTTCATTAG ATTCAAAATT TTGTAAAGTA
Conservation 100% 0%
7.320 7.340
Consensus AAGGTTTGGC TTCAAGCAAT TATTGATAAA TATTTGACTC GTCCAGTGAA
7.360 7.380 7.400
Consensus AATGATTCGT GACTTTCTAT TTAAATG--- --GTGGCCG- CAAGTAGCAT
7.420 7.440
Consensus ACGTGTTAAG TTTGTTAGGT ATAATTGGTA TAACTGCGTA TGAGATGCGT
7.460 7.480 7.500
Consensus AATCCTAAAT CAACAGCAGA AGACTTGGCT GAGCACTATG TTAATAGGC-

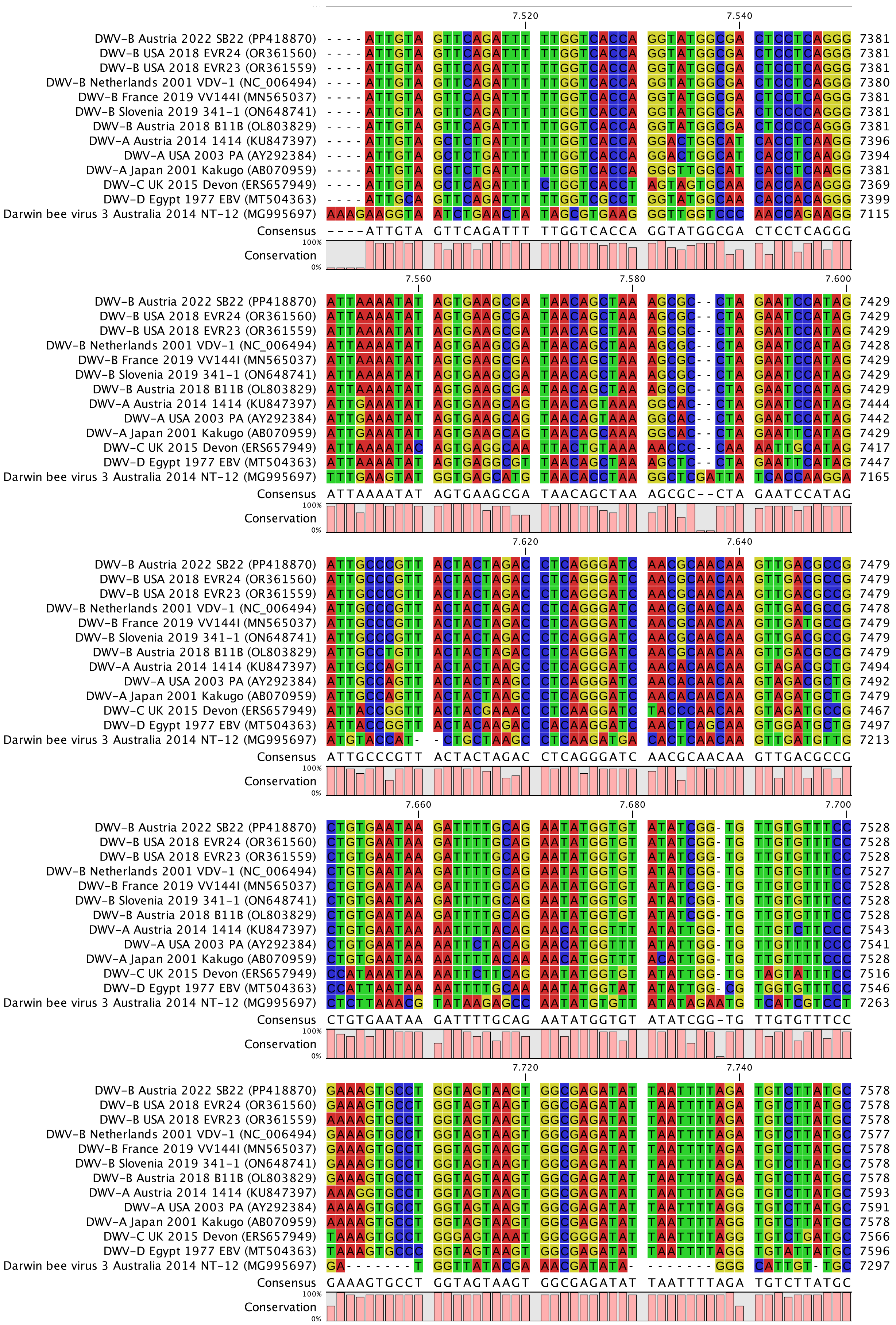

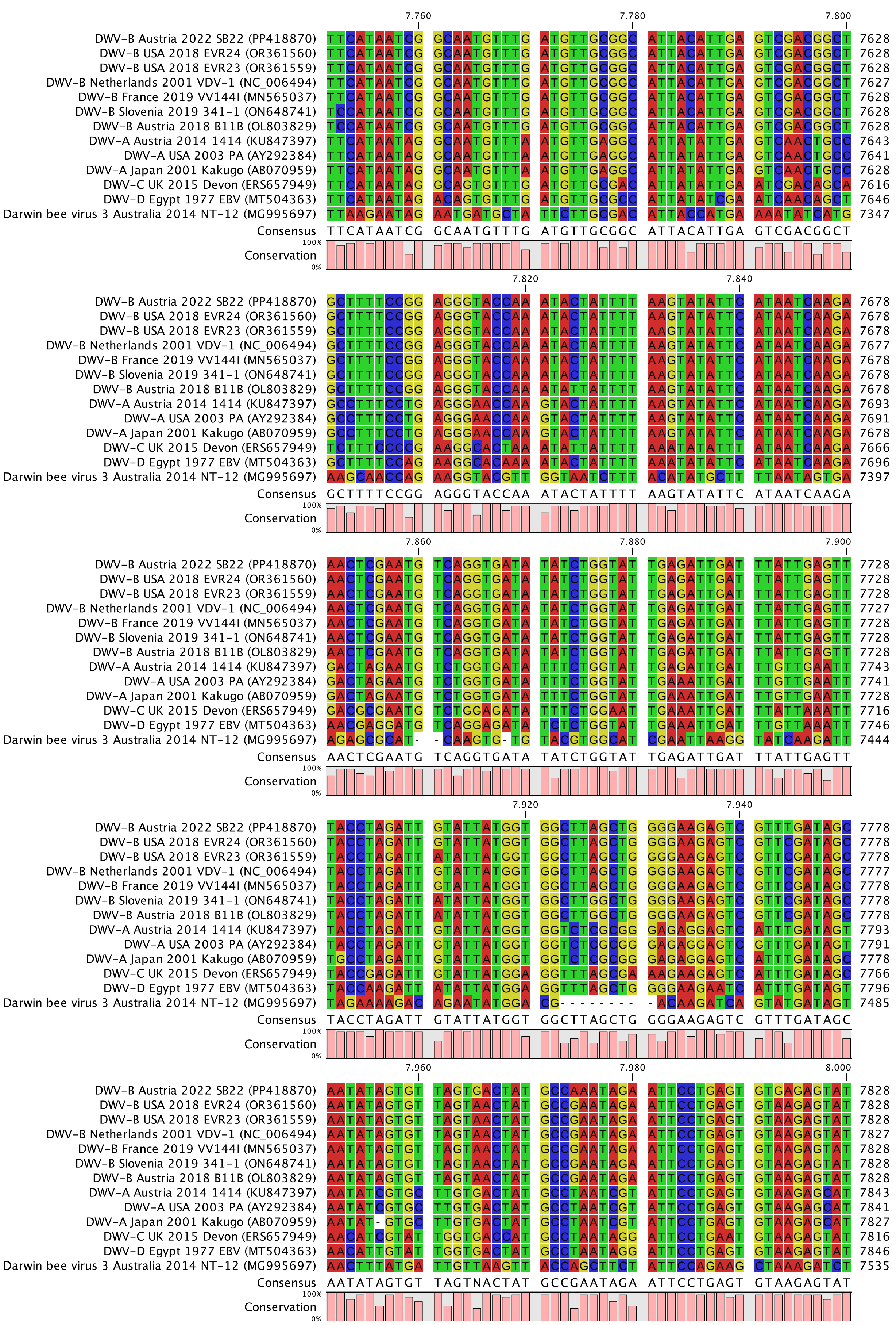

7.760
7.780
7.800
DWV-B Austria 2022 SB22 (PP418870)
DWV-B USA 2018 EVR24 (OR361560)
DWV-B USA 2018 EVR23 (OR361559)
DWV-B Netherlands 2001 VDV-1 (NC_006494)
DWV-B France 2019 VV1441 (MN565037)
DWV-B Slovenia 2019 341-1 (ON648741)
DWV-B Austria 2018 B11B (OL803829)
DWV-A Austria 2014 1414 (KU847397)
DWV-A USA 2003 PA (AY292384)
DWV-A Japan 2001 Kakugo (AB070959)
DWV-C UK 2015 Devon (ERS657949)
DWV-D Egypt 1977 EBV (MT504363)
Darwin bee virus 3 Australia 2014 NT-12 (MG995697)
Consensus TTCATAATCG GCAATGTTTG ATGTTGCGGC ATTACATTGA GTCGACGGCT
Conservation
7.820
7.840
Consensus GCTTTTCCGG AGGGTACCAA ATACTATTTT AAGTATATTC ATAATCAAGA
7.860
7.880
7.900
Consensus AACTCGAATG TCAGGTGATA TATCTGGTAT TGAGATTGAT TTATTGAGTT
7.920
7.940
Consensus TACCTAGATT GTATTATGGT GGCTTAGCTG GGGAAGAGTC GTTTGATAGC
7.960
7.980
8.000
Consensus AATATAGTGT TAGTNACTAT GCCGAATAGA ATTCCTGAGT GTAAGAGTAT

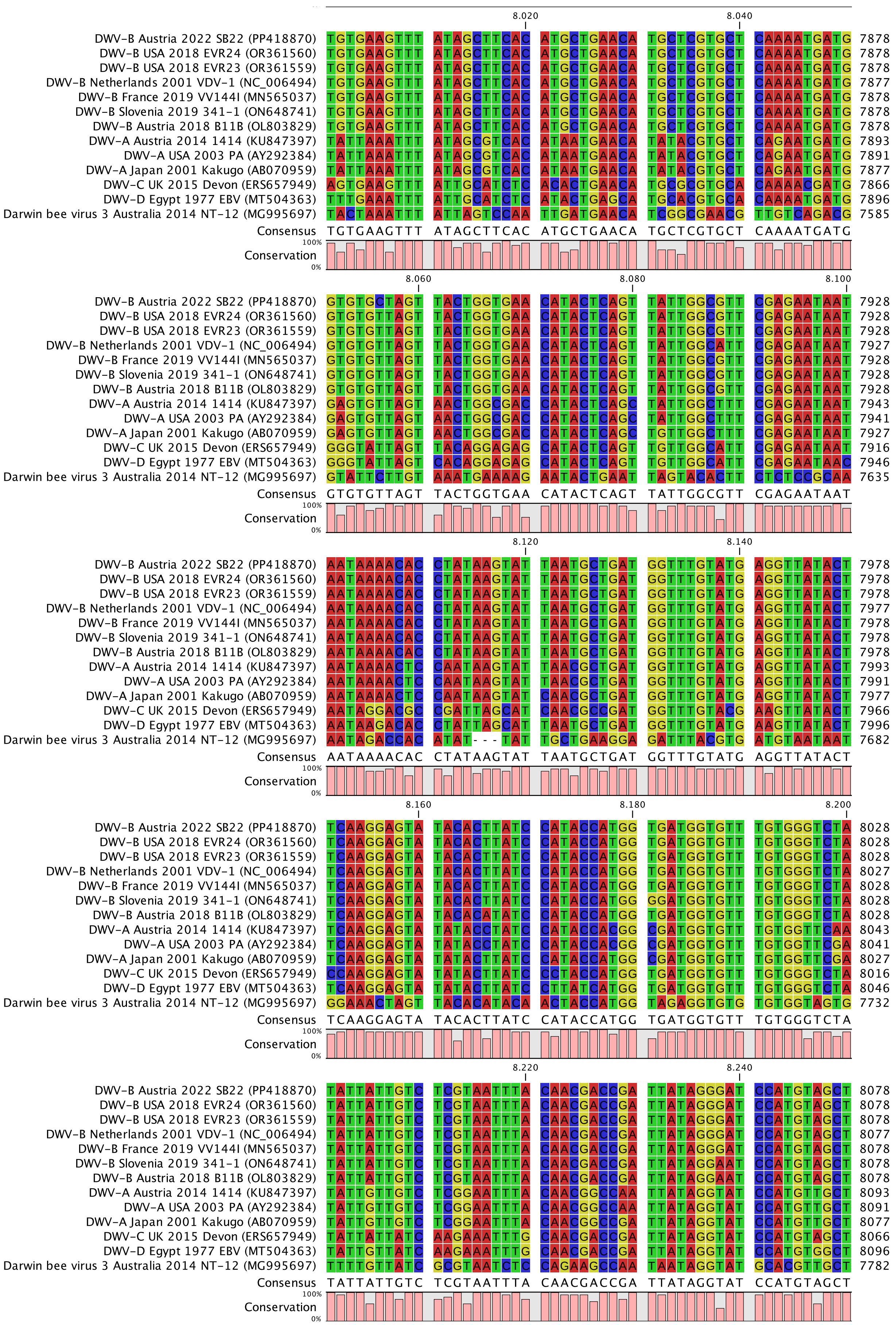
8.020 8.040
DWV-B Austria 2022 SB22 (PP418870) TGTGAAGTTT ATAGCTTCAC ATGCTGAACA TGCTCGTGCT CAAAATGATG 7878
DWV-B USA 2018 EVR24 (OR361560) TGTGAAGTTT ATAGCTTCAC ATGCTGAACA TGCTCGTGCT CAAAATGATG 7878
DWV-B USA 2018 EVR23 (OR361559) TGTGAAGTTT ATAGCTTCAC ATGCTGAACA TGCTCGTGCT CAAAATGATG 7878
DWV-B Netherlands 2001 VDV-1 (NC_006494) TGTGAAGTTT ATAGCTTCAC ATGCTGAACA TGCTCGTGCT CAAAATGATG 7877
DWV-B France 2019 VV144I (MN565037) TGTGAAGTTT ATAGCTTCAC ATGCTGAACA TGCTCGTGCT CAAAATGATG 7878
DWV-B Slovenia 2019 341-1 (ON648741) TGTGAAGTTT ATAGCTTCAC ATGCTGAACA TGCTCGTGCT CAAAATGATG 7878
DWV-B Austria 2018 B11B (OL803829) TGTGAAGTTT ATAGCTTCAC ATGCTGAACA TGCTCGTGCT CAAAATGATG 7878
DWV-A Austria 2014 1414 (KU847397) TATTAAATTT ATAGCGTCAC ATAATGAACA TATACGTGCT CAGAATGATG 7893
DWV-A USA 2003 PA (AY292384) TATTAAATTT ATAGCGTCAC ATAATGAACA TATACGTGCT CAGAATGATG 7891
DWV-A Japan 2001 Kakugo (AB070959) TATTAAATTT ATAGCGTCAC ATAATGAACA TATACGTGCT CAGAATGATG 7877
DWV-C UK 2015 Devon (ERS657949) AGTGAAGTTT ATTGCATCTC ACACTGAACA TGCGCGTGCA CAAAACGATG 7866
DWV-D Egypt 1977 EBV (MT504363) TTTGAAATTT ATTGCATCTC ATACTGAGCA TGCACGTGCA CAAAATGATG 7896
Darwin bee virus 3 Australia 2014 NT-12 (MG995697) TACTAAATTT ATTAGTCCAA TTGATGAACA TCGGCGAACG TTGTCAGACG 7585
Consensus TGTGAAGTTT ATAGCTTCAC ATGCTGAACA TGCTCGTGCT CAAAATGATG
100%
Conservation
0%
8.060 8.080 8.100
DWV-B Austria 2022 SB22 (PP418870) GTGTGCTAGT TACTGGTGAA CATACTCAGT TATTGGCGTT CGAGAATAAT 7928
DWV-B USA 2018 EVR24 (OR361560) GTGTGTTAGT TACTGGTGAA CATACTCAGT TATTGGCGTT CGAGAATAAT 7928
DWV-B USA 2018 EVR23 (OR361559) GTGTGTTAGT TACTGGTGAA CATACTCAGT TATTGGCGTT CGAGAATAAT 7928
DWV-B Netherlands 2001 VDV-1 (NC_006494) GTGTGTTAGT TACTGGTGAA CATACTCAGT TATTGGCATT CGAGAATAAT 7927
DWV-B France 2019 VV144I (MN565037) GTGTGTTAGT TACTGGTGAA CATACTCAGT TATTGGCGTT CGAGAATAAT 7928
DWV-B Slovenia 2019 341-1 (ON648741) GTGTGTTAGT TACTGGTGAA CATACTCAGT TATTGGCGTT CGAGAATAAT 7928
DWV-B Austria 2018 B11B (OL803829) GTGTGTTAGT TACTGGTGAA CATACTCAGT TATTGGCGTT CGAGAATAAT 7928
DWV-A Austria 2014 1414 (KU847397) GAGTGTTAGT AACTGGCGAC CATACTCAGC TATTGGCTTT CGAGAATAAT 7943
DWV-A USA 2003 PA (AY292384) GAGTGTTAGT AACTGGCGAC CATACTCAGC TATTGGCTTT CGAGAATAAT 7941
DWV-A Japan 2001 Kakugo (AB070959) GAGTGTTAGT AACTGGCGAC CATACTCAGC TGTTGGCTTT CGAGAATAAT 7927
DWV-C UK 2015 Devon (ERS657949) GGGTATTAGT TACAGGAGAG CATACTCAGT TGTTGGCATT CGAGAATAAT 7916
DWV-D Egypt 1977 EBV (MT504363) GGGTATTAGT CACAGGAGAG CATACTCAGT TGTTGGCATT CGAGAATAAC 7946
Darwin bee virus 3 Australia 2014 NT-12 (MG995697) GTATTCTTGT AAATGAAAAG AATACTGAAT TAGTACACTT CTCTCCGCAA 7635
Consensus GTGTGTTAGT TACTGGTGAA CATACTCAGT TATTGGCGTT CGAGAATAAT
100%
Conservation
0%
8.120 8.140
DWV-B Austria 2022 SB22 (PP418870) AATAAAACAC CTATAAGTAT TAATGCTGAT GGTTTGTATG AGGTTATACT 7978
DWV-B USA 2018 EVR24 (OR361560) AATAAAACAC CTATAAGTAT TAATGCTGAT GGTTTGTATG AGGTTATACT 7978
DWV-B USA 2018 EVR23 (OR361559) AATAAAACAC CTATAAGTAT TAATGCTGAT GGTTTGTATG AGGTTATACT 7978
DWV-B Netherlands 2001 VDV-1 (NC_006494) AATAAAACAC CTATAAGTAT TAATGCTGAT GGTTTGTATG AGGTTATACT 7977
DWV-B France 2019 VV144I (MN565037) AATAAAACAC CTATAAGTAT TAATGCTGAT GGTTTGTATG AGGTTATACT 7978
DWV-B Slovenia 2019 341-1 (ON648741) AATAAAACAC CTATAAGTAT TAATGCTGAT GGTTTGTATG AGGTTATACT 7978
DWV-B Austria 2018 B11B (OL803829) AATAAAACAC CTATAAGTAT TAATGCTGAT GGTTTGTATG AGGTTATACT 7978
DWV-A Austria 2014 1414 (KU847397) AATAAAACTC CAATAAGTAT TAACGCTGAT GGTTTGTATG AGGTTATACT 7993
DWV-A USA 2003 PA (AY292384) AATAAAACTC CAATAAGTAT TAACGCTGAT GGTTTGTATG AGGTTATACT 7991
DWV-A Japan 2001 Kakugo (AB070959) AATAAAACTC CAATAAGTAT CAACGCTGAT GGTTTGTATG AGGTTATACT 7977
DWV-C UK 2015 Devon (ERS657949) AATAGGACGC CGATTAGCAT CAACGCCGAT GGTTTGTACG AAGTTATACT 7966
DWV-D Egypt 1977 EBV (MT504363) AATAAGACAC CTATTAGCAT TAATGCTGAT GGTTTGTATG AAGTTATACT 7996
Darwin bee virus 3 Australia 2014 NT-12 (MG995697) AATAGACCAC ATAT---TAT TGCTGAAGGA GATTTACGTG ATGTAATAAT 7682
Consensus AATAAAACAC CTATAAGTAT TAATGCTGAT GGTTTGTATG AGGTTATACT
100%
Conservation
0%
8.160 8.180 8.200
DWV-B Austria 2022 SB22 (PP418870) TCAAGGAGTA TACACTTATC CATACCATGG TGATGGTGTT TGTGGGTCTA 8028
DWV-B USA 2018 EVR24 (OR361560) TCAAGGAGTA TACACTTATC CATACCATGG TGATGGTGTT TGTGGGTCTA 8028
DWV-B USA 2018 EVR23 (OR361559) TCAAGGAGTA TACACTTATC CATACCATGG TGATGGTGTT TGTGGGTCTA 8028
DWV-B Netherlands 2001 VDV-1 (NC_006494) TCAAGGAGTA TACACTTATC CATACCATGG TGATGGTGTT TGTGGGTCTA 8027
DWV-B France 2019 VV144I (MN565037) TCAAGGAGTA TACACTTATC CATACCATGG TGATGGTGTT TGTGGGTCTA 8028
DWV-B Slovenia 2019 341-1 (ON648741) TCAAGGAGTA TACACTTATC CATACCATGG GGATGGTGTT TGTGGGTCTA 8028
DWV-B Austria 2018 B11B (OL803829) TCAAGGAGTA TACACATATC CATACCATGG TGATGGTGTT TGTGGGTCTA 8028
DWV-A Austria 2014 1414 (KU847397) TCAAGGAGTA TATACCTATC CATACCACGG CGATGGTGTT TGTGGTTCAA 8043
DWV-A USA 2003 PA (AY292384) TCAAGGAGTA TATACCTATC CATACCACGG CGATGGTGTT TGTGGTTCGA 8041
DWV-A Japan 2001 Kakugo (AB070959) TCAAGGAGTA TATACTTATC CATACCATGG CGATGGTGTT TGTGGTTCGA 8027
DWV-C UK 2015 Devon (ERS657949) CCAAGGAGTA TATACTTATC CCTACCATGG TGATGGTGTT TGTGGGTCTA 8016
DWV-D Egypt 1977 EBV (MT504363) TCAAGGAGTA TATACTTATC CTTATCATGG TGATGGTGTT TGTGGGTCTA 8046
Darwin bee virus 3 Australia 2014 NT-12 (MG995697) GGAAACTAGT TACACATACA ACTACCATGG TAGAGGTGTG TGTGGTAGTG 7732
Consensus TCAAGGAGTA TACACTTATC CATACCATGG TGATGGTGTT TGTGGGTCTA
100%
Conservation
0%
8.220 8.240
DWV-B Austria 2022 SB22 (PP418870) TATTATTGTC TCGTAATTTA CAACGACCGA TTATAGGGAT CCATGTAGCT 8078
DWV-B USA 2018 EVR24 (OR361560) TATTATTGTC TCGTAATTTA CAACGACCGA TTATAGGGAT CCATGTAGCT 8078
DWV-B USA 2018 EVR23 (OR361559) TATTATTGTC TCGTAATTTA CAACGACCGA TTATAGGGAT CCATGTAGCT 8078
DWV-B Netherlands 2001 VDV-1 (NC_006494) TATTATTGTC TCGTAATTTA CAACGACCGA TTATAGGGAT CCATGTAGCT 8077
DWV-B France 2019 VV144I (MN565037) TATTATTGTC TCGTAATTTA CAACGACCGA TTATAGGGAT CCATGTAGCT 8078
DWV-B Slovenia 2019 341-1 (ON648741) TATTATTGTC TCGTAATTTA CAACGACCGA TTATAGGAAT CCATGTAGCT 8078
DWV-B Austria 2018 B11B (OL803829) TATTATTGTC TCGTAATTTA CAACGACCGA TTATAGGAAT CCATGTAGCT 8078
DWV-A Austria 2014 1414 (KU847397) TATTGTTGTC TCGGAATTTA CAACGGCCAA TTATAGGTAT CCATGTTGCT 8093
DWV-A USA 2003 PA (AY292384) TATTGTTGTC TCGGAATTTA CAACGGCCAA TTATAGGTAT CCATGTTGCT 8091
DWV-A Japan 2001 Kakugo (AB070959) TATTGTTGTC TCGGAATTTA CAACGGCCGA TTATAGGTAT CCATGTTGCT 8077
DWV-C UK 2015 Devon (ERS657949) TATTATTATC AAGAAATTTG CAACGACCGA TTATAGGTAT CCATGTAGCT 8066
DWV-D Egypt 1977 EBV (MT504363) TATTGTTATC AAGAAATTTG CAACGACCGA TTATAGGTAT CCATGTGGCT 8096
Darwin bee virus 3 Australia 2014 NT-12 (MG995697) TTTTGTTATC GCGTAATCTC CAGAAGCCAA TAATAGGTAT GCACGTTGCT 7782
Consensus TATTATTGTC TCGTAATTTA CAACGACCGA TTATAGGTAT CCATGTAGCT
100%
Conservation
0%

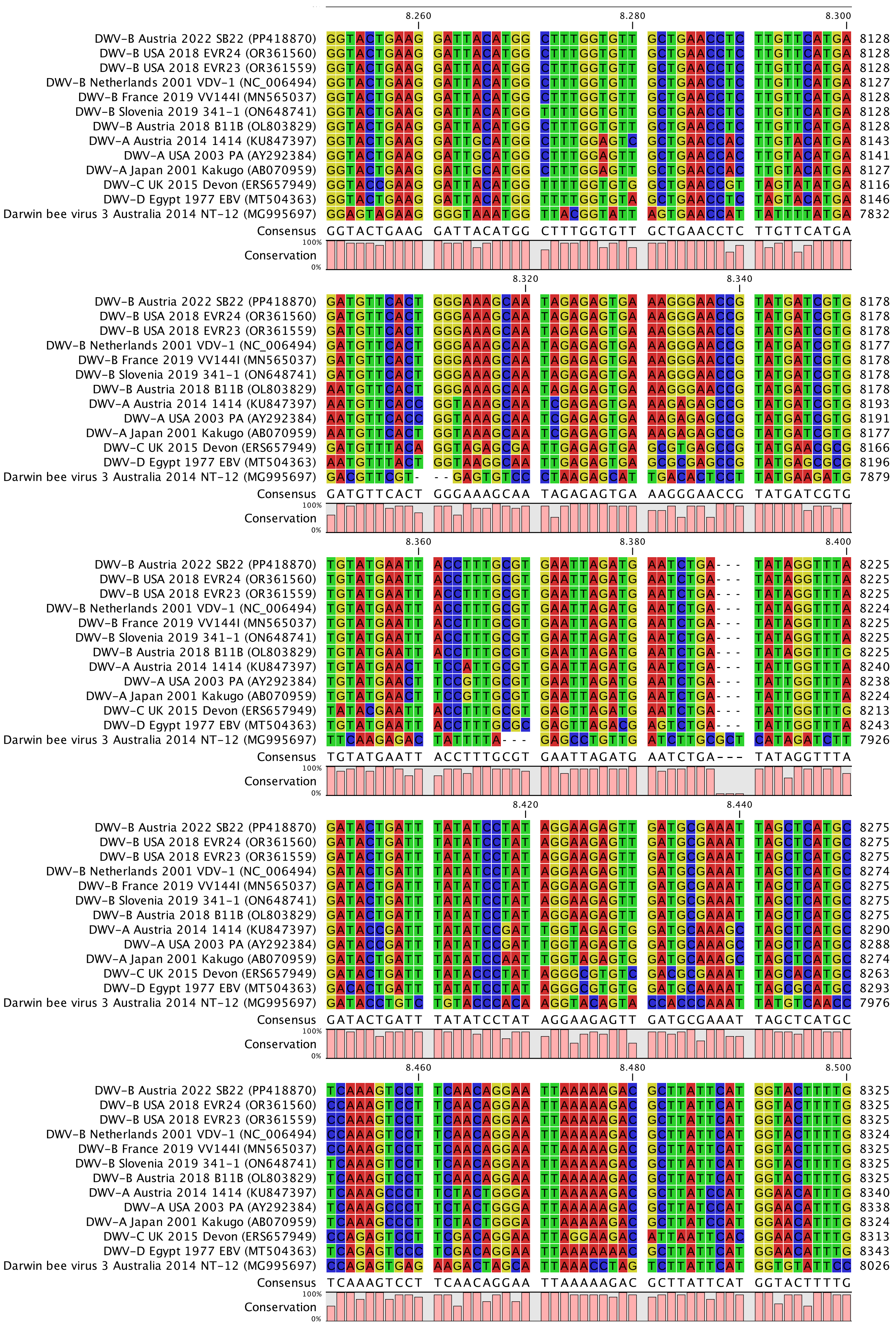
8.260 8.280 8.300
DWV-B Austria 2022 SB22 (PP418870) GGTACTGAAG GATTACATGG CTTTGGTGTT GCTGAACCTC TTGTTCATGA 8128
DWV-B USA 2018 EVR24 (OR361560) GGTACTGAAG GATTACATGG CTTTGGTGTT GCTGAACCTC TTGTTCATGA 8128
DWV-B USA 2018 EVR23 (OR361559) GGTACTGAAG GATTACATGG CTTTGGTGTT GCTGAACCTC TTGTTCATGA 8128
DWV-B Netherlands 2001 VDV-1 (NC_006494) GGTACTGAAG GATTACATGG CTTTGGTGTT GCTGAACCTC TTGTTCATGA 8127
DWV-B France 2019 VV1441 (MN565037) GGTACTGAAG GATTACATGG CTTTGGTGTT GCTGAACCTC TTGTTCATGA 8128
DWV-B Slovenia 2019 341-1 (ON648741) GGTACTGAAG GATTACATGG TTTTGGTGTT GCTGAACCTC TTGTTCATGA 8128
DWV-B Austria 2018 B11B (OL803829) GGTACTGAAG GATTACATGG CTTTGGTGTT GCTGAACCTC TTGTTCATGA 8128
DWV-A Austria 2014 1414 (KU847397) GGTACTGAAG GATTGCATGG CTTTGGAGTC GCTGAACCAC TTGTACATGA 8143
DWV-A USA 2003 PA (AY292384) GGTACTGAAG GATTGCATGG CTTTGGAGTT GCTGAACCAC TTGTACATGA 8141
DWV-A Japan 2001 Kakugo (AB070959) GGTACTGAAG GATTGCATGG CTTTGGAGTT GCTGAACCAC TTGTACATGA 8127
DWV-C UK 2015 Devon (ERS657949) GGTACCGAAG GATTACATGG TTTTGGTGTG GCTGAACCGT TAGTATATGA 8116
DWV-D Egypt 1977 EBV (MT504363) GGTACTGAAG GATTACATGG TTTTGGTGTA GCTGAACCTC TAGTACATGA 8146
Darwin bee virus 3 Australia 2014 NT-12 (MG995697) GGAGTAGAAG GGGTAAATGG TTACGGTATT AGTGAACCAT TATTTTATGA 7832
Consensus GGTACTGAAG GATTACATGG CTTTGGTGTT GCTGAACCTC TTGTTCATGA
100%
Conservation
0%
8.320 8.340
DWV-B Austria 2022 SB22 (PP418870) GATGTTCACT GGGAAAGCAA TAGAGAGTGA AAGGGAACCG TATGATCGTG 8178
DWV-B USA 2018 EVR24 (OR361560) GATGTTCACT GGGAAAGCAA TAGAGAGTGA AAGGGAACCG TATGATCGTG 8178
DWV-B USA 2018 EVR23 (OR361559) GATGTTCACT GGGAAAGCAA TAGAGAGTGA AAGGGAACCG TATGATCGTG 8178
DWV-B Netherlands 2001 VDV-1 (NC_006494) GATGTTCACT GGGAAAGCAA TAGAGAGTGA AAGGGAACCG TATGATCGTG 8177
DWV-B France 2019 VV1441 (MN565037) GATGTTCACT GGGAAAGCAA TAGAGAGTGA AAGGGAACCG TATGATCGTG 8178
DWV-B Slovenia 2019 341-1 (ON648741) GATGTTCACT GGGAAAGCAA TAGAGAGTGA AAGGGAACCG TATGATCGTG 8178
DWV-B Austria 2018 B11B (OL803829) AATGTTCACT GGGAAAGCAA TAGAGAGTGA AAGGGAACCG TATGATCGTG 8178
DWV-A Austria 2014 1414 (KU847397) AATGTTCACC GGTAAAGCAA TCGAGAGTGA AAGAGAGCCG TATGATCGTG 8193
DWV-A USA 2003 PA (AY292384) AATGTTCACC GGTAAAGCAA TCGAGAGTGA AAGAGAGCCG TATGATCGTG 8191
DWV-A Japan 2001 Kakugo (AB070959) AATGTTCACT GGTAAAGCAA TCGAGAGTGA AAGAGAGCCG TATGATCGTG 8177
DWV-C UK 2015 Devon (ERS657949) GATGTTTACA GGTAGAGCGA TTGAGAGTGA GCGTGAGCCG TATGAACGCG 8166
DWV-D Egypt 1977 EBV (MT504363) AATGTTTACT GGTAAGGCAA TTGAGAGTGA GCGCGAGCCG TATGAGCGCG 8196
Darwin bee virus 3 Australia 2014 NT-12 (MG995697) GACGTTCGT- --GAGTGTCC CTAAGAGCAT TGACACTCCT TATGAAGATG 7879
Consensus GATGTTCACT GGGAAAGCAA TAGAGAGTGA AAGGGAACCG TATGATCGTG
100%
Conservation
0%
8.360 8.380 8.400
DWV-B Austria 2022 SB22 (PP418870) TGTATGAATT ACCTTTGCGT GAATTAGATG AATCTGA--- TATAGGTTTA 8225
DWV-B USA 2018 EVR24 (OR361560) TGTATGAATT ACCTTTGCGT GAATTAGATG AATCTGA--- TATAGGTTTA 8225
DWV-B USA 2018 EVR23 (OR361559) TGTATGAATT ACCTTTGCGT GAATTAGATG AATCTGA--- TATAGGTTTA 8225
DWV-B Netherlands 2001 VDV-1 (NC_006494) TGTATGAATT ACCTTTGCGT GAATTAGATG AATCTGA--- TATAGGTTTA 8224
DWV-B France 2019 VV1441 (MN565037) TGTATGAATT ACCTTTGCGT GAATTAGATG AATCTGA--- TATAGGTTTA 8225
DWV-B Slovenia 2019 341-1 (ON648741) TGTATGAATT ACCTTTGCGT GAATTAGATG AATCTGA--- TATAGGTTTA 8225
DWV-B Austria 2018 B11B (OL803829) TGTATGAATT ACCTTTGCGT GAATTAGATG AATCTGA--- TATAGGTTTG 8225
DWV-A Austria 2014 1414 (KU847397) TGTATGAACT TCCATTGCGT GAATTAGATG AATCTGA--- TATTGGTTTA 8240
DWV-A USA 2003 PA (AY292384) TGTATGAACT TCCGTTGCGT GAATTAGATG AATCTGA--- TATTGGTTTA 8238
DWV-A Japan 2001 Kakugo (AB070959) TGTATGAACT TCCGTTGCGT GAATTAGATG AATCTGA--- TATTGGTTTA 8224
DWV-C UK 2015 Devon (ERS657949) TATACGAATT ACCTTTGCGT GAGTTAGATG AATCTGA--- TATTGGTTTG 8213
DWV-D Egypt 1977 EBV (MT504363) TGTATGAATT ACCTTTGCGC GAGTTAGACG AGTCTGA--- TATTGGTTTA 8243
Darwin bee virus 3 Australia 2014 NT-12 (MG995697) TTCAAGAGAC TATTTTA--- GAGCCTGTTG ATCTTGCGCT CATAGATCTT 7926
Consensus TGTATGAATT ACCTTTGCGT GAATTAGATG AATCTGA--- TATAGGTTTA
100%
Conservation
0%
8.420 8.440
DWV-B Austria 2022 SB22 (PP418870) GATACTGATT TATATCCTAT AGGAAGAGTT GATGCGAAAT TAGCTCATGC 8275
DWV-B USA 2018 EVR24 (OR361560) GATACTGATT TATATCCTAT AGGAAGAGTT GATGCGAAAT TAGCTCATGC 8275
DWV-B USA 2018 EVR23 (OR361559) GATACTGATT TATATCCTAT AGGAAGAGTT GATGCGAAAT TAGCTCATGC 8275
DWV-B Netherlands 2001 VDV-1 (NC_006494) GATACTGATT TATATCCTAT AGGAAGAGTT GATGCGAAAT TAGCTCATGC 8274
DWV-B France 2019 VV1441 (MN565037) GATACTGATT TATATCCTAT AGGAAGAGTT GATGCGAAAT TAGCTCATGC 8275
DWV-B Slovenia 2019 341-1 (ON648741) GATACTGATT TATATCCTAT AGGAAGAGTT GATGCGAAAT TAGCTCATGC 8275
DWV-B Austria 2018 B11B (OL803829) GATACTGATT TATATCCTAT AGGAAGAGTT GATGCGAAAT TAGCTCATGC 8275
DWV-A Austria 2014 1414 (KU847397) GATACCGATT TATATCCGAT TGGTAGAGTG GATGCAAAGC TAGCTCATGC 8290
DWV-A USA 2003 PA (AY292384) GATACCGATT TATATCCGAT TGGTAGAGTG GATGCAAAGC TAGCTCATGC 8288
DWV-A Japan 2001 Kakugo (AB070959) GATACTGATT TATATCCAAT TGGTAGAGTG GATGCAAAGC TAGCTCATGC 8274
DWV-C UK 2015 Devon (ERS657949) GATACTGATT TATACCCTAT AGGGCGTGTC GACGCGAAAT TAGCACATGC 8263
DWV-D Egypt 1977 EBV (MT504363) GACACTGATT TATATCCTAT AGGGCGTGTG GATGCAAAAT TAGCGCATGC 8293
Darwin bee virus 3 Australia 2014 NT-12 (MG995697) GATACCTGTC TGTACCCACA AGGTACAGTA CCACCCAAAT TATGTCAACC 7976
Consensus GATACTGATT TATATCCTAT AGGAAGAGTT GATGCGAAAT TAGCTCATGC
100%
Conservation
0%
8.460 8.480 8.500
DWV-B Austria 2022 SB22 (PP418870) TCAAAGTCCT TCAACAGGAA TTAAAAAGAC GCTTATTCAT GGTACTTTTG 8325
DWV-B USA 2018 EVR24 (OR361560) CCAAAGTCCT TCAACAGGAA TTAAAAAGAC GCTTATTCAT GGTACTTTTG 8325
DWV-B USA 2018 EVR23 (OR361559) CCAAAGTCCT TCAACAGGAA TTAAAAAGAC GCTTATTCAT GGTACTTTTG 8325
DWV-B Netherlands 2001 VDV-1 (NC_006494) CCAAAGTCCT TCAACAGGAA TTAAAAAGAC GCTTATTCAT GGTACTTTTG 8324
DWV-B France 2019 VV1441 (MN565037) CCAAAGTCCT TCAACAGGAA TTAAAAAGAC GCTTATTCAT GGTACTTTTG 8325
DWV-B Slovenia 2019 341-1 (ON648741) TCAAAGTCCT TCAACAGGAA TTAAAAAGAC GCTTATTCAT GGTACTTTTG 8325
DWV-B Austria 2018 B11B (OL803829) TCAAAGTCCT TCAACAGGAA TTAAAAAGAC GCTTATTCAT GGTACTTTTG 8325
DWV-A Austria 2014 1414 (KU847397) TCAAAGTCCT TCTACTGGGA TTAAAAAGAC GCTTATCCAT GGAACATTTG 8340
DWV-A USA 2003 PA (AY292384) TCAAAGCCCT TCTACTGGGA TTAAAAAGAC GCTTATCCAT GGAACATTTG 8338
DWV-A Japan 2001 Kakugo (AB070959) TCAAAGCCCT TCTACTGGGA TTAAAAAGAC GCTTATCCAT GGAACATTTG 8324
DWV-C UK 2015 Devon (ERS657949) CCAGAGTCCT TCGACAGGAA TTAGGAAGAC ATTAATTCAC GGAACATTTG 8313
DWV-D Egypt 1977 EBV (MT504363) TCAGAGTCCC TCGACAGGAA TTAAAAAAAC GCTTATTCAT GGAACATTTG 8343
Darwin bee virus 3 Australia 2014 NT-12 (MG995697) CCAGAGTGAG AAGACTAGCA TTAAACCTAG TCTTATTCAT GGTGTATTCC 8026
Consensus TCAAAGTCCT TCAACAGGAA TTAAAAAGAC GCTTATTCAT GGTACTTTTG
100%
Conservation
0%

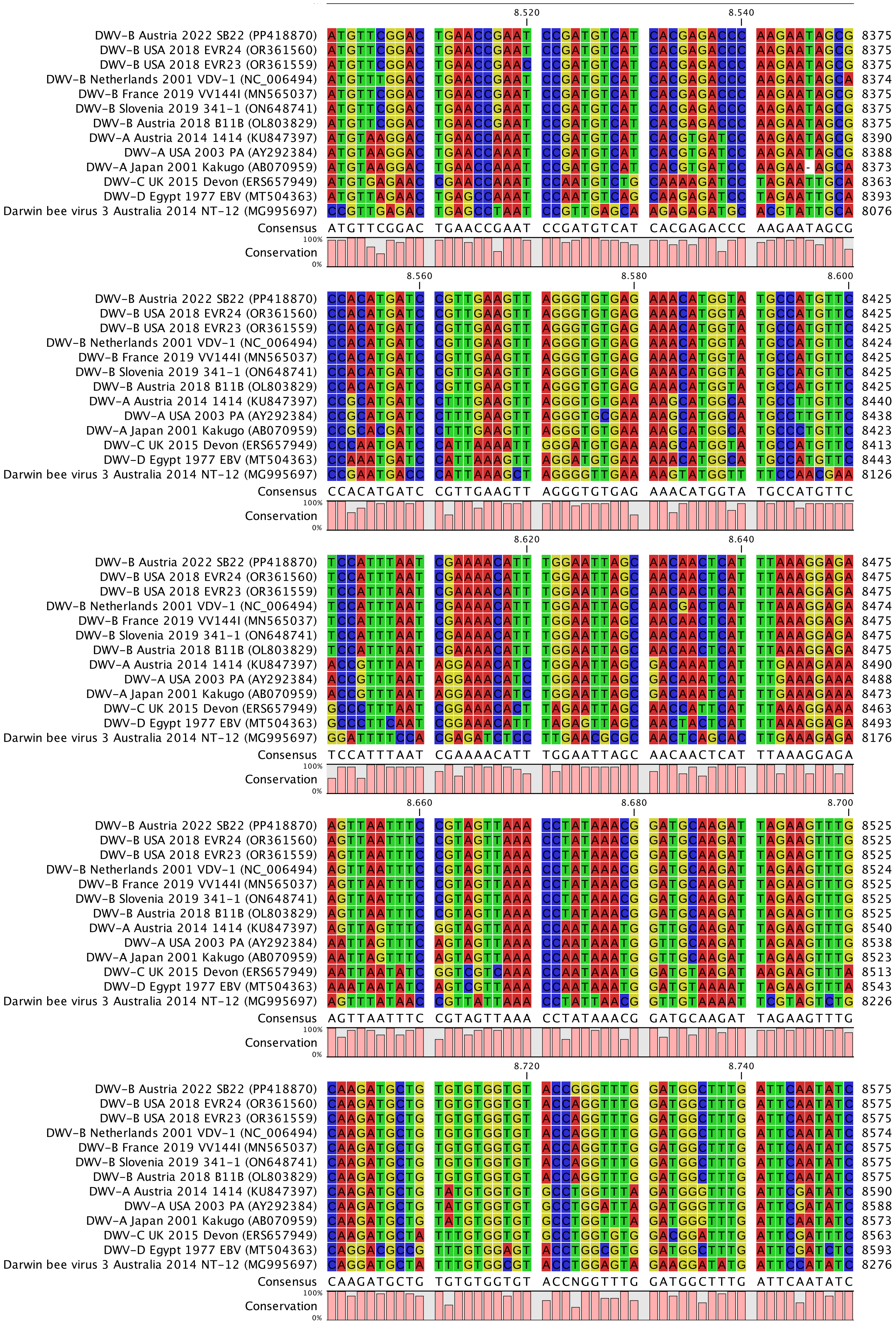
8.520 8.540
DWV-B Austria 2022 SB22 (PP418870) ATGTTCGGAC TGAACCGAAT CCGATGTCAT CACGAGACCC AAGAATAGCG 8375
DWV-B USA 2018 EVR24 (OR361560) ATGTTCGGAC TGAACCGAAT CCGATGTCAT CACGAGACCC AAGAATAGCG 8375
DWV-B USA 2018 EVR23 (OR361559) ATGTTCGGAC TGAACCGAAC CCGATGTCAT CACGAGACCC AAGAATAGCG 8375
DWV-B Netherlands 2001 VDV-1 (NC_006494) ATGTTTGGAC TGAACCGAAT CCGATGTCAT CACGAGACCC AAGAATAGCA 8374
DWV-B France 2019 VV144I (MN565037) ATGTTCGGAC TGAACCGAAT CCGATGTCAT CACGAGACCC AAGAATAGCG 8375
DWV-B Slovenia 2019 341-1 (ON648741) ATGTTCGGAC TGAACCGAAT CCGATGTCAT CACGAGACCC AAGAATAGCG 8375
DWV-B Austria 2018 B11B (OL803829) ATGTTCGGAC TGAACCGAAT CCGATGTCAT CACGAGACCC AAGAATAGCG 8375
DWV-A Austria 2014 1414 (KU847397) ATGTAAGGAC TGAACCAAAT CCGATGTCAT CACGTGATCC AAGAATAGCG 8390
DWV-A USA 2003 PA (AY292384) ATGTAAGGAC TGAACCAAAT CCGATGTCAT CACGTGATCC AAGAATAGCG 8388
DWV-A Japan 2001 Kakugo (AB070959) ATGTAAGGAC TGAACCAAAT CCGATGTCAT CACGTGATCC AAGAA-AGCA 8373
DWV-C UK 2015 Devon (ERS657949) ATGTGAGAAC CGAACCAAAT CCAATGTCTG CAAAAGATCC TAGAATTGCA 8363
DWV-D Egypt 1977 EBV (MT504363) ATGTTAGAAC TGAGCCAAAT CCAATGTCAG CAAGAGATCC TAGAATTGCA 8393
Darwin bee virus 3 Australia 2014 NT-12 (MG995697) CCGTTGAGAC TGAGCCTAAT CCGTTGAGCA AGAGAGATGC ACGTATTGCA 8076
Consensus ATGTTCGGAC TGAACCGAAT CCGATGTCAT CACGAGACCC AAGAATAGCG
Conservation 100% 0%
8.560 8.580 8.600
DWV-B Austria 2022 SB22 (PP418870) CCACATGATC CGTTGAAGTT AGGGTGTGAG AAACATGGTA TGCCATGTTC 8425
DWV-B USA 2018 EVR24 (OR361560) CCACATGATC CGTTGAAGTT AGGGTGTGAG AAACATGGTA TGCCATGTTC 8425
DWV-B USA 2018 EVR23 (OR361559) CCACATGATC CGTTGAAGTT AGGGTGTGAG AAACATGGTA TGCCATGTTC 8425
DWV-B Netherlands 2001 VDV-1 (NC_006494) CCACATGATC CGTTGAAGTT AGGGTGTGAG AAACATGGTA TGCCATGTTC 8424
DWV-B France 2019 VV144I (MN565037) CCACATGATC CGTTGAAGTT AGGGTGTGAG AAACATGGTA TGCCATGTTC 8425
DWV-B Slovenia 2019 341-1 (ON648741) CCACATGATC CGTTGAAGTT AGGGTGTGAG AAACATGGTA TGCCATGTTC 8425
DWV-B Austria 2018 B11B (OL803829) CCACATGATC CGTTGAAGTT AGGGTGTGAG AAACATGGTA TGCCATGTTC 8425
DWV-A Austria 2014 1414 (KU847397) CCGCATGATC CTTTGAAGTT AGGGTGTGAA AAGCATGGCA TGCCTTGTTC 8440
DWV-A USA 2003 PA (AY292384) CCGCATGATC CTTTGAAGTT AGGGTGCGAA AAGCATGGCA TGCCTTGTTC 8438
DWV-A Japan 2001 Kakugo (AB070959) CCGCACGATC CTTTGAAGTT AGGGTGTGAA AAGCATGGCA TGCCCTGTTC 8423
DWV-C UK 2015 Devon (ERS657949) CCCAATGATC CATTAAAATT GGGATGTGAA AAGCATGGTA TGCCATGTTC 8413
DWV-D Egypt 1977 EBV (MT504363) CCAAATGATC CATTAAAGTT AGGATGTGAA AAACATGGCA TGCCATGTTC 8443
Darwin bee virus 3 Australia 2014 NT-12 (MG995697) CCGAATGACC CATTAAAGCT AGGGGTTGAA AAGTATGGTT TTCCAACGAA 8126
Consensus CCACATGATC CGTTGAAGTT AGGGTGTGAG AAACATGGTA TGCCATGTTC
Conservation 100% 0%
8.620 8.640
DWV-B Austria 2022 SB22 (PP418870) TCCATTTAAT CGAAAACATT TGGAATTAGC AACAACTCAT TTAAAGGAGA 8475
DWV-B USA 2018 EVR24 (OR361560) TCCATTTAAT CGAAAACATT TGGAATTAGC AACAACTCAT TTAAAGGAGA 8475
DWV-B USA 2018 EVR23 (OR361559) TCCATTTAAT CGAAAACATT TGGAATTAGC AACAACTCAT TTAAAGGAGA 8475
DWV-B Netherlands 2001 VDV-1 (NC_006494) TCCATTTAAT CGAAAACATT TGGAATTAGC AACGACTCAT TTAAAGGAGA 8474
DWV-B France 2019 VV144I (MN565037) TCCATTTAAT CGAAAACATT TGGAATTAGC AACAACTCAT TTAAAGGAGA 8475
DWV-B Slovenia 2019 341-1 (ON648741) TCCATTTAAT CGAAAACATT TGGAATTAGC AACAACTCAT TTAAAGGAGA 8475
DWV-B Austria 2018 B11B (OL803829) TCCATTTAAT CGAAAACATT TGGAATTAGC AACAACTCAT TTAAAGGAGA 8475
DWV-A Austria 2014 1414 (KU847397) ACCGTTTAAT AGGAAACATC TGGAATTAGC GACAAATCAT TTGAAAGAAA 8490
DWV-A USA 2003 PA (AY292384) ACCGTTTAAT AGGAAACATC TGGAATTAGC GACAAATCAT TTGAAAGAAA 8488
DWV-A Japan 2001 Kakugo (AB070959) ACCGTTTAAT AGGAAACATC TGGAATTAGC GACAAATCAT TTGAAAGAAA 8473
DWV-C UK 2015 Devon (ERS657949) GCCCTTTAAT CGGAAACACT TAGAATTAGC AACCATTCAT TTAAAGGAAA 8463
DWV-D Egypt 1977 EBV (MT504363) GCCCTTCAAT CGGAAACATT TAGAGTTAGC AACTACTCAT TTAAAGGAGA 8493
Darwin bee virus 3 Australia 2014 NT-12 (MG995697) GGATTTTCCA CGAGATCTCC TTGAACGCGC AACTCAGCAC TTGAAAGAGA 8176
Consensus TCCATTTAAT CGAAAACATT TGGAATTAGC AACAACTCAT TTAAAGGAGA
Conservation 100% 0%
8.660 8.680 8.700
DWV-B Austria 2022 SB22 (PP418870) AGTTAATTTC CGTAGTTAAA CCTATAAACG GATGCAAGAT TAGAAGTTTG 8525
DWV-B USA 2018 EVR24 (OR361560) AGTTAATTTC CGTAGTTAAA CCTATAAACG GATGCAAGAT TAGAAGTTTG 8525
DWV-B USA 2018 EVR23 (OR361559) AGTTAATTTC CGTAGTTAAA CCTATAAACG GATGCAAGAT TAGAAGTTTG 8525
DWV-B Netherlands 2001 VDV-1 (NC_006494) AGTTAATTTC CGTAGTTAAA CCTATAAACG GATGCAAGAT TAGAAGTTTG 8524
DWV-B France 2019 VV144I (MN565037) AGTTAATTTC CGTAGTTAAA CCTATAAACG GATGCAAGAT TAGAAGTTTG 8525
DWV-B Slovenia 2019 341-1 (ON648741) AGTTAATTTC CGTAGTTAAA CCTATAAACG GATGCAAGAT TAGAAGTTTG 8525
DWV-B Austria 2018 B11B (OL803829) AGTTAATTTC CGTAGTTAAA CCTATAAACG GATGCAAGAT TAGAAGTTTG 8525
DWV-A Austria 2014 1414 (KU847397) AGTTAGTTTC GGTAGTTAAA CCAATAAATG GTTGCAAGAT TAGAAGTTTG 8540
DWV-A USA 2003 PA (AY292384) AATTAGTTTC AGTAGTTAAA CCAATAAATG GTTGCAAGAT TAGAAGTTTG 8538
DWV-A Japan 2001 Kakugo (AB070959) AATTAGTTTC AGTAGTTAAA CCAATAAATG GTTGCAAGAT TAGAAGTTTG 8523
DWV-C UK 2015 Devon (ERS657949) AATTAATATC GGTCGTCAAA CCAATAAATG GATGTAAGAT AAGAAGTTTA 8513
DWV-D Egypt 1977 EBV (MT504363) AAATAATATC AGTCGTTAAA CCAATAAATG GATGTAAAAT TAGAAGTTTA 8543
Darwin bee virus 3 Australia 2014 NT-12 (MG995697) AGTTTATAAC CGTTATTAAA CCTATTAACG GTTGTAAAAT TCGTAGTCTG 8226
Consensus AGTTAATTTC CGTAGTTAAA CCTATAAACG GATGCAAGAT TAGAAGTTTG
Conservation 100% 0%
8.720 8.740
DWV-B Austria 2022 SB22 (PP418870) CAAGATGCTG TGTGTGGTGT ACCGGGTTTG GATGGCTTTG ATTCAATATC 8575
DWV-B USA 2018 EVR24 (OR361560) CAAGATGCTG TGTGTGGTGT ACCAGGTTTG GATGGCTTTG ATTCAATATC 8575
DWV-B USA 2018 EVR23 (OR361559) CAAGATGCTG TGTGTGGTGT ACCAGGTTTG GATGGCTTTG ATTCAATATC 8575
DWV-B Netherlands 2001 VDV-1 (NC_006494) CAAGATGCTG TGTGTGGTGT ACCAGGTTTG GATGGCTTTG ATTCAATATC 8574
DWV-B France 2019 VV144I (MN565037) CAAGATGCTG TGTGTGGTGT ACCAGGTTTG GATGGCTTTG ATTCAATATC 8575
DWV-B Slovenia 2019 341-1 (ON648741) CAAGATGCTG TGTGTGGTGT ACCAGGTTTG GATGGCTTTG ATTCAATATC 8575
DWV-B Austria 2018 B11B (OL803829) CAAGATGCTG TGTGTGGTGT ACCAGGTTTG GATGGCTTTG ATTCAATATC 8575
DWV-A Austria 2014 1414 (KU847397) CAAGATGCTG TATGTGGTGT GCCTGGTTTA GATGGGTTTG ATTCGATATC 8590
DWV-A USA 2003 PA (AY292384) CAAGATGCTG TATGTGGTGT GCCTGGATTA GATGGGTTTG ATTCGATATC 8588
DWV-A Japan 2001 Kakugo (AB070959) CAAGATGCTG TATGTGGTGT GCCTGGTTTA GATGGGTTTG ATTCAATATC 8573
DWV-C UK 2015 Devon (ERS657949) CAAGATGCTA TTTGTGGTGT GCCTGGTGTG GACGGATTTG ATTCGATTTC 8563
DWV-D Egypt 1977 EBV (MT504363) CAGGACGCCG TTTGTGGAGT ACCTGGCGTG GATGGCTTTG ATTCGATCTC 8593
Darwin bee virus 3 Australia 2014 NT-12 (MG995697) CAGGATGCTA TTTGTGGCGT ACCTGGAGTA GAAGGATATG ATTCCATATC 8276
Consensus CAAGATGCTG TGTGTGGTGT ACCNGGTTTG GATGGCTTTG ATTCAATATC
Conservation 100% 0%

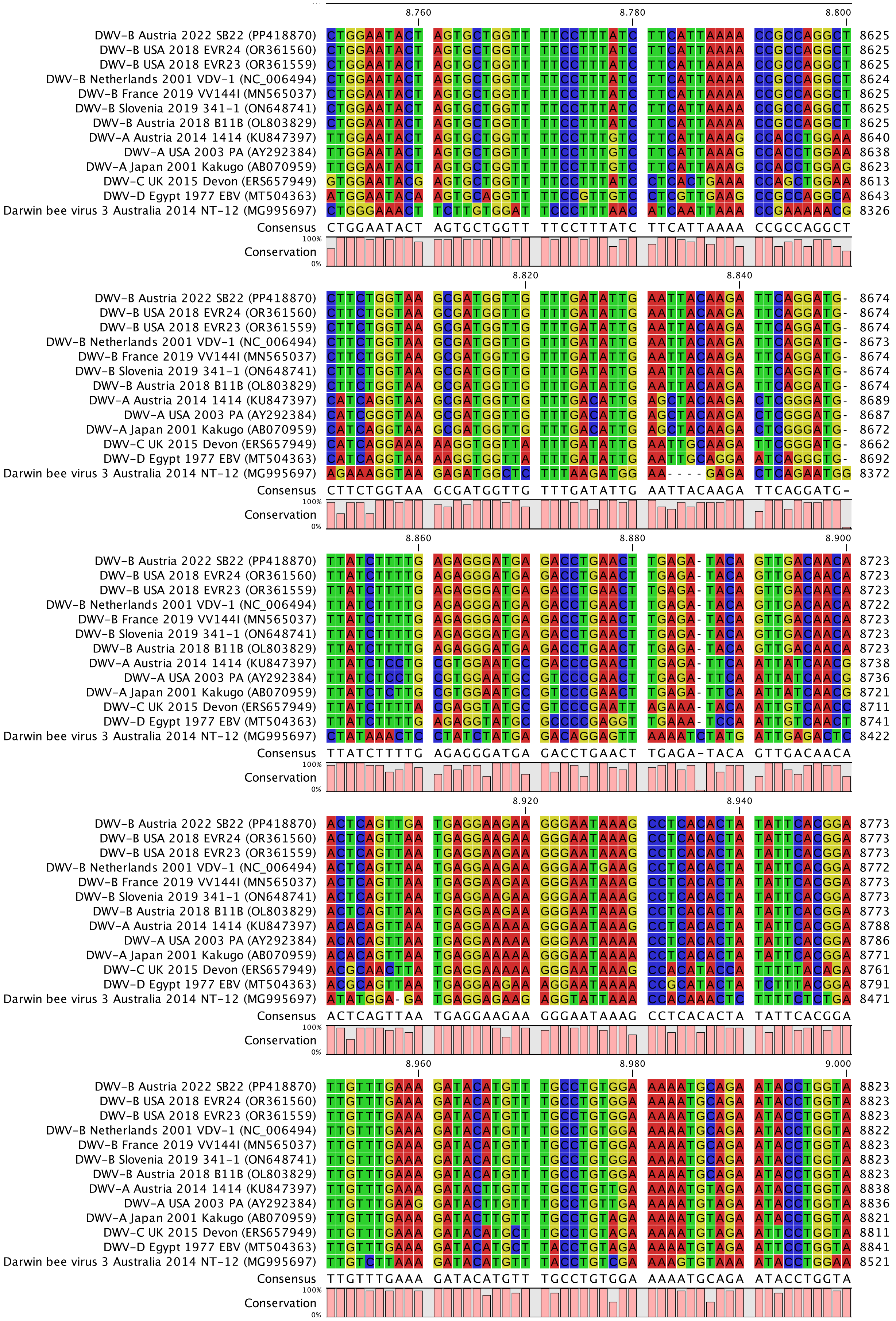
8.760 8.780 8.800
DWV-B Austria 2022 SB22 (PP418870) CTGGAATACT AGTGCTGGTT TTCCTTTATC TTCATTAAAA CCGCCAGGCT 8625
DWV-B USA 2018 EVR24 (OR361560) CTGGAATACT AGTGCTGGTT TTCCTTTATC TTCATTAAAA CCGCCAGGCT 8625
DWV-B USA 2018 EVR23 (OR361559) CTGGAATACT AGTGCTGGTT TTCCTTTATC TTCATTAAAA CCGCCAGGCT 8625
DWV-B Netherlands 2001 VDV-1 (NC_006494) CTGGAATACT AGTGCTGGTT TTCCTTTATC TTCATTAAAA CCGCCAGGCT 8624
DWV-B France 2019 VV144I (MN565037) CTGGAATACT AGTGCTGGTT TTCCTTTATC TTCATTAAAA CCGCCAGGCT 8625
DWV-B Slovenia 2019 341-1 (ON648741) CTGGAATACT AGTGCTGGTT TTCCTTTATC TTCATTAAAA CCGCCAGGCT 8625
DWV-B Austria 2018 B11B (OL803829) CTGGAATACT AGTGCTGGTT TTCCTTTATC TTCATTAAAA CCGCCAGGCT 8625
DWV-A Austria 2014 1414 (KU847397) TTGGAATACT AGTGCTGGTT TTCCTTTGTC TTCATTAAAG CCACCTGGAA 8640
DWV-A USA 2003 PA (AY292384) TTGGAATACT AGTGCTGGTT TTCCTTTGTC TTCATTAAAG CCACCTGGAA 8638
DWV-A Japan 2001 Kakugo (AB070959) TTGGAATACT AGTGCTGGTT TTCCTTTGTC TTCATTAAAG CCACCTGGAG 8623
DWV-C UK 2015 Devon (ERS657949) GTGGAATACG AGTGCTGGTT TTCCTTTATC CTCACTGAAA CCAGCTGGAA 8613
DWV-D Egypt 1977 EBV (MT504363) ATGGAATACA AGTGCAGGTT TTCCGTTGTC CTCGTTGAAG CCGCCAGGCA 8643
Darwin bee virus 3 Australia 2014 NT-12 (MG995697) CTGGGAAACT TCTTGTGGAT TCCCTTTAAC ATCAATTAAA CCGAAAAACG 8326
Consensus CTGGAATACT AGTGCTGGTT TTCCTTTATC TTCATTAAAA CCGCCAGGCT
100%
Conservation
0%
8.820 8.840
DWV-B Austria 2022 SB22 (PP418870) CTTCTGGTAA GCGATGGTTG TTTGATATTG AATTACAAGA TTCAGGATG- 8674
DWV-B USA 2018 EVR24 (OR361560) CTTCTGGTAA GCGATGGTTG TTTGATATTG AATTACAAGA TTCAGGATG- 8674
DWV-B USA 2018 EVR23 (OR361559) CTTCTGGTAA GCGATGGTTG TTTGATATTG AATTACAAGA TTCAGGATG- 8674
DWV-B Netherlands 2001 VDV-1 (NC_006494) CTTCTGGTAA GCGATGGTTG TTTGATATTG AATTACAAGA TTCAGGATG- 8673
DWV-B France 2019 VV144I (MN565037) CTTCTGGTAA GCGATGGTTG TTTGATATTG AATTACAAGA TTCAGGATG- 8674
DWV-B Slovenia 2019 341-1 (ON648741) CTTCTGGTAA GCGATGGTTG TTTGATATTG AATTACAAGA TTCAGGATG- 8674
DWV-B Austria 2018 B11B (OL803829) CTTCTGGTAA GCGATGGTTG TTTGATATTG AATTACAAGA TTCAGGATG- 8674
DWV-A Austria 2014 1414 (KU847397) CATCAGGTAA GCGATGGTTG TTTGACATTG AGCTACAAGA CTCGGGATG- 8689
DWV-A USA 2003 PA (AY292384) CATCGGGTAA GCGATGGTTG TTTGACATTG AGCTACAAGA CTCGGGATG- 8687
DWV-A Japan 2001 Kakugo (AB070959) CATCAGGTAA GCGATGGTTG TTTGACATTG AGCTACAAGA CTCGGGATG- 8672
DWV-C UK 2015 Devon (ERS657949) CATCAGGAAA AAGGTGGTTA TTTGATATTG AATTGCAAGA TTCGGGATG- 8662
DWV-D Egypt 1977 EBV (MT504363) CATCAGGTAA AAGGTGGTTA TTTGATATTG AATTGCAGGA ATCAGGGTG- 8692
Darwin bee virus 3 Australia 2014 NT-12 (MG995697) AGAAAGGTAA GAGATGGCTC TTTAAGATGG AA----GAGA CTCAGAATGG 8372
Consensus CTTCTGGTAA GCGATGGTTG TTTGATATTG AATTACAAGA TTCAGGATG-
100%
Conservation
0%
8.860 8.880 8.900
DWV-B Austria 2022 SB22 (PP418870) TTATCTTTTG AGAGGGATGA GACCTGAACT TGAGA-TACA GTTGACAACA 8723
DWV-B USA 2018 EVR24 (OR361560) TTATCTTTTG AGAGGGATGA GACCTGAACT TGAGA-TACA GTTGACAACA 8723
DWV-B USA 2018 EVR23 (OR361559) TTATCTTTTG AGAGGGATGA GACCTGAACT TGAGA-TACA GTTGACAACA 8723
DWV-B Netherlands 2001 VDV-1 (NC_006494) TTATCTTTTG AGAGGGATGA GACCTGAACT TGAGA-TACA GTTGACAACA 8722
DWV-B France 2019 VV144I (MN565037) TTATCTTTTG AGAGGGATGA GACCTGAACT TGAGA-TACA GTTGACAACA 8723
DWV-B Slovenia 2019 341-1 (ON648741) TTATCTTTTG AGAGGGATGA GACCTGAACT TGAGA-TACA GTTGACAACA 8723
DWV-B Austria 2018 B11B (OL803829) TTATCTTTTG AGAGGGATGA GACCTGAACT TGAGA-TACA GTTGACAACA 8723
DWV-A Austria 2014 1414 (KU847397) TTATCTCCTG CGTGGAATGC GACCCGAACT TGAGA-TTCA ATTATCAACG 8738
DWV-A USA 2003 PA (AY292384) TTATCTCCTG CGTGGAATGC GTCCCGAACT TGAGA-TTCA ATTATCAACG 8736
DWV-A Japan 2001 Kakugo (AB070959) TTATCTCTTG CGTGGAATGC GTCCCGAACT TGAGA-TTCA ATTATCAACG 8721
DWV-C UK 2015 Devon (ERS657949) TTATCTTTTA CGAGGTATGC GTCCCGAATT AGAAA-TACA ATTGTCAACC 8711
DWV-D Egypt 1977 EBV (MT504363) TTATCTTTTG AGAGGTATGC GCCCCGAGGT TGAAA-TCCA ATTGTCAACT 8741
Darwin bee virus 3 Australia 2014 NT-12 (MG995697) CTATAAACTC CTATCTATGA GACAGGAGTT AAAATCTATG ATTGAGACTC 8422
Consensus TTATCTTTTG AGAGGGATGA GACCTGAACT TGAGA-TACA GTTGACAACA
100%
Conservation
0%
8.920 8.940
DWV-B Austria 2022 SB22 (PP418870) ACTCAGTTGA TGAGGAAGAA GGGAATAAAG CCTCACACTA TATTCACGGA 8773
DWV-B USA 2018 EVR24 (OR361560) ACTCAGTTAA TGAGGAAGAA GGGAATAAAG CCTCACACTA TATTCACGGA 8773
DWV-B USA 2018 EVR23 (OR361559) ACTCAGTTAA TGAGGAAGAA GGGAATAAAG CCTCACACTA TATTCACGGA 8773
DWV-B Netherlands 2001 VDV-1 (NC_006494) ACTCAGTTAA TGAGGAAGAA GGGAATGAAG CCTCACACTA TATTCACGGA 8772
DWV-B France 2019 VV144I (MN565037) ACTCAGTTAA TGAGGAAGAA GGGAATAAAG CCTCACACTA TATTCACGGA 8773
DWV-B Slovenia 2019 341-1 (ON648741) ACTCAGTTAA TGAGGAAGAA GGGAATAAAG CCTCACACTA TATTCACGGA 8773
DWV-B Austria 2018 B11B (OL803829) ACTCAGTTAA TGAGGAAGAA GGGAATAAAG CCTCACACTA TATTCACGGA 8773
DWV-A Austria 2014 1414 (KU847397) ACACAGTTAA TGAGGAAAAA GGGAATAAAG CCTCACACTA TATTCACGGA 8788
DWV-A USA 2003 PA (AY292384) ACACAGTTAA TGAGGAAAAA GGGAATAAAA CCTCACACTA TATTCACGGA 8786
DWV-A Japan 2001 Kakugo (AB070959) ACACAGTTAA TGAGGAAAAA GGGAATAAAA CCTCACACTA TATTCACGGA 8771
DWV-C UK 2015 Devon (ERS657949) ACGCAACTTA TGAGGAAAAA GGGAATAAAG CCACATACCA TTTTTACAGA 8761
DWV-D Egypt 1977 EBV (MT504363) ACGCAGTTAA TGAGGAAGAA AGGAATAAAA CCGCATACTA TCTTTACGGA 8791
Darwin bee virus 3 Australia 2014 NT-12 (MG995697) ATATGGA-GA TGAGGAGAAG AGGTATTAAA CCACAAACTC TTTTCTCTGA 8471
Consensus ACTCAGTTAA TGAGGAAGAA GGGAATAAAG CCTCACACTA TATTCACGGA
100%
Conservation
0%
8.960 8.980 9.000
DWV-B Austria 2022 SB22 (PP418870) TTGTTTGAAA GATACATGTT TGCCTGTGGA AAAATGCAGA ATACCTGGTA 8823
DWV-B USA 2018 EVR24 (OR361560) TTGTTTGAAA GATACATGTT TGCCTGTGGA AAAATGCAGA ATACCTGGTA 8823
DWV-B USA 2018 EVR23 (OR361559) TTGTTTGAAA GATACATGTT TGCCTGTGGA AAAATGCAGA ATACCTGGTA 8823
DWV-B Netherlands 2001 VDV-1 (NC_006494) TTGTTTGAAA GATACATGTT TGCCTGTGGA AAAATGCAGA ATACCTGGTA 8822
DWV-B France 2019 VV144I (MN565037) TTGTTTGAAA GATACATGTT TGCCTGTGGA AAAATGCAGA ATACCTGGTA 8823
DWV-B Slovenia 2019 341-1 (ON648741) TTGTTTGAAA GATACATGTT TGCCTGTGGA AAAATGCAGA ATACCTGGTA 8823
DWV-B Austria 2018 B11B (OL803829) TTGTTTGAAA GATACATGTT TGCCTGTGGA AAAATGCAGA ATACCTGGTA 8823
DWV-A Austria 2014 1414 (KU847397) TTGTTTGAAA GATACTTGTT TGCCTGTTGA AAAATGTAGA ATACCTGGTA 8838
DWV-A USA 2003 PA (AY292384) TTGTTTGAAG GATACTTGTT TGCCTGTTGA AAAATGTAGA ATACCTGGTA 8836
DWV-A Japan 2001 Kakugo (AB070959) TTGTTTGAAA GATACTTGTT TGCCTGTAGA AAAATGTAGA ATACCTGGTA 8821
DWV-C UK 2015 Devon (ERS657949) TTGTTTGAAA GATACATGCT TGCCTGTAGA AAAATGTAGA ATTCCTGGTA 8811
DWV-D Egypt 1977 EBV (MT504363) TTGTTTGAAA GATACATGCT TACCTGTAGA AAAATGTAGA ATTCCTGGTA 8841
Darwin bee virus 3 Australia 2014 NT-12 (MG995697) TTGTCTTAAA GATACATGTT TACCTGTCGA AAAGTGTAAA ATACCTGGAA 8521
Consensus TTGTTTGAAA GATACATGTT TGCCTGTGGA AAAATGCAGA ATACCTGGTA
100%
Conservation
0%

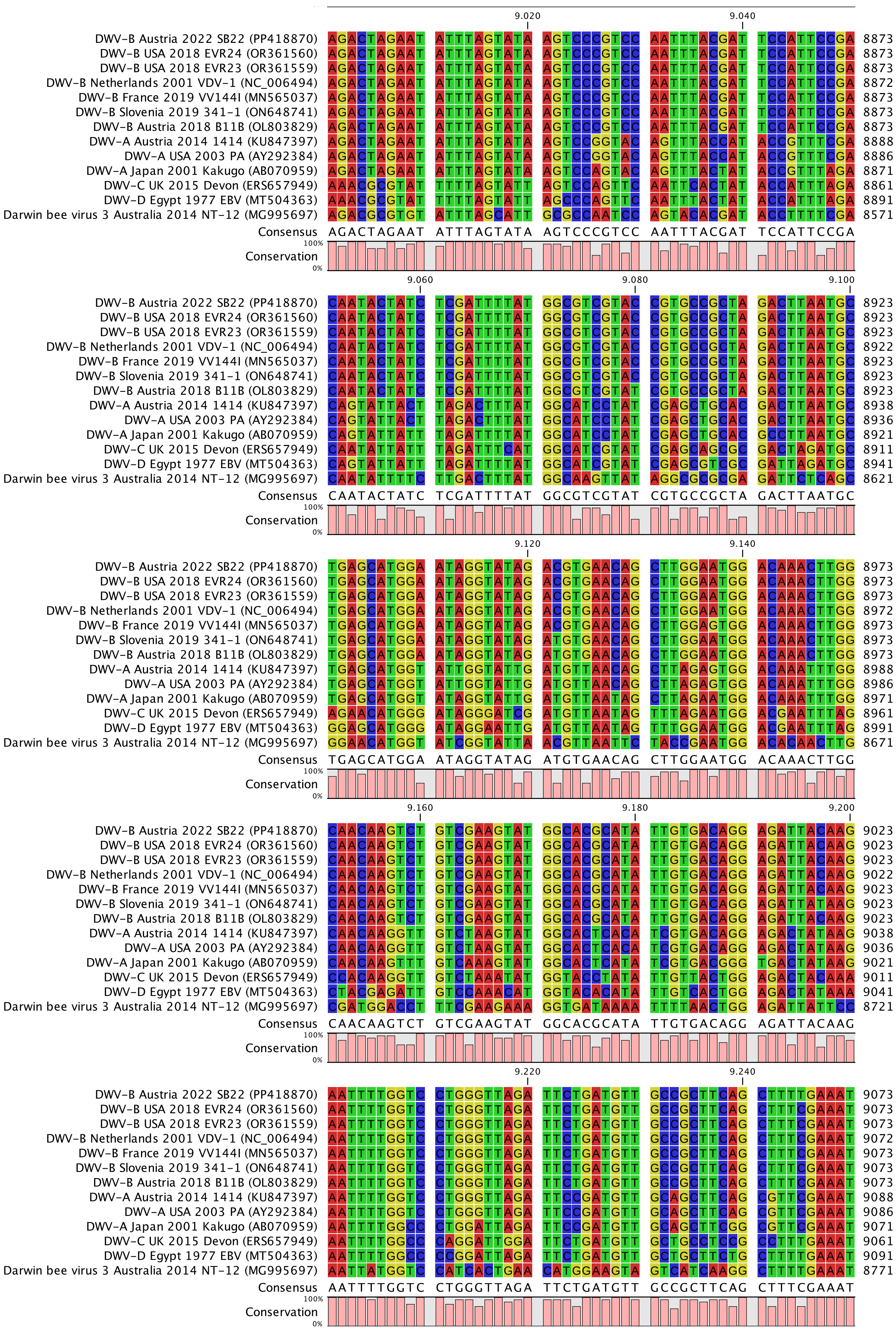

DWV-B Austria 2022 SB22 (PP418870)
DWV-B USA 2018 EVR24 (OR361560)
DWV-B USA 2018 EVR23 (OR361559)
DWV-B Netherlands 2001 VDV-1 (NC_006494)
DWV-B France 2019 VV144I (MN565037)
DWV-B Slovenia 2019 341-1 (ON648741)
DWV-B Austria 2018 B11B (OL803829)
DWV-A Austria 2014 1414 (KU847397)
DWV-A USA 2003 PA (AY292384)
DWV-A Japan 2001 Kakugo (AB070959)
DWV-C UK 2015 Devon (ERS657949)
DWV-D Egypt 1977 EBV (MT504363)
Darwin bee virus 3 Australia 2014 NT-12 (MG995697)
Consensus
Conservation
100%
0%
9.020
9.040
8873 8873 8873 8872 8873 8873 8873 8888 8886 8871 8861 8891 8571
AGACTAGAAT ATTTAGTATA AGTCCCGTCC AATTTACGAT TCCATTCCGA
9.060
9.080
9.100
8923 8923 8923 8922 8923 8923 8923 8938 8936 8921 8911 8941 8621
CAATACTATC TCGATTTTAT GGCGTCGTAT CGTGCCGCTA GACTTAATGC
9.120
9.140
8973 8973 8973 8972 8973 8973 8973 8988 8986 8971 8961 8991 8671
TGAGCATGGA ATAGGTATAG ATGTGAACAG CTTGGAATGG ACAAACTTGG
9.160
9.180
9.200
9023 9023 9023 9022 9023 9023 9023 9038 9036 9021 9011 9041 8721
CAACAAGTCT GTCGAAGTAT GGCACGCATA TTGTGACAGG AGATTACAAG
9.220
9.240
9073 9073 9073 9072 9073 9073 9073 9088 9086 9071 9061 9091 8771
AATTTTGGTC CTGGGTTAGA TTCTGATGTT GCCGCTTCAG CTTTCGAAAT

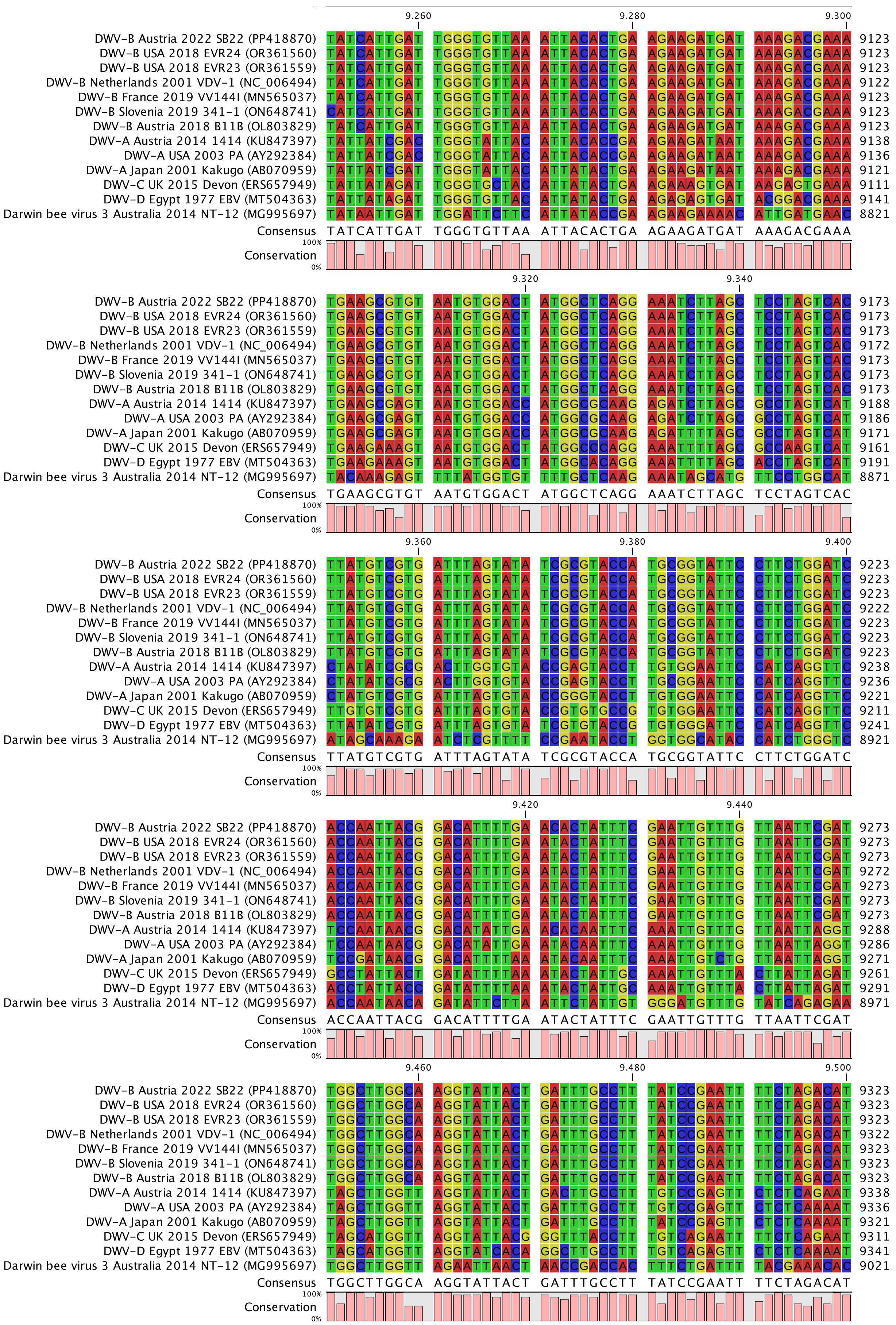

DWV-B Austria 2022 SB22 (PP418870)
DWV-B USA 2018 EVR24 (OR361560)
DWV-B USA 2018 EVR23 (OR361559)
DWV-B Netherlands 2001 VDV-1 (NC_006494)
DWV-B France 2019 VV1441 (MN565037)
DWV-B Slovenia 2019 341-1 (ON648741)
DWV-B Austria 2018 B11B (OL803829)
DWV-A Austria 2014 1414 (KU847397)
DWV-A USA 2003 PA (AY292384)
DWV-A Japan 2001 Kakugo (AB070959)
DWV-C UK 2015 Devon (ERS657949)
DWV-D Egypt 1977 EBV (MT504363)
Darwin bee virus 3 Australia 2014 NT-12 (MG995697)
9.260
9.280
9.300
Consensus TATCATTGAT TGGGTGTTAA ATTACACTGA AGAAGATGAT AAAGACGAAA
9.320
9.340
Consensus TGAAGCGTGT AATGTGGACT ATGGCTCAGG AAATCTTAGC TCCTAGTCAC
9.360
9.380
9.400
Consensus TTATGTCGTG ATTTAGTATA TCGCGTACCA TGCGGTATTC CTTCTGGATC
9.420
9.440
Consensus ACCAATTACG GACATTTTGA ATACTATTTC GAATTGTTTG TTAATTCGAT
9.460
9.480
9.500
Consensus TGGCTTGGCA AGGTATTACT GATTTGCCTT TATCCGAATT TTCTAGACAT
Conservation
100%
0%

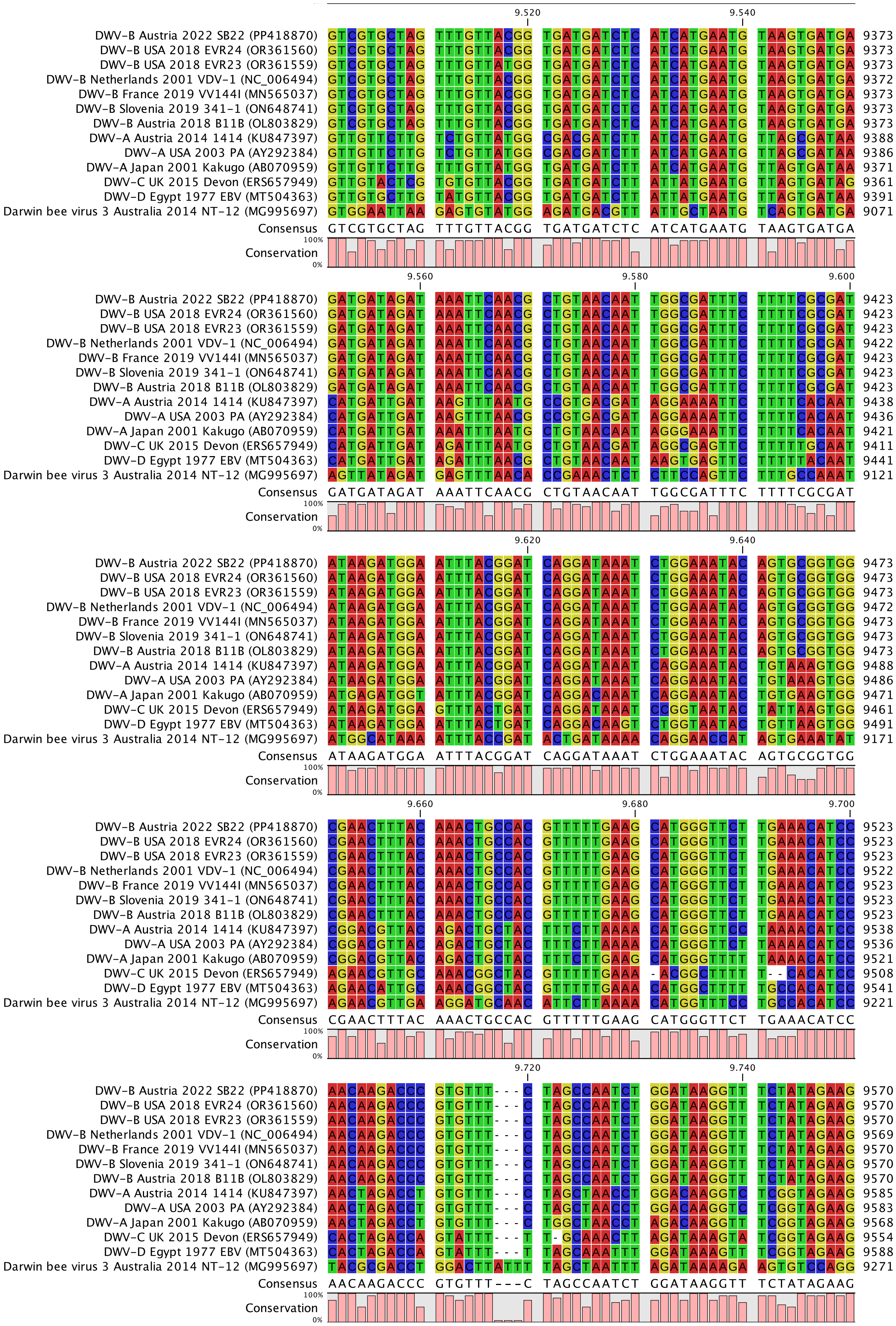
9.520
9.540
DWV-B Austria 2022 SB22 (PP418870) GTCGTGCTAG TTTGTTACGG TGATGATCTC ATCATGAATG TAAGTGATGA 9373
DWV-B USA 2018 EVR24 (OR361560) GTCGTGCTAG TTTGTTACGG TGATGATCTC ATCATGAATG TAAGTGATGA 9373
DWV-B USA 2018 EVR23 (OR361559) GTCGTGCTAG TTTGTTATGG TGATGATCTC ATCATGAATG TAAGTGATGA 9373
DWV-B Netherlands 2001 VDV-1 (NC_006494) GTCGTGCTAG TTTGTTACGG TGATGATCTC ATCATGAATG TAAGTGATGA 9372
DWV-B France 2019 VV144I (MN565037) GTCGTGCTAG TTTGTTACGG TGATGATCTC ATCATGAATG TAAGTGATGA 9373
DWV-B Slovenia 2019 341-1 (ON648741) GTCGTGCTAG TTTGTTACGG TGATGATCTC ATCATGAATG TAAGTGATGA 9373
DWV-B Austria 2018 B11B (OL803829) GTCGTGCTAG TTTGTTACGG TGATGATCTC ATCATGAATG TAAGTGATGA 9373
DWV-A Austria 2014 1414 (KU847397) GTTGTTCTTG TCTGTTATGG CGACGATCTT ATCATGAATG TTAGCGATAA 9388
DWV-A USA 2003 PA (AY292384) GTTGTTCTTG TCTGTTATGG CGACGATCTT ATCATGAATG TTAGCGATAA 9386
DWV-A Japan 2001 Kakugo (AB070959) GTTGTTCTTG TTTGTTATGG TGATGATCTT ATCATGAATG TTAGTGATAA 9371
DWV-C UK 2015 Devon (ERS657949) GTTGTACTCG TGTGTTACGG TGATGATCTT ATTATGAATG TTAGTGATAG 9361
DWV-D Egypt 1977 EBV (MT504363) GTTGTGCTTG TATGTTACGG TGATGATCTT ATTATGAATG TTAGTGATAA 9391
Darwin bee virus 3 Australia 2014 NT-12 (MG995697) GTGGAATTAA GAGTGTATGG AGATGACGTT ATTGCTAATG TCAGTGATGA 9071
Consensus GTCGTGCTAG TTTGTTACGG TGATGATCTC ATCATGAATG TAAGTGATGA
100%
Conservation
0%
9.560
9.580
9.600
DWV-B Austria 2022 SB22 (PP418870) GATGATAGAT AAATTCAACG CTGTAACAAT TGGCGATTTC TTTTCGCGAT 9423
DWV-B USA 2018 EVR24 (OR361560) GATGATAGAT AAATTCAACG CTGTAACAAT TGGCGATTTC TTTTCGCGAT 9423
DWV-B USA 2018 EVR23 (OR361559) GATGATAGAT AAATTCAACG CTGTAACAAT TGGCGATTTC TTTTCGCGAT 9423
DWV-B Netherlands 2001 VDV-1 (NC_006494) GATGATAGAT AAATTCAACG CTGTAACAAT TGGCGATTTC TTTTCGCGAT 9422
DWV-B France 2019 VV144I (MN565037) GATGATAGAT AAATTCAACG CTGTAACAAT TGGCGATTTC TTTTCGCGAT 9423
DWV-B Slovenia 2019 341-1 (ON648741) GATGATAGAT AAATTCAACG CTGTAACAAT TGGCGATTTC TTTTCGCGAT 9423
DWV-B Austria 2018 B11B (OL803829) GATGATAGAT AAATTCAACG CTGTAACAAT TGGCGATTTC TTTTCGCGAT 9423
DWV-A Austria 2014 1414 (KU847397) CATGATTGAT AAGTTTAATG CCGTGACGAT AGGAAAATTC TTTTCACAAT 9438
DWV-A USA 2003 PA (AY292384) CATGATTGAT AAGTTTAACG CCGTGACGAT AGGAAAATTC TTTTCACAAT 9436
DWV-A Japan 2001 Kakugo (AB070959) CATGATTGAT AAATTTAATG CTGTAACAAT AGGGAAATTC TTTTCACAAT 9421
DWV-C UK 2015 Devon (ERS657949) CATGATTGAT AGATTTAATG CTGTAACGAT AGGCGAGTTC TTTTTGCAAT 9411
DWV-D Egypt 1977 EBV (MT504363) CATGATTGAT AGATTTAACG CTGTAACAAT AAGTGAGTTC TTTTTACAAT 9441
Darwin bee virus 3 Australia 2014 NT-12 (MG995697) AGTTATAGAT GAGTTTAACA CCGAAACTCT CTTCCAGTTC TTTGCCAAAT 9121
Consensus GATGATAGAT AAATTCAACG CTGTAACAAT TGGCGATTTC TTTTCGCGAT
100%
Conservation
0%
9.620
9.640
DWV-B Austria 2022 SB22 (PP418870) ATAAGATGGA ATTTACGGAT CAGGATAAAT CTGGAAATAC AGTGCGGTGG 9473
DWV-B USA 2018 EVR24 (OR361560) ATAAGATGGA ATTTACGGAT CAGGATAAAT CTGGAAATAC AGTGCGGTGG 9473
DWV-B USA 2018 EVR23 (OR361559) ATAAGATGGA ATTTACGGAT CAGGATAAAT CTGGAAATAC AGTGCGGTGG 9473
DWV-B Netherlands 2001 VDV-1 (NC_006494) ATAAGATGGA ATTTACGGAT CAGGATAAAT CTGGAAATAC AGTGCGGTGG 9472
DWV-B France 2019 VV144I (MN565037) ATAAGATGGA ATTTACGGAT CAGGATAAAT CTGGAAATAC AGTGCGGTGG 9473
DWV-B Slovenia 2019 341-1 (ON648741) ATAAGATGGA ATTTACGGAT CAGGATAAAT CTGGAAATAC AGTGCGGTGG 9473
DWV-B Austria 2018 B11B (OL803829) ATAAGATGGA ATTTACGGAT CAGGATAAAT CTGGAAATAC AGTGCGGTGG 9473
DWV-A Austria 2014 1414 (KU847397) ATAAGATGGA ATTTACGGAT CAGGATAAAT CAGGAAATAC TGTAAAGTGG 9488
DWV-A USA 2003 PA (AY292384) ATAAGATGGA ATTTACGGAT CAGGATAAAT CAGGAAATAC TGTAAAGTGG 9486
DWV-A Japan 2001 Kakugo (AB070959) ATGAGATGGT ATTTACGGAT CAGGACAAAT CAGGAAATAC TGTGAAGTGG 9471
DWV-C UK 2015 Devon (ERS657949) ATAAGATGGA GTTTACTGAT CAGGATAAAT CCGGTAATAC TATTAAGTGG 9461
DWV-D Egypt 1977 EBV (MT504363) ATAAGATGGA ATTTACTGAT CAGGACAAGT CTGGTAATAC TGTTAAGTGG 9491
Darwin bee virus 3 Australia 2014 NT-12 (MG995697) ATGGCATAAA ATTTACCGAT ACTGATAAAA CAGGAACCAT AGTGAAATAT 9171
Consensus ATAAGATGGA ATTTACGGAT CAGGATAAAT CTGGAAATAC AGTGCGGTGG
100%
Conservation
0%
9.660
9.680
9.700
DWV-B Austria 2022 SB22 (PP418870) CGAACTTTAC AAACTGCCAC GTTTTTGAAG CATGGGTTCT TGAAACATCC 9523
DWV-B USA 2018 EVR24 (OR361560) CGAACTTTAC AAACTGCCAC GTTTTTGAAG CATGGGTTCT TGAAACATCC 9523
DWV-B USA 2018 EVR23 (OR361559) CGAACTTTAC AAACTGCCAC GTTTTTGAAG CATGGGTTCT TGAAACATCC 9523
DWV-B Netherlands 2001 VDV-1 (NC_006494) CGAACTTTAC AAACTGCCAC GTTTTTGAAG CATGGGTTCT TGAAACATCC 9522
DWV-B France 2019 VV144I (MN565037) CGAACTTTAC AAACTGCCAC GTTTTTGAAG CATGGGTTCT TGAAACATCC 9523
DWV-B Slovenia 2019 341-1 (ON648741) CGAACTTTAC AAACTGCCAC GTTTTTGAAG CATGGGTTCT TGAAACATCC 9523
DWV-B Austria 2018 B11B (OL803829) CGAACTTTAC AAACTGCCAC GTTTTTGAAG CATGGGTTCT TGAAACATCC 9523
DWV-A Austria 2014 1414 (KU847397) CGGACGTTAC AGACTGCTAC TTTCTTAAAA CATGGGTTCC TAAAACATCC 9538
DWV-A USA 2003 PA (AY292384) CGGACGTTAC AGACTGCTAC TTTCTTAAAA CATGGGTTCT TAAAACATCC 9536
DWV-A Japan 2001 Kakugo (AB070959) CGGACGTTAC AGACTGCTAC TTTCTTGAAG CATGGGTTTT TAAAACATCC 9521
DWV-C UK 2015 Devon (ERS657949) AGAACGTTGC AAACGGCTAC GTTTTTGAAA -ACGGCTTTT T--CACATCC 9508
DWV-D Egypt 1977 EBV (MT504363) AGAACATTGC AAACGGCTAC GTTTTTGAAA CATGGCTTTT TGCCACATCC 9541
Darwin bee virus 3 Australia 2014 NT-12 (MG995697) AGAACGTTGA AGGATGCAAC ATTCTTAAAA CATGGTTTCC TGCCACATCC 9221
Consensus CGAACTTTAC AAACTGCCAC GTTTTTGAAG CATGGGTTCT TGAAACATCC
100%
Conservation
0%
9.720
9.740
DWV-B Austria 2022 SB22 (PP418870) AACAAGACCC GTGTTT---C TAGCCAATCT GGATAAGGTT TCTATAGAAG 9570
DWV-B USA 2018 EVR24 (OR361560) AACAAGACCC GTGTTT---C TAGCCAATCT GGATAAGGTT TCTATAGAAG 9570
DWV-B USA 2018 EVR23 (OR361559) AACAAGACCC GTGTTT---C TAGCCAATCT GGATAAGGTT TCTATAGAAG 9570
DWV-B Netherlands 2001 VDV-1 (NC_006494) AACAAGACCC GTGTTT---C TAGCCAATCT GGATAAGGTT TCTATAGAAG 9569
DWV-B France 2019 VV144I (MN565037) AACAAGACCC GTGTTT---C TAGCCAATCT GGATAAGGTT TCTATAGAAG 9570
DWV-B Slovenia 2019 341-1 (ON648741) AACAAGACCC GTGTTT---C TAGCCAATCT GGATAAGGTT TCTATAGAAG 9570
DWV-B Austria 2018 B11B (OL803829) AACAAGACCC GTGTTT---C TAGCCAATCT GGATAAGGTT TCTATAGAAG 9570
DWV-A Austria 2014 1414 (KU847397) AACTAGACCT GTGTTT---C TAGCTAACCT GGACAAGGTC TCGGTAGAAG 9585
DWV-A USA 2003 PA (AY292384) AACTAGACCT GTGTTT---C TAGCTAACCT GGACAAGGTC TCGGTAGAAG 9583
DWV-A Japan 2001 Kakugo (AB070959) AACTAGACCT GTGTTT---C TGGCTAACCT AGACAAGGTT TCGGTAGAAG 9568
DWV-C UK 2015 Devon (ERS657949) CACTAGACCA GTATTT---T T-GCAAACTT AGATAAAGTA TCGGTAGAAG 9554
DWV-D Egypt 1977 EBV (MT504363) CACTAGACCA GTATTT---T TAGCAAATTT GGATAAAGTT TCGGTAGAAG 9588
Darwin bee virus 3 Australia 2014 NT-12 (MG995697) TACGCGACCT GGACTTATTT TAGCTAATTT AGATAAAAGA AGTGTCCAGG 9271
Consensus AACAAGACCC GTGTTT---C TAGCCAATCT GGATAAGGTT TCTATAGAAG
100%
Conservation
0%

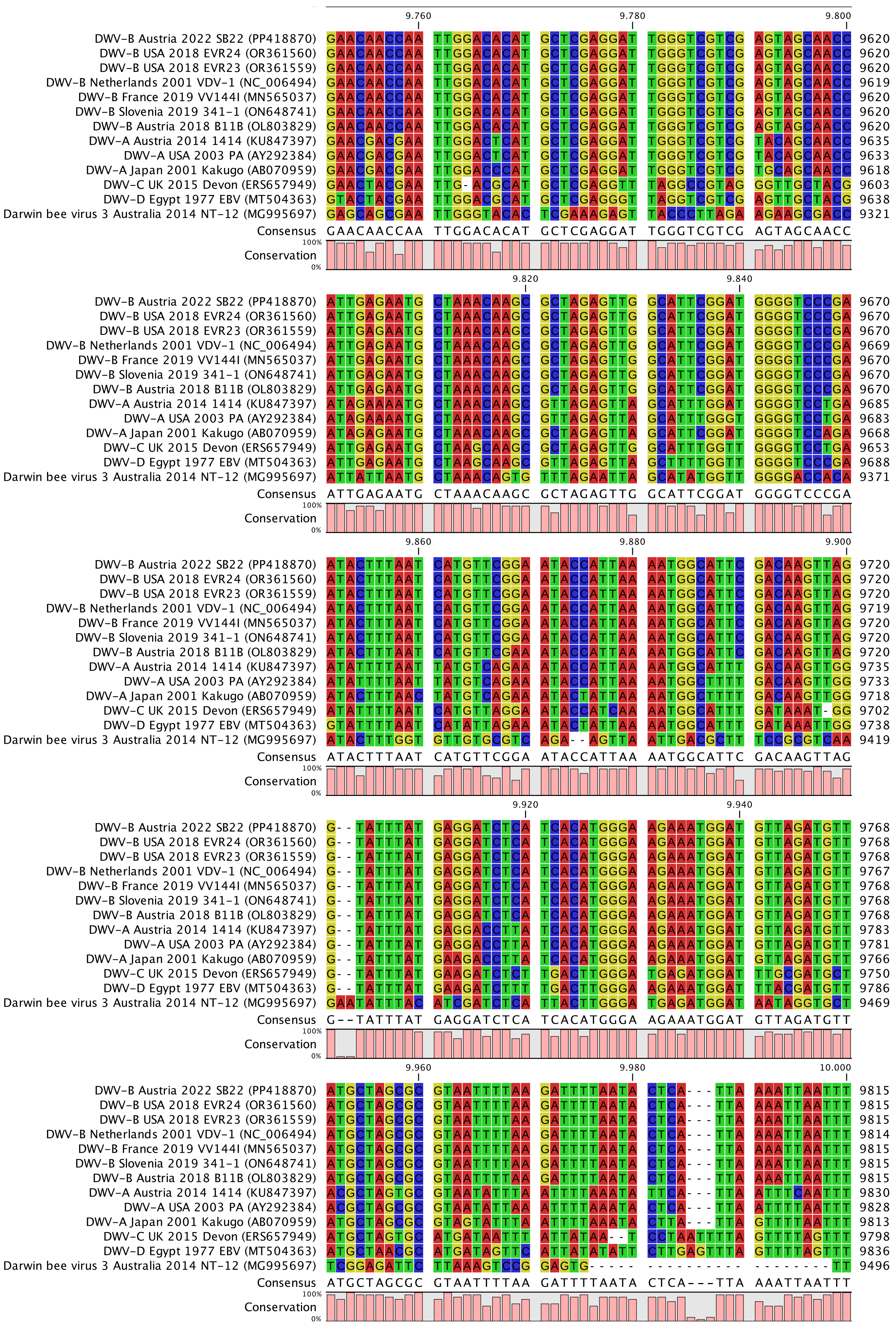
9.760 9.780 9.800
DWV-B Austria 2022 SB22 (PP418870)
DWV-B USA 2018 EVR24 (OR361560)
DWV-B USA 2018 EVR23 (OR361559)
DWV-B Netherlands 2001 VDV-1 (NC_006494)
DWV-B France 2019 VV1441 (MN565037)
DWV-B Slovenia 2019 341-1 (ON648741)
DWV-B Austria 2018 B11B (OL803829)
DWV-A Austria 2014 1414 (KU847397)
DWV-A USA 2003 PA (AY292384)
DWV-A Japan 2001 Kakugo (AB070959)
DWV-C UK 2015 Devon (ERS657949)
DWV-D Egypt 1977 EBV (MT504363)
Darwin bee virus 3 Australia 2014 NT-12 (MG995697)
9620 9620 9620 9619 9620 9620 9620 9635 9633 9618 9603 9638 9321
Consensus GAACAACCAA TTGGACACAT GCTCGAGGAT TGGGTCGTCG AGTAGCAACC
Conservation 100% 0%
9.820 9.840
9670 9670 9670 9669 9670 9670 9670 9685 9683 9668 9653 9688 9371
Consensus ATTGAGAATG CTAAACAAGC GCTAGAGTTG GCATTCGGAT GGGGTCCCGA
9.860 9.880 9.900
9720 9720 9720 9719 9720 9720 9720 9735 9733 9718 9702 9738 9419
Consensus ATACTTTAAT CATGTTCGGA ATACCATTAA AATGGCATTC GACAAGTTAG
9.920 9.940
9768 9768 9768 9767 9768 9768 9768 9783 9781 9766 9750 9786 9469
Consensus G--TATTTAT GAGGATCTCA TCACATGGGA AGAAATGGAT GTTAGATGTT
9.960 9.980 10.000
9815 9815 9815 9814 9815 9815 9815 9830 9828 9813 9798 9836 9496
Consensus ATGCTAGCGC GTAATTTTAA GATTTTAATA CTCA---TTA AAATTAATTT

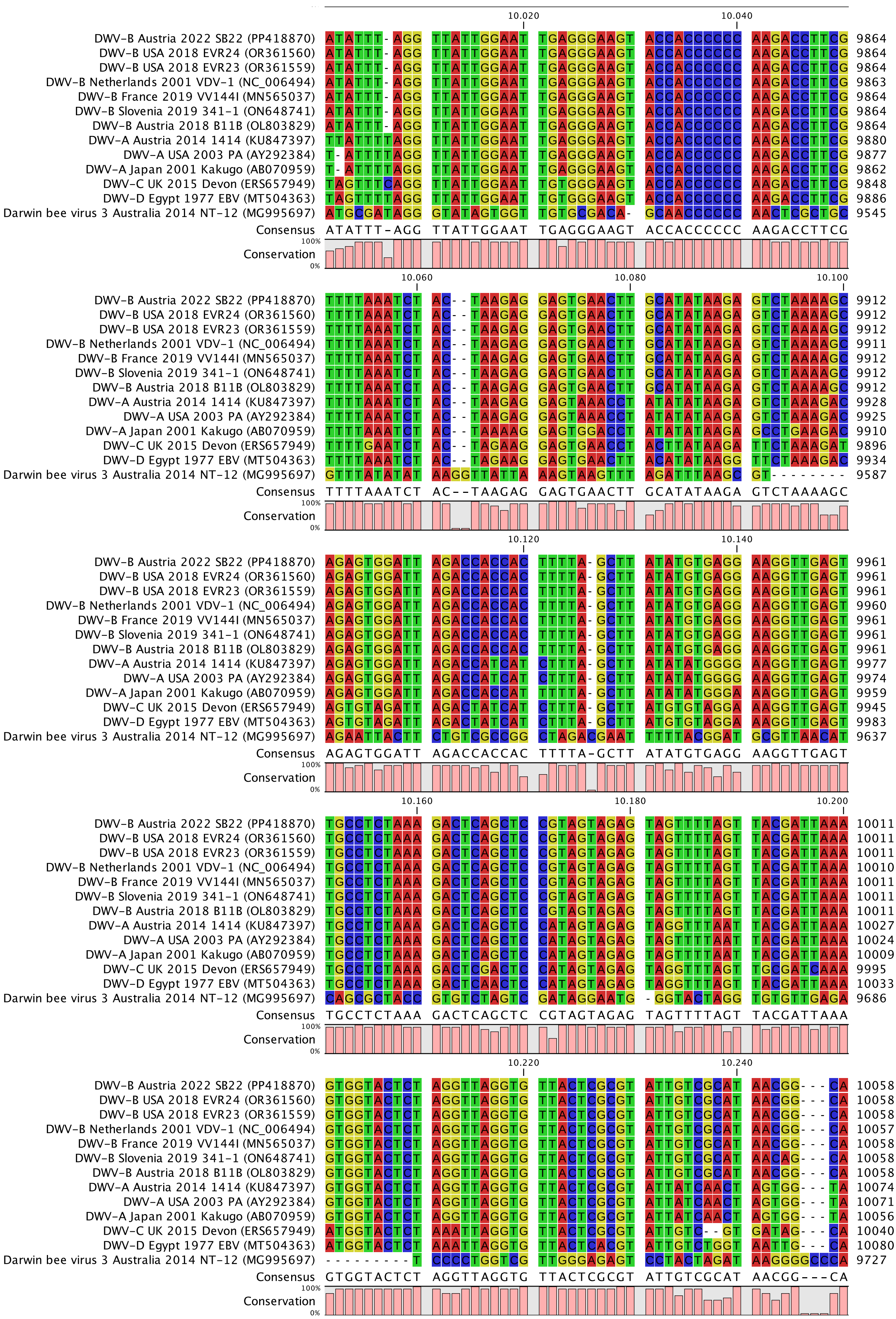
10.020 10.040
DWV-B Austria 2022 SB22 (PP418870) ATATTT-AGG TTATTGGAAT TGAGGGAAGT ACCACCCCCC AAGACCTTCG 9864
DWV-B USA 2018 EVR24 (OR361560) ATATTT-AGG TTATTGGAAT TGAGGGAAGT ACCACCCCCC AAGACCTTCG 9864
DWV-B USA 2018 EVR23 (OR361559) ATATTT-AGG TTATTGGAAT TGAGGGAAGT ACCACCCCCC AAGACCTTCG 9864
DWV-B Netherlands 2001 VDV-1 (NC_006494) ATATTT-AGG TTATTGGAAT TGAGGGAAGT ACCACCCCCC AAGACCTTCG 9863
DWV-B France 2019 VV144I (MN565037) ATATTT-AGG TTATTGGAAT TGAGGGAAGT ACCACCCCCC AAGACCTTCG 9864
DWV-B Slovenia 2019 341-1 (ON648741) ATATTT-AGG TTATTGGAAT TGAGGGAAGT ACCACCCCCC AAGACCTTCG 9864
DWV-B Austria 2018 B11B (OL803829) ATATTT-AGG TTATTGGAAT TGAGGGAAGT ACCACCCCCC AAGACCTTCG 9864
DWV-A Austria 2014 1414 (KU847397) TTATTTTAGG TTATTGGAAT TGAGGGAAGT ACCACCCCCC AAGACCTTCG 9880
DWV-A USA 2003 PA (AY292384) T-ATTTTAGG TTATTGGAAT TGAGGGAAGT ACCACCCCCC AAGACCTTCG 9877
DWV-A Japan 2001 Kakugo (AB070959) T-ATTTTAGG TTATTGGAAT TGAGGGAAGT ACCACCCCCC AAGACCTTCG 9862
DWV-C UK 2015 Devon (ERS657949) TAGTTTCAGG TTATTGGAAT TGTGGGAAGT ACCACCCCCC AAGACCTTCG 9848
DWV-D Egypt 1977 EBV (MT504363) TAGTTTTAGG TTATTGGAAT TGTGGGAAGT ACCACCCCCC AAGACCTTCG 9886
Darwin bee virus 3 Australia 2014 NT-12 (MG995697) ATGCGATAGG GTATAGTGGT TGTGCGACA- GCAACCCCCC AACTCGCTGC 9545
Consensus ATATTT-AGG TTATTGGAAT TGAGGGAAGT ACCACCCCCC AAGACCTTCG
100%
Conservation
0%
10.060 10.080 10.100
DWV-B Austria 2022 SB22 (PP418870) TTTTAAATCT AC--TAAGAG GAGTGAACTT GCATATAAGA GTCTAAAAGC 9912
DWV-B USA 2018 EVR24 (OR361560) TTTTAAATCT AC--TAAGAG GAGTGAACTT GCATATAAGA GTCTAAAAGC 9912
DWV-B USA 2018 EVR23 (OR361559) TTTTAAATCT AC--TAAGAG GAGTGAACTT GCATATAAGA GTCTAAAAGC 9912
DWV-B Netherlands 2001 VDV-1 (NC_006494) TTTTAAATCT AC--TAAGAG GAGTGAACTT GCATATAAGA GTCTAAAAGC 9911
DWV-B France 2019 VV144I (MN565037) TTTTAAATCT AC--TAAGAG GAGTGAACTT GCATATAAGA GTCTAAAAGC 9912
DWV-B Slovenia 2019 341-1 (ON648741) TTTTAAATCT AC--TAAGAG GAGTGAACTT GCATATAAGA GTCTAAAAGC 9912
DWV-B Austria 2018 B11B (OL803829) TTTTAAATCT AC--TAAGAG GAGTGAACTT GCATATAAGA GTCTAAAAGC 9912
DWV-A Austria 2014 1414 (KU847397) TTTTAAATCT AC--TAAGAG GAGTAAACCT ATATATAAGA GTCTAAAGAC 9928
DWV-A USA 2003 PA (AY292384) TTTTAAATCT AC--TAAGAG GAGTAAACCT ATATATAAGA GTCTAAAGAC 9925
DWV-A Japan 2001 Kakugo (AB070959) TTTTAAATCT AC--TAAAAG GAGTGGACCT ATATATAAGA GCCTGAAGAC 9910
DWV-C UK 2015 Devon (ERS657949) TTTTGAATCT AC--TAGAAG GAGTGAACCT ACTTATAAGA TTCTAAAGAT 9896
DWV-D Egypt 1977 EBV (MT504363) TTTTAAATCT AC--TAGAAG GAGTGAACTT ACATATAAGG TTCTAAAGAC 9934
Darwin bee virus 3 Australia 2014 NT-12 (MG995697) GTTTATATAT AAGGTTATTA AAGTAAGTTT AGATTTAAGC GT-------- 9587
Consensus TTTTAAATCT AC--TAAGAG GAGTGAACTT GCATATAAGA GTCTAAAAGC
100%
Conservation
0%
10.120 10.140
DWV-B Austria 2022 SB22 (PP418870) AGAGTGGATT AGACCACCAC TTTTA-GCTT ATATGTGAGG AAGGTTGAGT 9961
DWV-B USA 2018 EVR24 (OR361560) AGAGTGGATT AGACCACCAC TTTTA-GCTT ATATGTGAGG AAGGTTGAGT 9961
DWV-B USA 2018 EVR23 (OR361559) AGAGTGGATT AGACCACCAC TTTTA-GCTT ATATGTGAGG AAGGTTGAGT 9961
DWV-B Netherlands 2001 VDV-1 (NC_006494) AGAGTGGATT AGACCACCAC TTTTA-GCTT ATATGTGAGG AAGGTTGAGT 9960
DWV-B France 2019 VV144I (MN565037) AGAGTGGATT AGACCACCAC TTTTA-GCTT ATATGTGAGG AAGGTTGAGT 9961
DWV-B Slovenia 2019 341-1 (ON648741) AGAGTGGATT AGACCACCAC TTTTA-GCTT ATATGTGAGG AAGGTTGAGT 9961
DWV-B Austria 2018 B11B (OL803829) AGAGTGGATT AGACCACCAC TTTTA-GCTT ATATGTGAGG AAGGTTGAGT 9961
DWV-A Austria 2014 1414 (KU847397) AGAGTGGATT AGACCATCAT CTTTA-GCTT ATATATGGGG AAGGTTGAGT 9977
DWV-A USA 2003 PA (AY292384) AGAGTGGATT AGACCATCAT CTTTA-GCTT ATATATGGGG AAGGTTGAGT 9974
DWV-A Japan 2001 Kakugo (AB070959) AGAGTGGATT AGACCACCAT TTTTA-GCTT ATATATGGGA AAGGTTGAGT 9959
DWV-C UK 2015 Devon (ERS657949) AGTGTAGATT AGACTATCAT CTTTA-GCTT ATGTGTAGGA AAGGTTGAGT 9945
DWV-D Egypt 1977 EBV (MT504363) AGTGTAGATT AGACTATCAT CTTTA-GCTT ATGTGTAGGA AAGGTTGAGT 9983
Darwin bee virus 3 Australia 2014 NT-12 (MG995697) AGAATTACTT CTGTCGCCGG CTAGACGAAT TTTTACGGAT GCGTTAACAT 9637
Consensus AGAGTGGATT AGACCACCAC TTTTA-GCTT ATATGTGAGG AAGGTTGAGT
100%
Conservation
0%
10.160 10.180 10.200
DWV-B Austria 2022 SB22 (PP418870) TGCCTCTAAA GACTCAGCTC CGTAGTAGAG TAGTTTTAGT TACGATTAAA 10011
DWV-B USA 2018 EVR24 (OR361560) TGCCTCTAAA GACTCAGCTC CGTAGTAGAG TAGTTTTAGT TACGATTAAA 10011
DWV-B USA 2018 EVR23 (OR361559) TGCCTCTAAA GACTCAGCTC CGTAGTAGAG TAGTTTTAGT TACGATTAAA 10011
DWV-B Netherlands 2001 VDV-1 (NC_006494) TGCCTCTAAA GACTCAGCTC CGTAGTAGAG TAGTTTTAGT TACGATTAAA 10010
DWV-B France 2019 VV144I (MN565037) TGCCTCTAAA GACTCAGCTC CGTAGTAGAG TAGTTTTAGT TACGATTAAA 10011
DWV-B Slovenia 2019 341-1 (ON648741) TGCCTCTAAA GACTCAGCTC CGTAGTAGAG TAGTTTTAGT TACGATTAAA 10011
DWV-B Austria 2018 B11B (OL803829) TGCCTCTAAA GACTCAGCTC CGTAGTAGAG TAGTTTTAGT TACGATTAAA 10011
DWV-A Austria 2014 1414 (KU847397) TGCCTCTAAA GACTCAGCTC CATAGTAGAG TAGGTTTAAT TACGATTAAA 10027
DWV-A USA 2003 PA (AY292384) TGCCTCTAAA GACTCAGCTC CATAGTAGAG TAGTTTTAAT TACGATTAAA 10024
DWV-A Japan 2001 Kakugo (AB070959) TGCCTCTAAA GACTCAGCTC CATAGTAGAG TAGTTTTAAT TACGATTAAA 10009
DWV-C UK 2015 Devon (ERS657949) TGCCTCTAAA GACTCGACTC CATAGTAGAG TAGGTTTAGT TGCGATCAAA 9995
DWV-D Egypt 1977 EBV (MT504363) TGCCTCTAAA GACTCAACTC CATAGTAGAG TAGGTTTAGT TACGATTAAA 10033
Darwin bee virus 3 Australia 2014 NT-12 (MG995697) CAGCGCTACC GTGTCTAGTC GATAGGAATG -GGTACTAGG TGTGTTGAGA 9686
Consensus TGCCTCTAAA GACTCAGCTC CGTAGTAGAG TAGTTTTAGT TACGATTAAA
100%
Conservation
0%
10.220 10.240
DWV-B Austria 2022 SB22 (PP418870) GTGGTACTCT AGGTTAGGTG TTACTCGCGT ATTGTCGCAT AACGG---CA 10058
DWV-B USA 2018 EVR24 (OR361560) GTGGTACTCT AGGTTAGGTG TTACTCGCGT ATTGTCGCAT AACGG---CA 10058
DWV-B USA 2018 EVR23 (OR361559) GTGGTACTCT AGGTTAGGTG TTACTCGCGT ATTGTCGCAT AACGG---CA 10058
DWV-B Netherlands 2001 VDV-1 (NC_006494) GTGGTACTCT AGGTTAGGTG TTACTCGCGT ATTGTCGCAT AACGG---CA 10057
DWV-B France 2019 VV144I (MN565037) GTGGTACTCT AGGTTAGGTG TTACTCGCGT ATTGTCGCAT AACGG---CA 10058
DWV-B Slovenia 2019 341-1 (ON648741) GTGGTACTCT AGGTTAGGTG TTACTCGCGT ATTGTCGCAT AACAG---CA 10058
DWV-B Austria 2018 B11B (OL803829) GTGGTACTCT AGGTTAGGTG TTACTCGCGT ATTGTCGCAT AACGG---CA 10058
DWV-A Austria 2014 1414 (KU847397) GTGGTACTCT AGGTTAGGTG TTACTCGCGT ATTATCAACT AGTGG---TA 10074
DWV-A USA 2003 PA (AY292384) GTGGTACTCT AGGTTAGGTG TTACTCGCGT ATTATCAACT AGTGG---TA 10071
DWV-A Japan 2001 Kakugo (AB070959) GTGGTACTCT AGGTTAGGTG TTACTCGCGT ATTATCAACT AGTGG---TA 10056
DWV-C UK 2015 Devon (ERS657949) ATGGTACTCT AAATTAGGTG TTACTCGCGT ATTGTC--GT GATAG---CA 10040
DWV-D Egypt 1977 EBV (MT504363) ATGGTACTCT AAATTAGGTG TTACTCACGT ATTGTCTGGT AATTG---CA 10080
Darwin bee virus 3 Australia 2014 NT-12 (MG995697) ---------T CCCCTGGTCG TTGGGAGAGT CCTACTAGAT AAGGGGCCCA 9727
Consensus GTGGTACTCT AGGTTAGGTG TTACTCGCGT ATTGTCGCAT AACGG---CA
100%
Conservation
0%

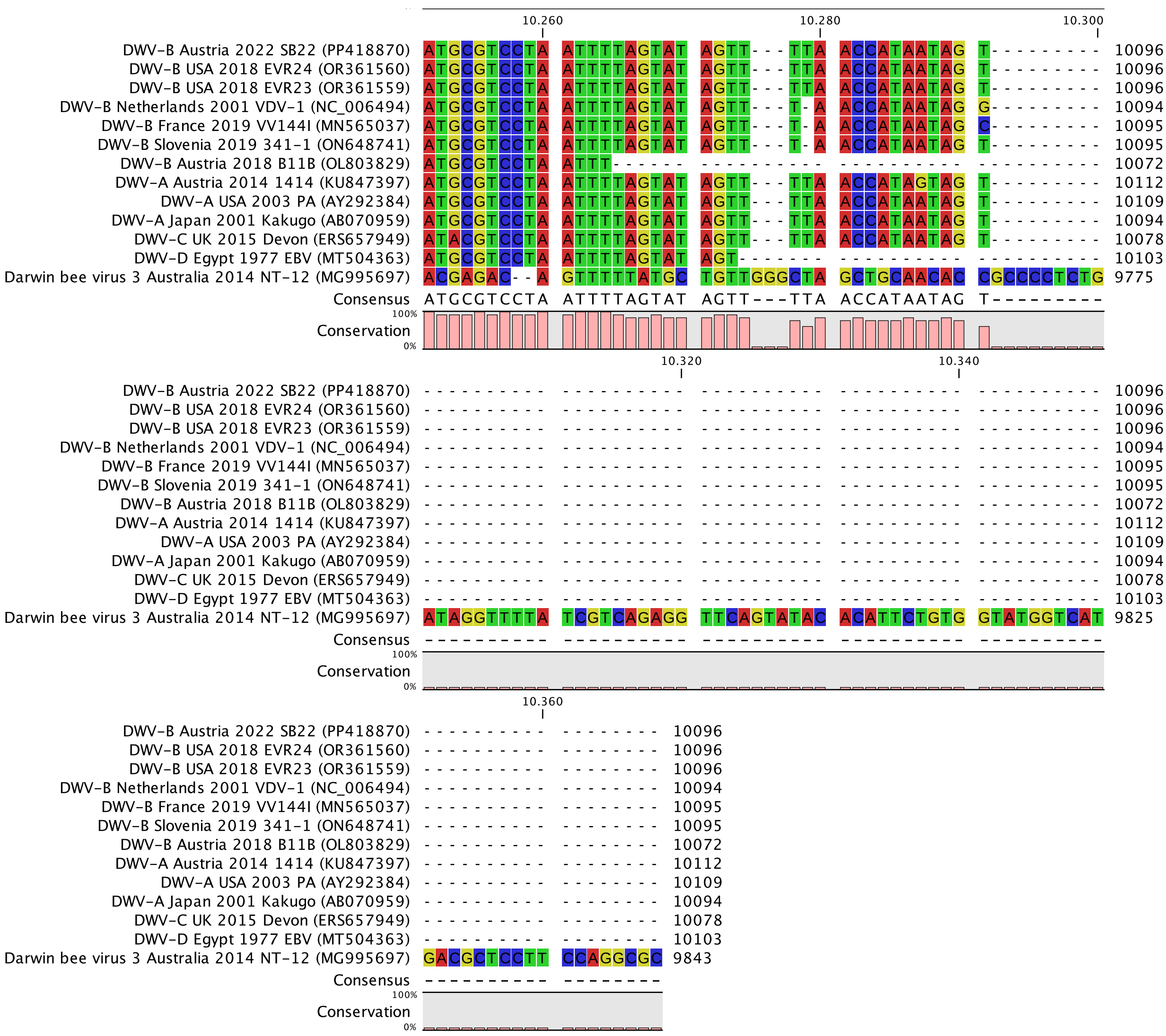
10.260
10.280
10.300
DWV-B Austria 2022 SB22 (PP418870) ATGCGTCCTA ATTTTAGTAT AGTT---TTA ACCATAATAG T--------- 10096
DWV-B USA 2018 EVR24 (OR361560) ATGCGTCCTA ATTTTAGTAT AGTT---TTA ACCATAATAG T--------- 10096
DWV-B USA 2018 EVR23 (OR361559) ATGCGTCCTA ATTTTAGTAT AGTT---TTA ACCATAATAG T--------- 10096
DWV-B Netherlands 2001 VDV-1 (NC_006494) ATGCGTCCTA ATTTTAGTAT AGTT---T-A ACCATAATAG G--------- 10094
DWV-B France 2019 VV144I (MN565037) ATGCGTCCTA ATTTTAGTAT AGTT---T-A ACCATAATAG C--------- 10095
DWV-B Slovenia 2019 341-1 (ON648741) ATGCGTCCTA ATTTTAGTAT AGTT---T-A ACCATAATAG T--------- 10095
DWV-B Austria 2018 B11B (OL803829) ATGCGTCCTA ATTT------ ---------- ---------- ---------- 10072
DWV-A Austria 2014 1414 (KU847397) ATGCGTCCTA ATTTTAGTAT AGTT---TTA ACCATAGTAG T--------- 10112
DWV-A USA 2003 PA (AY292384) ATGCGTCCTA ATTTTAGTAT AGTT---TTA ACCATAATAG T--------- 10109
DWV-A Japan 2001 Kakugo (AB070959) ATGCGTCCTA ATTTTAGTAT AGTT---TTA ACCATAATAG T--------- 10094
DWV-C UK 2015 Devon (ERS657949) ATACGTCCTA ATTTTAGTAT AGTT---TTA ACCATAATAG T--------- 10078
DWV-D Egypt 1977 EBV (MT504363) ATGCGTCCTA ATTTTAGTAT AGT------- ---------- ---------- 10103
Darwin bee virus 3 Australia 2014 NT-12 (MG995697) ACGAGAC--A GTTTTTATGC TGTTGGGCTA GCTGCAACAC CGCCCCTCTG 9775
Consensus ATGCGTCCTA ATTTTAGTAT AGTT---TTA ACCATAATAG T---------
100%
Conservation
0%
10.320
10.340
DWV-B Austria 2022 SB22 (PP418870) ---------- ---------- ---------- ---------- ---------- 10096
DWV-B USA 2018 EVR24 (OR361560) ---------- ---------- ---------- ---------- ---------- 10096
DWV-B USA 2018 EVR23 (OR361559) ---------- ---------- ---------- ---------- ---------- 10096
DWV-B Netherlands 2001 VDV-1 (NC_006494) ---------- ---------- ---------- ---------- ---------- 10094
DWV-B France 2019 VV144I (MN565037) ---------- ---------- ---------- ---------- ---------- 10095
DWV-B Slovenia 2019 341-1 (ON648741) ---------- ---------- ---------- ---------- ---------- 10095
DWV-B Austria 2018 B11B (OL803829) ---------- ---------- ---------- ---------- ---------- 10072
DWV-A Austria 2014 1414 (KU847397) ---------- ---------- ---------- ---------- ---------- 10112
DWV-A USA 2003 PA (AY292384) ---------- ---------- ---------- ---------- ---------- 10109
DWV-A Japan 2001 Kakugo (AB070959) ---------- ---------- ---------- ---------- ---------- 10094
DWV-C UK 2015 Devon (ERS657949) ---------- ---------- ---------- ---------- ---------- 10078
DWV-D Egypt 1977 EBV (MT504363) ---------- ---------- ---------- ---------- ---------- 10103
Darwin bee virus 3 Australia 2014 NT-12 (MG995697) ATAGGTTTTA TCGTCAGAGG TTCAGTATAC ACATTCTGTG GTATGGTCAT 9825
Consensus ---------- ---------- ---------- ---------- ----------
100%
Conservation
0%
10.360
DWV-B Austria 2022 SB22 (PP418870) ---------- -------- 10096
DWV-B USA 2018 EVR24 (OR361560) ---------- -------- 10096
DWV-B USA 2018 EVR23 (OR361559) ---------- -------- 10096
DWV-B Netherlands 2001 VDV-1 (NC_006494) ---------- -------- 10094
DWV-B France 2019 VV144I (MN565037) ---------- -------- 10095
DWV-B Slovenia 2019 341-1 (ON648741) ---------- -------- 10095
DWV-B Austria 2018 B11B (OL803829) ---------- -------- 10072
DWV-A Austria 2014 1414 (KU847397) ---------- -------- 10112
DWV-A USA 2003 PA (AY292384) ---------- -------- 10109
DWV-A Japan 2001 Kakugo (AB070959) ---------- -------- 10094
DWV-C UK 2015 Devon (ERS657949) ---------- -------- 10078
DWV-D Egypt 1977 EBV (MT504363) ---------- -------- 10103
Darwin bee virus 3 Australia 2014 NT-12 (MG995697) GACGCTCCTT CCAGGCGC 9843
Consensus ---------- --------
100%
Conservation
0%

Figure S3. Polyprotein alignment of DWVs. The amino acid sequences of DWV-B strains Austria-SB22 (PP418870), VDV-1 (NC_006494), ER23 (OR361559), ER24 (OR361560), B11B (OL803829), 341-1 (ON648741), and VV144I (MN565037) were aligned with three DWV-A master variant strains, namely PA (AY292384), 1414 (KU847397), and Kakugo (AB070959). DWV master variants C and D were represented by strains Devon (European Nucleotide Archive ER5657949) and Egypt bee virus (MT504363), respectively. Strain NT-12 (MG995697) of Darwin bee virus 3 was included in the alignments as an outgroup. For the DWV-C sequence (ER5657949), a crucial adjustment had to be made: the insertion of a C at position 5,327 to restore the open reading frame, which had been frame shifted at this point. Multiple sequence alignments were conducted using CLC Genomics Workbench (Version 7.7.1, Qiagen) with default parameters.

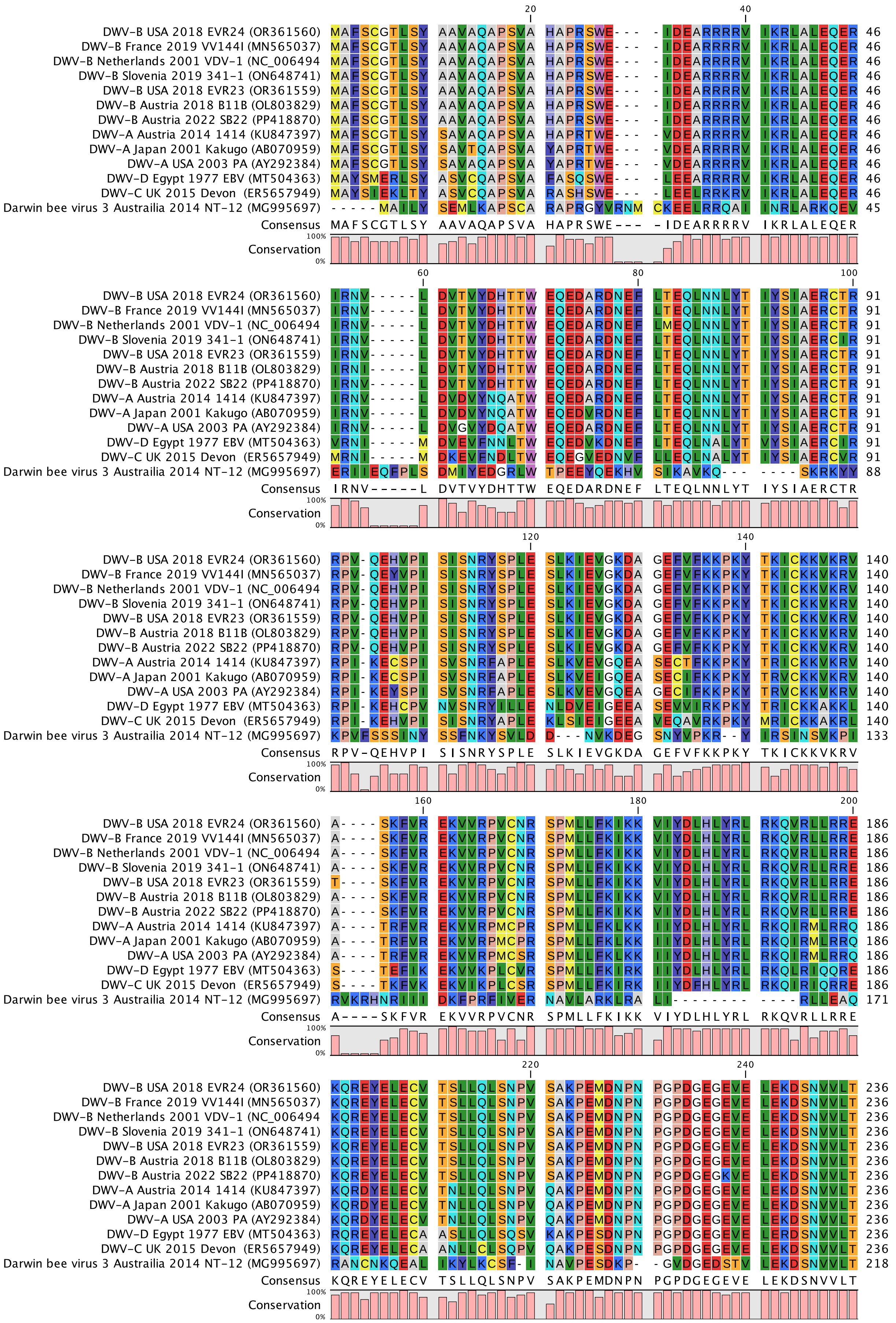
DWV-B USA 2018 EVR24 (OR361560)
DWV-B France 2019 VV144I (MN565037)
DWV-B Netherlands 2001 VDV-1 (NC_006494
DWV-B Slovenia 2019 341-1 (ON648741)
DWV-B USA 2018 EVR23 (OR361559)
DWV-B Austria 2018 B11B (OL803829)
DWV-B Austria 2022 SB22 (PP418870)
DWV-A Austria 2014 1414 (KU847397)
DWV-A Japan 2001 Kakugo (AB070959)
DWV-A USA 2003 PA (AY292384)
DWV-D Egypt 1977 EBV (MT504363)
DWV-C UK 2015 Devon (ER5657949)
Darwin bee virus 3 Austrailia 2014 NT-12 (MG995697)
Consensus MAFSCGTLSY AAVAQAPSVA HAPRSWE--- -IDEARRRRV IKRLALEQER
Consensus IRNV-----L DVTVYDHTTW EQEDARDNEF LTEQLNNLYT IYSIAERCTR
Consensus RPV-QEHVPI SISNRYSPLE SLKIEVGKDA GEFVFKKPKY TKICKKVKRV
Consensus A----SKFVR EKVVRPVCNR SPMLLFKIKK VIYDLHLYRL RKQVRLLRRE
Consensus KQREYELECV TSLLQLSNPV SAKPEMDNPN PGPDGEGEVE LEKDSNVVLT
Conservation
100%
0%

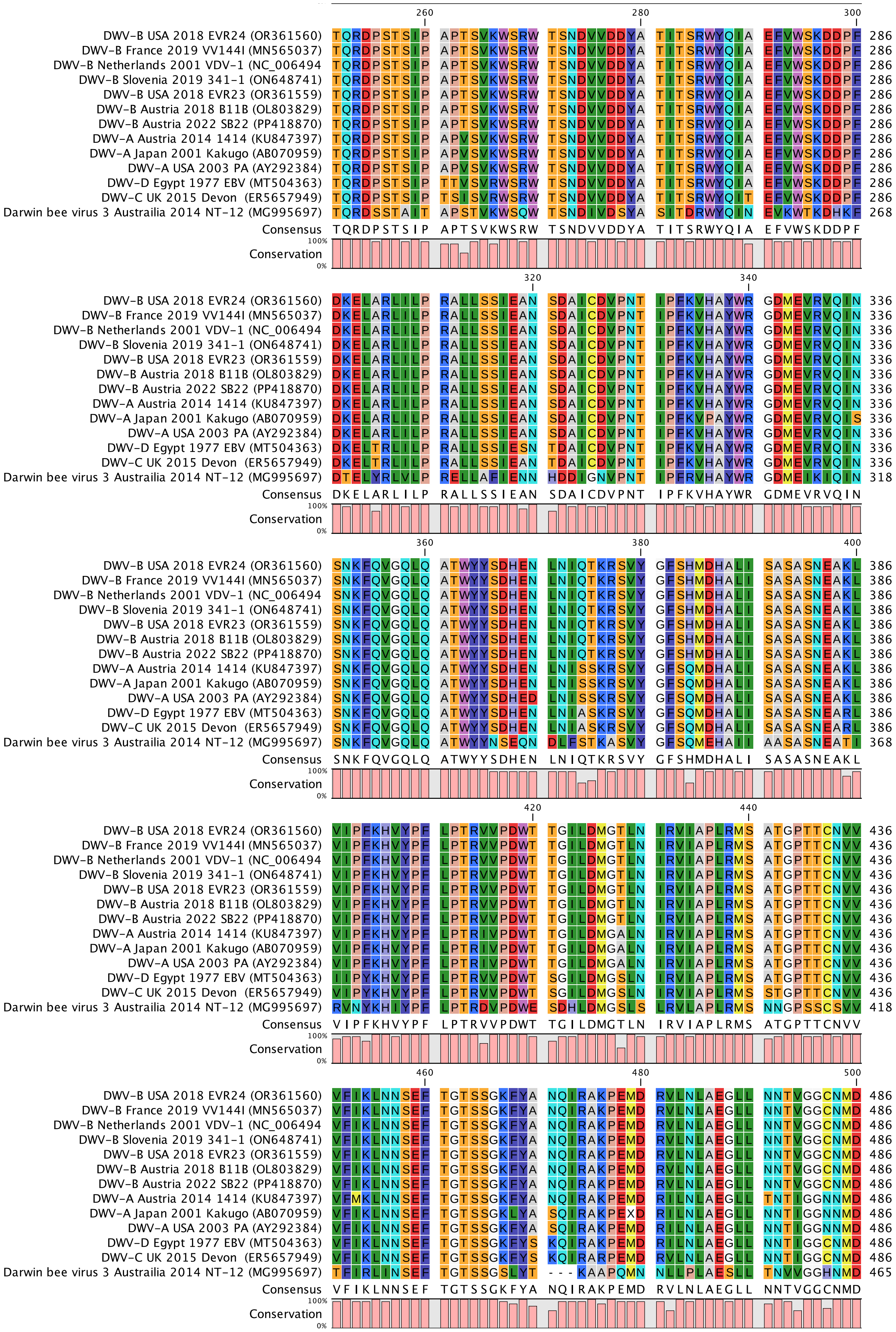

260 280 300
DWV-B USA 2018 EVR24 (OR361560) TQRDPSTSIP APTSVKWSRW TSNDVVDDYA TITSRWYQIA EFVWSKDDPF 286
DWV-B France 2019 VV144I (MN565037) TQRDPSTSIP APTSVKWSRW TSNDVVDDYA TITSRWYQIA EFVWSKDDPF 286
DWV-B Netherlands 2001 VDV-1 (NC_006494 TQRDPSTSIP APTSVKWSRW TSNDVVDDYA TITSRWYQIA EFVWSKDDPF 286
DWV-B Slovenia 2019 341-1 (ON648741) TQRDPSTSIP APTSVKWSRW TSNDVVDDYA TITSRWYQIA EFVWSKDDPF 286
DWV-B USA 2018 EVR23 (OR361559) TQRDPSTSIP APTSVKWSRW TSNDVVDDYA TITSRWYQIA EFVWSKDDPF 286
DWV-B Austria 2018 B11B (OL803829) TQRDPSTSIP APTSVKWSRW TSNDVVDDYA TITSRWYQIA EFVWSKDDPF 286
DWV-B Austria 2022 SB22 (PP418870) TQRDPSTSIP APTSVKWSRW TSNDVVDDYA TITSRWYQIA EFVWSKDDPF 286
DWV-A Austria 2014 1414 (KU847397) TQRDPSTSIP APVSVKWSRW TSNDVVDDYA TITSRWYQIA EFVWSKDDPF 286
DWV-A Japan 2001 Kakugo (AB070959) TQRDPSTSIP APVSVKWSRW TSNDVVDDYA TITSRWYQIA EFVWSKDDPF 286
DWV-A USA 2003 PA (AY292384) TQRDPSTSIP APVSVKWSRW TSNDVVDDYA TITSRWYQIA EFVWSKDDPF 286
DWV-D Egypt 1977 EBV (MT504363) TQRDPSTSIP TTVSVRWSRW TSNDVVDDYA TITSRWYQIA EFVWSKDDPF 286
DWV-C UK 2015 Devon (ER5657949) TQRDPSTSIP TSISVRWSRW TSNDVVDDYA TITSRWYQIT EFVWSKDDPF 286
Darwin bee virus 3 Austrailia 2014 NT-12 (MG995697) TQRDSSTAIT APSTVKWSQW TSNDIVDSYA SITDRWYQIN EVKWTKDHKF 268
Consensus TQRDPSTSIP APTSVKWSRW TSNDVVDDYA TITSRWYQIA EFVWSKDDPF
100%
Conservation
0%
320 340
DWV-B USA 2018 EVR24 (OR361560) DKELARLILP RALLSSIEAN SDAICDVPNT IPFKVHAYWR GDMEVRVQIN 336
DWV-B France 2019 VV144I (MN565037) DKELARLILP RALLSSIEAN SDAICDVPNT IPFKVHAYWR GDMEVRVQIN 336
DWV-B Netherlands 2001 VDV-1 (NC_006494 DKELARLILP RALLSSIEAN SDAICDVPNT IPFKVHAYWR GDMEVRVQIN 336
DWV-B Slovenia 2019 341-1 (ON648741) DKELARLILP RALLSSIEAN SDAICDVPNT IPFKVHAYWR GDMEVRVQIN 336
DWV-B USA 2018 EVR23 (OR361559) DKELARLILP RALLSSIEAN SDAICDVPNT IPFKVHAYWR GDMEVRVQIN 336
DWV-B Austria 2018 B11B (OL803829) DKELARLILP RALLSSIEAN SDAICDVPNT IPFKVHAYWR GDMEVRVQIN 336
DWV-B Austria 2022 SB22 (PP418870) DKELARLILP RALLSSIEAN SDAICDVPNT IPFKVHAYWR GDMEVRVQIN 336
DWV-A Austria 2014 1414 (KU847397) DKELARLILP RALLSSIEAN SDAICDVPNT IPFKVHAYWR GDMEVRVQIN 336
DWV-A Japan 2001 Kakugo (AB070959) DKELARLILP RALLSSIEAN SDAICDVPNT IPFKVPAYWR GDMEVRVQIS 336
DWV-A USA 2003 PA (AY292384) DKELARLILP RALLSSIEAN SDAICDVPNT IPFKVHAYWR GDMEVRVQIN 336
DWV-D Egypt 1977 EBV (MT504363) DKELTRLILP RALLSSIESN TDAICDVPNT IPFKVHAYWR GDMEVRVQIN 336
DWV-C UK 2015 Devon (ER5657949) DKELTRLILP RALLSSIEAN TDAICDVPNT IPFKVHAYWR GDMEVRVQIN 336
Darwin bee virus 3 Austrailia 2014 NT-12 (MG995697) DTELYRLVLP RELLAFIENN HDDIGNVPNT IPFRVHAYWR GDMEIKIQIN 318
Consensus DKELARLILP RALLSSIEAN SDAICDVPNT IPFKVHAYWR GDMEVRVQIN
100%
Conservation
0%
360 380 400
DWV-B USA 2018 EVR24 (OR361560) SNKFQVGQLQ ATWYYSDHEN LNIQTKRSVY GFSHMDHALI SASASNEAKL 386
DWV-B France 2019 VV144I (MN565037) SNKFQVGQLQ ATWYYSDHEN LNIQTKRSVY GFSHMDHALI SASASNEAKL 386
DWV-B Netherlands 2001 VDV-1 (NC_006494 SNKFQVGQLQ ATWYYSDHEN LNIQTKRSVY GFSHMDHALI SASASNEAKL 386
DWV-B Slovenia 2019 341-1 (ON648741) SNKFQVGQLQ ATWYYSDHEN LNIQTKRSVY GFSHMDHALI SASASNEAKL 386
DWV-B USA 2018 EVR23 (OR361559) SNKFQVGQLQ ATWYYSDHEN LNIQTKRSVY GFSHMDHALI SASASNEAKL 386
DWV-B Austria 2018 B11B (OL803829) SNKFQVGQLQ ATWYYSDHEN LNIQTKRSVY GFSHMDHALI SASASNEAKL 386
DWV-B Austria 2022 SB22 (PP418870) SNKFQVGQLQ ATWYYSDHEN LNIQTKRSVY GFSHMDHALI SASASNEAKL 386
DWV-A Austria 2014 1414 (KU847397) SNKFQVGQLQ ATWYYSDHEN LNISSKRSVY GFSQMDHALI SASASNEAKL 386
DWV-A Japan 2001 Kakugo (AB070959) SNKFQVGQLQ ATWYYSDHEN LNISSKRSVY GFSQMDHALI SASASNEAKL 386
DWV-A USA 2003 PA (AY292384) SNKFQVGQLQ ATWYYSDHED LNISSKRSVY GFSQMDHALI SASASNEAKL 386
DWV-D Egypt 1977 EBV (MT504363) SNKFQVGQLQ ATWYYSDHEN LNIASKRSVY GFSQMDHALI SASASNEARL 386
DWV-C UK 2015 Devon (ER5657949) SNKFQVGQLQ ATWYYSDHEN LNIASKRSVY GFSQMDHALI SASASNEARL 386
Darwin bee virus 3 Austrailia 2014 NT-12 (MG995697) SNKFQVGQLQ ATWYYNSEQN DLFSTKASVY GFSQMEHAII AASASNEATI 368
Consensus SNKFQVGQLQ ATWYYSDHEN LNIQTKRSVY GFSHMDHALI SASASNEAKL
100%
Conservation
0%
420 440
DWV-B USA 2018 EVR24 (OR361560) VIPFKHVYPF LPTRVVPDWT TGILDMGTLN IRVIAPLRMS ATGPTTCNVV 436
DWV-B France 2019 VV144I (MN565037) VIPFKHVYPF LPTRVVPDWT TGILDMGTLN IRVIAPLRMS ATGPTTCNVV 436
DWV-B Netherlands 2001 VDV-1 (NC_006494 VIPFKHVYPF LPTRVVPDWT TGILDMGTLN IRVIAPLRMS ATGPTTCNVV 436
DWV-B Slovenia 2019 341-1 (ON648741) VIPFKHVYPF LPTRVVPDWT TGILDMGTLN IRVIAPLRMS ATGPTTCNVV 436
DWV-B USA 2018 EVR23 (OR361559) VIPFKHVYPF LPTRVVPDWT TGILDMGTLN IRVIAPLRMS ATGPTTCNVV 436
DWV-B Austria 2018 B11B (OL803829) VIPFKHVYPF LPTRVVPDWT TGILDMGTLN IRVIAPLRMS ATGPTTCNVV 436
DWV-B Austria 2022 SB22 (PP418870) VIPFKHVYPF LPTRVVPDWT TGILDMGTLN IRVIAPLRMS ATGPTTCNVV 436
DWV-A Austria 2014 1414 (KU847397) VIPFKHVYPF LPTRIVPDWT TGILDMGALN IRVIAPLRMS ATGPTTCNVV 436
DWV-A Japan 2001 Kakugo (AB070959) VIPFKHVYPF LPTRIVPDWT TGILDMGALN IRVIAPLRMS ATGPTTCNVV 436
DWV-A USA 2003 PA (AY292384) VIPFKHVYPF LPTRIVPDWT TGILDMGALN IRVIAPLRMS ATGPTTCNVV 436
DWV-D Egypt 1977 EBV (MT504363) IIPYKHVYPF LPTRVVPDWT SGILDMGSLN IRVIAPLRMS ATGPTTCNVV 436
DWV-C UK 2015 Devon (ER5657949) VIPYKHVYPF LPTRVVPDWT SGILDMGSLN IRVIAPLRMS STGPTTCNVV 436
Darwin bee virus 3 Austrailia 2014 NT-12 (MG995697) RVNYKHIYPF LPTRDVPDWE SDHLDMGSLS LRVLAPLRMS NNGPSSCSVV 418
Consensus VIPFKHVYPF LPTRVVPDWT TGILDMGTLN IRVIAPLRMS ATGPTTCNVV
100%
Conservation
0%
460 480 500
DWV-B USA 2018 EVR24 (OR361560) VFIKLNNSEF TGTSSGKFYA NQIRAKPEMD RVLNLAEGLL NNTVGGCNMD 486
DWV-B France 2019 VV144I (MN565037) VFIKLNNSEF TGTSSGKFYA NQIRAKPEMD RVLNLAEGLL NNTVGGCNMD 486
DWV-B Netherlands 2001 VDV-1 (NC_006494 VFIKLNNSEF TGTSSGKFYA NQIRAKPEMD RVLNLAEGLL NNTVGGCNMD 486
DWV-B Slovenia 2019 341-1 (ON648741) VFIKLNNSEF TGTSSGKFYA NQIRAKPEMD RVLNLAEGLL NNTVGGCNMD 486
DWV-B USA 2018 EVR23 (OR361559) VFIKLNNSEF TGTSSGKFYA NQIRAKPEMD RVLNLAEGLL NNTVGGCNMD 486
DWV-B Austria 2018 B11B (OL803829) VFIKLNNSEF TGTSSGKFYA NQIRAKPEMD RVLNLAEGLL NNTVGGCNMD 486
DWV-B Austria 2022 SB22 (PP418870) VFIKLNNSEF TGTSSGKFYA NQIRAKPEMD RVLNLAEGLL NNTVGGCNMD 486
DWV-A Austria 2014 1414 (KU847397) VFMKLNNSEF TGTSSGKFYA NQIRAKPEMD RILNLAEGLL TNTIGGNNMD 486
DWV-A Japan 2001 Kakugo (AB070959) VFIKLNNSEF TGTSSGKLYA SQIRAKPEXD RILNLAEGLL NNTIGGNNMD 486
DWV-A USA 2003 PA (AY292384) VFIKLNNSEF TGTSSGKFYA SQIRAKPEMD RILNLAEGLL NNTIGGNNMD 486
DWV-D Egypt 1977 EBV (MT504363) VFIKLNNSEF TGTSSGKFYS KQIRAKPEMD RILNLAEGLL NNTIGGCNMD 486
DWV-C UK 2015 Devon (ER5657949) VFIKLNNSEF TGTSSGKFYS KQIRARPEMD RVLNLAEGLL NNTIGGCNMD 486
Darwin bee virus 3 Austrailia 2014 NT-12 (MG995697) TFIRLINSEF TGTSSGSLYT ---KAAPQMN NLLPLAESLL TNVVGGHNMD 465
Consensus VFIKLNNSEF TGTSSGKFYA NQIRAKPEMD RVLNLAEGLL NNTVGGCNMD
100%
Conservation
0%

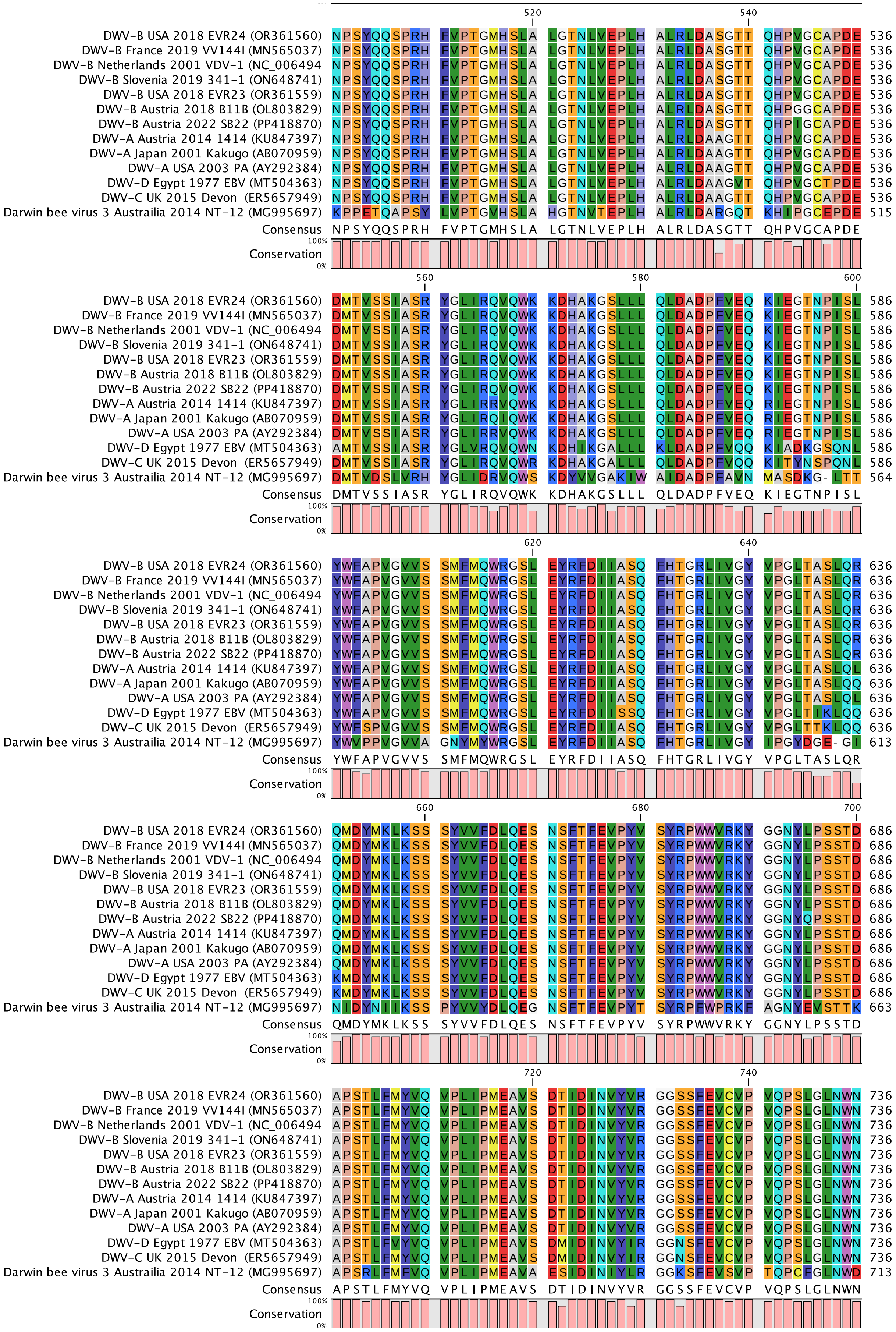
DWV-B USA 2018 EVR24 (OR361560)
DWV-B France 2019 VV144I (MN565037)
DWV-B Netherlands 2001 VDV-1 (NC_006494
DWV-B Slovenia 2019 341-1 (ON648741)
DWV-B USA 2018 EVR23 (OR361559)
DWV-B Austria 2018 B11B (OL803829)
DWV-B Austria 2022 SB22 (PP418870)
DWV-A Austria 2014 1414 (KU847397)
DWV-A Japan 2001 Kakugo (AB070959)
DWV-A USA 2003 PA (AY292384)
DWV-D Egypt 1977 EBV (MT504363)
DWV-C UK 2015 Devon (ER5657949)
Darwin bee virus 3 Austrailia 2014 NT-12 (MG995697)
Consensus NPSYQQSPRH FVPTGMHSLA LGTNLVEPLH ALRLDASGTT QHPVGCAPDE
Consensus DMTVSSIASR YGLIRQVQWK KDHAKGSLLL QLDADPFVEQ KIEGTNPISL
Consensus YWFAPVGVVS SMFMQWRGSL EYRFDIIASQ FHTGRLIVGY VPGLTASLQR
Consensus QMDYMKLKSS SYVVFDLQES NSFTFEVPYV SYRPWWVRKY GGNYLPSSTD
Consensus APSTLFMYVQ VPLIPMEAVS DTIDINVYVR GGSSFEVCVP VQPSLGLNWN
Conservation

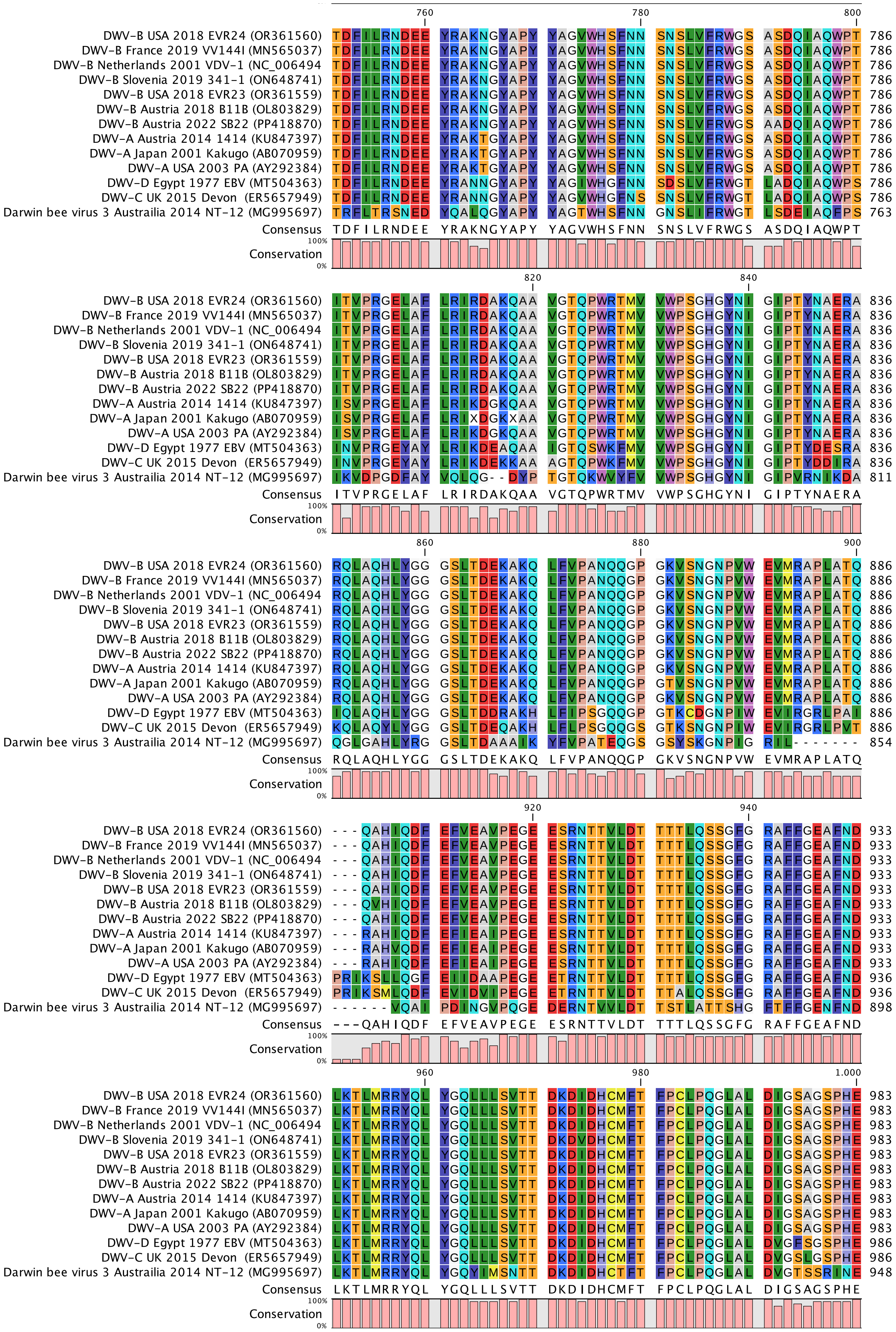

760
780
800
DWV-B USA 2018 EVR24 (OR361560)
DWV-B France 2019 VV1441 (MN565037)
DWV-B Netherlands 2001 VDV-1 (NC_006494
DWV-B Slovenia 2019 341-1 (ON648741)
DWV-B USA 2018 EVR23 (OR361559)
DWV-B Austria 2018 B11B (OL803829)
DWV-B Austria 2022 SB22 (PP418870)
DWV-A Austria 2014 1414 (KU847397)
DWV-A Japan 2001 Kakugo (AB070959)
DWV-A USA 2003 PA (AY292384)
DWV-D Egypt 1977 EBV (MT504363)
DWV-C UK 2015 Devon (ER5657949)
Darwin bee virus 3 Austrailia 2014 NT-12 (MG995697)
Consensus TDFILRNDEE YRAKNGYAPY YAGVWHSFNN SNSLVFRWGS ASDQIAQWPT
Conservation
820
840
Consensus ITVPRGELAF LRIRDAKQAA VGTQPWRTMV VWPSGHGYNI GIPTYNAERA
860
880
900
Consensus RQLAQHLYGG GSLTDEKAKQ LFVPANQQGP GKVSNGNPVW EVMRAPLATQ
920
940
Consensus ---QAHIQDF EFVEAVPEGE ESRNTTVLDT TTTLQSSGFG RAFFGEAFND
960
980
1.000
Consensus LKTLMRRYQL YGQLLLSVTT DKDIDHCMFT FPCLPQGLAL DIGSAGSPHE

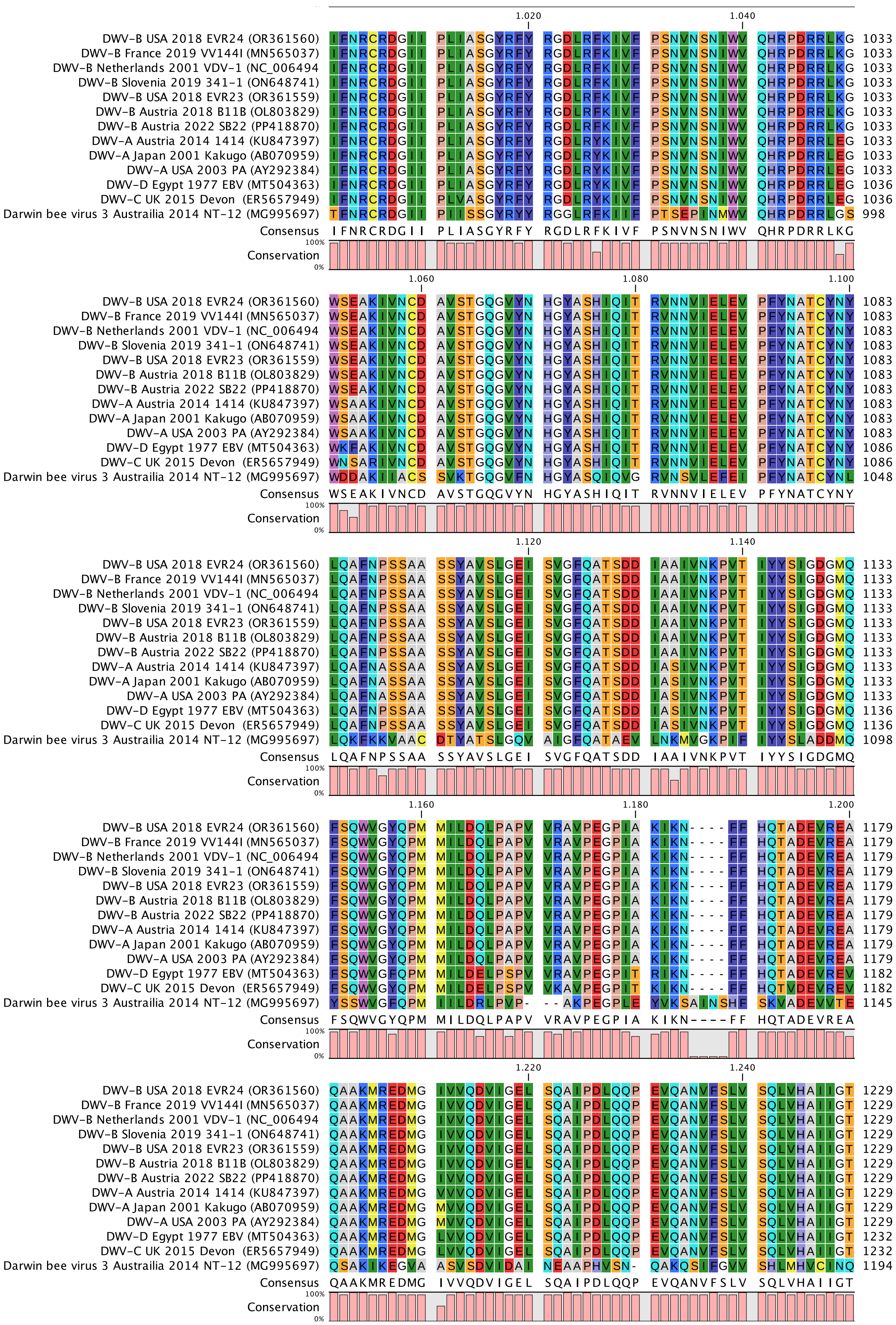
1.020 1.040
DWV-B USA 2018 EVR24 (OR361560) IFNRCRDGII PLIASGYRFY RGDLRFKIVF PSNVNSNIWV QHRPDRRLKG 1033
DWV-B France 2019 VV144I (MN565037) IFNRCRDGII PLIASGYRFY RGDLRFKIVF PSNVNSNIWV QHRPDRRLKG 1033
DWV-B Netherlands 2001 VDV-1 (NC_006494 IFNRCRDGII PLIASGYRFY RGDLRFKIVF PSNVNSNIWV QHRPDRRLKG 1033
DWV-B Slovenia 2019 341-1 (ON648741) IFNRCRDGII PLIASGYRFY RGDLRFKIVF PSNVNSNIWV QHRPDRRLKG 1033
DWV-B USA 2018 EVR23 (OR361559) IFNRCRDGII PLIASGYRFY RGDLRFKIVF PSNVNSNIWV QHRPDRRLKG 1033
DWV-B Austria 2018 B11B (OL803829) IFNRCRDGII PLIASGYRFY RGDLRFKIVF PSNVNSNIWV QHRPDRRLKG 1033
DWV-B Austria 2022 SB22 (PP418870) IFNRCRDGII PLIASGYRFY RGDLRFKIVF PSNVNSNIWV QHRPDRRLKG 1033
DWV-A Austria 2014 1414 (KU847397) IFNRCRDGII PLIASGYRFY RGDLRYKIVF PSNVNSNIWV QHRPDRRLEG 1033
DWV-A Japan 2001 Kakugo (AB070959) IFNRCRDGII PLIASGYRFY RGDLRYKIVF PSNVNSNIWV QHRPDRRLEG 1033
DWV-A USA 2003 PA (AY292384) IFNRCRDGII PLIASGYRFY RGDLRYKIVF PSNVNSNIWV QHRPDRRLEG 1033
DWV-D Egypt 1977 EBV (MT504363) IFNRCRDGII PLIASGYRFY RGDLRYKIVF PSNVNSNIWV QHRPDRRLEG 1036
DWV-C UK 2015 Devon (ER5657949) IFNRCRDGII PLVASGYRFY RGDLRYKIVF PSNVNSNIWV QHRPDRRLEG 1036
Darwin bee virus 3 Austrailia 2014 NT-12 (MG995697) TFNRCRDGII PIISSGYRYY RGGLRFKIIF PTSEPINMWV QHRPDRRLGS 998
Consensus IFNRCRDGII PLIASGYRFY RGDLRFKIVF PSNVNSNIWV QHRPDRRLKG
Conservation 100% 0%
1.060 1.080 1.100
DWV-B USA 2018 EVR24 (OR361560) WSEAKIVNCD AVSTGQGVYN HGYASHIQIT RVNNVIELEV PFYNATCYNY 1083
DWV-B France 2019 VV144I (MN565037) WSEAKIVNCD AVSTGQGVYN HGYASHIQIT RVNNVIELEV PFYNATCYNY 1083
DWV-B Netherlands 2001 VDV-1 (NC_006494 WSEAKIVNCD AVSTGQGVYN HGYASHIQIT RVNNVIELEV PFYNATCYNY 1083
DWV-B Slovenia 2019 341-1 (ON648741) WSEAKIVNCD AVSTGQGVYN HGYASHIQIT RVNNVIELEV PFYNATCYNY 1083
DWV-B USA 2018 EVR23 (OR361559) WSEAKIVNCD AVSTGQGVYN HGYASHIQIT RVNNVIELEV PFYNATCYNY 1083
DWV-B Austria 2018 B11B (OL803829) WSEAKIVNCD AVSTGQGVYN HGYASHIQIT RVNNVIELEV PFYNATCYNY 1083
DWV-B Austria 2022 SB22 (PP418870) WSEAKIVNCD AVSTGQGVYN HGYASHIQIT RVNNVIELEV PFYNATCYNY 1083
DWV-A Austria 2014 1414 (KU847397) WSAAKIVNCD AVSTGQGVYN HGYASHIQIT RVNNVIELEV PFYNATCYNY 1083
DWV-A Japan 2001 Kakugo (AB070959) WSAAKIVNCD AVSTGQGVYN HGYASHIQIT RVNNVIELEV PFYNATCYNY 1083
DWV-A USA 2003 PA (AY292384) WSAAKIVNCD AVSTGQGVYN HGYASHIQIT RVNNVIELEV PFYNATCYNY 1083
DWV-D Egypt 1977 EBV (MT504363) WKFAKIVNCD AVSTGQGVYN HGYASHIQIT RVNNVIELEV PFYNATCYNY 1086
DWV-C UK 2015 Devon (ER5657949) WNSARIVNCD AVSTGQGVYN HGYASHIQIT RVNNVIELEV PFYNATCYNY 1086
Darwin bee virus 3 Austrailia 2014 NT-12 (MG995697) WDDAKIIACS SVKTGQGVFN HGYASQIQVG RVNSVLEFEI PFYNATCYNL 1048
Consensus WSEAKIVNCD AVSTGQGVYN HGYASHIQIT RVNNVIELEV PFYNATCYNY
Conservation 100% 0%
1.120 1.140
DWV-B USA 2018 EVR24 (OR361560) LQAFNPSSAA SSYAVSLGEI SVGFQATSDD IAAIVNKPVT IYYSIGDGMQ 1133
DWV-B France 2019 VV144I (MN565037) LQAFNPSSAA SSYAVSLGEI SVGFQATSDD IAAIVNKPVT IYYSIGDGMQ 1133
DWV-B Netherlands 2001 VDV-1 (NC_006494 LQAFNPSSAA SSYAVSLGEI SVGFQATSDD IAAIVNKPVT IYYSIGDGMQ 1133
DWV-B Slovenia 2019 341-1 (ON648741) LQAFNPSSAA SSYAVSLGEI SVGFQATSDD IAAIVNKPVT IYYSIGDGMQ 1133
DWV-B USA 2018 EVR23 (OR361559) LQAFNPSSAA SSYAVSLGEI SVGFQATSDD IAAIVNKPVT IYYSIGDGMQ 1133
DWV-B Austria 2018 B11B (OL803829) LQAFNPSSAA SSYAVSLGEI SVGFQATSDD IAAIVNKPVT IYYSIGDGMQ 1133
DWV-B Austria 2022 SB22 (PP418870) LQAFNPSSAA SSYAVSLGEI SVGFQATSDD IAAIVNKPVT IYYSIGDGMQ 1133
DWV-A Austria 2014 1414 (KU847397) LQAFNASSAA SSYAVSLGEI SVGFQATSDD IASIVNKPVT IYYSIGDGMQ 1133
DWV-A Japan 2001 Kakugo (AB070959) LQAFNASSAA SSYAVSLGEI SVGFQATSDD IASIVNKPVT IYYSIGDGMQ 1133
DWV-A USA 2003 PA (AY292384) LQAFNASSAA SSYAVSLGEI SVGFQATSDD IASIVNKPVT IYYSIGDGMQ 1133
DWV-D Egypt 1977 EBV (MT504363) LQAFNPSSAA SSYAVSLGEI SVGFQATSDD IASIVNKPVT IYYSIGDGMQ 1136
DWV-C UK 2015 Devon (ER5657949) LQAFNPSSAA SSYAVSLGEI SVGFQATSDD IASIVNKPVT IYYSIGDGMQ 1136
Darwin bee virus 3 Austrailia 2014 NT-12 (MG995697) LQKFKKVAAC DTYATSLGQV AIGFQATAEV LNKMVGKPIF IYYSLADDMQ 1098
Consensus LQAFNPSSAA SSYAVSLGEI SVGFQATSDD IAAIVNKPVT IYYSIGDGMQ
Conservation 100% 0%
1.160 1.180 1.200
DWV-B USA 2018 EVR24 (OR361560) FSQWVGYQPM MILDQLPAPV VRAVPEGPIA KIKN----FF HQTADEVREA 1179
DWV-B France 2019 VV144I (MN565037) FSQWVGYQPM MILDQLPAPV VRAVPEGPIA KIKN----FF HQTADEVREA 1179
DWV-B Netherlands 2001 VDV-1 (NC_006494 FSQWVGYQPM MILDQLPAPV VRAVPEGPIA KIKN----FF HQTADEVREA 1179
DWV-B Slovenia 2019 341-1 (ON648741) FSQWVGYQPM MILDQLPAPV VRAVPEGPIA KIKN----FF HQTADEVREA 1179
DWV-B USA 2018 EVR23 (OR361559) FSQWVGYQPM MILDQLPAPV VRAVPEGPIA KIKN----FF HQTADEVREA 1179
DWV-B Austria 2018 B11B (OL803829) FSQWVGYQPM MILDQLPAPV VRAVPEGPIA KIKN----FF HQTADEVREA 1179
DWV-B Austria 2022 SB22 (PP418870) FSQWVGYQPM MILDQLPAPV VRAVPEGPIA KIKN----FF HQTADEVREA 1179
DWV-A Austria 2014 1414 (KU847397) FSQWVGYQPM MILDQLPAPV VRAVPEGPIA KIKN----FF HQTADEVREA 1179
DWV-A Japan 2001 Kakugo (AB070959) FSQWVGYQPM MILDQLPAPV VRAVPEGPIA KIKN----FF HQTADEVREA 1179
DWV-A USA 2003 PA (AY292384) FSQWVGYQPM MILDQLPAPV VRAVPEGPIA KIKN----FF HQTADEVREA 1179
DWV-D Egypt 1977 EBV (MT504363) FSQWVGFQPM MILDELPSPV VRAVPEGPIT RIKN----FF HQTADEVREV 1182
DWV-C UK 2015 Devon (ER5657949) FSQWVGYQPM MILDELPSPV VKAVPEGPIT KIKN----FF HQTVDEVREV 1182
Darwin bee virus 3 Austrailia 2014 NT-12 (MG995697) YSSWVGFQPM IILDRLPVP- --AKPEGPLE YVKSAINSHF SKVADEVVTE 1145
Consensus FSQWVGYQPM MILDQLPAPV VRAVPEGPIA KIKN----FF HQTADEVREA
Conservation 100% 0%
1.220 1.240
DWV-B USA 2018 EVR24 (OR361560) QAAKMREDMG IVVQDVIGEL SQAIPDLQQP EVQANVFSLV SQLVHAIIGT 1229
DWV-B France 2019 VV144I (MN565037) QAAKMREDMG IVVQDVIGEL SQAIPDLQQP EVQANVFSLV SQLVHAIIGT 1229
DWV-B Netherlands 2001 VDV-1 (NC_006494 QAAKMREDMG IVVQDVIGEL SQAIPDLQQP EVQANVFSLV SQLVHAIIGT 1229
DWV-B Slovenia 2019 341-1 (ON648741) QAAKMREDMG IVVQDVIGEL SQAIPDLQQP EVQANVFSLV SQLVHAIIGT 1229
DWV-B USA 2018 EVR23 (OR361559) QAAKMREDMG IVVQDVIGEL SQAIPDLQQP EVQANVFSLV SQLVHAIIGT 1229
DWV-B Austria 2018 B11B (OL803829) QAAKMREDMG IVVQDVIGEL SQAIPDLQQP EVQANVFSLV SQLVHAIIGT 1229
DWV-B Austria 2022 SB22 (PP418870) QAAKMREDMG IVVQDVIGEL SQAIPDLQQP EVQANVFSLV SQLVHAIIGT 1229
DWV-A Austria 2014 1414 (KU847397) QAAKMREDMG VVVQDVIGEL SQAIPDLQQP EVQANVFSLV SQLVHAIIGT 1229
DWV-A Japan 2001 Kakugo (AB070959) QAAKMREDMG MVVQDVIGEL SQAIPDLQQP EVQANVFSLV SQLVHAIIGT 1229
DWV-A USA 2003 PA (AY292384) QAAKMREDMG MVVQDVIGEL SQAIPDLQQP EVQANVFSLV SQLVHAIIGT 1229
DWV-D Egypt 1977 EBV (MT504363) QAAKMREDMG LVVQDVIGEL SQAIPDLQQP EVQANVFSLV SQLVHAIIGT 1232
DWV-C UK 2015 Devon (ER5657949) QAAKMREDMG LVVQDVIGEL SQAIPDLQQP EVQANVFSLV SQLVHAIIGT 1232
Darwin bee virus 3 Austrailia 2014 NT-12 (MG995697) QSAKIKEGVA ASVSDVIDAI NEAAPHVSN- QAKQSIFGVV SHLMHVCINQ 1194
Consensus QAAKMREDMG IVVQDVIGEL SQAIPDLQQP EVQANVFSLV SQLVHAIIGT
Conservation 100% 0%

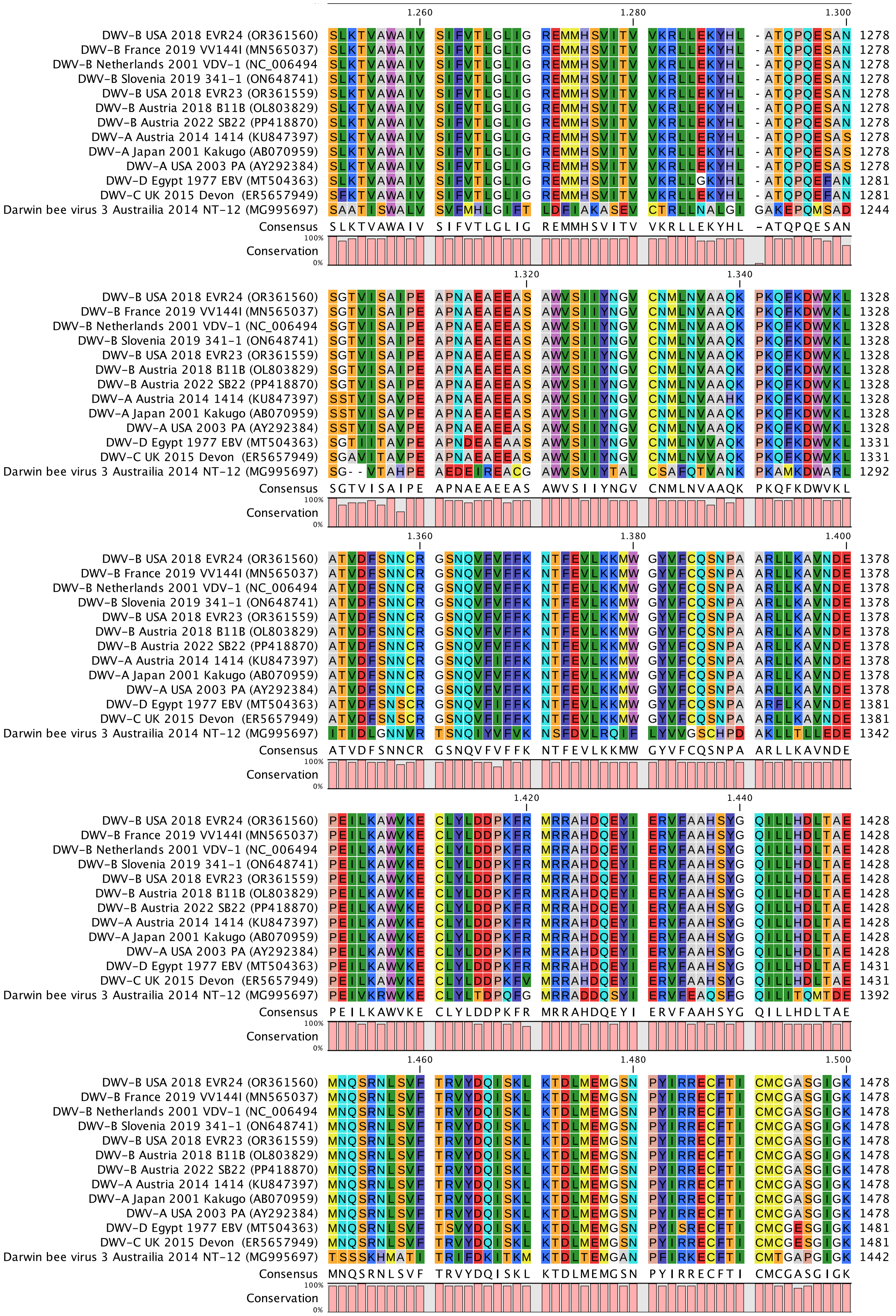
DWV-B USA 2018 EVR24 (OR361560)
DWV-B France 2019 VV144I (MN565037)
DWV-B Netherlands 2001 VDV-1 (NC_006494
DWV-B Slovenia 2019 341-1 (ON648741)
DWV-B USA 2018 EVR23 (OR361559)
DWV-B Austria 2018 B11B (OL803829)
DWV-B Austria 2022 SB22 (PP418870)
DWV-A Austria 2014 1414 (KU847397)
DWV-A Japan 2001 Kakugo (AB070959)
DWV-A USA 2003 PA (AY292384)
DWV-D Egypt 1977 EBV (MT504363)
DWV-C UK 2015 Devon (ER5657949)
Darwin bee virus 3 Austrailia 2014 NT-12 (MG995697)
Consensus
Conservation
100%
0%
1.260 1.280 1.300
Consensus SLKTVAWAIV SIFVTLGLIG REMMHSVITV VKRLLEKYHL -ATQPQESAN
1.320 1.340
Consensus SGTVISAIPE APNAEAEEAS AWVSIIYNGV CNMLNVAAQK PKQFKDWVKL
1.360 1.380 1.400
Consensus ATVDFSNNCR GSNQVFVFFK NTFEVLKKMW GYVFCQSNPA ARLLKAVNDE
1.420 1.440
Consensus PEILKAWVKE CLYLDDPKFR MRRAHDQEYI ERVFAAHSYG QILLHDLTAE
1.460 1.480 1.500
Consensus MNQSRNLSVF TRVYDQISKL KTDLMEMGSN PYIRRECFTI CMCGASGIGK

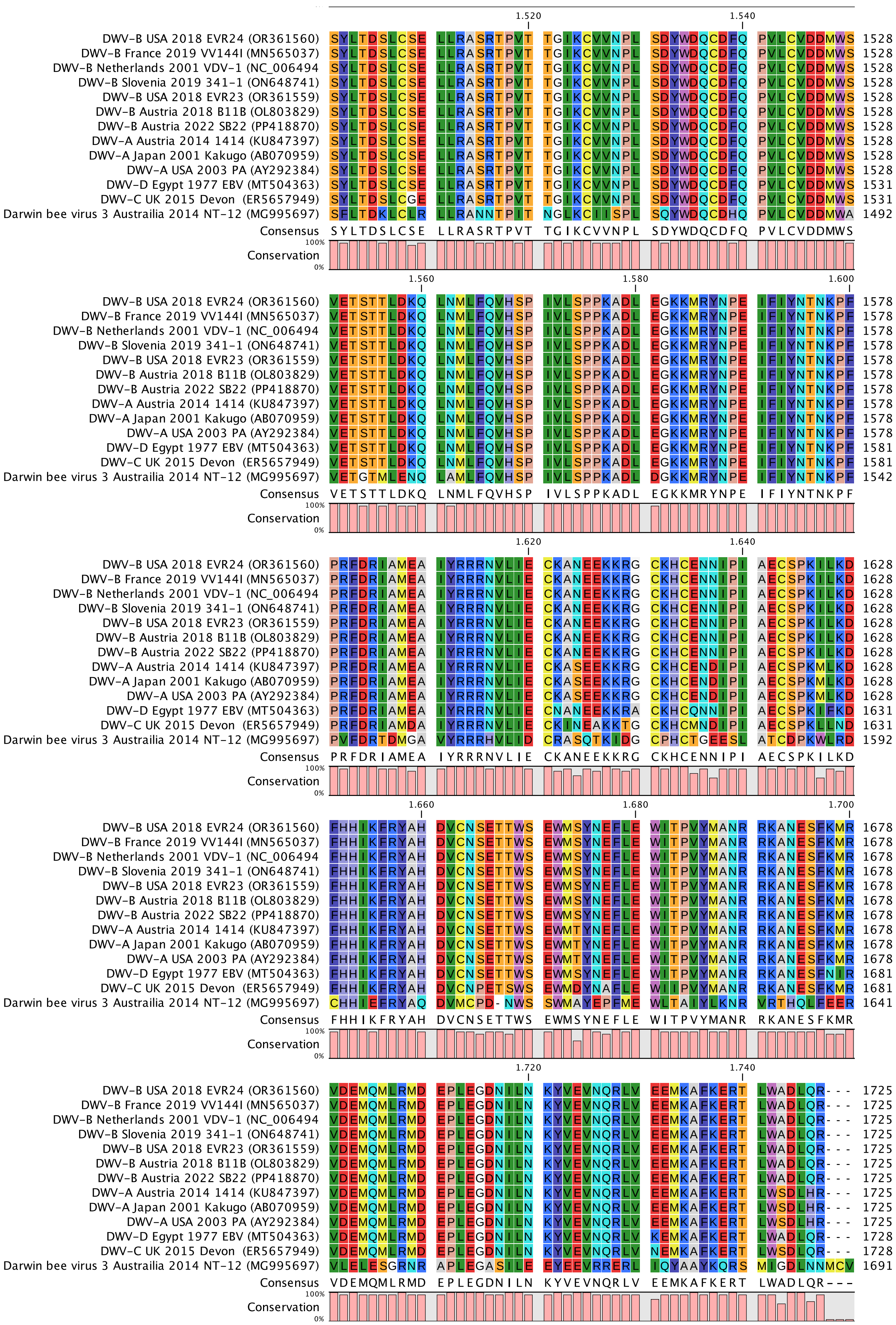
1.520 1.540
DWV-B USA 2018 EVR24 (OR361560) SYLTDSLCSE LLRASRTPVT TGIKCVVNPL SDYWDQCDFQ PVLCVDDMWS 1528
DWV-B France 2019 VV144I (MN565037) SYLTDSLCSE LLRASRTPVT TGIKCVVNPL SDYWDQCDFQ PVLCVDDMWS 1528
DWV-B Netherlands 2001 VDV-1 (NC_006494 SYLTDSLCSE LLRASRTPVT TGIKCVVNPL SDYWDQCDFQ PVLCVDDMWS 1528
DWV-B Slovenia 2019 341-1 (ON648741) SYLTDSLCSE LLRASRTPVT TGIKCVVNPL SDYWDQCDFQ PVLCVDDMWS 1528
DWV-B USA 2018 EVR23 (OR361559) SYLTDSLCSE LLRASRTPVT TGIKCVVNPL SDYWDQCDFQ PVLCVDDMWS 1528
DWV-B Austria 2018 B11B (OL803829) SYLTDSLCSE LLRASRTPVT TGIKCVVNPL SDYWDQCDFQ PVLCVDDMWS 1528
DWV-B Austria 2022 SB22 (PP418870) SYLTDSLCSE LLRASRTPVT TGIKCVVNPL SDYWDQCDFQ PVLCVDDMWS 1528
DWV-A Austria 2014 1414 (KU847397) SYLTDSLCSE LLRASRTPVT TGIKCVVNPL SDYWDQCDFQ PVLCVDDMWS 1528
DWV-A Japan 2001 Kakugo (AB070959) SYLTDSLCSE LLRASRTPVT TGIKCVVNPL SDYWDQCDFQ PVLCVDDMWS 1528
DWV-A USA 2003 PA (AY292384) SYLTDSLCSE LLRASRTPVT TGIKCVVNPL SDYWDQCDFQ PVLCVDDMWS 1528
DWV-D Egypt 1977 EBV (MT504363) SYLTDSLCSE LLRASRTPVT TGIKCVVNPL SDYWDQCDFQ PVLCVDDMWS 1531
DWV-C UK 2015 Devon (ER5657949) SYLTDSLCGE LLRASRTPVT TGIKCVVNPL SDYWDQCDFQ PVLCVDDMWS 1531
Darwin bee virus 3 Austrailia 2014 NT-12 (MG995697) SFLTDKLCLR LLRANNTPIT NGLKCIISPL SQYWDQCDHQ PVLCVDDMWA 1492
Consensus SYLTDSLCSE LLRASRTPVT TGIKCVVNPL SDYWDQCDFQ PVLCVDDMWS
Conservation 100% 0%
1.560 1.580 1.600
DWV-B USA 2018 EVR24 (OR361560) VETSTTLDKQ LNMLFQVHSP IVLSPPKADL EGKKMRYNPE IFIYNTNKPF 1578
DWV-B France 2019 VV144I (MN565037) VETSTTLDKQ LNMLFQVHSP IVLSPPKADL EGKKMRYNPE IFIYNTNKPF 1578
DWV-B Netherlands 2001 VDV-1 (NC_006494 VETSTTLDKQ LNMLFQVHSP IVLSPPKADL EGKKMRYNPE IFIYNTNKPF 1578
DWV-B Slovenia 2019 341-1 (ON648741) VETSTTLDKQ LNMLFQVHSP IVLSPPKADL EGKKMRYNPE IFIYNTNKPF 1578
DWV-B USA 2018 EVR23 (OR361559) VETSTTLDKQ LNMLFQVHSP IVLSPPKADL EGKKMRYNPE IFIYNTNKPF 1578
DWV-B Austria 2018 B11B (OL803829) VETSTTLDKQ LNMLFQVHSP IVLSPPKADL EGKKMRYNPE IFIYNTNKPF 1578
DWV-B Austria 2022 SB22 (PP418870) VETSTTLDKQ LNMLFQVHSP IVLSPPKADL EGKKMRYNPE IFIYNTNKPF 1578
DWV-A Austria 2014 1414 (KU847397) VETSTTLDKQ LNMLFQVHSP IVLSPPKADL EGKKMRYNPE IFIYNTNKPF 1578
DWV-A Japan 2001 Kakugo (AB070959) VETSTTLDKQ LNMLFQVHSP IVLSPPKADL EGKKMRYNPE IFIYNTNKPF 1578
DWV-A USA 2003 PA (AY292384) VETSTTLDKQ LNMLFQVHSP IVLSPPKADL EGKKMRYNPE IFIYNTNKPF 1578
DWV-D Egypt 1977 EBV (MT504363) VETSTTLDKQ LNMLFQVHSP IVLSPPKADL EGKKMRYNPE IFIYNTNKPF 1581
DWV-C UK 2015 Devon (ER5657949) VETSTTLDKQ LNMLFQVHSP IVLSPPKADL EGKKMRYNPE IFIYNTNKPF 1581
Darwin bee virus 3 Austrailia 2014 NT-12 (MG995697) VETGTMLENQ LAMLFQVHSP IVLSPPKADL DGKKMRYNPE IFIYNTNKPF 1542
Consensus VETSTTLDKQ LNMLFQVHSP IVLSPPKADL EGKKMRYNPE IFIYNTNKPF
Conservation 100% 0%
1.620 1.640
DWV-B USA 2018 EVR24 (OR361560) PRFDRIAMEA IYRRRNVLIE CKANEEKKRG CKHCENNIPI AECSPKILKD 1628
DWV-B France 2019 VV144I (MN565037) PRFDRIAMEA IYRRRNVLIE CKANEEKKRG CKHCENNIPI AECSPKILKD 1628
DWV-B Netherlands 2001 VDV-1 (NC_006494 PRFDRIAMEA IYRRRNVLIE CKANEEKKRG CKHCENNIPI AECSPKILKD 1628
DWV-B Slovenia 2019 341-1 (ON648741) PRFDRIAMEA IYRRRNVLIE CKANEEKKRG CKHCENNIPI AECSPKILKD 1628
DWV-B USA 2018 EVR23 (OR361559) PRFDRIAMEA IYRRRNVLIE CKANEEKKRG CKHCENNIPI AECSPKILKD 1628
DWV-B Austria 2018 B11B (OL803829) PRFDRIAMEA IYRRRNVLIE CKANEEKKRG CKHCENNIPI AECSPKILKD 1628
DWV-B Austria 2022 SB22 (PP418870) PRFDRIAMEA IYRRRNVLIE CKANEEKKRG CKHCENNIPI AECSPKILKD 1628
DWV-A Austria 2014 1414 (KU847397) PRFDRIAMEA IYRRRNVLIE CKASEEKKRG CKHCENDIPI AECSPKMLKD 1628
DWV-A Japan 2001 Kakugo (AB070959) PRFDRIAMEA IYRRRNVLIE CKASEEKKRG CKHCENDIPI AECSPKMLKD 1628
DWV-A USA 2003 PA (AY292384) PRFDRIAMEA IYRRRNVLIE CKASEEKKRG CKHCENDIPI AECSPKMLKD 1628
DWV-D Egypt 1977 EBV (MT504363) PRFDRIAMEA IYRRRNVLIE CNANEEKKRA CKHCQNNIPI AECSPKIFKD 1631
DWV-C UK 2015 Devon (ER5657949) PRFDRIAMDA IYRRRNVLIE CKINEAKKTG CKHCMNDIPI AECSPKLLND 1631
Darwin bee virus 3 Austrailia 2014 NT-12 (MG995697) PVFDRTDMGA VYRRRHVLID CRASQTKIDG CPHCTGEESL ATCDPKWLRD 1592
Consensus PRFDRIAMEA IYRRRNVLIE CKANEEKKRG CKHCENNIPI AECSPKILKD
Conservation 100% 0%
1.660 1.680 1.700
DWV-B USA 2018 EVR24 (OR361560) FHHIKFRYAH DVCNSETTWS EWMSYNEFLE WITPVYMANR RKANESFKMR 1678
DWV-B France 2019 VV144I (MN565037) FHHIKFRYAH DVCNSETTWS EWMSYNEFLE WITPVYMANR RKANESFKMR 1678
DWV-B Netherlands 2001 VDV-1 (NC_006494 FHHIKFRYAH DVCNSETTWS EWMSYNEFLE WITPVYMANR RKANESFKMR 1678
DWV-B Slovenia 2019 341-1 (ON648741) FHHIKFRYAH DVCNSETTWS EWMSYNEFLE WITPVYMANR RKANESFKMR 1678
DWV-B USA 2018 EVR23 (OR361559) FHHIKFRYAH DVCNSETTWS EWMSYNEFLE WITPVYMANR RKANESFKMR 1678
DWV-B Austria 2018 B11B (OL803829) FHHIKFRYAH DVCNSETTWS EWMSYNEFLE WITPVYMANR RKANESFKMR 1678
DWV-B Austria 2022 SB22 (PP418870) FHHIKFRYAH DVCNSETTWS EWMSYNEFLE WITPVYMANR RKANESFKMR 1678
DWV-A Austria 2014 1414 (KU847397) FHHIKFRYAH DVCNSETTWS EWMTYNEFLE WITPVYMANR RKANESFKMR 1678
DWV-A Japan 2001 Kakugo (AB070959) FHHIKFRYAH DVCNSETTWS EWMTYNEFLE WITPVYMANR RKANESFKMR 1678
DWV-A USA 2003 PA (AY292384) FHHIKFRYAH DVCNSETTWS EWMTYNEFLE WITPVYMANR RKANESFKMR 1678
DWV-D Egypt 1977 EBV (MT504363) FHHIKFRYAH DVCNSETTWS EWMSYNEFLE WITPVYMANR RKANESFNIR 1681
DWV-C UK 2015 Devon (ER5657949) FHHIKFRYAH DVCNPETSWS EWMDYNAFLE WIIPVYMANR RKANESFKMR 1681
Darwin bee virus 3 Austrailia 2014 NT-12 (MG995697) CHHIEFRYAQ DVMCPD-NWS SWMAYEPFME WLTAIYLKNR VRTHQLFEER 1641
Consensus FHHIKFRYAH DVCNSETTWS EWMSYNEFLE WITPVYMANR RKANESFKMR
Conservation 100% 0%
1.720 1.740
DWV-B USA 2018 EVR24 (OR361560) VDEMQMLRMD EPLEGDNILN KYVEVNQRLV EEMKAFKERT LWADLQR--- 1725
DWV-B France 2019 VV144I (MN565037) VDEMQMLRMD EPLEGDNILN KYVEVNQRLV EEMKAFKERT LWADLQR--- 1725
DWV-B Netherlands 2001 VDV-1 (NC_006494 VDEMQMLRMD EPLEGDNILN KYVEVNQRLV EEMKAFKERT LWADLQR--- 1725
DWV-B Slovenia 2019 341-1 (ON648741) VDEMQMLRMD EPLEGDNILN KYVEVNQRLV EEMKAFKERT LWADLQR--- 1725
DWV-B USA 2018 EVR23 (OR361559) VDEMQMLRMD EPLEGDNILN KYVEVNQRLV EEMKAFKERT LWADLQR--- 1725
DWV-B Austria 2018 B11B (OL803829) VDEMQMLRMD EPLEGDNILN KYVEVNQRLV EEMKAFKERT LWADLQR--- 1725
DWV-B Austria 2022 SB22 (PP418870) VDEMQMLRMD EPLEGDNILN KYVEVNQRLV EEMKAFKERT LWADLQR--- 1725
DWV-A Austria 2014 1414 (KU847397) VDEMQMLRMD EPLEGDNILN KYVEVNQRLV EEMKAFKERT LWSDLHR--- 1725
DWV-A Japan 2001 Kakugo (AB070959) VDEMQMLRMD EPLEGDNILN KYVEVNQRLV EEMKAFKERT LWSDLHR--- 1725
DWV-A USA 2003 PA (AY292384) VDEMQMLRMD EPLEGDNILN KYVEVNQRLV EEMKAFKERT LWSDLHR--- 1725
DWV-D Egypt 1977 EBV (MT504363) VDEMQMLRMD EPLEGDNILN KYVEVNQRLV KEMKAFKERT LWADLQR--- 1728
DWV-C UK 2015 Devon (ER5657949) VDEMQMLRMD EPLEGDNILN KYVEVNQRLV NEMKAFKERT LWSDLQR--- 1728
Darwin bee virus 3 Austrailia 2014 NT-12 (MG995697) VLELESGRNR APLEGASILE EYEEVRRERL IQYAAYKQRS MIGDLNNMCV 1691
Consensus VDEMQMLRMD EPLEGDNILN KYVEVNQRLV EEMKAFKERT LWADLQR---
Conservation 100% 0%

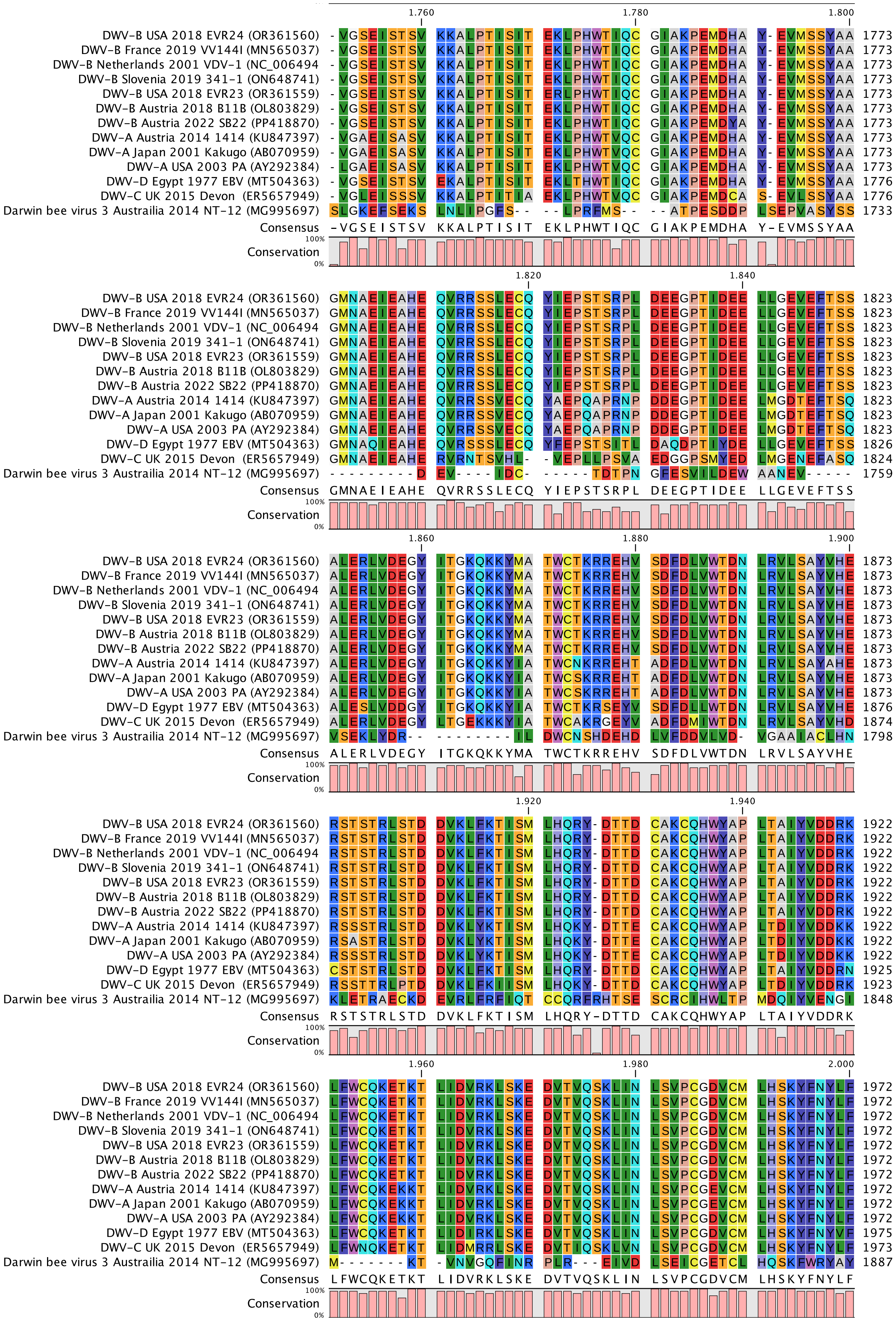
1.760
1.780
1.800
1.820
1.840
1.860
1.880
1.900
1.920
1.940
1.960
1.980
2.000
DWV-B USA 2018 EVR24 (OR361560)
DWV-B France 2019 VV144I (MN565037)
DWV-B Netherlands 2001 VDV-1 (NC_006494
DWV-B Slovenia 2019 341-1 (ON648741)
DWV-B USA 2018 EVR23 (OR361559)
DWV-B Austria 2018 B11B (OL803829)
DWV-B Austria 2022 SB22 (PP418870)
DWV-A Austria 2014 1414 (KU847397)
DWV-A Japan 2001 Kakugo (AB070959)
DWV-A USA 2003 PA (AY292384)
DWV-D Egypt 1977 EBV (MT504363)
DWV-C UK 2015 Devon (ER5657949)
Darwin bee virus 3 Austrailia 2014 NT-12 (MG995697)
Consensus -VGSEISTSV KKALPTISIT EKLPHWTIQC GIAKPEMDHA Y-EVMSSYAA
Consensus GMNAEIEAHE QVRRSSLECQ YIEPSTSRPL DEEGPTIDEE LLGEVEFTSS
Consensus ALERLVDEGY ITGKQKKYMA TWCTKRREHV SDFDLVWTDN LRVLSAYVHE
Consensus RSTSTRLSTD DVKLFKTISM LHQRY-DTTD CAKCQHWYAP LTAIYVDDRK
Consensus LFWCQKETKT LIDVRKLSKE DVTVQSKLIN LSVPCGDVCM LHSKYFNYLF
Conservation
100%
0%

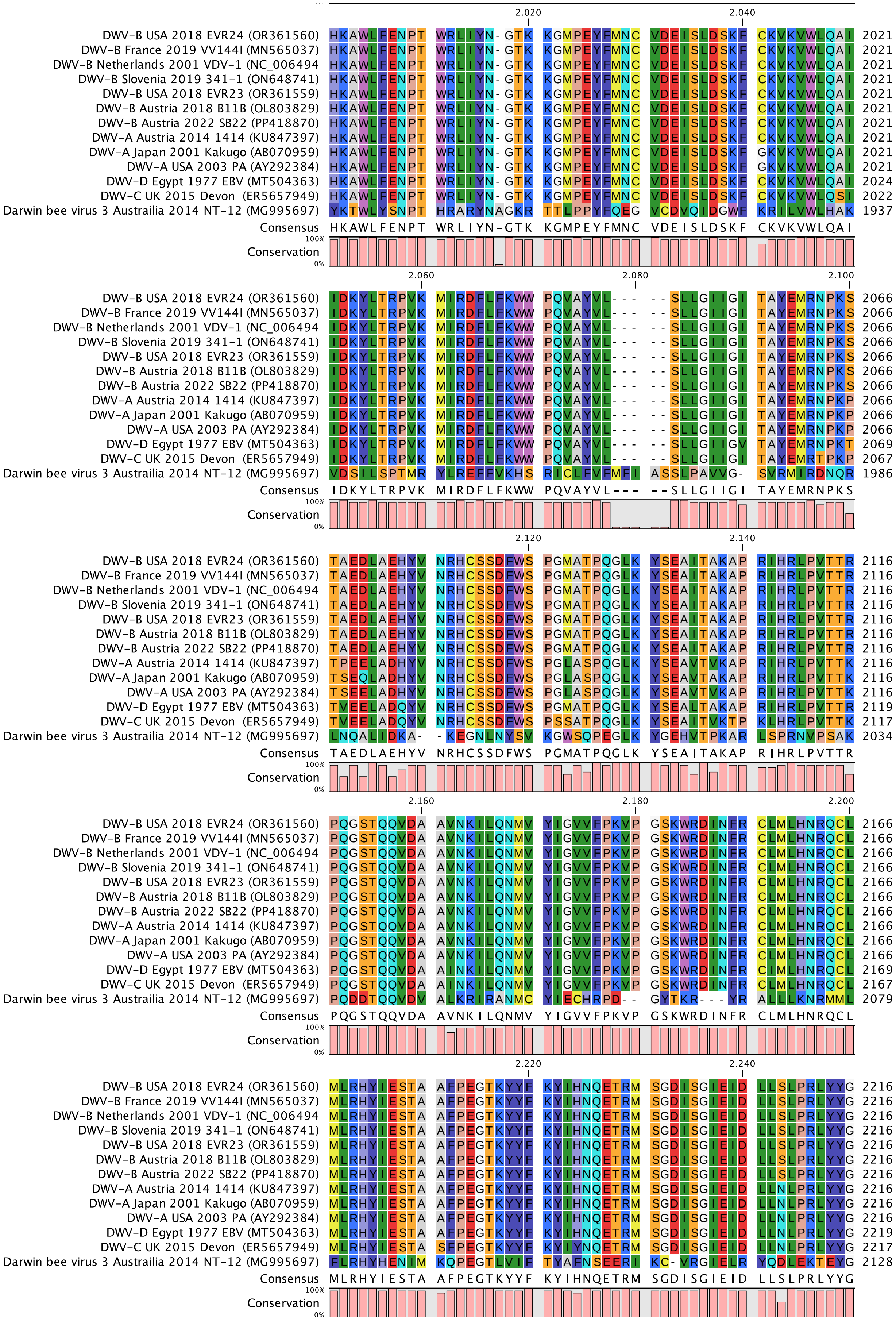
2.020
2.040
DWV-B USA 2018 EVR24 (OR361560) HKAWLFENPT WRLIYN-GTK KGMPEYFMNC VDEISLDSKF CKVKVWLQAI 2021
DWV-B France 2019 VV144I (MN565037) HKAWLFENPT WRLIYN-GTK KGMPEYFMNC VDEISLDSKF CKVKVWLQAI 2021
DWV-B Netherlands 2001 VDV-1 (NC_006494 HKAWLFENPT WRLIYN-GTK KGMPEYFMNC VDEISLDSKF CKVKVWLQAI 2021
DWV-B Slovenia 2019 341-1 (ON648741) HKAWLFENPT WRLIYN-GTK KGMPEYFMNC VDEISLDSKF CKVKVWLQAI 2021
DWV-B USA 2018 EVR23 (OR361559) HKAWLFENPT WRLIYN-GTK KGMPEYFMNC VDEISLDSKF CKVKVWLQAI 2021
DWV-B Austria 2018 B11B (OL803829) HKAWLFENPT WRLIYN-GTK KGMPEYFMNC VDEISLDSKF CKVKVWLQAI 2021
DWV-B Austria 2022 SB22 (PP418870) HKAWLFENPT WRLIYN-GTK KGMPEYFMNC VDEISLDSKF CKVKVWLQAI 2021
DWV-A Austria 2014 1414 (KU847397) HKAWLFENPT WRLIYN-GTK KGMPEYFMNC VDEISLDSKF CKVKVWLQAI 2021
DWV-A Japan 2001 Kakugo (AB070959) HKAWLFENPT WRLIYN-GTK KGMPEYFMNC VDEISLDSKF GKVKVWLQAI 2021
DWV-A USA 2003 PA (AY292384) HKAWLFENPT WRLIYN-GTK KGMPEYFMNC VDEISLDSKF GKVKVWLQAI 2021
DWV-D Egypt 1977 EBV (MT504363) HKAWLFENPT WRLIYN-GTK KGMPEYFMNC VDEISLDSKF CKVKVWLQAI 2024
DWV-C UK 2015 Devon (ER5657949) HKAWLFENPT WRLIYN-GTK KGMPEYFMNC VDEISLDSKF CKVKVWLQSI 2022
Darwin bee virus 3 Austrailia 2014 NT-12 (MG995697) YKTWLYSNPT HRARYNAGKR TTLPPYFQEG VCDVQIDGWF KRILVWLHAK 1937
Consensus HKAWLFENPT WRLIYN-GTK KGMPEYFMNC VDEISLDSKF CKVKVWLQAI
100%
Conservation
0%
2.060
2.080
2.100
DWV-B USA 2018 EVR24 (OR361560) IDKYLTRPVK MIRDFLFKWW PQVAYVL--- --SLLGIIGI TAYEMRNPKS 2066
DWV-B France 2019 VV144I (MN565037) IDKYLTRPVK MIRDFLFKWW PQVAYVL--- --SLLGIIGI TAYEMRNPKS 2066
DWV-B Netherlands 2001 VDV-1 (NC_006494 IDKYLTRPVK MIRDFLFKWW PQVAYVL--- --SLLGIIGI TAYEMRNPKS 2066
DWV-B Slovenia 2019 341-1 (ON648741) IDKYLTRPVK MIRDFLFKWW PQVAYVL--- --SLLGIIGI TAYEMRNPKS 2066
DWV-B USA 2018 EVR23 (OR361559) IDKYLTRPVK MIRDFLFKWW PQVAYVL--- --SLLGIIGI TAYEMRNPKS 2066
DWV-B Austria 2018 B11B (OL803829) IDKYLTRPVK MIRDFLFKWW PQVAYVL--- --SLLGIIGI TAYEMRNPKS 2066
DWV-B Austria 2022 SB22 (PP418870) IDKYLTRPVK MIRDFLFKWW PQVAYVL--- --SLLGIIGI TAYEMRNPKS 2066
DWV-A Austria 2014 1414 (KU847397) IDKYLTRPVK MIRDFLFKWW PQVAYVL--- --SLLGIIGI TAYEMRNPKP 2066
DWV-A Japan 2001 Kakugo (AB070959) IDKYLTRPVK MIRDFLFKWW PQVAYVL--- --SLLGIIGI TAYEMRNPKP 2066
DWV-A USA 2003 PA (AY292384) IDKYLTRPVK MIRDFLFKWW PQVAYVL--- --SLLGIIGI TAYEMRNPKP 2066
DWV-D Egypt 1977 EBV (MT504363) IDKYLTRPVK MIRDFLFKWW PQVAYVL--- --SLLGIIGV TAYEMRNPKT 2069
DWV-C UK 2015 Devon (ER5657949) IDKYLTRPVK MIRDFLFKWW PQVAYVL--- --SLLGIIGI TAYEMRTPKP 2067
Darwin bee virus 3 Austrailia 2014 NT-12 (MG995697) VDSILSPTMR YLREFFVKHS RICLFVFMFI ASSLPAVVG- SVRMIRDNQR 1986
Consensus IDKYLTRPVK MIRDFLFKWW PQVAYVL--- --SLLGIIGI TAYEMRNPKS
100%
Conservation
0%
2.120
2.140
DWV-B USA 2018 EVR24 (OR361560) TAEDLAEHYV NRHCSSDFWS PGMATPQGLK YSEAITAKAP RIHRLPVTTR 2116
DWV-B France 2019 VV144I (MN565037) TAEDLAEHYV NRHCSSDFWS PGMATPQGLK YSEAITAKAP RIHRLPVTTR 2116
DWV-B Netherlands 2001 VDV-1 (NC_006494 TAEDLAEHYV NRHCSSDFWS PGMATPQGLK YSEAITAKAP RIHRLPVTTR 2116
DWV-B Slovenia 2019 341-1 (ON648741) TAEDLAEHYV NRHCSSDFWS PGMATPQGLK YSEAITAKAP RIHRLPVTTR 2116
DWV-B USA 2018 EVR23 (OR361559) TAEDLAEHYV NRHCSSDFWS PGMATPQGLK YSEAITAKAP RIHRLPVTTR 2116
DWV-B Austria 2018 B11B (OL803829) TAEDLAEHYV NRHCSSDFWS PGMATPQGLK YSEAITAKAP RIHRLPVTTR 2116
DWV-B Austria 2022 SB22 (PP418870) TAEDLAEHYV NRHCSSDFWS PGMATPQGLK YSEAITAKAP RIHRLPVTTR 2116
DWV-A Austria 2014 1414 (KU847397) TPEELADHYV NRHCSSDFWS PGLASPQGLK YSEAVTVKAP RIHRLPVTTK 2116
DWV-A Japan 2001 Kakugo (AB070959) TSEQLADHYV NRHCSSDFWS PGLASPQGLK YSEAVTAKAP RIHRLPVTTK 2116
DWV-A USA 2003 PA (AY292384) TSEELADHYV NRHCSSDFWS PGLASPQGLK YSEAVTVKAP RIHRLPVTTK 2116
DWV-D Egypt 1977 EBV (MT504363) TVEELADQYV NRHCSSDFWS PGMATPQGLK YSEALTAKAP RIHRLPVTTR 2119
DWV-C UK 2015 Devon (ER5657949) TVEELADQYV NRHCSSDFWS PSSATPQGLK YSEAITVKTP KLHRLPVTTK 2117
Darwin bee virus 3 Austrailia 2014 NT-12 (MG995697) LNQALIDKA- -KEGNLNYSV KGWSQPEGLK YGEHVTPKAR LSPRNVPSAK 2034
Consensus TAEDLAEHYV NRHCSSDFWS PGMATPQGLK YSEAITAKAP RIHRLPVTTR
100%
Conservation
0%
2.160
2.180
2.200
DWV-B USA 2018 EVR24 (OR361560) PQGSTQQVDA AVNKILQNMV YIGVVFPKVP GSKWRDINFR CLMLHNRQCL 2166
DWV-B France 2019 VV144I (MN565037) PQGSTQQVDA AVNKILQNMV YIGVVFPKVP GSKWRDINFR CLMLHNRQCL 2166
DWV-B Netherlands 2001 VDV-1 (NC_006494 PQGSTQQVDA AVNKILQNMV YIGVVFPKVP GSKWRDINFR CLMLHNRQCL 2166
DWV-B Slovenia 2019 341-1 (ON648741) PQGSTQQVDA AVNKILQNMV YIGVVFPKVP GSKWRDINFR CLMLHNRQCL 2166
DWV-B USA 2018 EVR23 (OR361559) PQGSTQQVDA AVNKILQNMV YIGVVFPKVP GSKWRDINFR CLMLHNRQCL 2166
DWV-B Austria 2018 B11B (OL803829) PQGSTQQVDA AVNKILQNMV YIGVVFPKVP GSKWRDINFR CLMLHNRQCL 2166
DWV-B Austria 2022 SB22 (PP418870) PQGSTQQVDA AVNKILQNMV YIGVVFPKVP GSKWRDINFR CLMLHNRQCL 2166
DWV-A Austria 2014 1414 (KU847397) PQGSTQQVDA AVNKILQNMV YIGVVFPKVP GSKWRDINFR CLMLHNRQCL 2166
DWV-A Japan 2001 Kakugo (AB070959) PQGSTQQVDA AVNKILQNMV YIGVVFPKVP GSKWRDINFR CLMLHNRQCL 2166
DWV-A USA 2003 PA (AY292384) PQGSTQQVDA AVNKILQNMV YIGVVFPKVP GSKWRDINFR CLMLHNRQCL 2166
DWV-D Egypt 1977 EBV (MT504363) PQGSTQQVDA AINKILQNMV YIGVVFPKVP GSKWRDINFR CIMLHNRQCL 2169
DWV-C UK 2015 Devon (ER5657949) PQGSTQQVDA AINKILQNMV YIGVVFPKVP GSKWRDINFR CLMLHNRQCL 2167
Darwin bee virus 3 Austrailia 2014 NT-12 (MG995697) PQDDTQQVDV ALKRIRANMC YIECHRPD-- GYTKR---YR ALLLKNRMML 2079
Consensus PQGSTQQVDA AVNKILQNMV YIGVVFPKVP GSKWRDINFR CLMLHNRQCL
100%
Conservation
0%
2.220
2.240
DWV-B USA 2018 EVR24 (OR361560) MLRHYIESTA AFPEGTKYYF KYIHNQETRM SGDISGIEID LLSLPRLYYG 2216
DWV-B France 2019 VV144I (MN565037) MLRHYIESTA AFPEGTKYYF KYIHNQETRM SGDISGIEID LLSLPRLYYG 2216
DWV-B Netherlands 2001 VDV-1 (NC_006494 MLRHYIESTA AFPEGTKYYF KYIHNQETRM SGDISGIEID LLSLPRLYYG 2216
DWV-B Slovenia 2019 341-1 (ON648741) MLRHYIESTA AFPEGTKYYF KYIHNQETRM SGDISGIEID LLSLPRLYYG 2216
DWV-B USA 2018 EVR23 (OR361559) MLRHYIESTA AFPEGTKYYF KYIHNQETRM SGDISGIEID LLSLPRLYYG 2216
DWV-B Austria 2018 B11B (OL803829) MLRHYIESTA AFPEGTKYYF KYIHNQETRM SGDISGIEID LLSLPRLYYG 2216
DWV-B Austria 2022 SB22 (PP418870) MLRHYIESTA AFPEGTKYYF KYIHNQETRM SGDISGIEID LLSLPRLYYG 2216
DWV-A Austria 2014 1414 (KU847397) MLRHYIESTA AFPEGTKYYF KYIHNQETRM SGDISGIEID LLNLPRLYYG 2216
DWV-A Japan 2001 Kakugo (AB070959) MLRHYIESTA AFPEGTKYYF KYIHNQETRM SGDISGIEID LLNLPRLYYG 2216
DWV-A USA 2003 PA (AY292384) MLRHYIESTA AFPEGTKYYF KYIHNQETRM SGDISGIEID LLNLPRLYYG 2216
DWV-D Egypt 1977 EBV (MT504363) MLRHYIESTA AFPEGTKYYF KYIHNQETRM SGDISGIEID LLNLPRLYYG 2219
DWV-C UK 2015 Devon (ER5657949) MLRHYIESTA SFPEGTKYYF KYIYNQETRM SGDISGIEID LLNLPRLYYG 2217
Darwin bee virus 3 Austrailia 2014 NT-12 (MG995697) FLRHYHENIM KQPEGTLVIF TYAFNSEERI KC-VRGIELR YQDLEKTEYG 2128
Consensus MLRHYIESTA AFPEGTKYYF KYIHNQETRM SGDISGIEID LLSLPRLYYG
100%
Conservation
0%

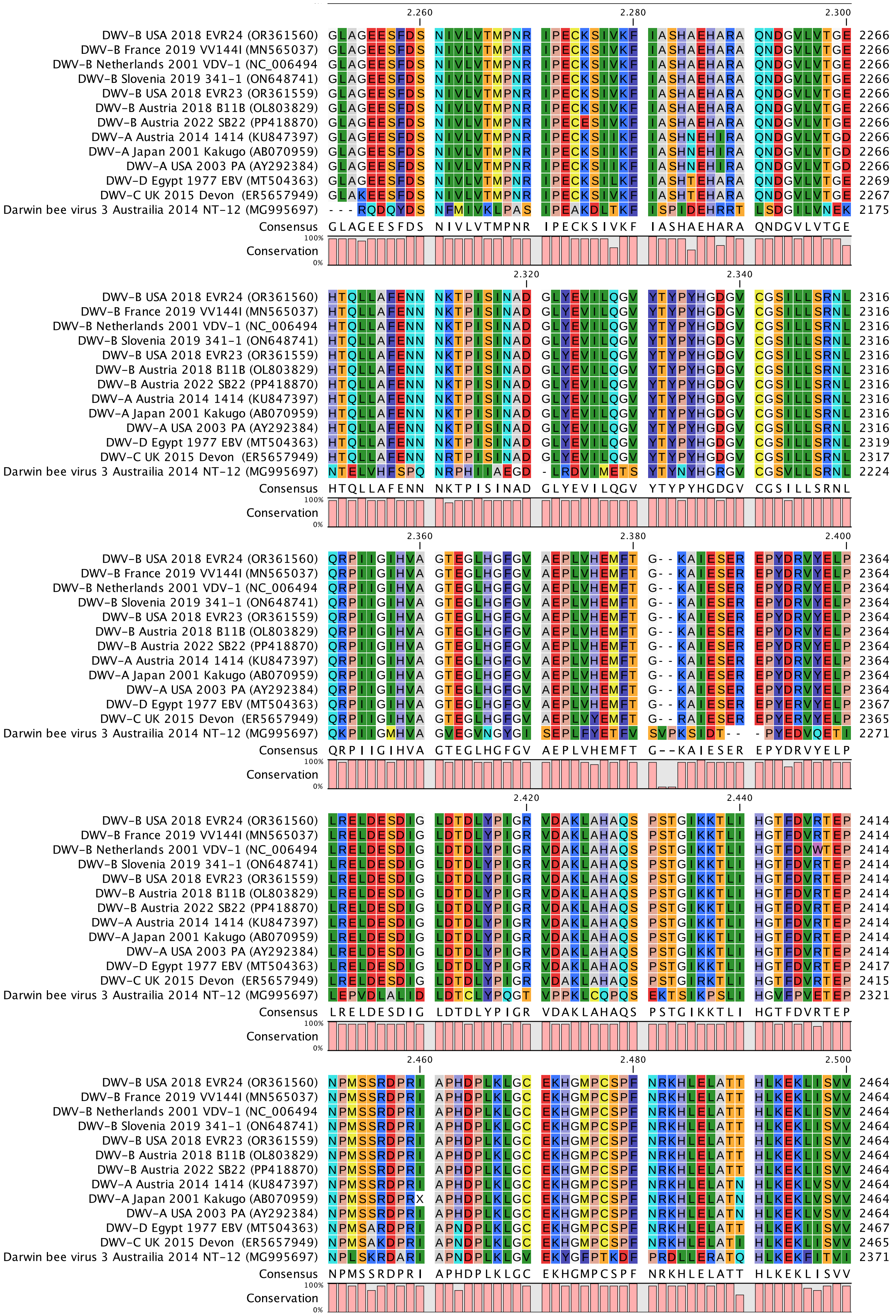

2.260
2.280
2.300
DWV-B USA 2018 EVR24 (OR361560)
DWV-B France 2019 VV1441 (MN565037)
DWV-B Netherlands 2001 VDV-1 (NC_006494
DWV-B Slovenia 2019 341-1 (ON648741)
DWV-B USA 2018 EVR23 (OR361559)
DWV-B Austria 2018 B11B (OL803829)
DWV-B Austria 2022 SB22 (PP418870)
DWV-A Austria 2014 1414 (KU847397)
DWV-A Japan 2001 Kakugo (AB070959)
DWV-A USA 2003 PA (AY292384)
DWV-D Egypt 1977 EBV (MT504363)
DWV-C UK 2015 Devon (ER5657949)
Darwin bee virus 3 Austrailia 2014 NT-12 (MG995697)
Consensus GLAGEESFDS NIVLVTMPNR IPECKSIVKF IASHAEHARA QNDGVLVTGE
Conservation
100%
0%
2.320
2.340
Consensus HTQLLAFENN NKTPISINAD GLYEVILQGV YTYPYHGDGV CGSILLSRNL
2.360
2.380
2.400
Consensus QRPIIGIHVA GTEGLHGFGV AEPLVHEMFT G--KAIESER EPYDRVYELP
2.420
2.440
Consensus LRELDESDIG LDTDLYPIGR VDAKLAHAQS PSTGIKKTLI HGTFDVRTEP
2.460
2.480
2.500
Consensus NPMSSRDPRI APHDPLKLGC EKHGMPCSPF NRKHLELATT HLKEKLISVV

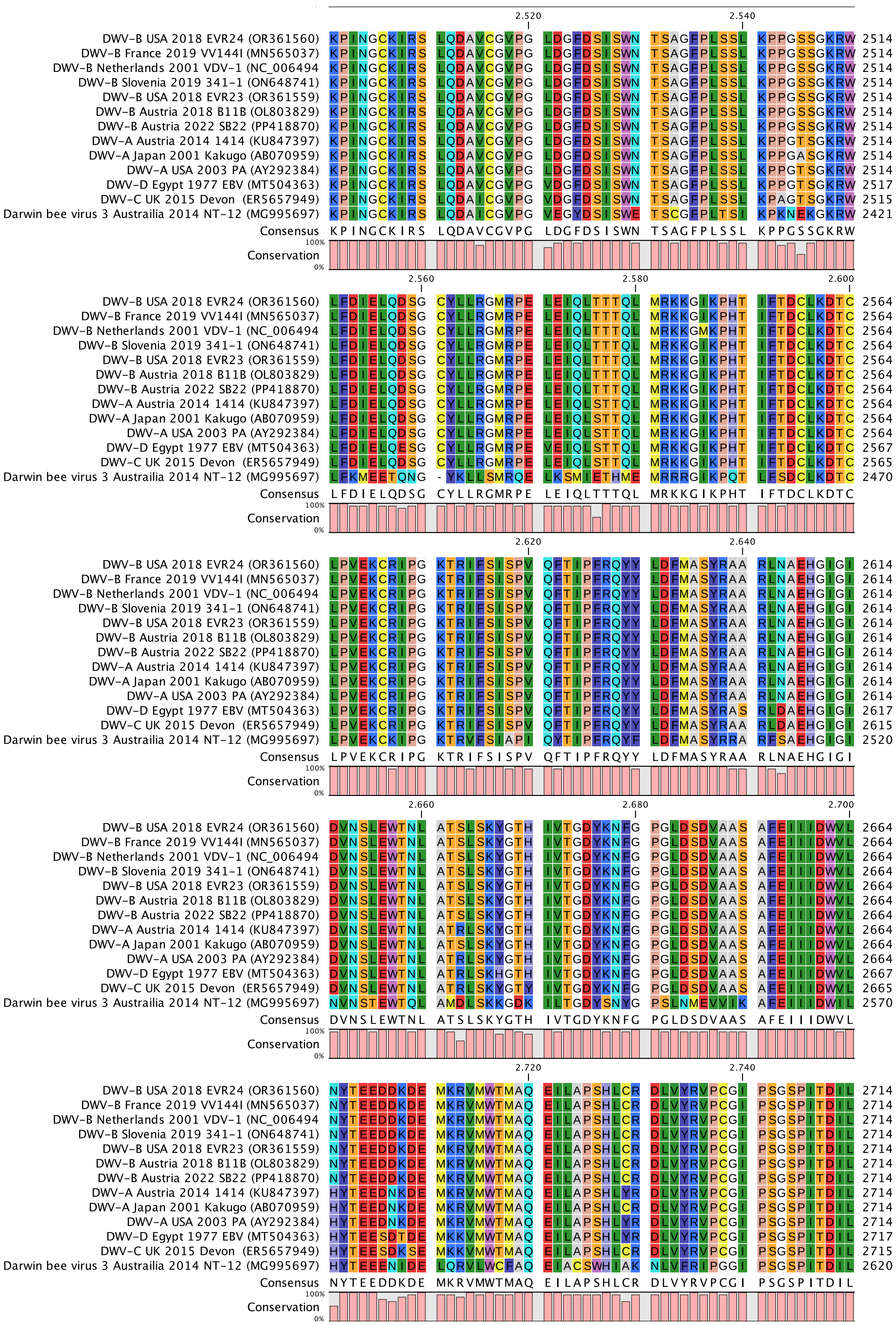
2.520 2.540
DWV-B USA 2018 EVR24 (OR361560) KPINGCKIRS LQDAVCGVPG LDGFDSISWN TSAGFPLSSL KPPGSSGKRW 2514
DWV-B France 2019 VV144I (MN565037) KPINGCKIRS LQDAVCGVPG LDGFDSISWN TSAGFPLSSL KPPGSSGKRW 2514
DWV-B Netherlands 2001 VDV-1 (NC_006494 KPINGCKIRS LQDAVCGVPG LDGFDSISWN TSAGFPLSSL KPPGSSGKRW 2514
DWV-B Slovenia 2019 341-1 (ON648741) KPINGCKIRS LQDAVCGVPG LDGFDSISWN TSAGFPLSSL KPPGSSGKRW 2514
DWV-B USA 2018 EVR23 (OR361559) KPINGCKIRS LQDAVCGVPG LDGFDSISWN TSAGFPLSSL KPPGSSGKRW 2514
DWV-B Austria 2018 B11B (OL803829) KPINGCKIRS LQDAVCGVPG LDGFDSISWN TSAGFPLSSL KPPGSSGKRW 2514
DWV-B Austria 2022 SB22 (PP418870) KPINGCKIRS LQDAVCGVPG LDGFDSISWN TSAGFPLSSL KPPGSSGKRW 2514
DWV-A Austria 2014 1414 (KU847397) KPINGCKIRS LQDAVCGVPG LDGFDSISWN TSAGFPLSSL KPPGTSGKRW 2514
DWV-A Japan 2001 Kakugo (AB070959) KPINGCKIRS LQDAVCGVPG LDGFDSISWN TSAGFPLSSL KPPGASGKRW 2514
DWV-A USA 2003 PA (AY292384) KPINGCKIRS LQDAVCGVPG LDGFDSISWN TSAGFPLSSL KPPGTSGKRW 2514
DWV-D Egypt 1977 EBV (MT504363) KPINGCKIRS LQDAVCGVPG VDGFDSISWN TSAGFPLSSL KPPGTSGKRW 2517
DWV-C UK 2015 Devon (ER5657949) KPINGCKIRS LQDAICGVPG VDGFDSISWN TSAGFPLSSL KPAGTSGKRW 2515
Darwin bee virus 3 Austrailia 2014 NT-12 (MG995697) KPINGCKIRS LQDAICGVPG VEGYDSISWE TSCGFPLTSI KPKNEKGKRW 2421
Consensus KPINGCKIRS LQDAVCGVPG LDGFDSISWN TSAGFPLSSL KPPGSSGKRW
Conservation 100% 0%
2.560 2.580 2.600
DWV-B USA 2018 EVR24 (OR361560) LFDIELQDSG CYLLRGMRPE LEIQLTTTQL MRKKGIKPHT IFTDCLKDTC 2564
DWV-B France 2019 VV144I (MN565037) LFDIELQDSG CYLLRGMRPE LEIQLTTTQL MRKKGIKPHT IFTDCLKDTC 2564
DWV-B Netherlands 2001 VDV-1 (NC_006494 LFDIELQDSG CYLLRGMRPE LEIQLTTTQL MRKKGMKPHT IFTDCLKDTC 2564
DWV-B Slovenia 2019 341-1 (ON648741) LFDIELQDSG CYLLRGMRPE LEIQLTTTQL MRKKGIKPHT IFTDCLKDTC 2564
DWV-B USA 2018 EVR23 (OR361559) LFDIELQDSG CYLLRGMRPE LEIQLTTTQL MRKKGIKPHT IFTDCLKDTC 2564
DWV-B Austria 2018 B11B (OL803829) LFDIELQDSG CYLLRGMRPE LEIQLTTTQL MRKKGIKPHT IFTDCLKDTC 2564
DWV-B Austria 2022 SB22 (PP418870) LFDIELQDSG CYLLRGMRPE LEIQLTTTQL MRKKGIKPHT IFTDCLKDTC 2564
DWV-A Austria 2014 1414 (KU847397) LFDIELQDSG CYLLRGMRPE LEIQLSTTQL MRKKGIKPHT IFTDCLKDTC 2564
DWV-A Japan 2001 Kakugo (AB070959) LFDIELQDSG CYLLRGMRPE LEIQLSTTQL MRKKGIKPHT IFTDCLKDTC 2564
DWV-A USA 2003 PA (AY292384) LFDIELQDSG CYLLRGMRPE LEIQLSTTQL MRKKGIKPHT IFTDCLKDTC 2564
DWV-D Egypt 1977 EBV (MT504363) LFDIELQESG CYLLRGMRPE VEIQLSTTQL MRKKGIKPHT IFTDCLKDTC 2567
DWV-C UK 2015 Devon (ER5657949) LFDIELQDSG CYLLRGMRPE LEIQLSTTQL MRKKGIKPHT IFTDCLKDTC 2565
Darwin bee virus 3 Austrailia 2014 NT-12 (MG995697) LFKMEETQNG -YKLLSMRQE LKSMIETHME MRRRGIKPQT LFSDCLKDTC 2470
Consensus LFDIELQDSG CYLLRGMRPE LEIQLTTTQL MRKKGIKPHT IFTDCLKDTC
Conservation 100% 0%
2.620 2.640
DWV-B USA 2018 EVR24 (OR361560) LPVEKCRIPG KTRIFSISPV QFTIPFRQYY LDFMASYRAA RLNAEHGIGI 2614
DWV-B France 2019 VV144I (MN565037) LPVEKCRIPG KTRIFSISPV QFTIPFRQYY LDFMASYRAA RLNAEHGIGI 2614
DWV-B Netherlands 2001 VDV-1 (NC_006494 LPVEKCRIPG KTRIFSISPV QFTIPFRQYY LDFMASYRAA RLNAEHGIGI 2614
DWV-B Slovenia 2019 341-1 (ON648741) LPVEKCRIPG KTRIFSISPV QFTIPFRQYY LDFMASYRAA RLNAEHGIGI 2614
DWV-B USA 2018 EVR23 (OR361559) LPVEKCRIPG KTRIFSISPV QFTIPFRQYY LDFMASYRAA RLNAEHGIGI 2614
DWV-B Austria 2018 B11B (OL803829) LPVEKCRIPG KTRIFSISPV QFTIPFRQYY LDFMASYRAA RLNAEHGIGI 2614
DWV-B Austria 2022 SB22 (PP418870) LPVEKCRIPG KTRIFSISPV QFTIPFRQYY LDFMASYRAA RLNAEHGIGI 2614
DWV-A Austria 2014 1414 (KU847397) LPVEKCRIPG KTRIFSISPV QFTIPFRQYY LDFMASYRAA RLNAEHGIGI 2614
DWV-A Japan 2001 Kakugo (AB070959) LPVEKCRIPG KTRIFSISPV QFTIPFRQYY LDFMASYRAA RLNAEHGIGI 2614
DWV-A USA 2003 PA (AY292384) LPVEKCRIPG KTRIFSISPV QFTIPFRQYY LDFMASYRAA RLNAEHGIGI 2614
DWV-D Egypt 1977 EBV (MT504363) LPVEKCRIPG KTRIFSISPV QFTIPFRQYY LDFMASYRAS RLDAEHGIGI 2617
DWV-C UK 2015 Devon (ER5657949) LPVEKCRIPG KTRIFSISPV QFTIPFRQYY LDFMASYRAA RLDAEHGIGI 2615
Darwin bee virus 3 Austrailia 2014 NT-12 (MG995697) LPVEKCKIPG KTRVFSIAPI QYTIPFRQYF LDFMASYRRA RFSAEHGIGI 2520
Consensus LPVEKCRIPG KTRIFSISPV QFTIPFRQYY LDFMASYRAA RLNAEHGIGI
Conservation 100% 0%
2.660 2.680 2.700
DWV-B USA 2018 EVR24 (OR361560) DVNSLEWTNL ATSLSKYGTH IVTGDYKNFG PGLDSDVAAS AFEIIIDWVL 2664
DWV-B France 2019 VV144I (MN565037) DVNSLEWTNL ATSLSKYGTH IVTGDYKNFG PGLDSDVAAS AFEIIIDWVL 2664
DWV-B Netherlands 2001 VDV-1 (NC_006494 DVNSLEWTNL ATSLSKYGTH IVTGDYKNFG PGLDSDVAAS AFEIIIDWVL 2664
DWV-B Slovenia 2019 341-1 (ON648741) DVNSLEWTNL ATSLSKYGTH IVTGDYKNFG PGLDSDVAAS AFEIIIDWVL 2664
DWV-B USA 2018 EVR23 (OR361559) DVNSLEWTNL ATSLSKYGTH IVTGDYKNFG PGLDSDVAAS AFEIIIDWVL 2664
DWV-B Austria 2018 B11B (OL803829) DVNSLEWTNL ATSLSKYGTH IVTGDYKNFG PGLDSDVAAS AFEIIIDWVL 2664
DWV-B Austria 2022 SB22 (PP418870) DVNSLEWTNL ATSLSKYGTH IVTGDYKNFG PGLDSDVAAS AFEIIIDWVL 2664
DWV-A Austria 2014 1414 (KU847397) DVNSLEWTNL ATRLSKYGTH IVTGDYKNFG PGLDSDVAAS AFEIIIDWVL 2664
DWV-A Japan 2001 Kakugo (AB070959) DVNSLEWTNL ATSLSKYGTH IVTGDYKNFG PGLDSDVAAS AFEIIIDWVL 2664
DWV-A USA 2003 PA (AY292384) DVNSLEWTNL ATRLSKYGTH IVTGDYKNFG PGLDSDVAAS AFEIIIDWVL 2664
DWV-D Egypt 1977 EBV (MT504363) DVNSLEWTNL ATRLSKHGTH IVTGDYKNFG PGLDSDVAAS AFEIIIDWVL 2667
DWV-C UK 2015 Devon (ER5657949) DVNSLEWTNL ATRLSKYGTY IVTGDYKNFG PGLDSDVAAS AFEIIIDWVL 2665
Darwin bee virus 3 Austrailia 2014 NT-12 (MG995697) NVNSTEWTQL AMDLSKKGDK ILTGDYSNYG PSLNMEVVIK AFEIIIDWIL 2570
Consensus DVNSLEWTNL ATSLSKYGTH IVTGDYKNFG PGLDSDVAAS AFEIIIDWVL
Conservation 100% 0%
2.720 2.740
DWV-B USA 2018 EVR24 (OR361560) NYTEEDDKDE MKRVMWTMAQ EILAPSHLCR DLVYRVPCGI PSGSPITDIL 2714
DWV-B France 2019 VV144I (MN565037) NYTEEDDKDE MKRVMWTMAQ EILAPSHLCR DLVYRVPCGI PSGSPITDIL 2714
DWV-B Netherlands 2001 VDV-1 (NC_006494 NYTEEDDKDE MKRVMWTMAQ EILAPSHLCR DLVYRVPCGI PSGSPITDIL 2714
DWV-B Slovenia 2019 341-1 (ON648741) NYTEEDDKDE MKRVMWTMAQ EILAPSHLCR DLVYRVPCGI PSGSPITDIL 2714
DWV-B USA 2018 EVR23 (OR361559) NYTEEDDKDE MKRVMWTMAQ EILAPSHLCR DLVYRVPCGI PSGSPITDIL 2714
DWV-B Austria 2018 B11B (OL803829) NYTEEDDKDE MKRVMWTMAQ EILAPSHLCR DLVYRVPCGI PSGSPITDIL 2714
DWV-B Austria 2022 SB22 (PP418870) NYTEEDDKDE MKRVMWTMAQ EILAPSHLCR DLVYRVPCGI PSGSPITDIL 2714
DWV-A Austria 2014 1414 (KU847397) HYTEEDNKDE MKRVMWTMAQ EILAPSHLYR DLVYRVPCGI PSGSPITDIL 2714
DWV-A Japan 2001 Kakugo (AB070959) HYTEEDNKDE MKRVMWTMAQ EILAPSHLCR DLVYRVPCGI PSGSPITDIL 2714
DWV-A USA 2003 PA (AY292384) HYTEEDNKDE MKRVMWTMAQ EILAPSHLYR DLVYRVPCGI PSGSPITDIL 2714
DWV-D Egypt 1977 EBV (MT504363) HYTEESDTDE MKKVMWTMAQ EILAPSHLYR DLVYRVPCGI PSGSPITDIL 2717
DWV-C UK 2015 Devon (ER5657949) HYTEESDKSE MKKVMWTMAQ EILAPSHLCR DLVYRVPCGI PSGSPITDIL 2715
Darwin bee virus 3 Austrailia 2014 NT-12 (MG995697) HYTEEENIDE LQRVLWCFAQ EIACSWHIAK NLVFRIPGGI PSGSPITDIL 2620
Consensus NYTEEDDKDE MKRVMWTMAQ EILAPSHLCR DLVYRVPCGI PSGSPITDIL
Conservation 100% 0%

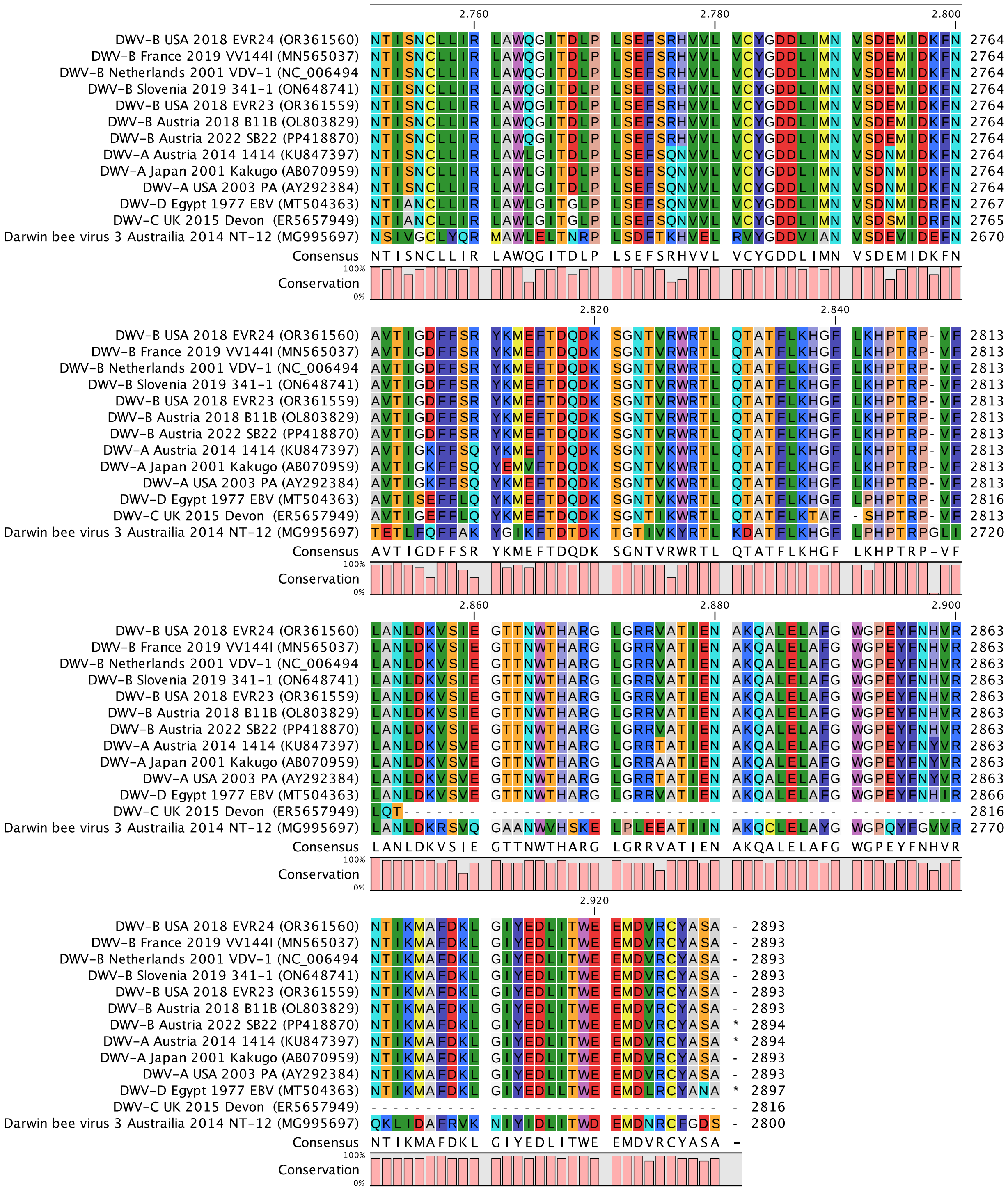
2.760 2.780 2.800
DWV-B USA 2018 EVR24 (OR361560) NTISNCLLIR LAWQGITDLP LSEFSRHVVL VCYGDDLIMN VSDEMIDKFN 2764
DWV-B France 2019 VV144I (MN565037) NTISNCLLIR LAWQGITDLP LSEFSRHVVL VCYGDDLIMN VSDEMIDKFN 2764
DWV-B Netherlands 2001 VDV-1 (NC_006494 NTISNCLLIR LAWQGITDLP LSEFSRHVVL VCYGDDLIMN VSDEMIDKFN 2764
DWV-B Slovenia 2019 341-1 (ON648741) NTISNCLLIR LAWQGITDLP LSEFSRHVVL VCYGDDLIMN VSDEMIDKFN 2764
DWV-B USA 2018 EVR23 (OR361559) NTISNCLLIR LAWQGITDLP LSEFSRHVVL VCYGDDLIMN VSDEMIDKFN 2764
DWV-B Austria 2018 B11B (OL803829) NTISNCLLIR LAWQGITDLP LSEFSRHVVL VCYGDDLIMN VSDEMIDKFN 2764
DWV-B Austria 2022 SB22 (PP418870) NTISNCLLIR LAWQGITDLP LSEFSRHVVL VCYGDDLIMN VSDEMIDKFN 2764
DWV-A Austria 2014 1414 (KU847397) NTISNCLLIR LAWLGITDLP LSEFSQNVVL VCYGDDLIMN VSDNMIDKFN 2764
DWV-A Japan 2001 Kakugo (AB070959) NTISNCLLIR LAWLGITDLP LSEFSQNVVL VCYGDDLIMN VSDNMIDKFN 2764
DWV-A USA 2003 PA (AY292384) NTISNCLLIR LAWLGITDLP LSEFSQNVVL VCYGDDLIMN VSDNMIDKFN 2764
DWV-D Egypt 1977 EBV (MT504363) NTIANCLLIR LAWLGITGLP LSEFSQNVVL VCYGDDLIMN VSDNMIDRFN 2767
DWV-C UK 2015 Devon (ER5657949) NTIANCLLIR LAWLGITGLP LSEFSQNVVL VCYGDDLIMN VSDSMIDRFN 2765
Darwin bee virus 3 Austrailia 2014 NT-12 (MG995697) NSIVGCLYQR MAWLELTNRP LSDFTKHVEL RVYGDDVIAN VSDEVIDEFN 2670
Consensus NTISNCLLIR LAWQGITDLP LSEFSRHVVL VCYGDDLIMN VSDEMIDKFN
Conservation 100% 0%
2.820 2.840
DWV-B USA 2018 EVR24 (OR361560) AVTIGDFFSR YKMEFTDQDK SGNTVRWRTL QTATFLKHGF LKHPTRP-VF 2813
DWV-B France 2019 VV144I (MN565037) AVTIGDFFSR YKMEFTDQDK SGNTVRWRTL QTATFLKHGF LKHPTRP-VF 2813
DWV-B Netherlands 2001 VDV-1 (NC_006494 AVTIGDFFSR YKMEFTDQDK SGNTVRWRTL QTATFLKHGF LKHPTRP-VF 2813
DWV-B Slovenia 2019 341-1 (ON648741) AVTIGDFFSR YKMEFTDQDK SGNTVRWRTL QTATFLKHGF LKHPTRP-VF 2813
DWV-B USA 2018 EVR23 (OR361559) AVTIGDFFSR YKMEFTDQDK SGNTVRWRTL QTATFLKHGF LKHPTRP-VF 2813
DWV-B Austria 2018 B11B (OL803829) AVTIGDFFSR YKMEFTDQDK SGNTVRWRTL QTATFLKHGF LKHPTRP-VF 2813
DWV-B Austria 2022 SB22 (PP418870) AVTIGDFFSR YKMEFTDQDK SGNTVRWRTL QTATFLKHGF LKHPTRP-VF 2813
DWV-A Austria 2014 1414 (KU847397) AVTIGKFFSQ YKMEFTDQDK SGNTVKWRTL QTATFLKHGF LKHPTRP-VF 2813
DWV-A Japan 2001 Kakugo (AB070959) AVTIGKFFSQ YEMVFTDQDK SGNTVKWRTL QTATFLKHGF LKHPTRP-VF 2813
DWV-A USA 2003 PA (AY292384) AVTIGKFFSQ YKMEFTDQDK SGNTVKWRTL QTATFLKHGF LKHPTRP-VF 2813
DWV-D Egypt 1977 EBV (MT504363) AVTISEFFLQ YKMEFTDQDK SGNTVKWRTL QTATFLKHGF LPHPTRP-VF 2816
DWV-C UK 2015 Devon (ER5657949) AVTIGEFFLQ YKMEFTDQDK SGNTIKWRTL QTATFLKTAF -SHPTRP-VF 2813
Darwin bee virus 3 Austrailia 2014 NT-12 (MG995697) TETLFQFFAK YGIKFTDTDK TGTIVKYRTL KDATFLKHGF LPHPTRPGLI 2720
Consensus AVTIGDFFSR YKMEFTDQDK SGNTVRWRTL QTATFLKHGF LKHPTRP-VF
Conservation 100% 0%
2.860 2.880 2.900
DWV-B USA 2018 EVR24 (OR361560) LANLDKVSIE GTTNWTHARG LGRRVATIEN AKQALELAFG WGPEYFNHVR 2863
DWV-B France 2019 VV144I (MN565037) LANLDKVSIE GTTNWTHARG LGRRVATIEN AKQALELAFG WGPEYFNHVR 2863
DWV-B Netherlands 2001 VDV-1 (NC_006494 LANLDKVSIE GTTNWTHARG LGRRVATIEN AKQALELAFG WGPEYFNHVR 2863
DWV-B Slovenia 2019 341-1 (ON648741) LANLDKVSIE GTTNWTHARG LGRRVATIEN AKQALELAFG WGPEYFNHVR 2863
DWV-B USA 2018 EVR23 (OR361559) LANLDKVSIE GTTNWTHARG LGRRVATIEN AKQALELAFG WGPEYFNHVR 2863
DWV-B Austria 2018 B11B (OL803829) LANLDKVSIE GTTNWTHARG LGRRVATIEN AKQALELAFG WGPEYFNHVR 2863
DWV-B Austria 2022 SB22 (PP418870) LANLDKVSIE GTTNWTHARG LGRRVATIEN AKQALELAFG WGPEYFNHVR 2863
DWV-A Austria 2014 1414 (KU847397) LANLDKVSVE GTTNWTHARG LGRRTATIEN AKQALELAFG WGPEYFNYVR 2863
DWV-A Japan 2001 Kakugo (AB070959) LANLDKVSVE GTTNWTHARG LGRRAATIEN AKQALELAFG WGPEYFNYVR 2863
DWV-A USA 2003 PA (AY292384) LANLDKVSVE GTTNWTHARG LGRRTATIEN AKQALELAFG WGPEYFNYVR 2863
DWV-D Egypt 1977 EBV (MT504363) LANLDKVSVE GTTNWTHARG LGRRVATIEN AKQALELAFG WGPEYFNHIR 2866
DWV-C UK 2015 Devon (ER5657949) LQT------- ---------- ---------- ---------- ---------- 2816
Darwin bee virus 3 Austrailia 2014 NT-12 (MG995697) LANLDKRSVQ GAANWVHSKE LPLEEATIIN AKQCLELAYG WGPQYFGVVR 2770
Consensus LANLDKVSIE GTTNWTHARG LGRRVATIEN AKQALELAFG WGPEYFNHVR
Conservation 100% 0%
2.920
DWV-B USA 2018 EVR24 (OR361560) NTIKMAFDKL GIYEDLITWE EMDVRCYASA - 2893
DWV-B France 2019 VV144I (MN565037) NTIKMAFDKL GIYEDLITWE EMDVRCYASA - 2893
DWV-B Netherlands 2001 VDV-1 (NC_006494 NTIKMAFDKL GIYEDLITWE EMDVRCYASA - 2893
DWV-B Slovenia 2019 341-1 (ON648741) NTIKMAFDKL GIYEDLITWE EMDVRCYASA - 2893
DWV-B USA 2018 EVR23 (OR361559) NTIKMAFDKL GIYEDLITWE EMDVRCYASA - 2893
DWV-B Austria 2018 B11B (OL803829) NTIKMAFDKL GIYEDLITWE EMDVRCYASA - 2893
DWV-B Austria 2022 SB22 (PP418870) NTIKMAFDKL GIYEDLITWE EMDVRCYASA * 2894
DWV-A Austria 2014 1414 (KU847397) NTIKMAFDKL GIYEDLITWE EMDVRCYASA * 2894
DWV-A Japan 2001 Kakugo (AB070959) NTIKMAFDKL GIYEDLITWE EMDVRCYASA - 2893
DWV-A USA 2003 PA (AY292384) NTIKMAFDKL GIYEDLITWE EMDVRCYASA - 2893
DWV-D Egypt 1977 EBV (MT504363) NTIKMAFDKL GIYEDLLTWE EMDLRCYANA * 2897
DWV-C UK 2015 Devon (ER5657949) ---------- ---------- ---------- - 2816
Darwin bee virus 3 Austrailia 2014 NT-12 (MG995697) QKLIDAFRVK NIYIDLITWD EMDNRCFGDS - 2800
Consensus NTIKMAFDKL GIYEDLITWE EMDVRCYASA -
Conservation 100% 0%

Figure S4. Insertion of a molecular marker into the DWV-B genome. Honey bee pupae underwent either infection with the DWV-B field virus (P1 SB22), transfection with *in vitro* transcribed RNA of the genetically marked rDWV-B (rDWV-B-T_{9645}G), or mock transfection using PBS (Mock control). After three days of incubation, the pupae were harvested and homogenized. (A) Western blot analysis of VP1 expression. Total bee protein was resolved by SDS-PAGE, followed by blotting and probing using a VP1 specific Mab. While the mock control exhibited no signal, wtDWV and rDWV-B-T_{9645}G displayed clear signals indicative of productive infection. (B) Amplification of the marked genome region of rDWV-B by RT-PCR. Total RNA was extracted from bee lysates. A 1,531 bp region containing the 3′-end of the DWV-B genome was amplified by PCR and analyzed post-agarose gel electrophoresis. No PCR product was obtained from the mock transfected pupa. Reactions of wtDWV-B (P1 SB22) and the rDWV-B-T_{9645}G infected/transfected pupae showed an amplicon of the expected size. Weak additional bands are observed at 1.4 and 3.5 kb. (C) Application of the genetic marker. Aliquots of the PCR products were digested with XhoI and analyzed post-gel electrophoresis. The PCR amplicon of the wtDWV was completely fragmented by XhoI, resulting in distinct bands at about 1.0 and 0.5 kb, while the marked genome region of rDWV-B-T_{9645}G proved resistant to digestion.

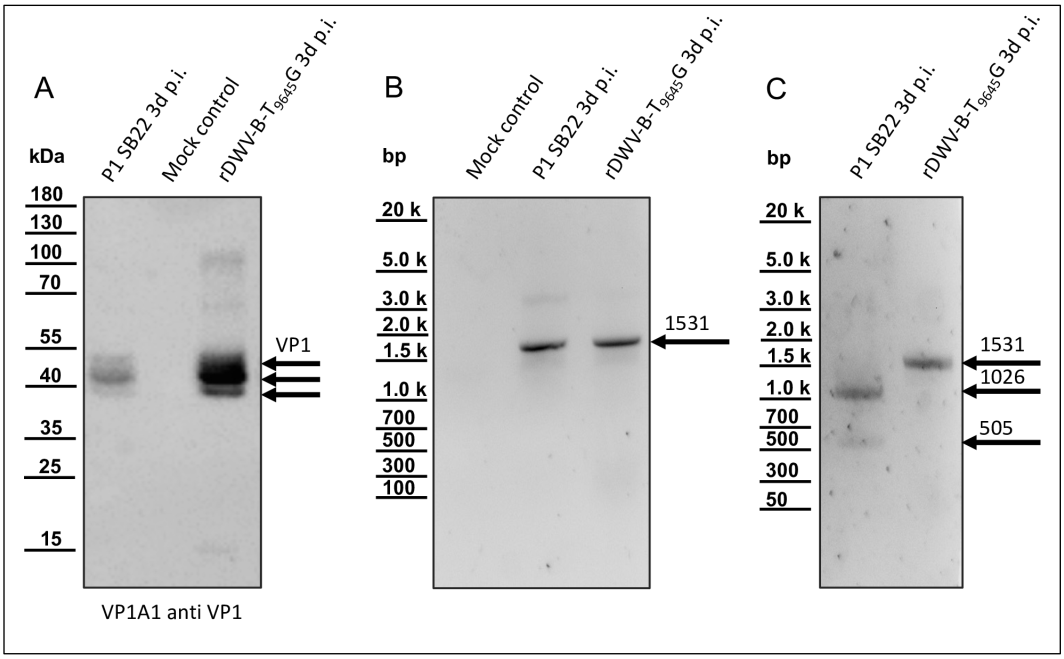

Figure S5. Primary culture of rDWV-B-GFP infected cells. Single cells extracted from both mock and rDWV-GFP transfected bee pupae were harvested post-evisceration. Primary cell cultures were grown in conditioned insect cell medium. After a 48-hour incubation period, cellular imaging was conducted to monitor the infection of rDWV-GFP at single cell level. In the mock transfected cells, no visible fluorescence was detected. Conversely, in cultures of rDWV-GFP transfected pupae, spherical suspension cells of varying sizes, along with larger adherent epithelial cells displaying characteristic GFP fluorescence, were observed.

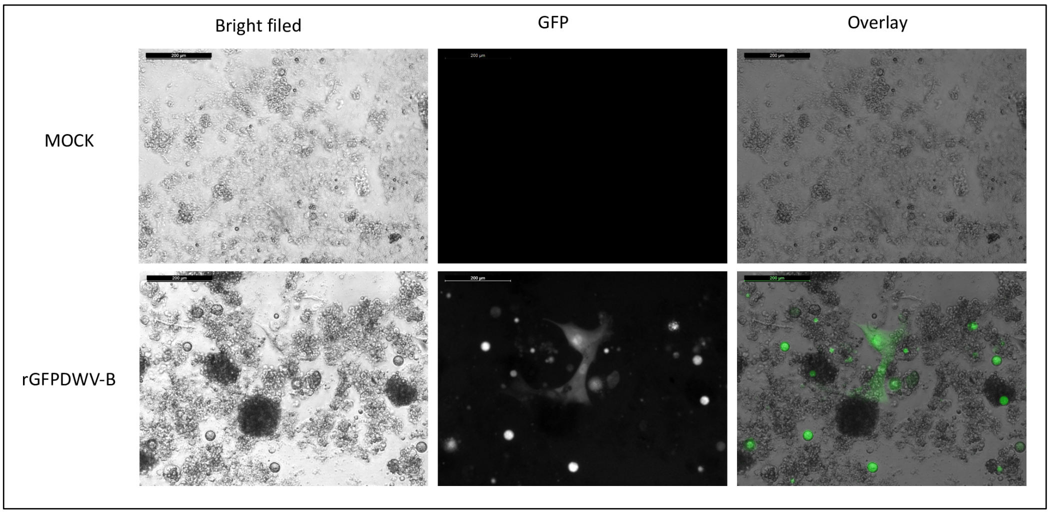

Table S1. Divergence of DWV nucleotide sequences. We compared the genomes of various DWV master variants in pairs, using complete functional genomes (strain 1414 of DWV-A and strain SB22 of DWV-B), reference strains (strain PA for DWV-A and strain VDV-1 for DWV-B), and available sequences for strain Devon (DWV-C) and strain EBV (DWV-D). Nucleotide identity percentages were determined using alignments, excluding the 5'-UTR sequences, which are unknown for most strains. Each percentage value is provided along with the number of matching nucleotides out of the total nucleotides used in the alignment (in parentheses).

DWV strains	DWV-A, strain 1414	DWV-A, strain PA	DWV-B, strain SB22	DWV-B, strain VDV-1	DWV-C, strain Devon	DWV-D, strain EBV
DWV-A, strain 1414	100%	98.254% (9961/10138)	84.262% (8545/10141)	84.301% (8549/10141)	79.491% (8054/10132)	80.765% (8196/10148)
DWV-A, strain PA		100%	84.346% (8551/10138)	84.395% (8556/10138)	79.200% (8023/10130)	80.761% (8194/10146)
DWV-B, strain SB22			100%	99.180% (10039/10122)	79.275% (8029/10128)	82.604% (8386/10152)
DWV-B, strain VDV-1				100%	79.228 (8025/10129)	82.634% (8389/10152)
DWV-C, strain Devon					100%	85.628% (8669/10124)
DWV-D, strain EBV						100%

Table S2. Divergence of DWV polyprotein sequences. We compared DWV master variant polyproteins using complete functional genomes (DWV-A strain 1414 and DWV-B strain SB22), reference strains (DWV-A strain PA and DWV-B strain VDV-1), and available sequences for strains Devon (DWV-C) and EBV (DWV-D). Amino acid sequence identity, homology, and summed similarity percentages were determined via alignments and presented along with the number of matching nucleotides out of the total used in the alignment (in parentheses).

DWV strains	DWV-A, strain 1414	DWV-A, strain PA	DWV-B, strain SB22	DWV-B, strain VDV-1	DWV-C, strain Devon	DWV-D, strain EBV
DWV-A, strain 1414	100%	Identity 99.205% (2871/2894) Homology = 0.415% (12/2894) Total = 99.620% (2883/2894)	Identity = 95.197% (2755/2894) Homology = 3.041% (88/2894) Total = 98.238% (2843/2894)	Identity = 95.266% (2757/2894) Homology = 2.903% (84/2894) Total = 98.169% (2841/2894)	Identity = 86.883% (2517/2897) Homology = 5.109% (148/2897) Total = 91.992% (2665/2897)	Identity = 89.921% (2605/2897) Homology = 5.350% (155/2897) Total = 95.271% (2760/2897)
DWV-A, strain PA		100%	Identity = 95.266% (2757/2894) Homology = 3.214% (93/2894) Total = 98.480% (2850/2894)	Identity = 95.403% (2760/2893) Homology = 3.076% (89/2893) Total = 98.479% (2849/2893)	Identity = 86.948% (2518/2896) Homology = 5.214% (151/2896) Total = 92.162% (2669/2896)	Identity = 89.852% (2603/2897) Homology = 5.523% (160/2897) Total = 95.375% (2763/2897)
DWV-B, strain SB22			100%	Identity = 99.654% (2884/2894) Homology = 0.207% (6/2894) Total = 99.862% (2890/2894)	Identity = 86.400% (2503/2897) Homology = 5.557% (161/2897) Total = 91.957% (2664/2897)	Identity = 90.542% (2623/2897) Homology = 5.316% (154/2897) Total = 95.858% (2777/2897)
DWV-B, strain VDV-1				100%	Identity = 86.464% (2504/2896) Homology = 5.559% (161/2896) Total = 92.023% (2665/2896)	Identity = 90.542% (2623/2897) Homology = 5.247% (152/2897) Total = 95.789% (2775/2897)
DWV-C, strain Devon					100%	Identity = 89.023% (2579/2897) Homology = 3.970% (115/2897) Total = 92.993% (2694/2897)
DWV-D, strain EBV						100%

Table S3. Genome equivalents of individual animals 3 days post transfection/infection.

RNA or virus	Average GE DWV-B/bee* (Average GE DWV-A/bee*)
Mock 1	n.d.* (n.d.)
Mock 2	n.d.* (n.d.)
Mock 3	n.d.* (n.d.)
wtDWV-B, SB22 (Virus) 1	1.2×10^{11} (n.d.)
wtDWV-B, SB22 (Virus) 2	1.8×10^{11} (n.d.)
wtDWV-B, SB22 (Virus) 3	1.1×10^{11} (n.d.)
RNA rDWV-B-E_{1102}Stop 1	1.2×10^{5} (n.d.)
RNA rDWV-B-E_{1102}Stop 2	9.2×10^{3} (n.d.)
RNA rDWV-B-E_{1102}Stop 3	3.1×10^{3} (n.d.)
P1 rDWV-B-E_{1102}Stop (Virus) 1	n.d. (n.d.)
P1 rDWV-B-E_{1102}Stop (Virus) 2	n.d. (n.d.)
P1 rDWV-B-E_{1102}Stop (Virus) 3	1.7×10^{3} (n.d.)
RNA rDWV-B 1	3.0×10^{10} (n.d.)
RNA rDWV-B 2	1.2×10^{10} (n.d.)
RNA rDWV-B 3	1.2×10^{10} (n.d.)
P1 rDWV-B (Virus) 1	1.3×10^{10} (n.d.)
P1 rDWV-B (Virus) 2	4.1×10^{11} (n.d.)
P1 rDWV-B (Virus) 3	7.4×10^{10} (n.d.)
RNA rDWV-A-Q_{2118}A 1	n.d. (2.4×10^{4})
RNA rDWV-A-Q_{2118}A 2	n.d. (9.9×10^{3})
RNA rDWV-A-Q_{2118}A 3	n.d. (2.5×10^{3})
P1 rDWV-A-Q_{2118}A (Virus) 1	n.d. (n.d.)
P1 rDWV-A-Q_{2118}A (Virus) 2	n.d. (n.d.)
P1 rDWV-A-Q_{2118}A (Virus) 3	n.d. (n.d.)
RNA rDWV-A 1	n.d. (8.6×10^{9})

RNA rDWV-A 2	n.d.<br>(3.2 x 10^{9})
RNA rDWV-A 3	n.d.<br>(8.0 x 10^{9})
P1 rDWV-A (Virus) 1	n.d.<br>(2.6 x 10^{10})
P1 rDWV-A (Virus) 2	n.d.<br>(1.0 x 10^{9})
P1 rDWV-A (Virus) 3	n.d.<br>(1.0 x 10^{9})

* Average titer of three individual bees, measured in three technical replicates

** n.d. means not detected
